# Single-cell copy number variant detection reveals the dynamics and diversity of adaptation

**DOI:** 10.1371/journal.pbio.3000069

**Published:** 2018-12-18

**Authors:** Stephanie Lauer, Grace Avecilla, Pieter Spealman, Gunjan Sethia, Nathan Brandt, Sasha F. Levy, David Gresham

**Affiliations:** 1 Center for Genomics and Systems Biology, Department of Biology, New York University, New York, New York, United States of America; 2 Department of Biology, New York University, New York, New York, United States of America; 3 Joint Initiative for Metrology in Biology, National Institute of Standards and Technology, Stanford University, Stanford, California, United States of America; Wageningen University, NETHERLANDS

## Abstract

Copy number variants (CNVs) are a pervasive source of genetic variation and evolutionary potential, but the dynamics and diversity of CNVs within evolving populations remain unclear. Long-term evolution experiments in chemostats provide an ideal system for studying the molecular processes underlying CNV formation and the temporal dynamics with which they are generated, selected, and maintained. Here, we developed a fluorescent CNV reporter to detect de novo gene amplifications and deletions in individual cells. We used the CNV reporter in *Saccharomyces cerevisiae* to study CNV formation at the *GAP1* locus, which encodes the general amino acid permease, in different nutrient-limited chemostat conditions. We find that under strong selection, *GAP1* CNVs are repeatedly generated and selected during the early stages of adaptive evolution, resulting in predictable dynamics. Molecular characterization of CNV-containing lineages shows that the CNV reporter detects different classes of CNVs, including aneuploidies, nonreciprocal translocations, tandem duplications, and complex CNVs. Despite *GAP1*’s proximity to repeat sequences that facilitate intrachromosomal recombination, breakpoint analysis revealed that short inverted repeat sequences mediate formation of at least 50% of *GAP1* CNVs. Inverted repeat sequences are also found at breakpoints at the *DUR3* locus, where CNVs are selected in urea-limited chemostats. Analysis of 28 CNV breakpoints indicates that inverted repeats are typically 8 nucleotides in length and separated by 40 bases. The features of these CNVs are consistent with origin-dependent inverted-repeat amplification (ODIRA), suggesting that replication-based mechanisms of CNV formation may be a common source of gene amplification. We combined the CNV reporter with barcode lineage tracking and found that 10^2^–10^4^ independent CNV-containing lineages initially compete within populations, resulting in extreme clonal interference. However, only a small number (18–21) of CNV lineages ever constitute more than 1% of the CNV subpopulation, and as selection progresses, the diversity of CNV lineages declines. Our study introduces a novel means of studying CNVs in heterogeneous cell populations and provides insight into their dynamics, diversity, and formation mechanisms in the context of adaptive evolution.

## Abstract 2

A fluorescent reporter system reveals that copy number variants (CNVs) are repeatedly generated and selected during the early stages of adaptive evolution, resulting in initially predictable dynamics with thousands of independent CNV-containing lineages competing within populations.

## Author summary

Duplications and deletions of genomic sequence, known as copy number variants, are a common source of genetic diversity across all domains of life. Copy number variants play a crucial role in driving evolutionary processes but can also cause genetic disease and cancer. Although copy number variants are important drivers of diversity, adaptation, and disease, the underlying dynamics of their formation and selection are poorly understood. Copy number variants are difficult to detect, especially when present at low frequencies in heterogenous evolving populations. To overcome this challenge, we developed a novel fluorescent reporter that allows us to visualize copy number variants as they emerge and to track them throughout hundreds of generations of laboratory evolution. We show that copy number variants arise early and repeatedly, that they are diverse in size and copy number, and that they are generated at a high rate, leading to competition among cells containing different copy number variants. Molecular characterization of copy number variants indicates that many of them are likely generated by errors during DNA replication. This method is broadly applicable to studying the molecular mechanisms underlying formation of copy number variants, as well as their role in driving evolutionary processes and cancer.

## Introduction

Copy number variants (CNVs) drive rapid adaptive evolution in diverse scenarios ranging from niche specialization to speciation and tumor evolution [1–4]. CNVs, which include duplications and deletions of genomic segments, underlie phenotypic diversity in natural populations [5–10] and provide a substrate for evolutionary novelty through modification of existing heritable material [11–14]. Beneficial CNVs are associated with defense against disease in plants, increased nutrient transport in microbes, and drug-resistant phenotypes in parasites and viruses [9,15–18]. Despite the importance of CNVs for phenotypic variation, evolution, and disease, the dynamics with which these alleles are generated and selected in evolving populations are not well understood.

Long-term experimental evolution provides an efficient means of gaining insights into evolutionary processes using controlled and replicated selective conditions [19,20]. Chemostats are devices that maintain cells in a constant nutrient-poor growth state using continuous culturing [21]. Nutrient limitation in chemostats provides a defined and strong selective pressure in which CNVs have been repeatedly identified as major drivers of adaptation. CNVs containing the gene responsible for transporting the limiting nutrient are repeatedly selected in a variety of organisms and conditions including *Escherichia coli* limited for lactose [22], *Salmonella typhimurium* in different carbon source limitations [23], and *Saccharomyces cerevisiae* in glucose-, phosphate-, sulfur-, and nitrogen-limited chemostats [24–30]. CNVs confer large selective advantages, and multiple, independent CNV alleles have been identified within experimental evolution populations [25–27,31]. These findings suggest that CNVs are generated at a high rate, but estimates differ greatly, ranging from 1 × 10^−10^ to 3.4 × 10^−6^ duplications per cell per division, with variation in CNV formation rates potentially differing between loci and/or condition [32,33]. A high rate of CNV formation suggests that multiple, independent CNV-containing lineages may compete during adaptive evolution, resulting in clonal interference, which is characteristic of large, evolving populations [29,34–36]. However, the extent to which clonal interference among CNV-containing lineages influences the dynamics of adaptation is unknown.

The general amino acid permease gene, *GAP1*, is well suited to studying the role of CNVs in adaptive evolution. *GAP1* encodes a high-affinity transporter for all naturally occurring amino acids, and it is highly expressed in nitrogen-poor conditions [37,38]. We have previously shown that two classes of CNVs are selected at the *GAP1* locus in *S*. *cerevisiae* when a sole nitrogen source is provided: *GAP1* amplification alleles are selected in glutamine and glutamate-limited chemostats, and *GAP1* deletion alleles are selected in urea- and allantoin-limited chemostats [24,25]. *GAP1* CNVs are also found in natural populations. In the nectar yeast *Metschnikowia reukaufii*, multiple tandem copies of *GAP1* result in a competitive advantage over other microbes when amino acids are scarce [39]. As a target of selection in adverse environments in both experimental and natural populations, *GAP1* is a model locus for studying the dynamics and mechanisms underlying both gene amplification and deletion in evolving populations.

CNVs are generated by two primary classes of mechanisms: homologous recombination and DNA replication [40–42]. DNA double-strand breaks (DSBs) are typically repaired by homologous recombination and do not result in CNV formation. However, nonallelic homologous recombination (NAHR) can generate CNVs when the incorrect repair template is used, which occurs more often with repetitive DNA sequences such as transposable elements and long terminal repeats (LTRs) [43]. During DNA replication, stalled and broken replication forks can reinitiate DNA replication through processes including break-induced replication (BIR), microhomology-mediated break-induced replication (MMBIR), and fork stalling and template switching (FoSTes) [44–46]. BIR is driven by homologous sequences, whereas MMBIR relies on shorter stretches of sequence homology. Recently, origin-dependent inverted-repeat amplification (ODIRA) has been identified as a novel mechanism underlying amplification of the *SUL1* locus in yeast [47,48]. ODIRA is mediated by short inverted repeat sequences that facilitate ligation of the leading and lagging strands following regression of the replication fork during DNA synthesis. ODIRA is hypothesized to involve the formation of an extrachromosomal circular intermediate that replicates independently and therefore requires an origin of replication within the amplified region. Subsequent integration of the circle into the original locus via homologous recombination results in an inverted triplication. Extrachromosomal circular DNA is common in yeast [49], can drive tumorigenesis [50], and may represent a rapid and reversible mechanism of generating adaptive CNVs [51,52]. Previously, we found that some *GAP1* amplifications are extrachromosomal circular elements. We hypothesized that *GAP1*^circle^ alleles are generated as a result of NAHR between flanking LTRs, resulting in their excision from the chromosome [25]. Identifying the mechanisms underlying CNV formation is required for understanding the roles of CNVs in evolutionary processes and human disease.

A key limitation to the study of CNVs in evolving populations is the challenge of identifying them at low frequencies in heterogeneous populations. CNVs are typically detected using molecular methods including quantitative PCR (qPCR), Southern blotting, DNA microarrays, and sequencing [24–26]. However, using any of these methods, de novo CNVs are undetectable in a heterogeneous population until present at high frequency (e.g., >50%). This precludes analysis of the early dynamics with which CNVs emerge and compete in evolving populations. As CNVs usually comprise genomic regions that include multiple neighboring genes [24], we hypothesized that CNVs could be identified on the basis of increased expression of a constitutively expressed fluorescent reporter gene inserted adjacent to a target gene of interest. A major benefit of this approach is that it detects CNVs independently of whole-genome sequencing, enabling a high-resolution and efficient assay of CNV dynamics with single-cell resolution in evolving populations.

In this study, we constructed strains containing a fluorescent CNV reporter adjacent to *GAP1* in *S*. *cerevisiae* and performed evolution experiments in different selective environments using chemostats. The CNV reporter allowed us to visualize selection of CNVs at the *GAP1* locus in real time with unprecedented temporal resolution. We find that CNV dynamics occur in two distinct phases: CNVs are selected early during adaptive evolution and quickly rise to high frequencies, but the subsequent dynamics are complex. We find that *GAP1* CNVs are diverse in size and copy number and can be generated by a range of processes including aneuploidy, nonreciprocal translocations, and tandem duplication by NAHR. Nucleotide resolution analysis of *GAP1* CNV breakpoints revealed that CNV formation is mediated by short, interrupted inverted repeats for half of the resolvable cases, suggesting that replication-based mechanisms also underlie gene amplification at the *GAP1* locus. The presence of inverted repeats, in combination with a replication origin and inverted triplication, is consistent with *GAP1* CNV formation through ODIRA. ODIRA may be a major source of de novo CNVs in yeast, as these breakpoint features also characterize CNVs at an additional locus identified in our study, *DUR3*. To determine the underlying structure of the CNV subpopulation, we generated a lineage-tracking library using random DNA barcodes. Fluorescence-activated cell sorting (FACS)-based fractionation of CNV lineages and barcode sequencing identified hundreds to thousands of individual CNV lineages within populations, consistent with a high CNV supply rate and extreme clonal interference. Together, our results show that CNVs are generated repeatedly by diverse processes, resulting in predictable dynamics, but that the long-term fate of CNV-containing lineages in evolving populations is shaped by clonal interference and additional variation.

## Results

### Protein fluorescence increases proportionally with gene copy number

We sought to construct a reporter for CNVs that occur at a given locus of interest. Based on previous studies [53–56], we hypothesized that CNVs that alter the number of copies of a constitutively expressed fluorescent protein gene would facilitate single-cell detection of de novo copy number variation. To test the feasibility of this approach, we constructed haploid *S*. *cerevisiae* strains isogenic to the reference strain (S288c) with one or two copies of a constitutively expressed green fluorescent protein (GFP) variant mCitrine [57] and diploid strains with 1–4 copies of mCitrine integrated into the genome (S1 Table).

**Fig 1:**
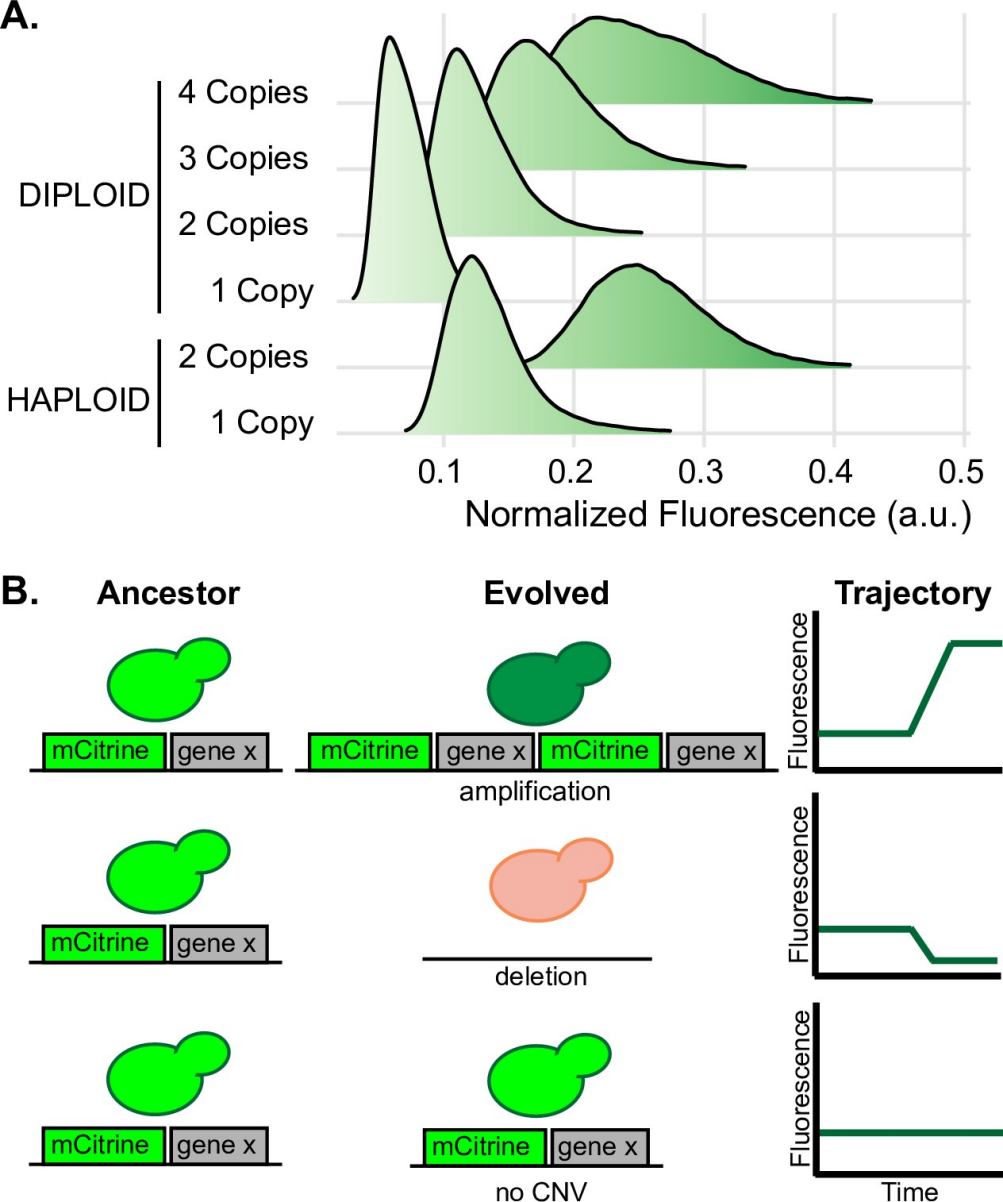
Fluorescent protein signal is proportional to gene copy number. (A) Protein fluorescence increases with increasing copies of the mCitrine gene. We determined the fluorescence of haploid and diploid cells containing variable numbers of a constitutively expressed mCitrine gene integrated at either the *HO* locus and/or the dubious ORF, *YLR123C*. The two-copy diploid is heterozygous at both loci. Each distribution was estimated using 100,000 single-cell measurements normalized by forward scatter. (B) Schematic representation of how the fluorescent reporter enables CNV detection in heterogeneous evolving populations through quantitative changes in protein fluorescence. Data and computer code used to generate this figure can be accessed in OSF: https://osf.io/fxhze/. a.u., arbitrary units; CNV, copy number variant.

Flow cytometry analysis confirmed that additional copies of mCitrine produce quantitatively distinct distributions of protein fluorescence (Fig 1A). Haploid cells with two copies of mCitrine have higher fluorescence than those with a single copy, and there is minimal overlap between the distributions of fluorescent signal in the two strains. Normalization of the fluorescent signal by forward scatter, which is correlated with cell size, shows that the concentration of fluorescent protein is proportional to the ploidy normalized copy number of the mCitrine gene (i.e., one copy in a haploid results in a signal equivalent to two copies in a diploid, and two copies in a haploid results in a signal similar to four copies in a diploid). Thus, the cell size–normalized fluorescent signal, or concentration, accurately reports on the number of copies of the fluorescent gene in single cells. Therefore, integrating a constitutively expressed fluorescent protein gene proximate to an anticipated target of selection functions as a CNV reporter for tracking gene amplifications and deletions in evolving populations (Fig 1B).

### A CNV reporter tracks the dynamics of *GAP1* CNVs in real time

Previous work has shown that spontaneous *GAP1* amplifications are positively selected when glutamine is the sole limiting nitrogen source during evolution experiments in chemostats [25]. *GAP1* copy number amplifications result in increased amino acid transporters on the plasma membrane, providing cells with a selective advantage when nitrogen is scarce [24,25]. Conversely, *GAP1* deletions provide a fitness benefit and are selected in urea-limited conditions [25], which may be due to two nonexclusive reasons: either (1) because *GAP1* is highly expressed regardless of the type of limiting nitrogen source [58] but unable to transport urea, it confers a gene expression burden; or (2) when the extracellular concentration of amino acids is low compared to the intracellular concentration, the electrochemical gradient drives their export through the GAP1 permease. Thus, the use of different nitrogen sources in nitrogen-limited chemostats enables the study of both *GAP1* amplification and deletion, making it an ideal system for studying the dynamics of CNV selection in evolving populations.

We constructed a haploid strain containing a mCitrine CNV reporter located 1,118 bases upstream of the *GAP1* start codon to ensure that the native regulation of *GAP1* was unaffected [59]. We inoculated the *GAP1* CNV reporter strain into 9 glutamine-, 9 urea-, and 8 glucose-limited chemostats for a total of 26 populations (S2 Table). For each of the three selection conditions, we included two control populations: one containing a single copy of the mCitrine CNV reporter at a neutral locus (one copy control) and one containing two copies of the mCitrine CNV reporter at two neutral loci (two copy control). All populations were maintained in continuous mode (dilution rate = 0.12 culture volumes/hour; population doubling time = 5.8 hours) for 267 generations over 65 days. We sampled each of the 32 populations every 8 generations and used flow cytometry to measure fluorescence of 100,000 cells per sample.

**Fig 2:**
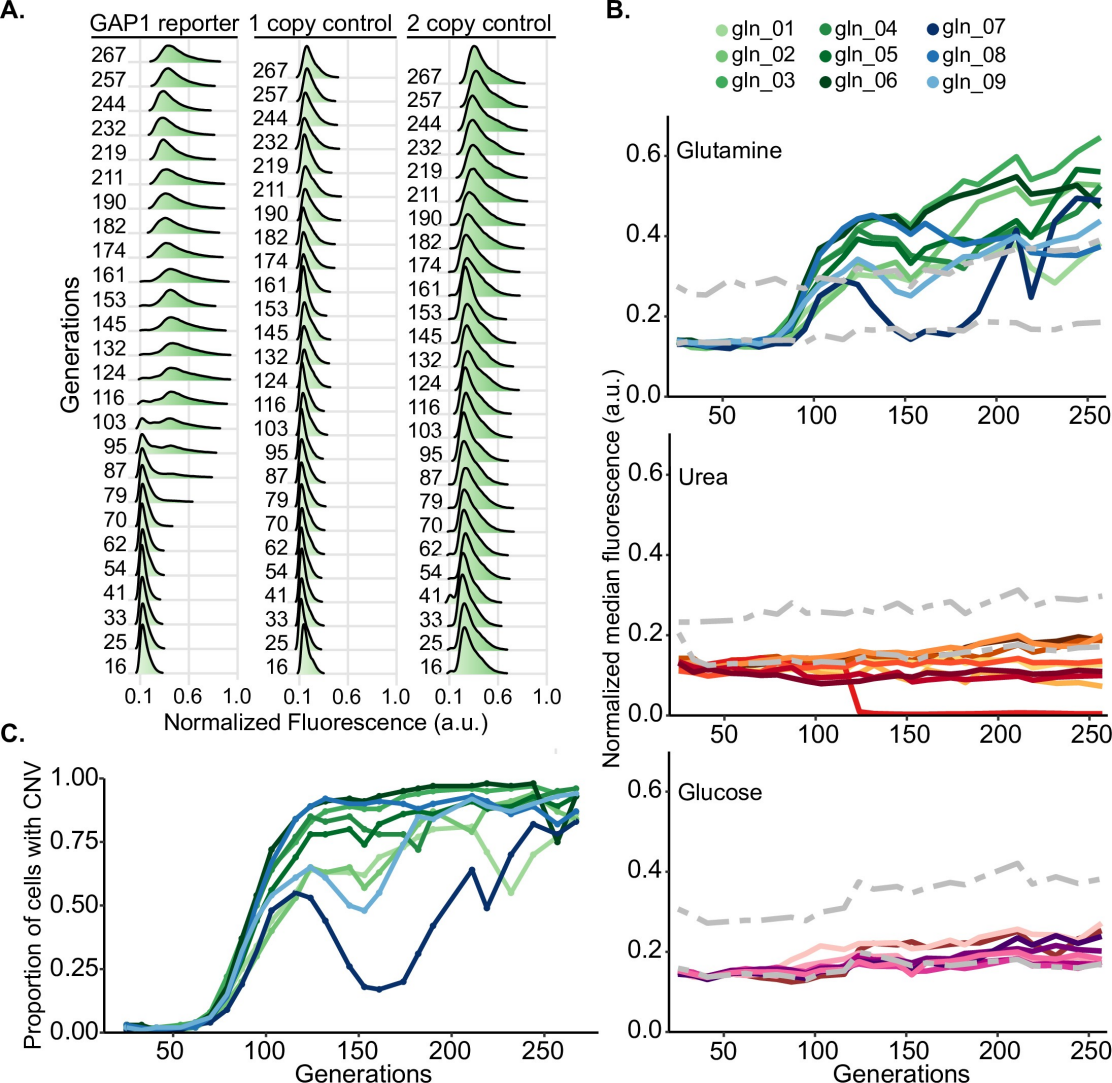
Dynamics of *GAP1* CNVs in evolving populations. (A) Normalized distributions of single-cell fluorescence over time for a representative *GAP1* CNV reporter strain and one- and two-copy control strains evolving in glutamine-limited chemostats. Single-cell fluorescence is normalized by the forward scatter measurement of the cell. (B) Normalized median fluorescence for each population evolving in glutamine- (*n* = 9), urea- (*n* = 9), and glucose-limited (*n* = 8) chemostats. The fluorescence of the one- and two-copy control strains is plotted for reference (gray dotted lines). (C) Estimates of the proportion of cells with *GAP1* amplifications over time for nine glutamine-limited populations containing the *GAP1* CNV reporter. Data and computer code used to generate this figure can be accessed in OSF: https://osf.io/fxhze/. a.u., arbitrary units; CNV, copy number variant.

Experimental evolution in a glutamine-limited chemostat resulted in clear increases in fluorescence in individual cells containing the *GAP1* CNV reporter by generation 79 (Fig 2A). By contrast, populations containing one or two copies of mCitrine at neutral loci exhibited stable fluorescence for the duration of the experiment (Fig 2A). Maintenance of protein fluorescence in one- and two-copy control populations is consistent with the absence of a detectable fitness cost associated with one or two copies of the CNV reporter in glutamine-limited chemostats, which we confirmed using competition assays (S1 Fig). Analysis of eight additional independent populations evolving in glutamine-limited chemostats showed qualitatively similar dynamics of single-cell fluorescence over time (S2 Fig). To summarize the dynamics of CNVs in evolving populations, we determined the median normalized fluorescence in each population at each time point. The fluorescent signal of the *GAP1* CNV reporter increases during selection in all populations evolving in glutamine-limited chemostats (Fig 2B), consistent with the de novo generation and selection of CNVs at the *GAP1* locus in all 9 populations.

Populations evolving in urea-limited and glucose-limited chemostats do not show substantial changes in fluorescence, with one exception (Fig 2B). In a single urea-limited population (ure_05), we detected a complete loss of fluorescent signal by generation 125, indicating the occurrence of a *GAP1* deletion that subsequently swept to fixation. Thus, the *GAP1* CNV reporter detects both amplification and deletion alleles at the *GAP1* locus in evolving populations. The absence of increases or decreases in fluorescence in all glucose-limited populations is consistent with the absence of selection for *GAP1* CNVs in conditions that are irrelevant for *GAP1* function.

To quantify the proportion of cells containing a *GAP1* duplication, we used one- and two-copy control strains to define flow cytometry gates. We found that the fluorescence of control strains varied slightly (S3A Fig), which may be indicative of either instrument variation or changes in cell physiology and morphology during the experiment, as suggested by systematic changes in forward scatter with time (S3B Fig). Using a conservative method to classify individual cells containing *GAP1* amplifications (Methods), we find that *GAP1* amplification alleles are selected with remarkably reproducible dynamics in the nine glutamine-limited populations (Fig 2C). CNVs are predominantly duplications (two copies), but quantification of fluorescence suggests that many cells contain three or more copies of the *GAP1* locus (S4 Fig).

We quantified the dynamics of CNVs in each population evolved in glutamine-limited chemostats using metrics defined by Lang and colleagues [60]. CNVs are detected by generation 70–75 (average = 72.8) in all 9 populations (T_up_) (Table 1). To estimate the fitness of all CNV lineages relative to the mean population fitness, we calculated S_up_, the rate of increase in the abundance of the CNV subpopulation (see Methods and S1 Text). The average relative fitness of the CNV subpopulation is 1.077 (S_up_), and CNV alleles are at frequencies greater than 75% in all populations by 250 generations (Table 1). Thus, in all replicated glutamine-limited selection experiments, *GAP1* amplifications emerge early, increase in frequency rapidly, and are maintained in each population throughout the selection.

**Table 1:** Summary statistics of *GAP1* CNV dynamics in glutamine-limited chemostats. T_up_ is the number of elapsed generations before CNVs are reliably detected (>7% frequency, see Methods). S_up_ is the rate of increase in CNV abundance during the initial expansion of the CNV subpopulation (S1 Text). The frequency of CNVs in the population at generation 150 and generation 250, when genome sequencing was performed, is also reported. Data and computer code used to generate this table can be accessed in OSF: https://osf.io/fxhze/.

Population	T_up_	1 + S_up_ ± SE	g150%	g250%
gln_01	70	1.066 ± 0.0038	62	77
gln_02	75	1.071 ± 0.0034	57	87
gln_03	70	1.071 ± 0.0037	88	94
gln_04	70	1.079 ± 0.0036	80	95
gln_05	75	1.077 ± 0.0041	74	89
gln_06	70	1.082 ± 0.0043	91	75
gln_07	75	1.094 ± 0.0048	18	78
gln_08	75	1.090 ± 0.0052	90	82
gln_09	75	1.066 ± 0.0050	48	93
AVG ± SD	72.8 ± 2.6	1.077 ± 0.01	68 ± 24	86 ± 8

Abbreviations: AVG, average; CNV, copy number variant; SD, standard deviation.

*GAP1* CNVs undergo two distinct phases of population dynamics. The initial dynamics with which CNV subpopulations emerge and increase in frequency are highly reproducible in independent evolving populations. However, after 125 generations, the trajectories of the CNV subpopulation in the different replicate populations diverge. Many populations maintain a high frequency of *GAP1* amplification alleles, but in some populations, they decrease in frequency. In one population, *GAP1* CNV alleles are nearly lost from the population before subsequently increasing to an appreciable frequency (gln_07).

### *GAP1* CNV alleles are diverse within and between replicate populations

**Fig 3:**
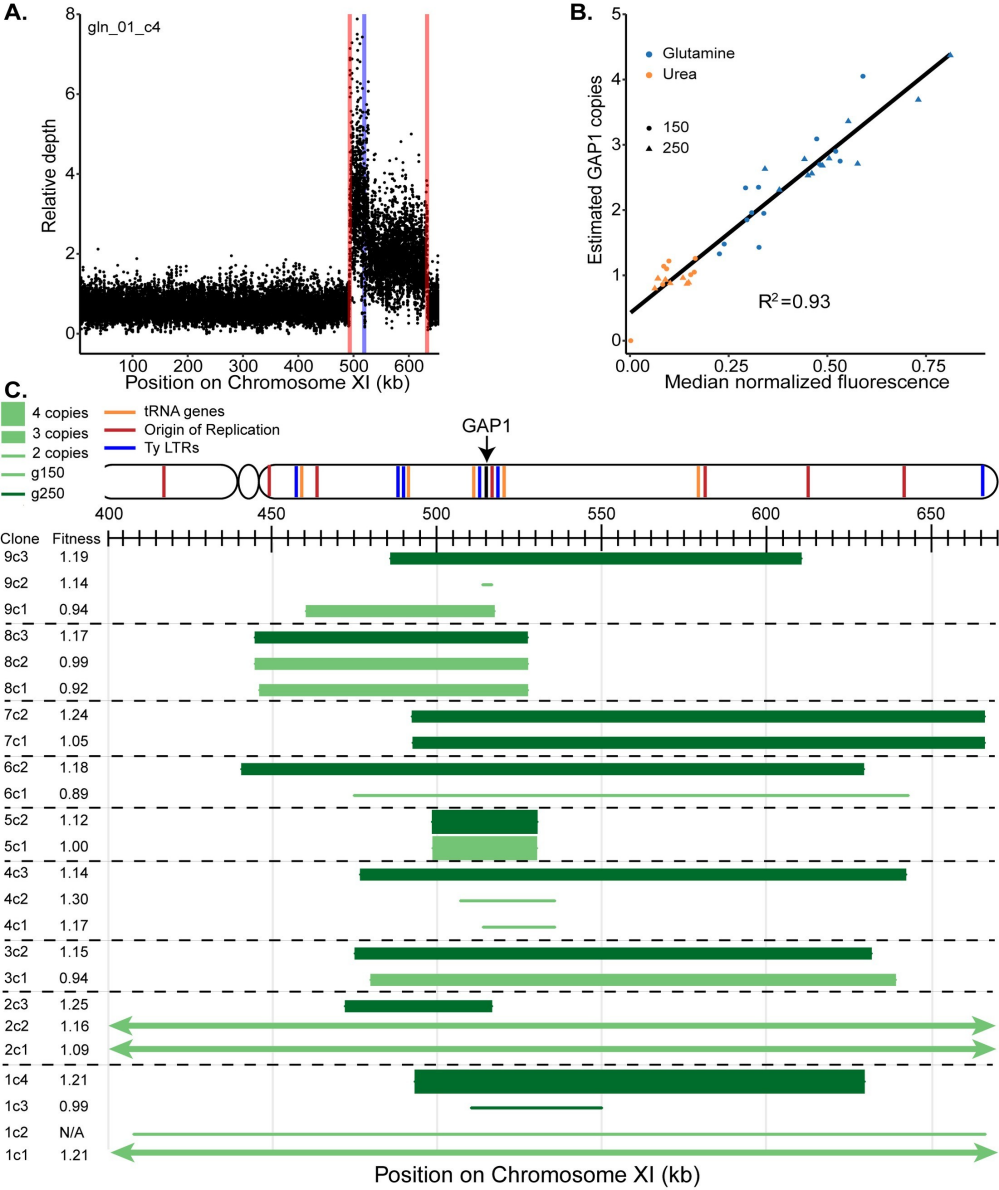
Diversity and fitness effects of *GAP1* CNVs. (A) Representative sequence read depth plot from a glutamine-limited clone (gln_01_c4). The nucleotide coordinates of *GAP1* in our CNV reporter strain are Chromosome XI: 518438–520246 (blue line). Estimated breakpoint boundaries are shown in red. Read depth was normalized to the average read depth on Chromosome XI. Reads at each nucleotide position were randomly downsampled for presentation purposes. (B) Read depth–based estimates of *GAP1* copy number are positively correlated with median fluorescence of glutamine-limited clones, indicating that fluorescence is informative about the copy number of de novo CNVs. (C) Schematic representation of CNVs identified in clones isolated from glutamine-limited populations. The relative fitness of each clone is also indicated. Copy number and CNV boundaries were estimated using read depth. This schematic is simplified for presentation purposes: the reported copy number refers specifically to the *GAP1* coding sequence and does not necessarily reflect copy number throughout the entire CNV, which may vary. For read depth measurements across the entirety of Chromosome XI, see S2 Text. Data and computer code used to generate this figure can be accessed in OSF: https://osf.io/fxhze/. CNV, copy number variant; LTR, long terminal repeat; N/A, not applicable; g150, generation 150; g250, generation 250.

Based on prior studies [24,26], we hypothesized that multiple CNV alleles exist within each population. To characterize the diversity of *GAP1* CNVs, we isolated a total of 29 clones containing increased fluorescence from glutamine-limited chemostats at 150 and 250 generations for whole-genome sequencing (S3 Table). We used read depth to calculate *GAP1* copy number and to estimate CNV boundaries (Fig 3A, S4 Table, and Methods). We find that *GAP1* copy number estimated by sequencing read depth correlates with the fluorescent signal for individual clones (Fig 3B), indicating that fluorescent signal is predictive of copy number. In 3 clones, we find increased read depth across the entirety of Chromosome XI consistent with aneuploidy. Thus, the CNV reporter is able to detect aneuploid chromosomes as well as subchromosomal CNVs.

We identified diverse *GAP1* CNVs between and within populations (Fig 3C). In the majority of populations (6/9), different clones had different CNVs. For example, in population gln_01 at generation 150, we identified a large *GAP1* CNV that includes the entire right arm of Chromosome XI and another clone that was aneuploid for Chromosome XI. At generation 250, clones isolated from population gln_01 have CNV alleles that are distinct from each other and from those observed at generation 150. Clones from the 8 additional glutamine-limited populations show evidence for CNV diversity within and between the two time points analyzed (Fig 3C), suggesting the presence of multiple CNV lineages within evolving populations. Furthermore, the diversity of *GAP1* CNVs indicates that they are not predominantly formed through a recurrent mechanism as might be anticipated by the presence of proximate repetitive elements.

We used pulsed-field gel electrophoresis and Southern blotting to confirm CNV structures (S5 Fig). Using *GAP1* and *CEN11* probes for Southern blotting, we identified size shifts in some samples consistent with the large CNVs (>140 kilobases) we identified in several clones. In some cases, we identified two discrete bands in our *GAP1* Southern blot, indicating that the additional copies of *GAP1* were not contained on Chromosome XI. The *GAP1* Southern also provided further evidence for the *GAP1* deletion in a clone isolated from urea limitation. Whereas control populations evolving in glutamine-limited chemostats did not show evidence for *GAP1* CNVs on the basis of fluorescence, sequence and Southern blotting analysis identified *GAP1* amplifications in lineages isolated from these populations (S2 Text and S5 Fig). As one- and two-copy control strains do not have the *GAP1* CNV reporter, this suggests that *GAP1* CNV formation and selection are not affected by the reporter. Moreover, we find no evidence that the molecular features of *GAP1* CNVs are affected by the presence of the CNV reporter.

We determined the fitness of *GAP1* CNV-containing clones using pairwise competitive fitness assays in glutamine-limited chemostats (S6 Fig and Fig 3C). Four independent competition assays with the ancestral strain containing the *GAP1* CNV reporter showed no significant differences in fitness compared to the isogenic nonfluorescent reference strain. The majority of evolved clones (18/28) have higher relative fitness than the ancestor, indicating that *GAP1* CNVs typically confer large fitness benefits. Several clones have neutral (8/28) or lower (2/28) relative fitness, which indicates that either (1) the fitness effect of *GAP1* CNVs may be context specific or (2) not all *GAP1* CNVs confer a fitness benefit.

### *DUR3* CNVs are repeatedly selected during urea limitation

We analyzed the genome sequences of 21 clones that were randomly isolated from urea-limited populations at generation 150 and generation 250 and identified multiple CNVs at the *DUR3* locus (S7A Fig and S2 Text). *DUR3* encodes a high-affinity urea transporter, and we have previously reported *DUR3* amplifications during experimental evolution in a urea-limited chemostat [24]. We compared properties of *GAP1* and *DUR3* amplifications and found that the average copy number for clones with *GAP1* CNVs is 3 (S7B Fig), whereas clones with *DUR3* CNVs contain significantly more copies, with an average copy number of 5 (S7C Fig, *t* test, *p*-value < 0.01). Copy number within clones does not significantly increase between 150 and 250 generations at either locus. *DUR3* CNV alleles (average of 26 kilobases) are also significantly smaller than *GAP1* CNVs (average of 105 kilobases) (S7D–S7E Fig, *t* test, *p*-value < 0.01). Thus, comparison of *GAP1* and *DUR3* CNVs suggests differences in the properties of selected CNVs as a function of locus and selective condition.

### CNV breakpoints are characterized by short, interrupted inverted repeats

To resolve CNV breakpoint sequences, we generated a pipeline integrating CNV calls from multiple existing CNV detection methods (CNVnator, Pindel, LUMPY, and SvABA [61–64]) and optimized their performance on synthetic yeast genome data (S3 Text) simulating both clonal (S8 Fig) and heterogeneous populations (S9 Fig). Although these algorithms perform well using simulated data, we found that they had a high false positive and false negative rate when applied to real data (S5 Table and S6 Table) and, in general, were not informative about the novel sequence formed at CNV boundaries. Therefore, we developed a breakpoint detection pipeline that integrates information from read depth, discordant reads, and split reads. To define the breakpoint sequence, we performed de novo assembly using split reads and aligned the resulting contig against the reference genome (Methods). In addition to *GAP1* and *DUR3* CNVs, we identified 3 structural variants in our clonal sequencing data using this method (S7 Table). A read depth–based approach was also used to characterize CNVs genome-wide (S8 Table) and calculate ribosomal DNA (rDNA) and *CUP1* copy number, which exhibit variation among lineages (S4 Table).

We analyzed 29 lineages containing *GAP1* CNVs and inferred the underlying mechanisms for 19 (66%) of them on the basis of copy number and breakpoint sequences (Methods). Of the 19 *GAP1* CNVs that can be reliably resolved, 3 are the result of aneuploidies and 2 are the result of nonreciprocal interchromosomal translocations (S5 Table). Translocations were confirmed using pulsed-field gel electrophoresis and Southern blot analysis (S5 Fig), which clearly shows that the second copy of *GAP1* is located on a different chromosome. Southern blotting also indicates that an additional 3 *GAP1* CNVs are the result of partial (i.e., segmental) aneuploidies, which include the Chromosome XI centromere (*CEN11*) but are smaller than the ancestral Chromosome XI (S5 Fig). At least 4 *GAP1* CNVs appear to be the result of a tandem duplication mediated by NAHR. For two of these CNVs, novel junction sequences were obtained that included a hybrid sequence composed of half of each flanking LTR (*YKRCdelta11*/*YKRCdelta12*), similar to our previous report [25]. This mechanism is also likely to underlie the *GAP1* deletion that we identified in one urea-limited population.

**Fig 4:**
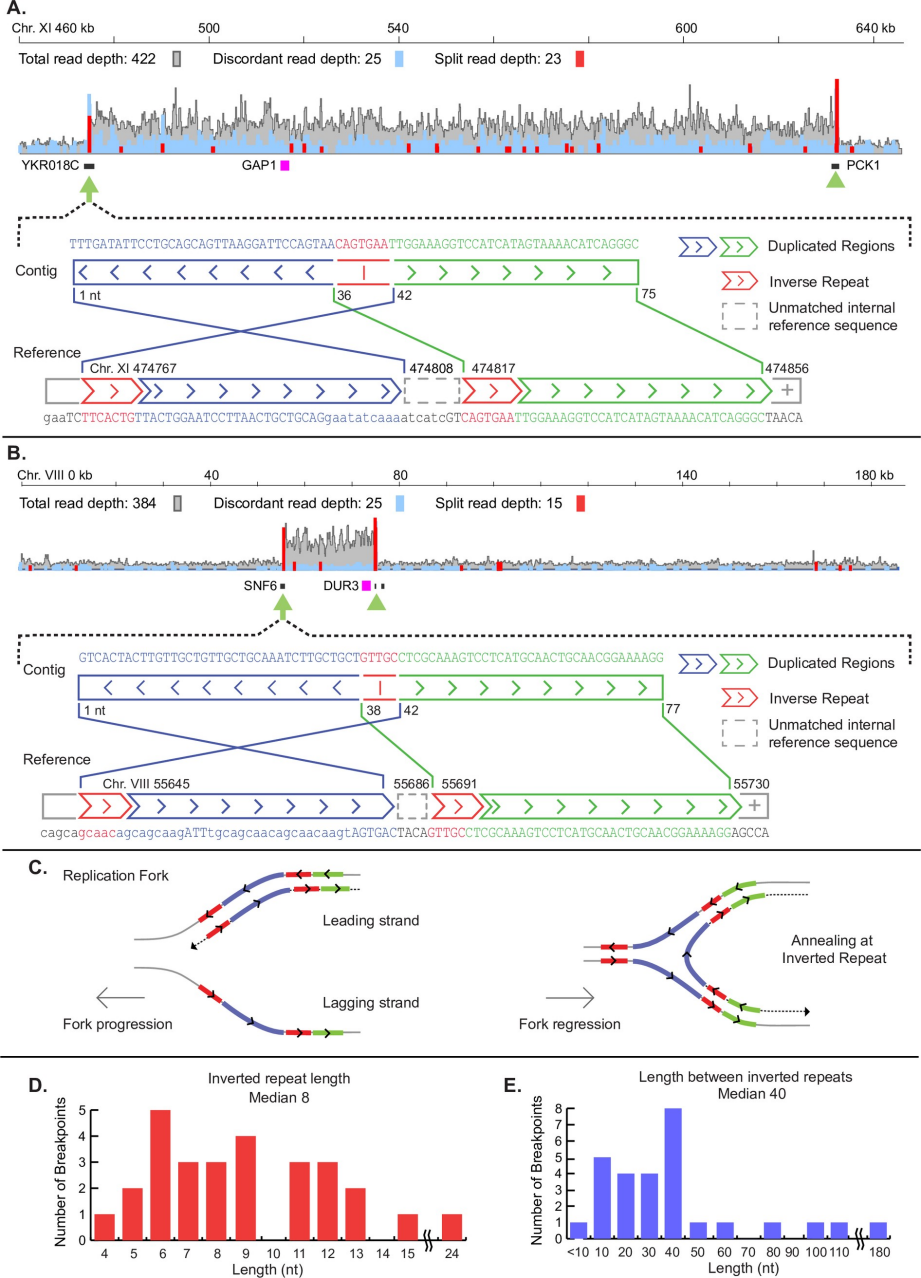
Inverted repeats mediate CNV formation. Nucleotide (“nt”) resolution of CNV breakpoints for (A) *GAP1* and (B) *DUR3* CNVs were identified using a combination of discordant and split reads. To characterize novel sequence, we identified all supporting split reads, performed de novo assembly, and aligned the resulting sequence against the reference genome. Sequences in the reference genome (blue) are inversely oriented in the assembled contig, suggesting an inverted structure within CNVs. (C) Schematic representation of replication-based CNV formation. After fork stalling, fork regression results in the newly replicated inverted repeat sequence annealing to the complementary sequence and ligating to the lagging strand. (D–E) Distribution of sequence features across 28 breakpoints at the *GAP1* and *DUR3* loci that contain inverted repeats. Data and computer code used to generate this figure can be accessed in OSF: https://osf.io/fxhze/. CNV, copy number variant.

For 12 out of 29 (41%) *GAP1* CNVs and 8 out of 9 (89%) *DUR3* CNVs, we identified a pair of short, interrupted, inverted repeats proximate to at least one breakpoint (Fig 4 and S2 Text). We were able to resolve breakpoints at both ends of the CNV for 12 of the 20 CNVs. Analysis of these breakpoints indicates that inverted repeat sequences range in length from 4 to 24 base pairs (Fig 4D) and are typically separated by 40 base pairs (Fig 4E). Microhomology at breakpoint junctions is characteristic of replication-based CNV formation, including MMBIR and ODIRA. ODIRA has several other requirements, including the presence of at least one replication origin within the CNV, an internal inversion, and an odd copy number. The identification of inverted sequence relative to the reference at all identified breakpoint junctions is consistent with an inverted structure. We find that 6/29 *GAP1* CNVs and 8/9 *DUR3* CNVs meet these criteria and thus are likely the result of ODIRA. In cases when the CNV lacks an odd copy number (see Methods) we cannot reliably infer the mechanism (S5 Table). In one case (ure_07_c1), the CNV meets all the requirements of ODIRA but does not contain a DNA replication origin (see Discussion).

### Whole-genome population sequencing provides insight into population heterogeneity

To comprehensively characterize genomic variation in populations, we performed whole-population, whole-genome sequencing of glutamine-, urea-, and glucose-limited populations at generations 150 and 250 (S3 Table). Analysis of relative sequence read depth is consistent with high-frequency *GAP1* CNVs in glutamine-limited populations (S2 Text). Population sequencing also confirmed the fixation of a *GAP1* deletion (ure_05) in a urea-limited population. Relative sequence read depth at the *GAP1* locus correlates well with the normalized fluorescence of the *GAP1* CNV reporter in populations (S10 Fig), providing additional evidence for the utility of the CNV reporter. In glutamine-limited chemostats, *GAP1* copy number estimated within populations (which is a function of copy number within clones and allele frequencies) ranges from 2 to 4 copies, with a trend toward increased copy number over time (S10 Fig).

We performed single-nucleotide variant (SNV) analysis using genome sequencing data from populations (S9 Table) and clones (S10 Table) at generations 150 and 250. More nonsynonymous SNVs were identified in glucose-limited populations than the glutamine- and urea-limited populations (Table 2), which contained *GAP1* and *DUR3* amplifications at high frequencies at 150 and 250 generations. In contrast to previous studies [28,29], we did not identify CNVs at the *HXT6/7* locus in glucose-limited populations. Increased nucleotide variation within these populations may reflect alternative adaptive strategies in glucose-limited populations.

**Table 2:** Summary of single nucleotide variation in three different selection conditions. Populations were sequenced at 150 and 250 generations. For variants that were identified at both time points, we determined whether they increased (**↑**) or decreased (**↓**) in frequency between generation 150 and 250.

	Glucose (*n* = 10)				Urea (*n* = 11)				Glutamine (*n* = 11)			
	Total		Trend		Total		Trend		Total		Trend	
Predicted effect	g150	g250	↑	↓	g150	g250	↑	↓	g150	g250	↑	↓
Noncoding	4	9	2	1	8	12	1	0	6	5	0	0
Missense	47	61	17	4	22	34	6	2	12	22	4	1
Frameshift	4	5	1	1	5	6	2	0	2	2	0	0
Synonymous	1	7	0	0	4	10	2	0	4	3	0	0
Stop gained	4	10	2	0	7	4	0	2	0	5	0	0
Start lost	0	1	0	0	0	0	0	0	0	0	0	0
Splice variant	2	3	0	1	0	0	0	0	0	1	0	0
Mito. genome	0	0	0	0	0	2	0	0	0	0	0	0
In-frame insertion	0	0	0	0	0	0	0	0	0	1	0	0
Total variants	62	96	22	7	46	68	11	4	24	39	4	1

Abbreviation: Mito., mitochondrial.

We find several genes with multiple independent, nonsynonymous variation in glutamine-limited populations (Table 3), including *MCK1*, a protein kinase with potential roles in nonhomologous end joining (NHEJ); *SOG2*, a member of the regulation of Ace2p activity and cellular morphogenesis (RAM) signaling pathway and regulator of bud separation after mitosis; and *TAO3*, another member of the RAM network. We previously reported mutations in *MCK1* from selection in glutamine- and arginine-limited chemostats [24], suggesting that it is a recurrent target of selection in these conditions. Changes in cell morphology are potentially adaptive in nutrient-poor conditions, which may result from defects in cell cycle progression and bud separation associated with mutations in the RAM pathway [65]. However, the effect of these mutations on bud separation is likely to be minor, as we did not observe increases in forward scatter (which varies with cell size) in flow cytometry data, except in one glucose-limited population (S3 Fig).

**Table 3:** Genes with multiple, independent, nonsynonymous acquired mutations. Variants found at greater than 5% frequency within each population.

Glucose limitation		Urea limitation		Glutamine limitation	
Gene name	Total variants	Gene name	Total variants	Gene name	Total variants
*TRK1*	11	*DUR1*,*2*	14	*MCK1*	3
*SVF1*	2			*SOG2*	3
*CDC48*	3			*TAO3*	2
*WHI2*	3			*GPB2*	2

In the nine urea-limited populations, we identified 14 independent nonsynonymous variants in *DUR1*,*2* (Table 3). *DUR1*,*2* encodes urea amidolyase, which metabolizes urea to ammonium. At two different nucleotide positions, we find that the same nucleotide was mutated multiple times independently. In a third location, we identified an SNV at the exact nucleotide position as we previously reported [24]. Thus, a subset of variants in *DUR1*,*2* appear to be uniquely beneficial and recurrently selected in urea-limited environments.

In glucose-limited populations, we identified multiple, independent mutations in four genes (Table 3): *TRK1*, a component of the potassium transport system; *SVF1*, which is important for the diauxic growth shift and is implicated in cell survival during aneuploidy [66]; *CDC48*, an ATPase associated with diverse cellular activities (AAA); and *WHI2*, which is a mediator of the cellular stress response. Previous studies have identified loss-of-function mutations in *WHI2*, suggesting it is a general target of selection across different conditions [24,27,67].

Analysis of clonal samples (S10 Table) was largely consistent with population sequencing. We identified two cases in which SNVs occurred within *GAP1* CNVs. These SNVs are present at frequencies of 53% in a lineage containing a *GAP1* duplication and 30% in a lineage containing a *GAP1* triplication, indicating that they are present on only one of the copies within the CNV. We also identified polymorphisms within *DUR3* amplifications (S10 Table). This suggests that individual copies of a gene within a CNV can accumulate additional nucleotide variation even in relatively short-term evolutionary scenarios. Eight of the 9 clones with *DUR3* amplifications also acquired a variant in *DUR1*,*2*, which may be indicative of a synergistic relationship between CNVs and SNVs.

### Lineage tracking reveals extensive clonal interference among CNV lineages

The reproducible dynamics of CNV lineages observed during glutamine-limited experimental evolution may be due to two nonexclusive reasons: either (1) a high supply rate of de novo CNVs or (2) preexisting CNVs in the ancestral population (S11 Fig). In both scenarios, a single CNV or multiple, competing CNVs may underlie the reproducible dynamics. Sequence analysis of clonal lineages suggests at least two, and as many as four, CNV lineages may coexist in populations (Fig 3); however, genome sequencing is uninformative about the total number of lineages for two key reasons. First, the recurrent formation of CNVs confounds distinguishing CNVs that are identical by state from those that are identical by descent. Second, CNVs that arise de novo may subsequently diversify over time, resulting in distinct alleles that are derived from a common event.

**Fig 5:**
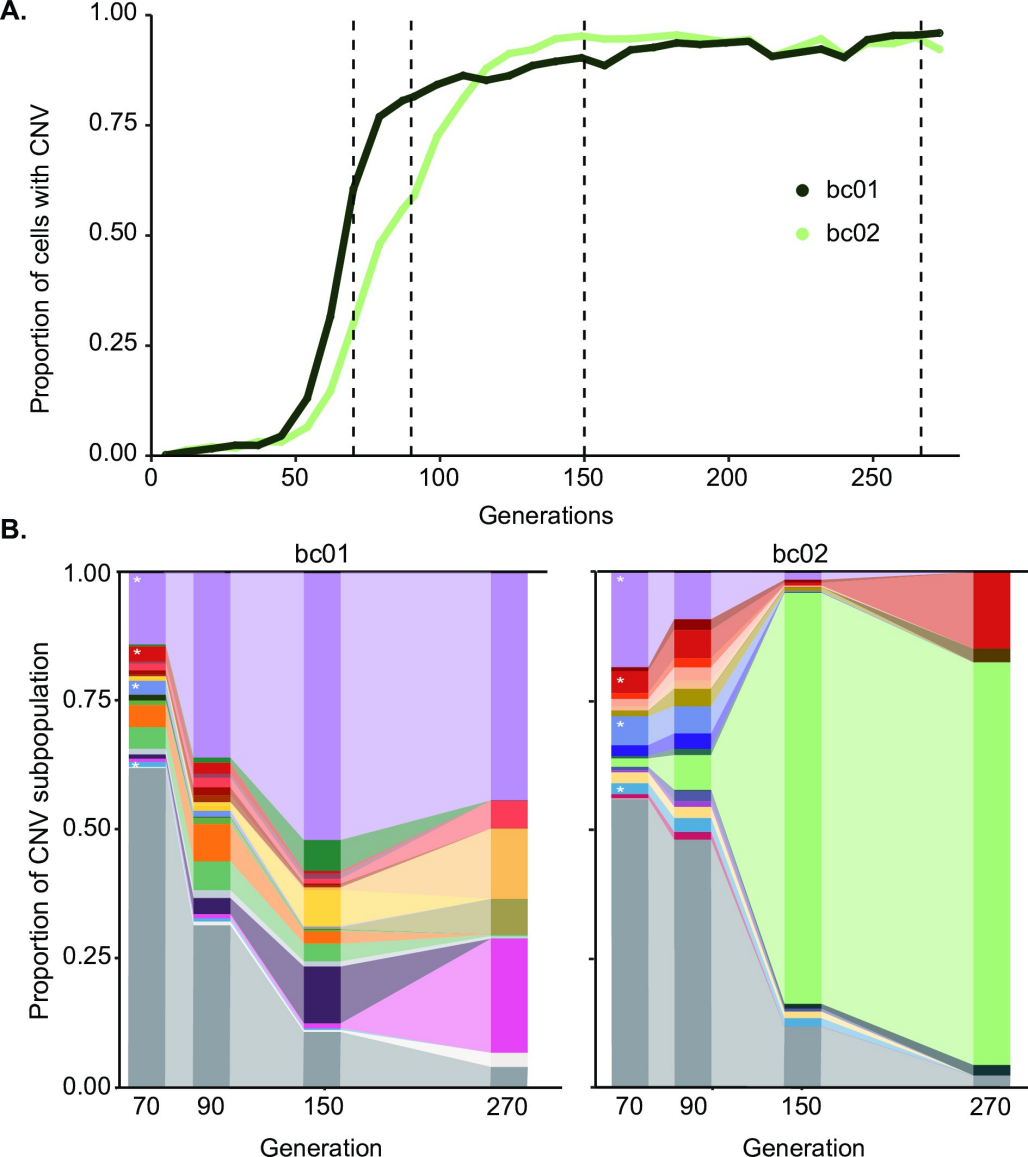
Lineage tracking reveals extensive clonal interference among CNV-containing lineages. (A) We used FACS to fractionate cells containing *GAP1* CNVs from two populations at four time points (dashed black lines) and performed barcode sequencing. (B) Using a sample- and time point–specific false positive correction, we identified 7,067, 973, 131, and 76 barcodes in one population (bc01; left) and 5,305, 5,351, 583, and 28 barcodes in another population (bc02; right), at generations 70, 90, 150, and 270, respectively. Each barcode found at >1% frequency in at least one time point is represented by a unique color in the plot, for a total of 21 barcodes in bc01 and 18 barcodes in bc02. All other lineages that are never detected at >1% frequency are shown in gray. Lineages denoted by a * are found at >1% frequency in both populations. Data and computer code used to generate this figure can be accessed in OSF: https://osf.io/fxhze/. CNV, copy number variant; FACS, fluorescence-activated cell sorting.

To quantify the number, relationship, and dynamics of individual CNV lineages, we constructed a lineage-tracking library using random DNA barcodes [68]. We constructed a library of approximately 80,000 unique barcodes (S12 Fig) in the background of the *GAP1* CNV reporter and performed six independent replicate experiments in glutamine-limited chemostats. Real-time monitoring of CNV dynamics using the *GAP1* CNV reporter recapitulated the dynamics of our original experiment (Fig 5A, S13A Fig, and S11 Table), although CNV lineages appeared significantly earlier in these populations (T_up_; *t* test *p*-value < 0.01). As the lineage-tracking strain was independently derived from the strain used in our original experiment, these results indicate that selection of *GAP1* CNVs in glutamine-limited chemostats is reproducible and independent of genetic background.

To quantify individual lineages, we isolated the subpopulation containing CNVs from two populations (bc01 and bc02) at multiple time points (generations 70, 90, 150, and 270). Isolation of the CNV subpopulation was performed by FACS using gates based on one- and two-copy control populations (Fig 5A, S14 Fig). We sequenced barcodes from the CNV subpopulation at each time point and determined the number of unique lineages ([69] and Methods). To account for variation in the purity of the FACS-isolated CNV subpopulation, we analyzed individual clones using a flow cytometer. Using these data, we estimated a false positive rate, which we find varies between time points (S13B Fig and Methods), and applied this correction to barcode counts (Table 4).

**Table 4:** Estimation of CNV lineages in evolving populations across time. We determined the number of *GAP1* CNV-containing lineages by correcting the number of identified barcodes by the estimated false positive rate associated with CNV isolation using FACS. High-confidence *GAP1* CNV lineages are defined as those that are found at two or more consecutive time points. Data and computer code used to generate this table can be accessed in OSF: https://osf.io/fxhze/.

Population	Generation	Number of detected barcodes	FP	FP-corrected barcode count	Barcodes identified at >1 time point
bc01	70	9,650	0.27	7,067	891
bc01	90	1,064	0.09	973	891
bc01	150	136	0.04	131	131
bc01	270	79	0.04	76	38
bc02	70	7,243	0.27	5,305	2,676
bc02	90	5,851	0.09	5,351	2,710
bc02	150	606	0.04	583	162
bc02	270	29	0.04	28	22

Abbreviations: CNV, copy number variant; FACS, fluorescence-activated cell sorting; FP, false positive rate.

We detect thousands of independent *GAP1* CNV lineages at generation 70, indicating that a large number of independent *GAP1* CNVs are generated and selected in the early stages of the evolution experiments (Fig 5B). Applying a conservative false positive correction, we identified 7,067 *GAP1* CNV lineages in bc01 and 5,305 *GAP1* CNV lineages in bc02 at generation 70 (Table 4). If we only consider lineages detected in the CNV subpopulation at multiple time points, we identify 891 CNV lineages in bc01 and 2,676 CNV lineages at generation 70 (Table 4). Thus, between 10^2^ and 10^4^ independent CNV lineages in each population of 10^8^ cells initially compete with each other. The overall diversity of CNV lineages decreases with time, consistent with decreases in lineage diversity observed in other evolution experiments [68,70]. By generation 270, we detect only 76 CNV lineages in bc01 and 28 CNV lineages in bc02. To determine the dominant lineages in each population, we identified barcodes that reached greater than 1% frequency in the CNV subpopulation in at least one time point: 21 independent lineages are found at greater than 1% frequency in bc01, and 18 independent lineages are found at greater than 1% frequency in bc02 (Fig 5B). These results indicate the presence and persistence of multiple *GAP1* CNVs across hundreds of generation of selection, during which there is a continuous reduction in the overall diversity of CNV lineages.

Although CNVs rise to high frequencies in both populations (Fig 5A), the composition of competing CNV lineages is dramatically different: in bc02, a single lineage dominates the population by generation 150 (Fig 5B), whereas in bc01, there is much greater diversity at later time points. In both populations, several CNV lineages that comprise a large fraction of the CNV subpopulation at early generations (generations 70, 90, or 150) are extinct by generation 270. Thus, within populations, individual CNV lineages do not increase in frequency with uniform dynamics, despite the consistent and reproducible dynamics of the entire CNV subpopulations (Fig 5A and Fig 2). Differences in fitness between individual CNV lineages, possibly as a result of variation in copy number, CNV size, and secondary adaptive mutations, are likely to contribute to these dynamics.

### CNV subpopulations comprise de novo and preexisting CNV alleles

To distinguish the contribution of preexisting genetic variation (i.e., CNVs introduced to the population before chemostat inoculation; S11 Fig) and de novo variation (i.e., CNVs introduced to the population following chemostat inoculation) to CNV lineage dynamics, we assessed whether barcodes were shared between CNV lineages in independent populations. We identified four barcodes at greater than 1% frequency that are common to both populations (Fig 5B). At generation 70, one of these barcodes (indicated in light purple) was present at 14% and 19% in bc01 and bc02, respectively. We find that the barcode for this lineage was overrepresented in the ancestral unselected population (an initial frequency of 0.014%, which is one order of magnitude greater than the average starting frequency of 0.0011%; S12 Fig). Although there is a possibility that de novo CNVs formed independently in this barcode lineage, it is more likely that this lineage contained a preexisting CNV in the ancestral population. Although this lineage represented a sizable fraction of the CNV subpopulation in both replicate populations, it was only maintained at high frequency in one of them (bc01). Only one of the four preexisting CNV lineages persists throughout the experiment in both populations. By contrast, in each population, we identified 17 and 14 unique high-frequency CNV lineages that are most likely new CNVs. These results indicate that both preexisting CNVs and de novo CNVs that arise during glutamine limitation contribute to adaptive evolution.

## Discussion

CNVs are an important class of genetic variation and adaptive potential. In this study, we sought to understand the short-term fate of CNVs as they are generated and selected in evolving populations. Previous work from our laboratory and others has shown that the defined, strong selective conditions of a chemostat provides an ideal system for studying CNVs. We used nitrogen limitation to establish conditions that select for amplification and deletion of the gene *GAP1*, which encodes the general amino acid permease, in *S*. *cerevisiae*.

### A *GAP1* CNV reporter reveals the dynamics of selection

To determine the dynamics with which CNVs are selected at the *GAP1* locus, we inserted a constitutively expressed fluorescent gene adjacent to *GAP1* and tracked changes in single-cell fluorescence over time. Whereas one- and two-copy control strains with *mCitrine* at neutral loci maintain a steady fluorescent signal over 250 generations of selection, all glutamine-limited populations with the *GAP1* CNV reporter show increased fluorescence by generation 75. The structure and breakpoints of CNVs within and between populations are different, indicating independent formation of CNVs. Control strains were inoculated independently and have different genetic backgrounds but also form CNVs at the *GAP1* locus, as determined by whole-genome sequencing and Southern blot analysis. These data indicate that *GAP1* CNVs are positively selected early and repeatedly in glutamine-limited environments.

Although the majority of evolved clones with *GAP1* CNVs (18/28) have higher relative fitness in glutamine-limited chemostats compared to the ancestor, several clones have neutral (8/28) or lower (2/28) relative fitness. CNV-containing clones were selected on the basis of increased fluorescence, which does not necessarily mean the clone had higher fitness than the ancestor. The fitness effect of a CNV within the chemostat environment is context specific and may depend on factors such as frequency-dependent selection. In addition, if *GAP1* CNVs are generated at a high rate, as we have hypothesized, neutral or deleterious CNVs could be present for several generations before these lineages are purged from the population or acquire additional adaptive mutations.

### Inferences of CNV formation mechanisms

Whole-genome sequencing of *GAP1* CNV lineages isolated on the basis of increased fluorescence uncovered a wide range of CNV structures within and between populations. We found cases in which distinct alleles were identified within populations at different time points and cases in which we identified the same CNV allele 100 generations later. *GAP1* CNV alleles are 105 kilobases on average but can include the entire right arm of Chromosome XI (260 kilobases). A previous study in bacteria showed that there is a cost to gene duplication, with a fitness reduction of 0.15% per kilobase [71]. Therefore, we hypothesized that CNVs would decrease in size over evolutionary time through a refinement process in order to reduce the fitness burden. However, we failed to detect a significant reduction in CNV allele size over time. This may be because increased CNV size does not confer a fitness cost in yeast, the fitness benefit of the *GAP1* CNV outweighs this cost, or there are other genes within the CNV whose amplification confers a fitness benefit.

Our reporter detects increases in gene copy number that result from a variety of processes such as aneuploidy, nonreciprocal translocation, tandem duplication, and complex CNVs, including inverted triplications. The ability to track and isolate these diverse gene amplifications allows us to enumerate the frequency of each type and characterize the mechanisms underlying their formation. Combining our approach with molecular techniques allowed us to further understand the nature of these *GAP1* CNVs. Three particularly interesting *GAP1* CNV-containing clones appear to have partial (i.e., segmental) aneuploidies that encompass centromere 11 (S5 Fig). As the presence of two centromeres in one chromosome is extremely unlikely, it is plausible that these exist as independent, supernumerary chromosomes [72]. Similar adaptive rearrangements occur in other yeast species: isochromosome formation, potentially mediated by the presence of inverted repeats, has been observed during treatment of *Candida albicans* with antifungal drugs [73]. The use of a CNV reporter should facilitate determination of the frequency with which these and other complex mechanisms give rise to CNVs at a given locus.

Breakpoint analysis provided further insight into the mechanisms underlying CNV formation. We identified breakpoints within LTRs and other repetitive elements for 4 unique glutamine-limited clones that have 2 copies of *GAP1*. These findings suggest that these CNVs were formed by a tandem duplication mediated through NAHR. Of these, 3 *GAP1* gene amplifications (3/28) are formed after NAHR between flanking LTRs *YKRCdelta11* and *YKRCdelta12*. The *GAP1* deletion, which occurred in one population undergoing urea limitation, also had breakpoints in these flanking elements consistent with NAHR-mediated gene deletion. NAHR may drive the nonreciprocal translocations we identified and additional unresolved events with breakpoints adjacent to LTRs. We did not find evidence for the selection of *GAP1*^*circle*^ CNVs in any population. Thus, it may be that circular elements containing beneficial genes only exist transiently in cells and may rapidly resolve to chromosomal amplifications via homologous recombination–mediated reintegration.

We identified 9 *GAP1* CNVs and 8 *DUR3* CNVs that contain breakpoints characterized by closely spaced inverted repeat sequences. Of these, the majority (14/17) also had an odd copy number and contained an origin of replication consistent with the ODIRA mechanism [47,48]. However, we also identified one *DUR3* CNV that does not include a replication origin (ure_07_c1), although the origin is nearby (<1 kilobase). This could result from a distinct replication-based mechanism of CNV generation. For example, MMBIR is a RAD51-independent process that relies on short stretches of homology (“microhomology”) to restart a stalled replication fork [45]. Though we cannot explicitly distinguish between these models, the short stretches of homology in the inverted repeats is inconsistent with formation of this CNV by NAHR. Thus, while NAHR plays an important role in CNV formation, our results suggest that replication-based mechanisms may be a major source of gene amplification in yeast. This is consistent with increasing evidence for replication-based CNV formation in diverse organisms including yeast, mice, and humans [74–77].

Comparison between *DUR3* and *GAP1* CNVs identified quantitative differences in CNV formation at the two loci. We primarily identified CNVs with 2 or 3 copies of *GAP1* in glutamine-limited clones, but urea-limited clones always contained 5 copies of *DUR3*. The size (average of 26 kilobases) of *DUR3* CNVs was also significantly smaller than *GAP1* CNVs. Molecular characterization revealed a diverse range of processes underlying *GAP1* CNV formation, whereas *DUR3* CNVs are all characterized by inversions mediated by short, interrupted, inverted repeats. These data suggest that generation and selection of CNVs vary as a function of locus and selective condition. The CNV reporter can readily be integrated throughout the genome to further test whether there are fundamental differences in CNV formation mechanisms at different loci and how these differences change the temporal dynamics of CNV selection.

### Clonal interference underlies CNV dynamics

By combining a CNV reporter with lineage tracking, we identified a surprisingly large number of independent CNV lineages. Whereas clonal isolation and sequencing suggested at least four independent lineages within populations, lineage tracking indicates that hundreds to thousands of individual CNV lineages emerge within fewer than 100 generations. Most of these lineages do not achieve high frequency, as we identified only 18–21 lineages present at >1% frequency in the CNV subpopulation. The number of independent CNV lineages we identified is remarkable. Although we have attempted to account for technical factors that may inflate this number, unanticipated aspects of barcode transformation and library construction, cell sorting, and barcode sequencing and identification may impact this estimation. Conversely, the exact number of CNV lineages may be underestimated, as the unselected barcode library was not maximally diverse and each unique barcode was shared by multiple founding cells.

Although we found lineages that were common to both populations (at least one of which is likely to contain a preexisting CNV), ancestral CNV lineages do not drive the evolutionary dynamics. Preexisting CNV lineages have different dynamics in each population and do not prevent the emergence of unique de novo CNV lineages. This demonstrates that the ultimate fate of a CNV lineage depends on multiple factors, and a high frequency at an early generation does not guarantee that a lineage will persist in the population. Thus, CNV dynamics result from preexisting and de novo variation and are characterized by extensive clonal interference and replacement among competing CNV lineages.

The large number of CNV lineages identified in our study indicates that they occur at a high rate. Recent studies have suggested that adaptive mutations may be stimulated by the environment. Stress can lead to increases in genome-wide mutation rates in both bacteria and yeast [78–80], and replicative stress can lead directly to increased formation of CNVs [81,82]. Other groups have proposed an interplay between transcription and CNV generation and that active transcription units might even be “hotspots” of CNV formation [83–85]. These hotspots, often designated as common fragile sites, may occur in long, late-replicating genes, with large interorigin distances [82]. Local transcription at the rDNA locus leads to rDNA amplification and is thought to be regulated in response to the environment [86,87]. Transcription of the *CUP1* locus in response to environmental copper leads to promoter activity that further destabilizes stalled replication forks and generates CNVs [88]. Given the high level of *GAP1* transcription in nitrogen-limited chemostats [58], it is tempting to speculate that this condition may promote the formation of *GAP1* CNVs. Further studies are required to understand the full extent of processes that underlie CNV formation at the *GAP1* locus and how these different mechanisms may contribute to the fitness and overall success of CNV lineages.

The frequency of *GAP1* CNVs can be attributed to a combination of factors, including a high mutation supply rate due in part to the large chemostat population size (approximately 10^8^ cells), the strength of selection, and the fitness benefit typically conferred by *GAP1* amplification. Together, these factors contribute to an early, deterministic phase, during which CNVs are formed at a high rate and thousands of lineages with CNVs rapidly increase in frequency. During a second phase, the dynamics are more variable, as competition from different types of adaptive lineages and additional acquired variation influence evolutionary trajectories of individual CNV lineages. This phenomenon has recently been observed in other evolution experiments, in which early events are driven by multiple competing single-mutant lineages [70], but later dynamics are influenced by stochastic factors and secondary mutations [68].

The high degree of clonal interference observed among a single class of adaptive mutations may have important implications for adaptive evolution. CNVs are alleles of large effect that can simultaneously change the dosage of multiple protein-coding genes and subsequently lead to changes in cell physiology. Epistatic relationships between CNVs and other adaptive mutations could therefore dramatically alter the fitness landscape [31]. Additionally, CNVs can confer a fitness benefit per se but also serve to increase the amount of DNA in the genome that can accumulate mutations. Therefore, CNVs can potentially increase the rate of adaptive evolution by increasing the target size for adaptive mutations. In this study, we found evidence for polymorphisms within individual CNVs and potential epistasis between SNVs and CNV alleles, two phenomena that require further exploration as we continue to define the role of CNVs in driving rapid adaptive evolution.

### Conclusion

The combined use of a fluorescent CNV reporter and barcode lineage tracking provides unprecedented insight into this important class of mutation. Previous studies have tracked specific mutations and their fitness effects [60], but ours is the first single cell–based approach to identify an entire class of mutations and follow evolutionary trajectories with high resolution. Whereas barcode tracking alone provides information about the number of adaptive lineages and their fitness effects, the CNV reporter enables us to specifically determine the number of unique CNV events. In addition, the reporter provides an estimate of the total proportion of CNVs in the population, which we can use to inform our understanding of lineage dynamics. Using these tools, we have shown that CNVs are generated at a high rate through diverse mechanisms including homologous recombination and replication-based errors. These processes lead to the formation of many distinct CNV alleles segregating within populations. One limitation of our approach is that a complex CNV could be the product of multiple, independent events (e.g., a duplication followed by a subsequent triplication). Evolution experiments that start with a preexisting CNV would be informative for studying how CNVs diversify when maintained under selection.

Our results demonstrate an important role for CNVs in driving rapid adaptive evolution in microbial populations but could be broadly applicable to plants, animals, and humans. Our system provides a facile means for studying the molecular processes underlying CNV generation as well as evolutionary aspects of CNVs, including whether there are fundamental differences in CNV formation and selection at different loci, the impact of a high rate of CNV formation on the evolutionary dynamics of other adaptive lineages, how CNVs are maintained or refined over longer evolutionary timescales, how CNVs interact with other adaptive mutations to influence fitness landscapes, whether there are consequences and tradeoffs in alternative environments, and how the formation of CNVs impacts gene expression and genome architecture. Extension of this method is likely to be useful for addressing additional fundamental questions regarding the evolutionary and pathogenic role of CNVs in diverse systems.

## Methods

### Strains and media

We used FY4 and FY4/5, haploid and diploid derivatives of the reference strain S288c, for all experiments. S1 Table is a comprehensive list of strains constructed and used in this study. To generate fluorescent strains, we performed high-efficiency yeast transformation [89] with an *mCitrine* gene under control of the constitutively expressed *ACT1* promoter (*ACT1pr*::*mCitrine*::*ADH1term*) and marked by the KanMX G418-resistance cassette (*TEFpr*::*KanMX*::*TEFterm*). The entire construct, which we refer to as the mCitrine CNV reporter, is 3,375 base pairs. For control strains, the mCitrine reporter was integrated at two neutral loci: *HO* (*YDL227C*) on Chromosome IV and the dubious ORF, *YLR123C*, on Chromosome XII. Diploid control strains containing 3 and 4 copies of the mCitrine CNV reporter were generated using a combination of backcrossing and mating. We constructed the *GAP1* CNV reporter by integrating the mCitrine construct at an intergenic region 1,118 base pairs upstream of *GAP1* (integration coordinates, Chromosome XI: 513945–517320). PCR and Sanger sequencing were used to confirm integration of the *GAP1* CNV reporter at each location (all PCR primer sequences are provided in S12 Table). Transformants were subsequently backcrossed and sporulated, and the resulting segregants were genotyped.

For the purpose of lineage tracking, we constructed a strain containing a landing pad and the *GAP1* CNV reporter by segregation analysis after mating the original *GAP1* CNV reporter strain to a landing pad strain (derived from BY4709) [68]. As the kanMX cassette is present at two loci in this cross, we performed tetrad dissection and identified four spore tetrads that exhibited 2:2 G418 resistance. A segregant with the correct genotype (G418 resistant, ura-) was identified and confirmed using a combination of PCR (S12 Table) and fluorescence analysis. We introduced a library of random barcodes by transformation and selection on SC-ura plates [68]. We plated an average of 500 transformants on 200 petri plates and estimated 78,000 independent transformants.

Nitrogen-limiting media (glutamine and urea limitations) contained 800 μM nitrogen regardless of molecular form and 1 g/L CaCl_2_-2H_2_O, 1 g/L of NaCl, 5 g/L of MgSO_4_-7H_2_O, 10 g/L KH_2_PO_4_, 2% glucose and trace metals and vitamins as previously described [24]. Glucose-limiting media contained 0.08% glucose, 1 g/L CaCl_2_-2H_2_O, 1 g/L of NaCl, 5 g/L of MgSO_4_-7H_2_O, 10 g/L KH_2_PO_4_, 50 g/L (NH_4_)_2_SO_4_ and trace metals and vitamins [90].

### Long-term experimental evolution

We inoculated the *GAP1* CNV reporter strain into 20-mL ministat vessels [91] containing either glutamine-, urea-, or glucose-limited media. Control populations containing either one or two copies of the CNV reporter at neutral loci (*HO* and *YLR123C*) were also inoculated in ministat vessels for each media condition. Ministats were maintained at 30°C in aerobic conditions and diluted at a rate of 0.12 hour^−1^ (corresponding to a population doubling time of 5.8 hours). Steady-state populations of 3 × 10^8^ cells were maintained in continuous mode for 270 generations (65 days). Every 30 generations, we archived 2-mL population samples at −80°C in 15% glycerol.

### Flow cytometry sampling and analysis

To monitor the dynamics of CNVs, we sampled 1 mL from each population about every 8 generations. We performed sonication to disrupt any cellular aggregates and immediately analyzed the samples on an Accuri flow cytometer, measuring 100,000 cells per population for mCitrine fluorescence signal (excitation = 516 nm, emission = 529 nm, filter = 514/20 nm), cell size (forward scatter), and cell complexity (side scatter). We generated a modified version of our laboratory flow cytometry pipeline for this analysis (https://github.com/GreshamLab/flow), which uses the R package *flowCore* [92]. We used forward scatter height (FSC-H) and forward scatter area (FSC-A) to filter out doublets and FSC-A and side scatter area (SSC-A) to filter debris. We quantified fluorescence for each cell and divided this value by the forward scatter measurement for the cell to account for differences in cell size. To determine population frequencies of cells with zero, one, two, and three or more copies of *GAP1*, we used one- and two-copy control strains grown in glutamine-limited chemostats to define gates and perform manual gating. We used a conservative gating approach to reduce the number of false positive CNV calls by manually drawing first a liberal gate for the one-copy control strain and then a nonoverlapping gate for the two-copy control strain. Flow cytometry data and code used to generate all figures and tables can be accessed in OSF: https://osf.io/fxhze/.

### Quantification of CNV dynamics

To quantify the dynamics of CNVs in evolving populations, we defined summary statistics as in [60]. T_up_ is the generation at which CNVs are initially detected, and S_up_ is the slope of the linear fit during initial population expansion of CNVs. We first determined the proportion of cells with a CNV and the proportion of cells without CNVs at each time point, using the manually defined gates. To calculate T_up_, we defined a false positive rate for CNV detection in evolving one-copy control strains from generations 1–153 (defined as the average plus one standard deviation = 7.1%). We designate T_up_ once an experimental population surpasses this threshold. To calculate S_up_, we plotted the natural log of the ratio of the proportion of cells with and without a CNV against time and calculated the linear fit during initial population expansion of CNVs. We defined the linear phase on the basis of R^2^ values (S1 Text). S_up_ can also be defined as the percent increase in CNVs per generation, which is an approximation for the relative average fitness of all CNV alleles in the population.

### Isolation and analysis of evolved clones

Clonal isolates were obtained from each glutamine- and urea-limited population at generation 150 and generation 250. We isolated clones by plating cells onto rich media (YPD) and randomly selecting individual colonies. We inoculated each clone into 96-well plates containing the limited media used for evolution experiments and analyzed them on an Accuri flow cytometer following 24 hours of growth. We compared fluorescence to unevolved ancestral strains, evolved 1- and 2-copy controls grown under the same conditions, and chose a subset of clones for whole-genome sequencing (S4 Table).

To measure the fitness coefficient of evolved clones, we performed pairwise competitive fitness assays in glutamine-limited chemostats using the same glutamine-limited conditions as our evolution experiments [24]. We cocultured our fluorescent evolved strains with a nonfluorescent, unevolved reference strain (FY4). We determined the relative abundance of each strain every 2–3 generations for approximately 15 generations using flow cytometry. We performed linear analysis of the natural log of the ratio of the two genotypes against time and estimated the fitness and associated error relative to the ancestral strain.

### Plug preparation, pulsed-field gel electrophoresis, and Southern blotting

Evolved clones were grown overnight in glutamine-limited media and embedded in agarose using Bio-rad plug molds. Plugs were incubated in zymolyase T100 (200 μg/mL) overnight at 37°C, proteinase K (4 mg/mL) overnight at 50°C, and PMSF (1 mM) for 1 hour at 4°C. PMSF was removed by washing plugs with 1 mL of CHEF TE 3 times for 30 minutes. Plugs were subsequently run in a 1X TAE, 1% agarose gel using a Bio-rad CHEF-DR II. Southern blotting was performed by alkaline transfer using Hybond-XL membranes. Blots were subsequently probed with ^32^P-labeled DNA complementary to *GAP1* or *CEN11*. Probes were created using nested PCR with primers listed in S12 Table. Signal from blots was detected using FujiFilm imaging plates and imaged using Typhoon FLA9000.

### Genome sequencing

For both population and clonal samples, we performed genomic DNA extraction using a modified Hoffman-Winston protocol [93]. We used SYBR Green I to measure gDNA concentration, standardized each sample to 2.5 ng/μL, and constructed libraries using tagmentation following a modified Illumina Nextera library preparation protocol [94]. To perform PCR clean-up and size selection, we used an Agilent Bravo liquid-handling robot. We measured the concentration of purified libraries using SYBR Green I and pooled libraries by balancing their concentrations. We measured fragment size with an Agilent TapeStation 2200 and performed qPCR to determine the final library concentration.

DNA libraries were sequenced using a paired-end (2 × 75) protocol on an Illumina NextSeq 500. Standard metrics were used to assess data quality (Q30 and %PF). To remove reads from a potentially contaminating organism that was introduced after recovery from the chemostats, we filtered any reads that aligned to *Pichia kudriavzevii*. Given the evolutionary divergence between these species, the majority of filtered reads belonged to rDNA and similar, deeply conserved sequences. The median percent contamination was 1.165%. We modified the *S*. *cerevisiae* reference genome from NCBI (assembly R64) to include the entire *GAP1* CNV reporter and aligned all reads to this reference. We aligned reads using bwa mem ([95], version 0.7.15) and generated BAM files using samtools ([96], version 1.3.1). Summary statistics for all sequenced samples are provided in S3 Table. FASTQ files for all sequencing are available from the SRA (accession SRP142330). Sequencing data and code used to generate all figures and tables can be accessed in OSF: https://osf.io/fxhze/.

### CNV detection using published algorithms

To assess the performance of CNV detection algorithms, we simulated CNVs ranging in size from 50 to 100,000 base pairs in 100 synthetic yeast genomes. We used SURVIVOR [97] to simulate CNVs in the reference yeast genome and wgsim [96] to generate corresponding paired-end FASTQ files. We used bwa mem [95] to map reads back to the reference and called CNVs with Pindel, CNVnator, LUMPY, and SvABA [61–64]. We assessed the effect of read depth on algorithm performance by downsampling a 100× coverage BAM file to 80×, 50×, 20×, 10×, and 5× coverage. We defined a CNV as being correctly predicted if the simulated and detected CNVs were (1) of the same type (e.g., duplication), (2) predicted to be on the same chromosome, and (3) contained in the same interval (defined by the start and stop position), which were considered overlapping if there was no gap between them (maxgap = 0) and had minimum overlap of 1 base pair (minoverlap = 1). For intervals [a,b] and [c,d], for which a ≤ b and c ≤ d, when c ≤ b and d ≥ a the two intervals overlap, and when c > b or d < a the two intervals do not overlap. If the gap between these two intervals is ≤maxgap and the length of overlap between these two intervals is ≥minoverlap, the two intervals are considered to be overlapping.

To assess the performance of these tools on heterogeneous population samples, we also simulated mixed samples by combining reads from a simulated CNV-containing genome and an unmodified reference yeast genome at varying proportions. The ratio of the reads from the CNV-containing genome varied between 20% and 90%, and the total coverage was 50×.

Performance comparisons for all benchmarking were based on false discovery rate (FDR) and F-score. The F-score (also known as F1 measure) combines sensitivity/recall(r) and precision(p) with an equal weight using the formula F = (2pr) / (p + r) [98]. An F-score reaches its best value at 1 and worst at 0 and was multiplied by 100 to convert to a percentage value. We called CNVs for each clone and population sample using an in-house pipeline that collates results from Pindel, SvABA, and LUMPY (S5 Table and S6 Table). Data and code used to generate these figures can be accessed in OSF: https://osf.io/fxhze/.

### Sequence read depth and breakpoint analysis

To manually estimate CNVs boundaries, we used a read depth–based approach. For each sample sequenced, we used samtools [96] to determine the read depth for each nucleotide in the genome. We liberally defined CNVs by identifying ≥300 base pairs of contiguous sequence when read depth was ≥3 times the standard deviation across Chromosome XI for *GAP1* or Chromosome VIII for *DUR3*. These boundaries were further refined by visual inspection of contiguous sequence ≥100 base pairs with read depth ≥3 times the standard deviation. These analyses were only performed on sequenced clones because population samples are likely to have multiple CNVs and breakpoints, thereby confounding read depth–based approaches. We compared manually estimated breakpoints to those identified by the algorithms (S5 Table) and defined a set of “high-confidence breakpoints.”

To determine CNV breakpoints at nucleotide resolution, we extracted split and discordant reads from bam files using samblaster [99]. Both split reads and discordant reads were used to identify breakpoints using a weighted scoring method wherein a split read was worth 1 and discordant reads were worth 3. Positively identified breakpoints required at least 4 split reads and a combined score of at least 9. Breakpoint sequences were generated by making local assemblies of breakpoint-associated split reads using MAFFT, EMBOSS, and velvet [100–102]. The relationship between breakpoint sequences and the reference genome was determined using BLAST+ [103], with blastn and blastn-short using default settings.

To infer the underlying mechanism by which CNVs were formed, we applied the following criteria. If at least one of the two CNV boundaries contained inverted repeat sequences, and we estimated an odd number of copies in the CNV, we classified the mechanism as ODIRA [26,47,48]. If both of the CNV boundaries occurred within repetitive sequence elements (LTRs or telomeres) and had two copies, we inferred tandem duplication by NAHR [40]. Aneuploids were defined on the basis of increased read depth throughout the entire chromosome but no detected novel sequence junctions. Translocations were identified by LUMPY and Southern blot analysis. All breakpoints that failed to meet these criteria were defined as unresolved.

In addition to CNVs at *GAP1* and *DUR3*, we also identified additional structural variants (S7 Table) and CNVs (S8 Table). Structural variants were identified using the split and discordant read approach described above. Additional CNVs were identified using a two-pass genome-wide read-depth approach. In the initial pass, each sample was scanned for regions (400 nucleotide minimum size) with read depth higher than 3 standard deviations relative to the genome. During the second pass, the read depth of each candidate is normalized by the median read depth of that region, as calculated using a subset of clones that lack a candidate in that region. This normalization allows for the correction of sequencing artifacts, batch effects, and the removal of CNV regions that are not substantially different between the evolved and ancestral clones (i.e., rDNA, Ty elements, etc.)

### SNV and variant identification

SNVs and indel variants were first identified using GATK4’s Mutect2 [104], which allows for the identification of variants in evolved samples (“Tumor”) after filtering using matched unevolved samples (“Normal”) and pool of normals (PON). The PON was constructed using 6 sequenced ancestral clones, whereas the paired normal was a single, deeply sequenced ancestor. Variants were further filtered using GATK’s FilterMutectCalls to remove low-quality predictions; only variants flagged as “passed” or “germline risk” were retained. Given the haploid nature of the evolved population and further downstream filtering of “too-recurrent” mutations, we allowed germline risk variants to be retained. Variants were further filtered if they occurred in low-complexity sequence; i.e., variants were filtered if the SNV or indel occurred in or generated a homogenous nucleotide stretch of five or more of the same nucleotide. Variants from within populations that were detected at less than 5% frequency were considered low confidence and excluded. Finally, variants were filtered if they were found to be “too recurrent”; i.e., if the exact nucleotide variant was identified in more than three independently evolved lineages, we deemed it more parsimonious to assume that the variant was present in the ancestor at low frequency.

### Quantifying the number of CNV lineages

We inoculated the lineage-tracking library into 20-mL ministat vessels [91] containing glutamine-limited media. Control populations containing either zero, one or two copies of the *GAP1* CNV reporter at neutral loci (*HO* and *YLR123C*) were also inoculated in ministat vessels for each media condition. Control populations did not contain lineage-tracking barcodes. Ministat vessels were maintained and archived as above. Samples were taken for flow cytometry about every 8 generations and analyzed as previously described.

We used FACS to isolate the subpopulation of cells containing two or more copies of the mCitrine CNV reporter using a FACSAria. We defined our gates using zero-, one-, and two-copy mCitrine control strains sampled from ministat vessels at the corresponding time points: 70, 90, 150, and 265 generations. Depending on the sample, we isolated 500,000–1,000,000 cells with increased fluorescence, corresponding to 2 or more copies of the reporter. We grew the isolated subpopulation containing CNVs for 48 hours in glutamine-limited media and performed genomic DNA extraction using a modified Hoffman-Winston protocol [93]. We verified FACS isolation of true CNVs by isolating clones from subpopulations sorted at generation 70, 90, and 150 (sorted from all lineage-tracking populations, bc01–bc06) and performing independent flow cytometry analysis using an Accuri. We estimated the average false positive rate of CNV isolation at each time point as the percent of clones from a population with FL1 less than one standard deviation above the median FL1 in the one copy control strain. Only subpopulations with fluorescence measurements for at least 25 clones were included in calculations of false positive rate.

We performed a sequential PCR protocol to amplify DNA barcodes and purified the products using a Nucleospin PCR clean-up kit [68]. We quantified DNA concentrations by qPCR before balancing and pooling libraries. DNA libraries were sequenced using a paired-end (2 × 150) protocol on an Illumina MiSeq 300 Cycle v2. Standard metrics were used to assess data quality (Q30 and %PF, S3 Table). However, the reverse read failed because of overclustering, so all analyses were performed only using the forward read. We used the Bartender algorithm with UMI handling to account for PCR duplicates and to cluster sequences with merging decisions based solely on distance except in cases of low coverage (<500 reads/barcode), for which the default cluster merging threshold was used [69]. Clusters with a size less than 4 or with high entropy (>0.75 quality score) were discarded. We estimated relative abundance of barcodes using the number of unique reads supporting a cluster compared to total library size. Data and code used to generate these figures and tables can be accessed in OSF: https://osf.io/fxhze/.

## Supporting information

S1 Text Calculation of CNV dynamics parameters. Graphic representation of linear fit (and corresponding R^2^ values) during initial population expansion of CNV alleles. Slope of the linear fit corresponds to the dynamics parameter S_up_ shown in Table 1 and was calculated for the original evolution experiment and the barcode experiment. Data and code used to generate these figures can be accessed in OSF: https://osf.io/fxhze/. CNV, copy number variant.
(PDF)

S2 Text Analysis of *GAP1* and *DUR3* CNVs. Relative read-depth plots for each population and corresponding clones isolated from these populations at generation 150 and 250. For a subset of clones with *GAP1* and *DUR3* CNVs, breakpoint maps are shown. Breakpoint maps were generated using local assembly of split reads and alignment to the reference genome. Code used to generate these figures can be accessed in OSF: https://osf.io/fxhze/. CNV, copy number variant.
(PDF)

S3 Text Performance of existing CNV detection algorithms. Application of existing CNV detection algorithms for analysis of genome sequencing data. CNV, copy number variant.
(DOCX)

S1 Fig Assessment of CNV reporter fitness effects. The fitness of strains carrying one (DGY500) or two copies (DGY1315) of a constitutively expressed mCitrine gene was assayed. Fluorescent strains were cocultured with the nonfluorescent, unevolved reference strain (FY4). We performed three independent competitive fitness assays in glutamine-limited chemostats using the same conditions as evolution experiments. No significant fitness defect was observed for either strain, indicating that constitutive expression of one or two copies of the fluorescent gene does not confer a fitness cost in these conditions. Error bars are 95% confidence intervals. Data and code used to generate this figure can be accessed in OSF: https://osf.io/fxhze/. CNV, copy number variant.
(PDF)

S2 Fig The *GAP1* CNV reporter indicates the emergence of *GAP1* CNVs in all glutamine-limited populations. Distributions of single-cell fluorescence over time for all glutamine-limited experimental populations. Fluorescent signal is normalized by forward scatter, which varies as a function of cell size. Each distribution is based on 100,000 single cell measurements. Data and code used to generate this figure can be accessed in OSF: https://osf.io/fxhze/. CNV, copy number variant.
(PDF)

S3 Fig Normalization by forward scatter mitigates effects of cell physiology and morphology variation on CNV reporter signal. Dashed gray lines represent one- and two-copy control populations. (A) Median unnormalized fluorescence across time for all evolving populations. (B) Median forward scatter over time for all populations. One glucose-limited population (pink) developed a bud separation defect, resulting in a cell aggregation phenotype and large forward scatter and fluorescence measurements. Normalizing by forward scatter accounts for this issue and other changes in overall cell physiology during the evolution experiments (see Fig 2B). Data and code used to generate this figure can be accessed in OSF: https://osf.io/fxhze/. CNV, copy number variant.
(PDF)

S4 Fig Gating flow cytometry data enables estimation of CNV alleles that contain more than two copies. The proportion of cells with zero, one, two, and three or more copies of *GAP1* in each glutamine-limited experimental population. Proportions were calculated after generating gating criteria based on one- and two-copy control populations. Data and code used to generate this figure can be accessed in OSF: https://osf.io/fxhze/. CNV, copy number variant.
(PDF)

S5 Fig Pulsed-field gel electrophoresis enables molecular characterization of *GAP1* CNVs. Analysis of ancestral and evolved clones. Whole chromosomes were visualized by ethidium bromide staining (left) and then probed for *GAP1* and *CEN11* (right). In the majority of cases, the *CEN11* probe correlates with *GAP1* probe signal, indicating that these *GAP1* amplifications are located on Chromosome XI. Instances when the *CEN11* and *GAP1* probes do not correlate are indicative of nonreciprocal translocations. Duplication of *CEN11* may indicate segmental aneuploidy. CNV, copy number variant.
(TIF)

S6 Fig *GAP1* CNV-containing lineages have a higher relative fitness than the ancestral strain. The fitness of evolved lineages containing *GAP1* CNVs was determined by pairwise competition experiments with a nonfluorescent, unevolved reference strain (FY4) in glutamine-limited chemostats. The majority (18/28) of evolved CNV-containing lineages have significantly higher fitness (*t* test, Bonferroni-corrected *p*-value < 0.00156) than the ancestor. Decreased (2/28) or insignificant fitness differences (8/28) may reflect context-specific fitness effects of *GAP1* CNV-containing lineages. Error bars are 95% confidence intervals. Data and code used to generate this figure can be accessed in OSF: https://osf.io/fxhze/. CNV, copy number variant.
(PDF)

S7 Fig Identification of CNV alleles at the *DUR3* locus. (A) A schematic illustrating the genomic context and estimated breakpoints for clones containing *DUR3* CNVs isolated from urea-limited chemostats at generation 150 and generation 250. Breakpoint boundaries were estimated using a read depth–based approach. Compared to (B) clones isolated from glutamine-limited chemostats containing *GAP1* CNVs, (C) clones isolated from urea-limited chemostats have a significantly higher copy number (*t* test *p*-value < 0.01). (D) *GAP1* CNV alleles are significantly larger than (E) *DUR3* CNV alleles (*t* test *p*-value < 0.01). Data and code used to generate this figure can be accessed in OSF: https://osf.io/fxhze/. ARS, autonomously replicating sequence; CNV, copy number variant.
(PDF)

S8 Fig Benchmarking existing CNV detection algorithms with simulated clonal samples. We simulated CNVs in the yeast genome at different average sequencing depths to assess the performance of CNVnator, LUMPY, Pindel, and SvABA. Algorithm performance was evaluated using and F-score. We find that with increased read depth, (A) the FDR increases for deletion detection, but (B) overall performance improves for all algorithms as determined by F-score. Conversely, for duplication detection, (C) the false positive rate is not increased with increasing read depth, and (D) overall performance improves with increased read depth. Data and code used to generate this figure can be accessed in OSF: https://osf.io/fxhze/. CNV, copy number variant; FDR, false discovery rate.
(PDF)

S9 Fig Benchmarking existing CNV detection algorithms with simulated heterogeneous population samples. We simulated heterogeneous populations containing CNVs at varying frequencies and assessed algorithm performance. Most algorithms perform reasonably well when CNVs are present at 50% or higher in the population. Data and code used to generate this figure can be accessed in OSF: https://osf.io/fxhze/. CNV, copy number variant.
(PDF)

S10 Fig Population estimates of *GAP1* copy number by CNV reporter and quantitative sequencing are linearly correlated and increase with time of adaptive evolution. Relative depth at the *GAP1* locus, calculated from whole-genome sequencing data, is strongly correlated with the median normalized fluorescence of the *GAP1* CNV reporter in populations. Glutamine-limited populations measured at generation 250 tend to have higher fluorescence and higher relative read depth at the *GAP1* locus than at generation 150. Data and code used to generate this figure can be accessed in OSF: https://osf.io/fxhze/. CNV, copy number variant.
(PDF)

S11 Fig Population prehistory of independent evolution experiments. All independent populations share a common history prior to founding of individual populations. The prehistory of experiments using the *GAP1* CNV reporter (A) differ with respect to the size of the founding population in experiments using a lineage-tracking library (B). Any variation that is introduced prior to founding of individual populations may contribute to the evolution of all populations. Variation that is introduced after separation into individual populations contributes to evolutionary outcomes in that population only. CNV, copy number variant; Gln-lim, glutamine limited; YPD, yeast extract-peptone-dextrose (rich media); YPGAL, yeast extract-peptone-galactose.
(PDF)

S12 Fig Distribution of barcode counts in ancestral populations. We determined the distribution of read counts supporting each unique barcode in the ancestral population, after filtering out low-confidence clusters. The relative frequencies of barcodes vary by over an order of magnitude, and we observe a long tail with a few barcodes significantly overrepresented in the ancestral population. The red arrow indicates an overrepresented barcode in the ancestral population that was identified in the CNV subpopulation in both independent barcoded evolution experiments (indicated in purple in Fig 5B). This distribution is consistent with that found in other barcode lineage-tracking experiments [68]. Data and code used to generate this figure can be accessed in OSF: https://osf.io/fxhze/. CNV, copy number variant.
(PDF)

S13 Fig Identification of barcoded *GAP1* CNV lineages in evolving populations. (A) *GAP1* CNV dynamics in barcoded populations assayed using a CNV reporter. (B) Estimation of true positive rate of CNV isolation by FACS at generations 70, 90, and 150. CNV subpopulations were isolated by FACS at each time point and clones isolated by plating for single colonies. The percentage of cells containing a CNV in the fractionated subpopulation was estimated using at least 25 clones. A one-copy control strain was used to define gates. Data and code used to generate this figure can be accessed in OSF: https://osf.io/fxhze/. CNV, copy number variant; FACS, fluorescence-activated cell sorting.
(PDF)

S14 Fig FACS reports for isolation of CNV subpopulation. Reports for CNV subpopulation isolation at generation 70 (A–E), 90 (F–J), 150 (K–O), and 270 (P–T). Gates were drawn based on zero-, one-, and two-copy control populations, and cells were isolated from the P4 population. CNV, copy number variant; FACS, fluorescence-activated cell sorting.
(PDF)

S1 Table List of strains used and generated in this study. (XLSX)

S2 Table List of all experimentally evolved populations. RD, read depth.
(XLSX)

S3 Table DNA sequencing summary statistics for all clonal and population samples. PF, pass filter.
(XLSX)

S4 Table Summary statistics of all evolved clones. Nucleotide resolution of CNV boundaries and size (in kilobases) of CNV alleles are presented. These metrics were calculated using an RD-based approach and were used to generate Fig 3. CNV, copy number variant; RD, read depth.
(XLSX)

S5 Table Breakpoint analysis of 29 *GAP1* CNVs and 9 *DUR3* CNVs. We compare all 3 CNV detection methods used in this study: breakpoint sequences determined through split read assembly and alignment, breakpoint identification using LUMPY, and CNV boundary classification using read depth and visual inspection. Left and right refers to breakpoint position relative to the location of *GAP1* or *DUR3* on the chromosome. A single event on either the left or right side can be represented by two or more nucleotide coordinates when a breakpoint is determined from split or discordant reads spanning a novel junction; see S2 Text. CNV, copy number variant.
(XLSX)

S6 Table Summary of CNV detection algorithm performance for all population samples. Data and code used to generate this table can be accessed in OSF: https://osf.io/fxhze/. BND, breakend; CNV, copy number variant; RD, read depth.
(XLSX)

S7 Table Additional structural variants identified by de novo assembly of split reads. (XLSX)

S8 Table Additional copy number variants identified using a read depth–based approach. (XLSX)

S9 Table SNVs identified from population sequencing data. If an SNV was identified at both time points, we indicated the trend: increases in frequency, decreases in frequency, or frequency remaining steady. SNVs present at frequencies greater than 0.05 are reported. CNV, copy number variant; SNV, single-nucleotide variant.
(XLSX)

S10 Table SNVs identified from clone sequencing data. We indicated SNVs that were identified in the boundaries of a *GAP1* or *DUR3* CNV. SNVs were filtered on the basis of their frequency in the clonal sequence data using a threshold of 0.25. CNV, copy number variant; SNV, single-nucleotide variant.
(XLSX)

S11 Table Summary statistics for *GAP1* CNV dynamics, determined using the *GAP1* CNV reporter, in replicated evolution experiments using lineage-tracking libraries. Summary statistics are defined as in Table 1. Data and code used to generate this table can be accessed in OSF: https://osf.io/fxhze/. CNV, copy number variant.
(XLSX)

S12 Table List of primers used to generate PCR products for strain construction, to confirm insertion of PCR products after transformation, and to generate probes for Southern hybridization. (XLSX)

## Abbreviations

AAA: ATPase associated with diverse cellular activities
a.u.: arbitrary units
BIR: break-induced replication
CNV: copy number variant
DSB: double-strand break
FACS: fluorescence-activated cell sorting
FoSTes: fork stalling and template switching
GFP: green fluorescent protein
LTR: long terminal repeat
MMBIR: microhomology-mediated break-induced replication
N/A: not applicable
NAHR: nonallelic homologous recombination
NHEJ: nonhomologous end joining
ODIRA: origin-dependent inverted repeat amplification
qPCR: quantitative PCR
RAM: regulation of Ace2p activity and cellular morphogenesis
rDNA: ribosomal DNA
SNV: single-nucleotide variant

## References

[1] Conant GC, Wolfe KH. Turning a hobby into a job: how duplicated genes find new functions. Nat Rev Genet. 2008;9: 938–950. 10.1038/nrg2482 19015656

[2] Zuellig MP, Sweigart AL. Gene duplicates cause hybrid lethality between sympatric species of Mimulus. PLoS Genet. 2018;14: e1007130 10.1371/journal.pgen.1007130 29649209 PMC5896889

[3] Shlien A, Malkin D. Copy number variations and cancer. Genome Med. 2009;1: 62–62. 10.1186/gm62 19566914 PMC2703871

[4] Stratton MR, Campbell PJ, Futreal PA. The cancer genome. Nature. 2009;458: 719–724. 10.1038/nature07943 19360079 PMC2821689

[5] Barreiro LB, Laval G, Quach H, Patin E, Quintana-Murci L. Natural selection has driven population differentiation in modern humans. Nat Genet. 2008;40: 340–345. 10.1038/ng.78 18246066

[6] Iskow RC, Gokcumen O, Abyzov A, Malukiewicz J, Zhu Q, Sukumar AT, et al Regulatory element copy number differences shape primate expression profiles. Proc Natl Acad Sci U S A. 2012;109: 12656–12661. 10.1073/pnas.1205199109 22797897 PMC3411951

[7] Clop A, Vidal O, Amills M. Copy number variation in the genomes of domestic animals. Anim Genet. 2012;43: 503–517. 10.1111/j.1365-2052.2012.02317.x 22497594

[8] Żmieńko A, Samelak A, Kozłowski P, Figlerowicz M. Copy number polymorphism in plant genomes. Theor Appl Genet. 2014;127: 1–18. 10.1007/s00122-013-2177-7 23989647 PMC4544587

[9] Greenblum S, Carr R, Borenstein E. Extensive strain-level copy-number variation across human gut microbiome species. Cell. 2015;160: 583–594. 10.1016/j.cell.2014.12.038 25640238 PMC4507803

[10] Zarrei M, MacDonald JR, Merico D, Scherer SW. A copy number variation map of the human genome. Nat Rev Genet. 2015;16: 172–183. 10.1038/nrg3871 25645873

[11] Ohno S. Evolution by Gene Duplication. Berlin, Heidelberg: Springer Berlin Heidelberg; 1970.

[12] Lynch M, Conery JS. The Evolutionary Fate and Consequences of Duplicate Genes. Science. 2000;290. 10.1126/science.290.5494.1151 11073452

[13] Hughes AL. The Evolution of Functionally Novel Proteins after Gene Duplication. Proceedings of the Royal Society of London B: Biological Sciences. 1994;256. 10.1098/rspb.1994.0058 8029240

[14] Anderson RP, Roth JR. Tandem Genetic Duplications in Phage and Bacteria. Annu Rev Microbiol. 1977;31: 473–505. 10.1146/annurev.mi.31.100177.002353 334045

[15] Iantorno SA, Durrant C, Khan A, Sanders MJ, Beverley SM, Warren WC, et al Gene Expression in Leishmania Is Regulated Predominantly by Gene Dosage. MBio. 2017;8 10.1128/mBio.01393-17 28900023 PMC5596349

[16] Cowell AN, Istvan ES, Lukens AK, Gomez-Lorenzo MG, Vanaerschot M, Sakata-Kato T, et al Mapping the malaria parasite druggable genome by using in vitro evolution and chemogenomics. Science. American Association for the Advancement of Science; 2018;359: 191–199. 10.1126/science.aan4472 PMC5925756 29326268

[17] Dolatabadian A, Patel DA, Edwards D, Batley J. Copy number variation and disease resistance in plants. Theor Appl Genet. 2017;130: 2479–2490. 10.1007/s00122-017-2993-2 29043379

[18] Elde NC, Child SJ, Eickbush MT, Kitzman JO, Rogers KS, Shendure J, et al Poxviruses deploy genomic accordions to adapt rapidly against host antiviral defenses. Cell. 2012;150: 831–841. 10.1016/j.cell.2012.05.049 22901812 PMC3499626

[19] Lenski RE, Rose MR, Simpson SC, Tadler SC. Long-Term Experimental Evolution in Escherichia coli. I. Adaptation and Divergence During 2,000 Generations. Am Nat. 1991;138: 1315–1341.

[20] Good BH, McDonald MJ, Barrick JE, Lenski RE, Desai MM. The dynamics of molecular evolution over 60,000 generations. Nature. 2017;551: 45–50. 10.1038/nature24287 29045390 PMC5788700

[21] Gresham D, Dunham MJ. The enduring utility of continuous culturing in experimental evolution. Genomics. 2014;104: 399–405. 10.1016/j.ygeno.2014.09.015 25281774 PMC4411559

[22] Horiuchi T, Horiuchi S, Novick A. The genetic basis of hyper-synthesis of beta-galactosidase. Genetics. 1963;48: 157–169. 13954911 10.1093/genetics/48.2.157 PMC1210458

[23] Sonti RV, Roth JR. Role of gene duplications in the adaptation of Salmonella typhimurium to growth on limiting carbon sources. Genetics. 1989;123: 19–28. 2680755 10.1093/genetics/123.1.19 PMC1203782

[24] Hong J, Gresham D. Molecular specificity, convergence and constraint shape adaptive evolution in nutrient-poor environments. PLoS Genet. 2014;10: e1004041–e1004041. 10.1371/journal.pgen.1004041 24415948 PMC3886903

[25] Gresham D, Usaite R, Germann SM, Lisby M, Botstein D, Regenberg B. Adaptation to diverse nitrogen-limited environments by deletion or extrachromosomal element formation of the GAP1 locus. Proc Natl Acad Sci U S A. 2010;107: 18551–18556. 10.1073/pnas.1014023107 20937885 PMC2972935

[26] Payen C, Di Rienzi SC, Ong GT, Pogachar JL, Sanchez JC, Sunshine AB, et al The dynamics of diverse segmental amplifications in populations of Saccharomyces cerevisiae adapting to strong selection. G3. 2014;4: 399–409. 10.1534/g3.113.009365 24368781 PMC3962480

[27] Gresham D, Desai MM, Tucker CM, Jenq HT, Pai DA, Ward A, et al The repertoire and dynamics of evolutionary adaptations to controlled nutrient-limited environments in yeast. PLoS Genet. 20084: e1000303–e1000303. 10.1371/journal.pgen.1000303 PMC2586090 19079573

[28] Brown CJ, Todd KM, Rosenzweig RF. Multiple duplications of yeast hexose transport genes in response to selection in a glucose-limited environment. Mol Biol Evol. 1998;15: 931–942. 10.1093/oxfordjournals.molbev.a026009 9718721

[29] Kao KC, Sherlock G. Molecular characterization of clonal interference during adaptive evolution in asexual populations of Saccharomyces cerevisiae. Nat Genet. 2008;40: 1499–1504. 10.1038/ng.280 19029899 PMC2596280

[30] Hansche PE. Gene duplication as a mechanism of genetic adaptation in Saccharomyces cerevisiae. Genetics. 1975;79: 661–674. 236976 10.1093/genetics/79.4.661 PMC1213303

[31] Kvitek DJ, Sherlock G. Reciprocal sign epistasis between frequently experimentally evolved adaptive mutations causes a rugged fitness landscape. PLoS Genet. 2011;7: e1002056–e1002056. 10.1371/journal.pgen.1002056 21552329 PMC3084205

[32] Dorsey M, Peterson C, Bray K, Paquin CE. Spontaneous amplification of the ADH4 gene in Saccharomyces cerevisiae. Genetics. 1992;132: 943–950. 1459445 10.1093/genetics/132.4.943 PMC1205250

[33] Lynch M, Sung W, Morris K, Coffey N, Landry CR, Dopman EB, et al A genome-wide view of the spectrum of spontaneous mutations in yeast. Proc Natl Acad Sci U S A. 2008;105: 9272–9277. 10.1073/pnas.0803466105 18583475 PMC2453693

[34] Lang GI, Rice DP, Hickman MJ, Sodergren E, Weinstock GM, Botstein D, et al Pervasive genetic hitchhiking and clonal interference in forty evolving yeast populations. Nature. 2013;500: 571–574. 10.1038/nature12344 23873039 PMC3758440

[35] Hughes JM, Lohman BK, Deckert GE, Nichols EP, Settles M, Abdo Z, et al The role of clonal interference in the evolutionary dynamics of plasmid-host adaptation. MBio. 2012;3: e00077–12. 10.1128/mBio.00077-12 22761390 PMC3398533

[36] Maddamsetti R, Lenski RE, Barrick JE. Adaptation, Clonal Interference, and Frequency-Dependent Interactions in a Long-Term Evolution Experiment with Escherichia coli. Genetics. 2015; 10.1534/genetics.115.176677 25911659 PMC4492384

[37] Grenson M, Hou C, Crabeel M. Multiplicity of the amino acid permeases in Saccharomyces cerevisiae. IV. Evidence for a general amino acid permease. J Bacteriol. 1970;103: 770–777. 5474888 10.1128/jb.103.3.770-777.1970 PMC248157

[38] Stanbrough M, Magasanik B. Transcriptional and posttranslational regulation of the general amino acid permease of Saccharomyces cerevisiae. J Bacteriol. 1995;177: 94–102. 7798155 10.1128/jb.177.1.94-102.1995 PMC176561

[39] Dhami MK, Hartwig T, Fukami T. Genetic basis of priority effects: insights from nectar yeast. Proc Biol Sci. 2016;283 10.1098/rspb.2016.1455 27708148 PMC5069511

[40] Hastings PJ, Lupski JR, Rosenberg SM, Ira G. Mechanisms of change in gene copy number. Nat Rev Genet. 2009;10: 551–564. 10.1038/nrg2593 19597530 PMC2864001

[41] Reams AB, Roth JR. Mechanisms of gene duplication and amplification. Cold Spring Harb Perspect Biol. 2015;7: a016592 10.1101/cshperspect.a016592 25646380 PMC4315931

[42] Carvalho CMB, Lupski JR. Mechanisms underlying structural variant formation in genomic disorders. Nat Rev Genet. 2016;17: 224–238. 10.1038/nrg.2015.25 26924765 PMC4827625

[43] Stankiewicz P, Lupski JR. Genome architecture, rearrangements and genomic disorders. Trends Genet. 2002;18: 74–82. 11818139 10.1016/s0168-9525(02)02592-1

[44] Lee JA, Carvalho CMB, Lupski JR. A DNA replication mechanism for generating nonrecurrent rearrangements associated with genomic disorders. Cell. 2007;131: 1235–1247. 10.1016/j.cell.2007.11.037 18160035

[45] Hastings PJ, Ira G, Lupski JR, Iafrate AJ, Feuk L, Rivera MN, et al A Microhomology-Mediated Break-Induced Replication Model for the Origin of Human Copy Number Variation. PLoS Genet. 2009;5: e1000327–e1000327. 10.1371/journal.pgen.1000327 19180184 PMC2621351

[46] Payen C, Koszul R, Dujon B, Fischer G, Bailey JA, Eichler EE, et al Segmental Duplications Arise from Pol32-Dependent Repair of Broken Forks through Two Alternative Replication-Based Mechanisms. PLoS Genet. 2008;4: e1000175–e1000175. 10.1371/journal.pgen.1000175 18773114 PMC2518615

[47] Brewer BJ, Payen C, Raghuraman MK, Dunham MJ. Origin-dependent inverted-repeat amplification: a replication-based model for generating palindromic amplicons. PLoS Genet. 2011;7: e1002016–e1002016. 10.1371/journal.pgen.1002016 21437266 PMC3060070

[48] Brewer BJ, Payen C, Di Rienzi SC, Higgins MM, Ong G, Dunham MJ, et al Origin-Dependent Inverted-Repeat Amplification: Tests of a Model for Inverted DNA Amplification. PLoS Genet. 2015;11: e1005699–e1005699. 10.1371/journal.pgen.1005699 26700858 PMC4689423

[49] Møller HD, Parsons L, Jørgensen TS, Botstein D, Regenberg B. Extrachromosomal circular DNA is common in yeast. Proc Natl Acad Sci U S A. 2015;112: E3114–22. 10.1073/pnas.1508825112 26038577 PMC4475933

[50] Turner KM, Deshpande V, Beyter D, Koga T, Rusert J, Lee C, et al Extrachromosomal oncogene amplification drives tumour evolution and genetic heterogeneity. Nature. 2017;543: 122–125. 10.1038/nature21356 28178237 PMC5334176

[51] Møller HD, Andersen KS, Regenberg B. A model for generating several adaptive phenotypes from a single genetic event: Saccharomyces cerevisiae GAP1 as a potential bet-hedging switch. Commun Integr Biol. 2013;6: e23933 10.4161/cib.23933 23713139 PMC3656021

[52] Cohen S, Segal D. Extrachromosomal circular DNA in eukaryotes: possible involvement in the plasticity of tandem repeats. Cytogenet Genome Res. 2009;124: 327–338. 10.1159/000218136 19556784

[53] Suzuki Y, St Onge RP, Mani R, King OD, Heilbut A, Labunskyy VM, et al Knocking out multigene redundancies via cycles of sexual assortment and fluorescence selection. Nat Methods. 2011;8: 159–164. 10.1038/nmeth.1550 21217751 PMC3076670

[54] Gruber JD, Vogel K, Kalay G, Wittkopp PJ. Contrasting properties of gene-specific regulatory, coding, and copy number mutations in Saccharomyces cerevisiae: frequency, effects, and dominance. PLoS Genet. 2012;8: e1002497 10.1371/journal.pgen.1002497 22346762 PMC3276545

[55] Kafri M, Metzl-Raz E, Jona G, Barkai N. The Cost of Protein Production. Cell Rep. 2016;14: 22–31. 10.1016/j.celrep.2015.12.015 26725116 PMC4709330

[56] Steinrueck M, Guet CC. Complex chromosomal neighborhood effects determine the adaptive potential of a gene under selection. Elife. 2017;6 10.7554/eLife.25100 28738969 PMC5526668

[57] Griesbeck O, Baird GS, Campbell RE, Zacharias DA, Tsien RY. Reducing the environmental sensitivity of yellow fluorescent protein. Mechanism and applications. J Biol Chem. 2001;276: 29188–29194. 10.1074/jbc.M102815200 11387331

[58] Airoldi EM, Miller D, Athanasiadou R, Brandt N, Abdul-Rahman F, Neymotin B, et al Steady-state and dynamic gene expression programs in Saccharomyces cerevisiae in response to variation in environmental nitrogen. Mol Biol Cell. 2016;27: 1383–1396. 10.1091/mbc.E14-05-1013 26941329 PMC4831890

[59] Stanbrough M, Magasanik B. Two transcription factors, Gln3p and Nil1p, use the same GATAAG sites to activate the expression of GAP1 of Saccharomyces cerevisiae. J Bacteriol. 1996;178: 2465–2468. 8636059 10.1128/jb.178.8.2465-2468.1996 PMC177966

[60] Lang GI, Botstein D, Desai MM. Genetic variation and the fate of beneficial mutations in asexual populations. Genetics. 2011;188: 647–661. 10.1534/genetics.111.128942 21546542 PMC3176544

[61] Ye K, Schulz MH, Long Q, Apweiler R, Ning Z. Pindel: a pattern growth approach to detect break points of large deletions and medium sized insertions from paired-end short reads. Bioinformatics. 2009;25: 2865–2871. 10.1093/bioinformatics/btp394 19561018 PMC2781750

[62] Layer RM, Chiang C, Quinlan AR, Hall IM, Alkan C, Coe BP, et al LUMPY: a probabilistic framework for structural variant discovery. Genome Biol. 2014;15: R84–R84. 10.1186/gb-2014-15-6-r84 24970577 PMC4197822

[63] Wala J, Bandopadhayay P, Greenwald NF, O’Rourke R, Sharpe T, Stewart C, et al SvABA: genome-wide detection of structural variants and indels by local assembly. Genome Res. 2018;28: 581–591. 10.1101/gr.221028.117 29535149 PMC5880247

[64] Abyzov A, Urban AE, Snyder M, Gerstein M. CNVnator: An approach to discover, genotype, and characterize typical and atypical CNVs from family and population genome sequencing. Genome Res. 2011;21: 974–984. 10.1101/gr.114876.110 21324876 PMC3106330

[65] Cullen PJ, Sprague GF Jr. The regulation of filamentous growth in yeast. Genetics. 2012;190: 23–49. 10.1534/genetics.111.127456 22219507 PMC3249369

[66] Torres EM, Dephoure N, Panneerselvam A, Tucker CM, Whittaker CA, Gygi SP, et al Identification of aneuploidy-tolerating mutations. Cell. 2010;143: 71–83. 10.1016/j.cell.2010.08.038 20850176 PMC2993244

[67] Payen C, Sunshine AB, Ong GT, Pogachar JL, Zhao W, Dunham MJ. High-Throughput Identification of Adaptive Mutations in Experimentally Evolved Yeast Populations. PLoS Genet. 2016;12: e1006339 10.1371/journal.pgen.1006339 27727276 PMC5065121

[68] Levy SF, Blundell JR, Venkataram S, Petrov DA, Fisher DS, Sherlock G. Quantitative evolutionary dynamics using high-resolution lineage tracking. Nature. 2015;519: 181–186. 10.1038/nature14279 25731169 PMC4426284

[69] Zhao L, Liu Z, Levy SF, Wu S. Bartender: a fast and accurate clustering algorithm to count barcode reads. Bioinformatics. 2017; 10.1093/bioinformatics/btx655 29069318 PMC6049041

[70] Blundell JR, Schwartz K, Francois D, Fisher DS, Sherlock GJ, Levy SF. The dynamics of adaptive genetic diversity during the early stages of clonal evolution [Internet]. bioRxiv. 2017 p. 170589 10.1101/170589 PMC6517070 30598529

[71] Adler M, Anjum M, Berg OG, Andersson DI, Sandegren L. High fitness costs and instability of gene duplications reduce rates of evolution of new genes by duplication-divergence mechanisms. Mol Biol Evol. 2014;31: 1526–1535. 10.1093/molbev/msu111 24659815

[72] Natesuntorn W, Iwami K, Matsubara Y, Sasano Y, Sugiyama M, Kaneko Y, et al Genome-wide construction of a series of designed segmental aneuploids in Saccharomyces cerevisiae. Sci Rep. 2015;5: 12510 10.1038/srep12510 26224198 PMC4519793

[73] Selmecki A, Forche A, Berman J. Aneuploidy and isochromosome formation in drug-resistant Candida albicans. Science. 2006;313: 367–370. 10.1126/science.1128242 16857942 PMC1717021

[74] Zhang F, Khajavi M, Connolly AM, Towne CF, Batish SD, Lupski JR. The DNA replication FoSTeS/MMBIR mechanism can generate genomic, genic and exonic complex rearrangements in humans. Nat Genet. 2009;41: 849–853. 10.1038/ng.399 19543269 PMC4461229

[75] Ottaviani D, LeCain M, Sheer D. The role of microhomology in genomic structural variation. Trends Genet. 2014;30: 85–94. 10.1016/j.tig.2014.01.001 24503142

[76] Arlt MF, Rajendran S, Birkeland SR, Wilson TE, Glover TW. De novo CNV formation in mouse embryonic stem cells occurs in the absence of Xrcc4-dependent nonhomologous end joining. PLoS Genet. 2012;8: e1002981 10.1371/journal.pgen.1002981 23028374 PMC3447954

[77] Sakofsky CJ, Ayyar S, Deem AK, Chung W-H, Ira G, Malkova A. Translesion Polymerases Drive Microhomology-Mediated Break-Induced Replication Leading to Complex Chromosomal Rearrangements. Mol Cell. 2015;60: 860–872. 10.1016/j.molcel.2015.10.041 26669261 PMC4688117

[78] Foster PL. Stress-induced mutagenesis in bacteria. Crit Rev Biochem Mol Biol. 2007;42: 373–397. 10.1080/10409230701648494 17917873 PMC2747772

[79] Galhardo RS, Hastings PJ, Rosenberg SM. Mutation as a stress response and the regulation of evolvability. Crit Rev Biochem Mol Biol. 2007;42: 399–435. 10.1080/10409230701648502 17917874 PMC3319127

[80] Shor E, Fox CA, Broach JR. The yeast environmental stress response regulates mutagenesis induced by proteotoxic stress. PLoS Genet. 2013;9: e1003680 10.1371/journal.pgen.1003680 23935537 PMC3731204

[81] Chen L, Zhou W, Zhang C, Lupski JR, Jin L, Zhang F. CNV instability associated with DNA replication dynamics: evidence for replicative mechanisms in CNV mutagenesis. Hum Mol Genet. 2015;24: 1574–1583. 10.1093/hmg/ddu572 25398944 PMC4381758

[82] Wilson TE, Arlt MF, Park SH, Rajendran S, Paulsen M, Ljungman M, et al Large transcription units unify copy number variants and common fragile sites arising under replication stress. Genome Res. 2015;25: 189–200. 10.1101/gr.177121.114 25373142 PMC4315293

[83] Thomas BJ, Rothstein R. Elevated recombination rates in transcriptionally active DNA. Cell. 1989;56: 619–630. 2645056 10.1016/0092-8674(89)90584-9

[84] Skourti-Stathaki K, Proudfoot NJ. A double-edged sword: R loops as threats to genome integrity and powerful regulators of gene expression. Genes Dev. 2014;28: 1384–1396. 10.1101/gad.242990.114 24990962 PMC4083084

[85] Aguilera A, Gaillard H. Transcription and recombination: when RNA meets DNA. Cold Spring Harb Perspect Biol. 2014;6: a016543–a016543. 10.1101/cshperspect.a016543 25085910 PMC4107990

[86] Jack CV, Cruz C, Hull RM, Keller MA, Ralser M, Houseley J. Regulation of ribosomal DNA amplification by the TOR pathway. Proc Natl Acad Sci U S A. 2015;112: 9674–9679. 10.1073/pnas.1505015112 26195783 PMC4534215

[87] Mansisidor AR, Molinar T Jr, Srivastava P, Dartis DD, Pino Delgado A, Blitzblau HG, et al Genomic copy-number loss is rescued by self-limiting production of DNA circles. Mol Cell. 2018;72: 583–593.e4. 10.1016/j.molcel.2018.08.036 30293780 PMC6214758

[88] Hull RM, Cruz C, Jack CV, Houseley J. Environmental change drives accelerated adaptation through stimulated copy number variation. PLoS Biol. 2017;15: e2001333 10.1371/journal.pbio.2001333 28654659 PMC5486974

[89] Gietz RD, Schiestl RH. High-efficiency yeast transformation using the LiAc/SS carrier DNA/PEG method. Nat Protoc. 2007;2: 31–34. 10.1038/nprot.2007.13 17401334

[90] Brauer MJ, Huttenhower C, Airoldi EM, Rosenstein R, Matese JC, Gresham D, et al Coordination of growth rate, cell cycle, stress response, and metabolic activity in yeast. Mol Biol Cell. 2008;19: 352–367. 10.1091/mbc.E07-08-0779 17959824 PMC2174172

[91] Miller AW, Befort C, Kerr EO, Dunham MJ. Design and use of multiplexed chemostat arrays. J Vis Exp. 2013; e50262 10.3791/50262 23462663 PMC3610398

[92] Ellis B, Haaland P, Hahne F, Meur NL, Gopalakrishnan N, Spidlen J And Jiang. flowCore: flowCore: Basic structures for flow cytometry data. 2016.

[93] Hoffman CS, Winston F. A ten-minute DNA preparation from yeast efficiently releases autonomous plasmids for transformation of Escherichia coli. Gene. 1987;57: 267–272. 3319781 10.1016/0378-1119(87)90131-4

[94] Baym M, Kryazhimskiy S, Lieberman TD, Chung H, Desai MM, Kishony R, et al Inexpensive Multiplexed Library Preparation for Megabase-Sized Genomes. PLoS ONE. 2015;10: e0128036–e0128036. 10.1371/journal.pone.0128036 26000737 PMC4441430

[95] Li H, Durbin R. Fast and accurate long-read alignment with Burrows-Wheeler transform. Bioinformatics. 2010;26: 589–595. 10.1093/bioinformatics/btp698 20080505 PMC2828108

[96] Li H, Handsaker B, Wysoker A, Fennell T, Ruan J, Homer N, et al The Sequence Alignment/Map format and SAMtools. Bioinformatics. 2009;25: 2078–2079. 10.1093/bioinformatics/btp352 19505943 PMC2723002

[97] Jeffares DC, Jolly C, Hoti M, Speed D, Shaw L, Rallis C, et al Transient structural variations have strong effects on quantitative traits and reproductive isolation in fission yeast. Nat Commun. 2017;8: 14061 10.1038/ncomms14061 28117401 PMC5286201

[98] Blair DC. Information Retrieval, 2nd ed. Van Rijsbergen C.J. London: Butterworths; 1979: 208 pp. J Am Soc Inf Sci. 1979;30: 374–375.

[99] Faust GG, Hall IM. SAMBLASTER: fast duplicate marking and structural variant read extraction. Bioinformatics. 2014;30: 2503–2505. 10.1093/bioinformatics/btu314 24812344 PMC4147885

[100] Katoh K, Standley DM. MAFFT multiple sequence alignment software version 7: improvements in performance and usability. Mol Biol Evol. 2013;30: 772–780. 10.1093/molbev/mst010 23329690 PMC3603318

[101] Rice P, Longden I, Bleasby A. EMBOSS: the European Molecular Biology Open Software Suite. Trends Genet. 2000;16: 276–277. 10827456 10.1016/s0168-9525(00)02024-2

[102] Baxevanis AD, Davison DB, Page RDM, Petsko GA, Stein LD, Stormo GD, editors. Using the Velvet de novo Assembler for Short-Read Sequencing Technologies. Current Protocols in Bioinformatics. Hoboken, NJ, USA: John Wiley & Sons; 2002 p. 810.

[103] Camacho C, Coulouris G, Avagyan V, Ma N, Papadopoulos J, Bealer K, et al BLAST+: architecture and applications. BMC Bioinformatics. 2009;10: 421 10.1186/1471-2105-10-421 20003500 PMC2803857

[104] McKenna A, Hanna M, Banks E, Sivachenko A, Cibulskis K, Kernytsky A, et al The Genome Analysis Toolkit: a MapReduce framework for analyzing next-generation DNA sequencing data. Genome Res. 2010;20: 1297–1303. 10.1101/gr.107524.110 20644199 PMC2928508

