# Supplementary material for: Single-cell copy number variant detection reveals the dynamics and diversity of adaptation
Source: PLoS Biol. 2018 Dec 18;16(12):e3000069. doi: 10.1371/journal.pbio.3000069 (PMC6298651; doi:10.1371/journal.pbio.3000069)
Supplement: S1 Text — Graphic representation of linear fit (and corresponding R2 values) during initial population expansion of CNV alleles. Slope of the linear fit corresponds to the dynamics parameter Sup shown in Table 1 and was calculated for the original evolution experiment and the barcode experiment. Data and code used to generate these figures can be accessed in OSF: https://osf.io/fxhze/. CNV, copy number variant. (PDF) [file pbio.3000069.s001.pdf]

Single-cell copy number variant detection reveals the dynamics and diversity of adaptation: S1 Text

Stephanie Lauer, Grace Avecilla, Pieter Spealman, Gunjan Sethia, Nathan Brandt, Sasha Levy and David Gresham

Compiled 2018-10-31

Contents

1 Quantification of CNV subpopulation dynamics for original evolution experiment 1

2 Quantification of CNV subpopulation dynamics for barcode evolution experiment 10

We performed linear regression to determine the relative fitness of the CNV subpopulation in each evolving population. The linear phase was determined using the variance explained (R^2) as indicated for each population below.

1 Quantification of CNV subpopulation dynamics for original evolution experiment

```
##
## Call:
## lm(formula = logECNV_noCNV ~ Generation, data = test)
##
## Coefficients:
## (Intercept)   Generation
##    -7.21808      0.06588
```

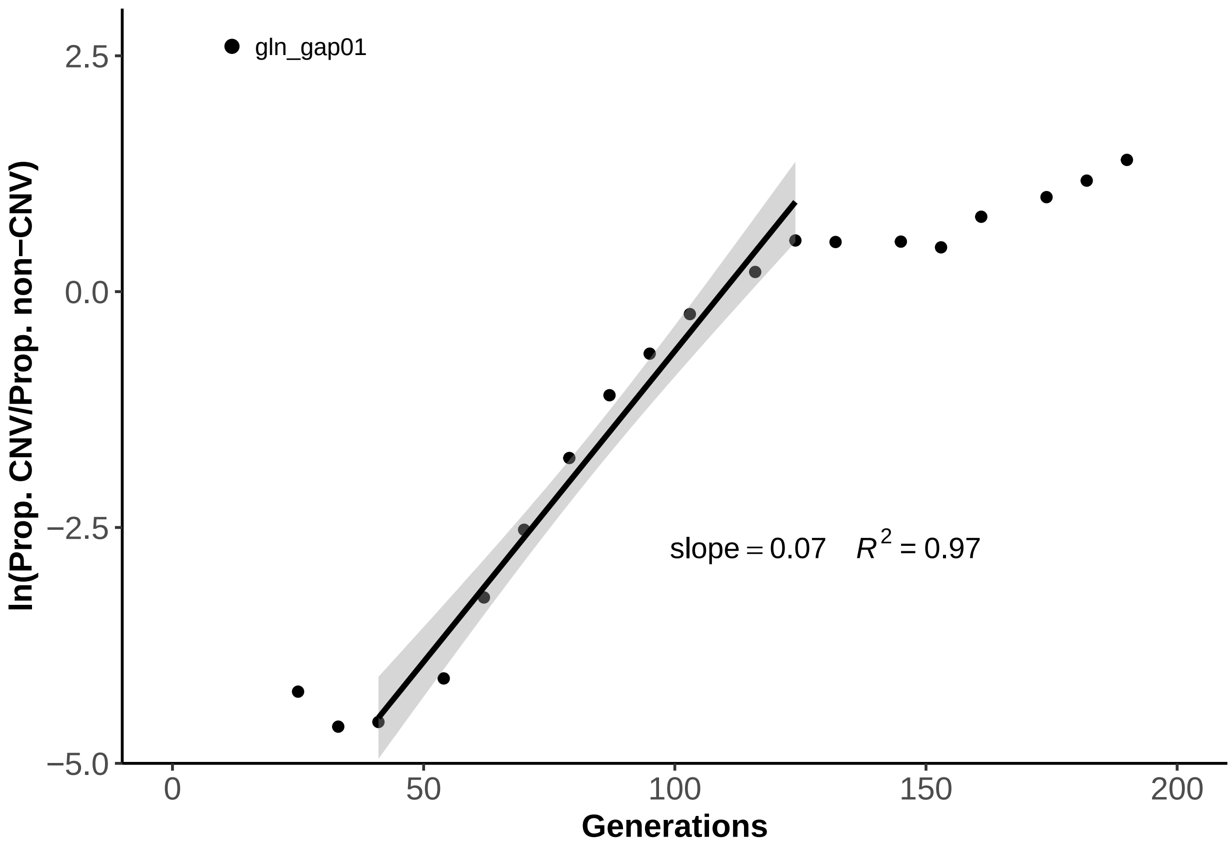


```
## [1] 0.003828795
```

```
##                  2.5 %     97.5 %
## Generation 0.05705222 0.07471065
```

```
##
## Call:
## lm(formula = logECNV_noCNV ~ Generation, data = test)
##
## Coefficients:
## (Intercept)   Generation
##     -7.9143       0.0711
```

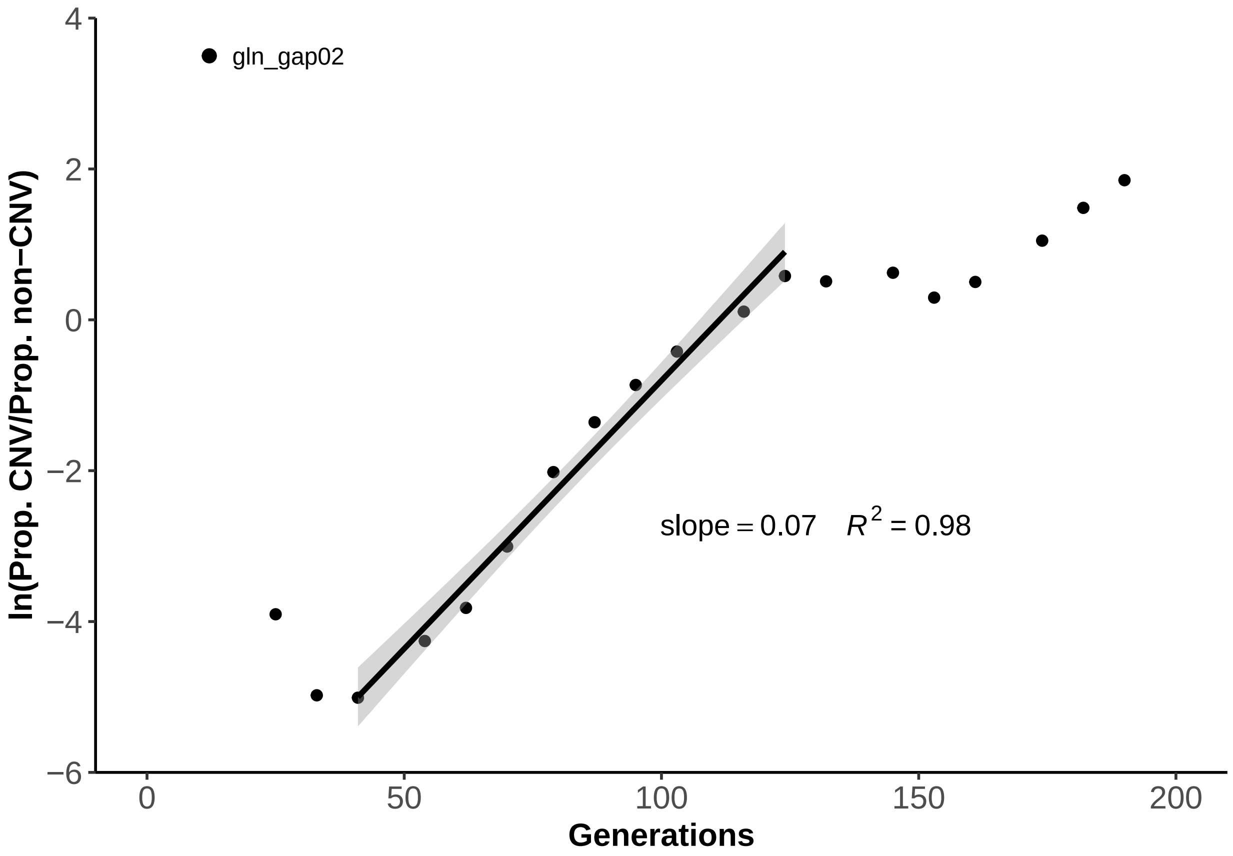


```
## [1] 0.003440376

##                  2.5 %     97.5 %
## Generation 0.06316211 0.07902915

##
## Call:
## lm(formula = logECNV_noCNV ~ Generation, data = test)
##
## Coefficients:
## (Intercept)   Generation
##    -7.11582      0.07131
```

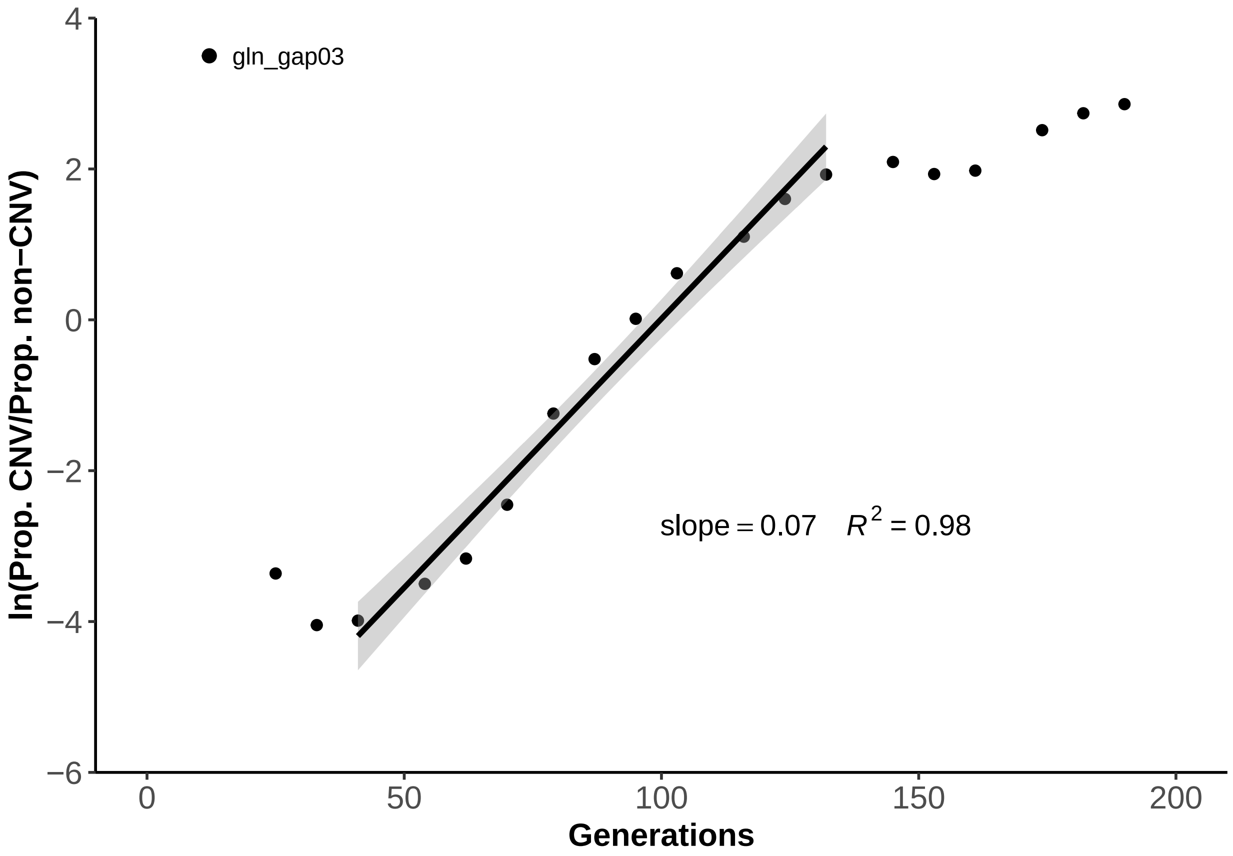


```
## [1] 0.003686966

##                  2.5 %     97.5 %
## Generation 0.06296517 0.07964616

##
## Call:
## lm(formula = logECNV_noCNV ~ Generation, data = test)
##
## Coefficients:
## (Intercept)   Generation
##    -7.88506      0.07932
```

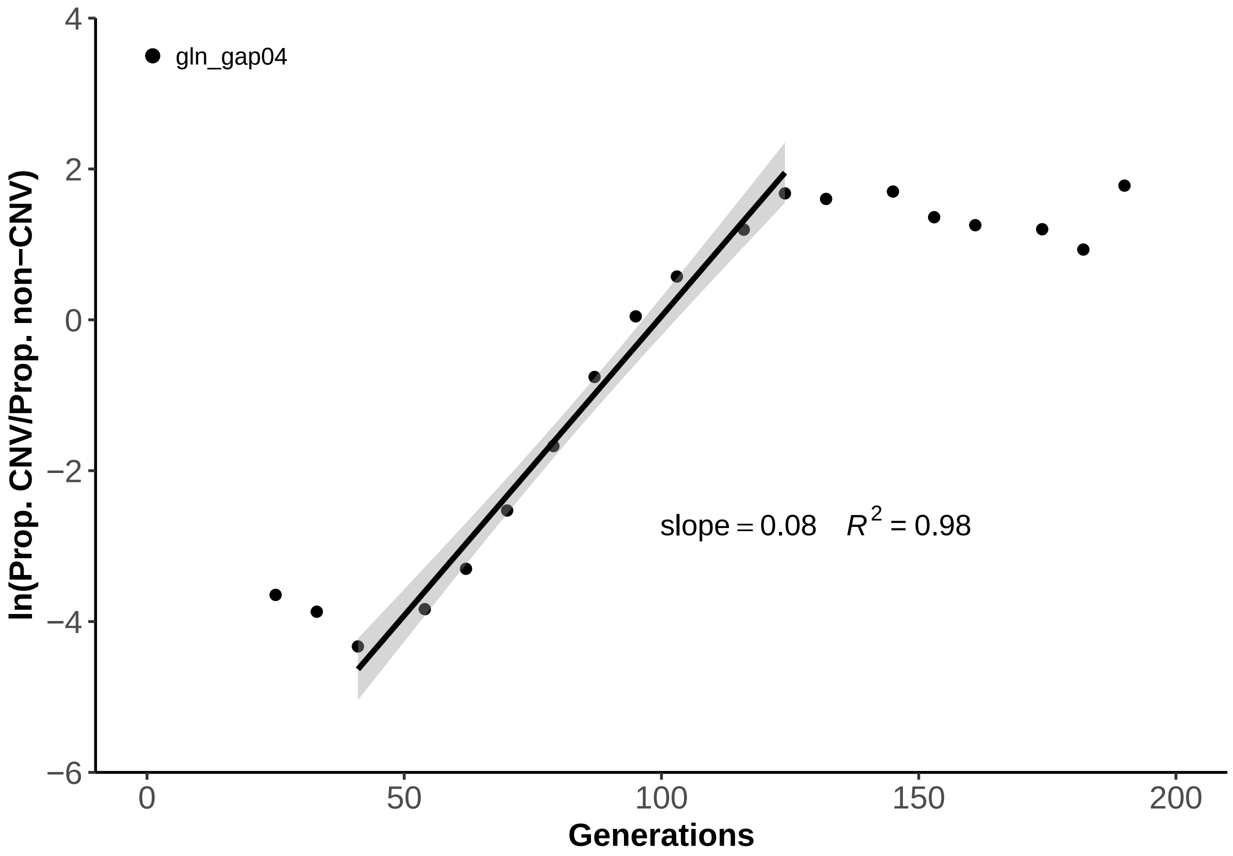


```
## [1] 0.003598147

##                  2.5 %     97.5 %
## Generation 0.07102378 0.08761847

##
## Call:
## lm(formula = logECNV_noCNV ~ Generation, data = test)
##
## Coefficients:
## (Intercept)   Generation
##    -7.92949      0.07674
```

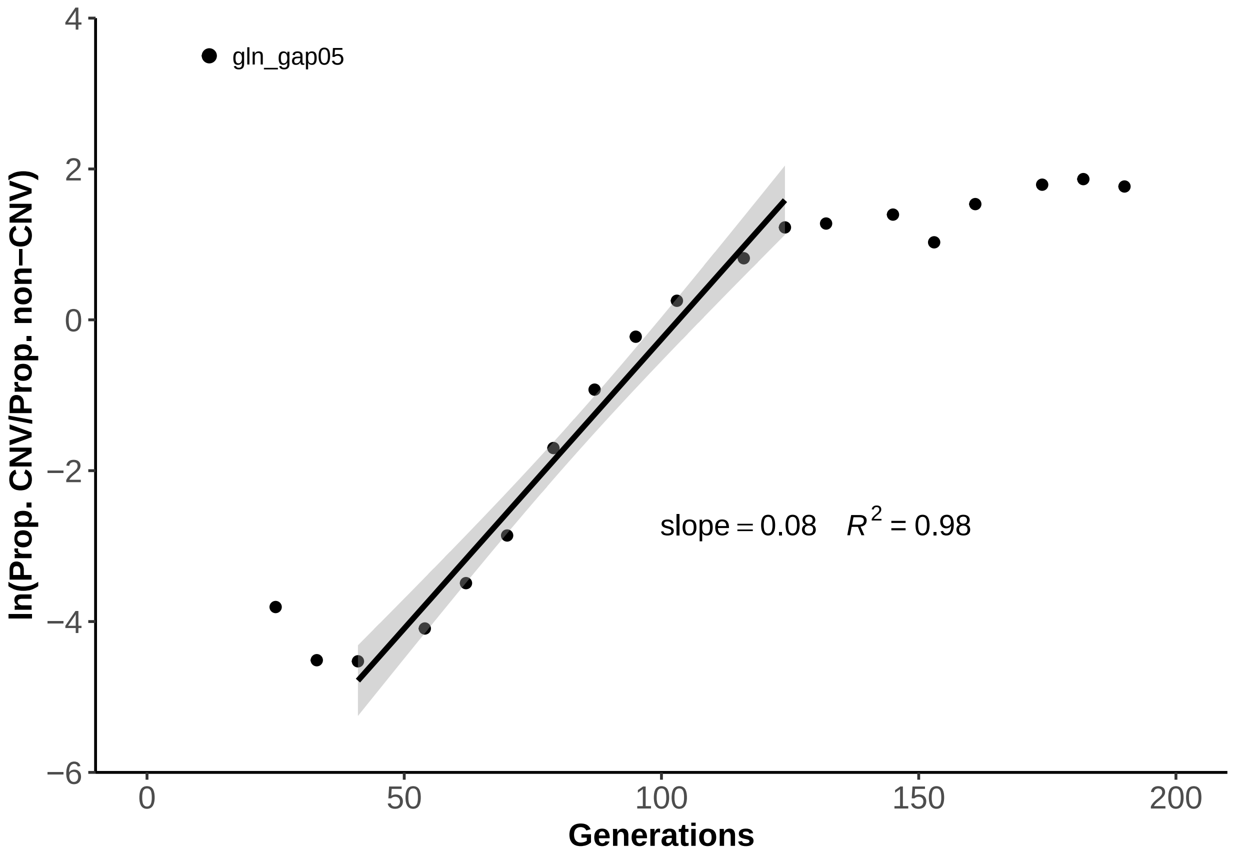


```
## [1] 0.004123018
```

```
##                   2.5 %     97.5 %
## Generation 0.06723107 0.08624646
```

```
##
## Call:
## lm(formula = logECNV_noCNV ~ Generation, data = test)
##
## Coefficients:
## (Intercept)   Generation
##    -8.02689      0.08212
```

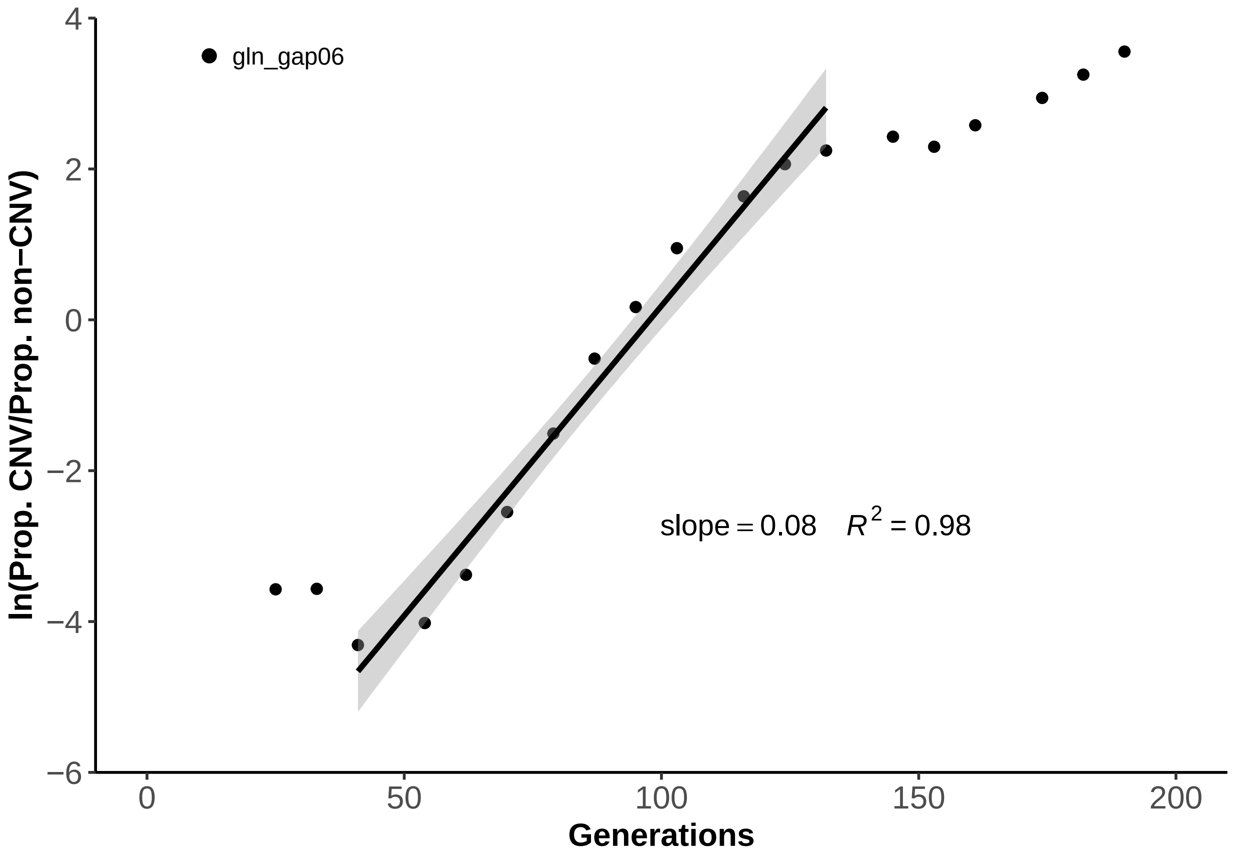


```
## [1] 0.004348782
```

```
##                   2.5 %     97.5 %
## Generation 0.07227934 0.09195459
```

```
##
## Call:
## lm(formula = logECNV_noCNV ~ Generation, data = test)
##
## Coefficients:
## (Intercept)   Generation
##    -9.70093      0.09409
```

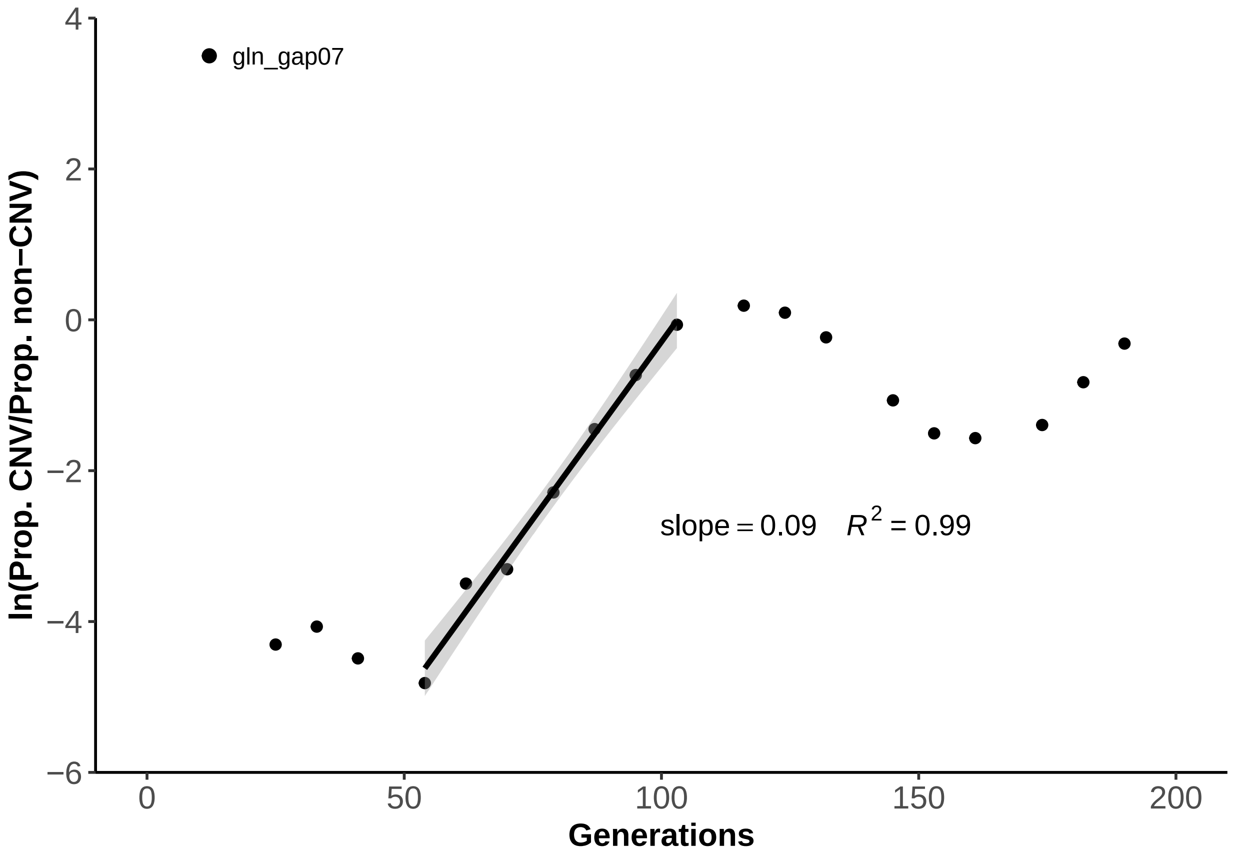


```
## [1] 0.004838433

##                  2.5 %    97.5 %
## Generation 0.08165719 0.1065324

##
## Call:
## lm(formula = logECNV_noCNV ~ Generation, data = test)
##
## Coefficients:
## (Intercept)   Generation
##    -8.89468      0.08955
```

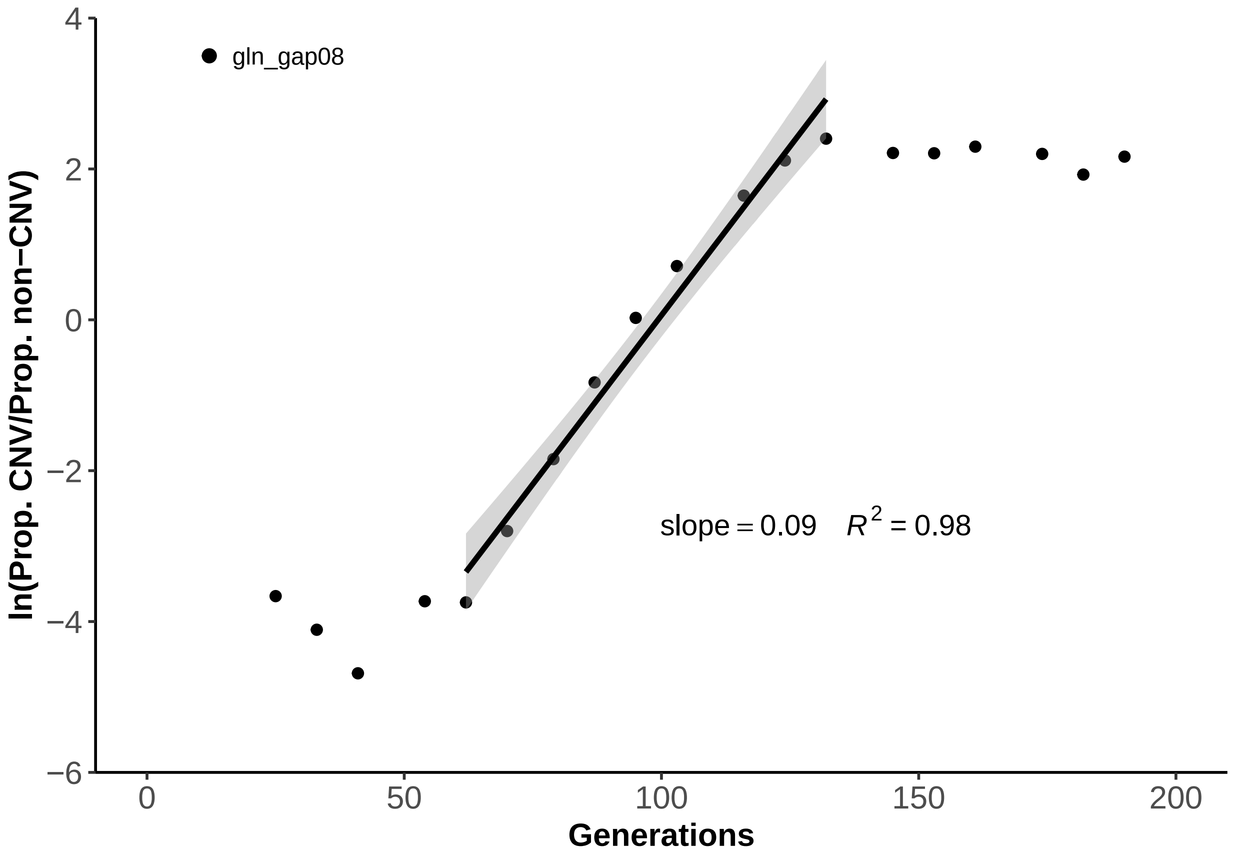


```
## [1] 0.00519789

##                 2.5 %    97.5 %
## Generation 0.07725805 0.1018402

##
## Call:
## lm(formula = logECNV_noCNV ~ Generation, data = test)
##
## Coefficients:
## (Intercept)   Generation
##    -6.94183      0.06637
```

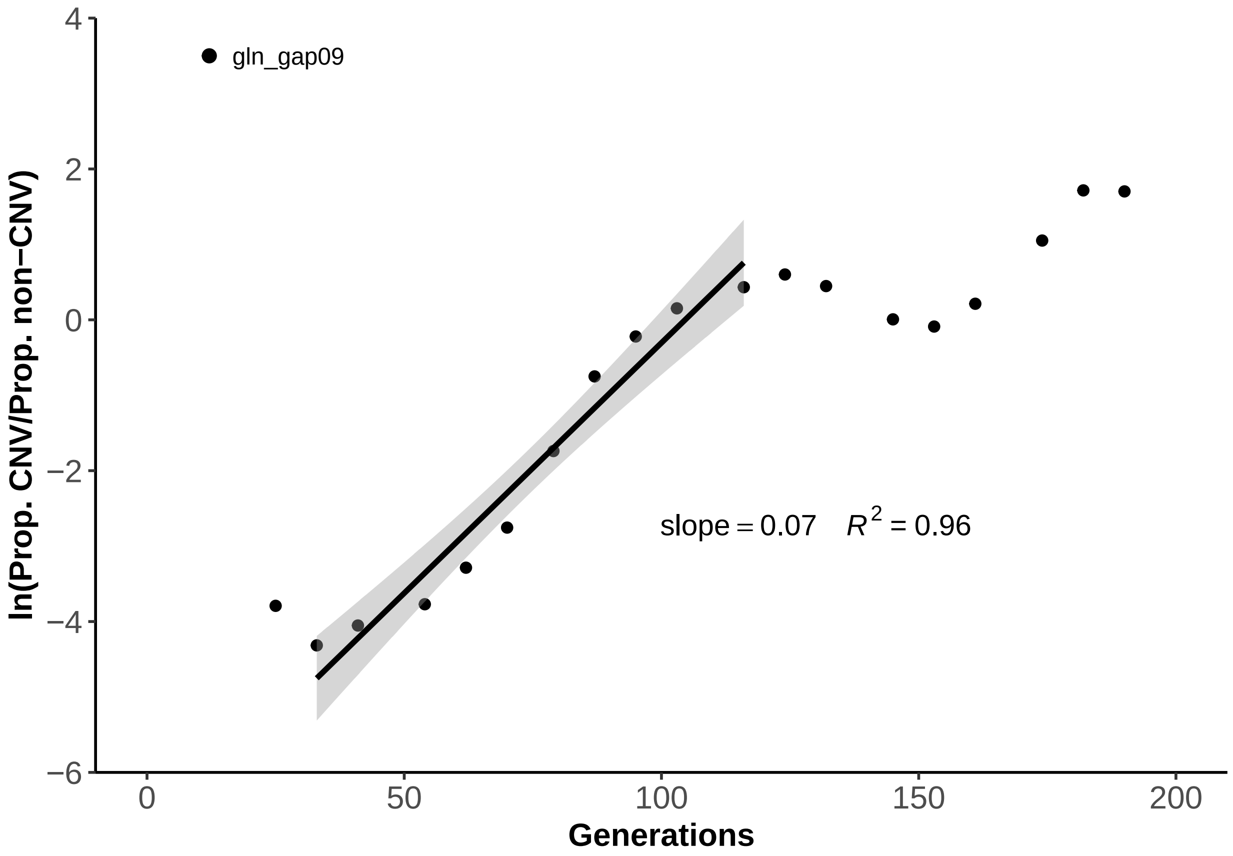


```
## [1] 0.00503076
```

```
##                  2.5 %     97.5 %
## Generation 0.05476645 0.07796836
```

2 Quantification of CNV subpopulation dynamics for barcode evolution experiment

```
##
## Call:
## lm(formula = logECNV_noCNV ~ Generation, data = test)
##
## Coefficients:
## (Intercept)   Generation
##     -8.4747       0.1242
```

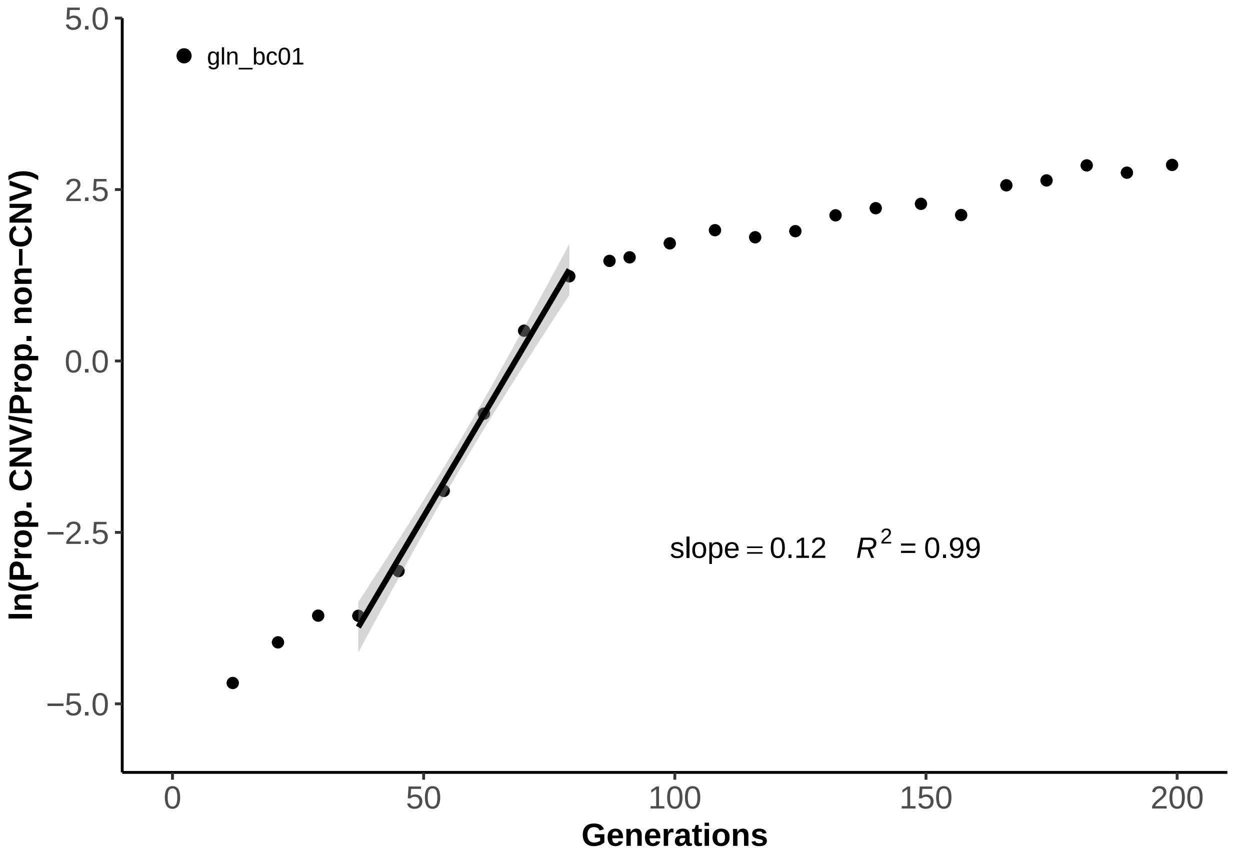


```
## [1] 0.005234006
```

```
##                  2.5 %    97.5 %
## Generation 0.1096185 0.1386823
```

```
##
## Call:
## lm(formula = logECNV_noCNV ~ Generation, data = test)
##
## Coefficients:
## (Intercept)   Generation
##    -6.24177      0.07281
```

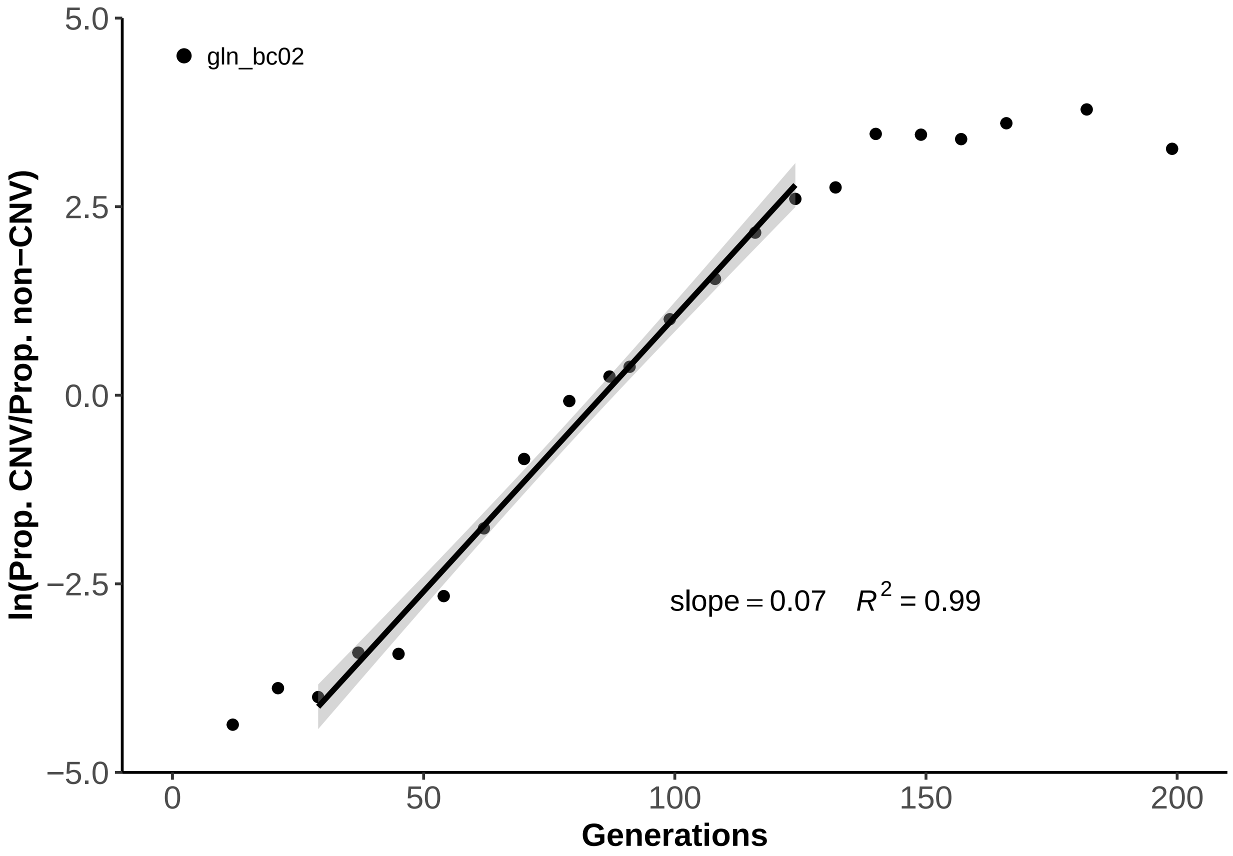


```
## [1] 0.002390899
```

```
##                  2.5 %     97.5 %
## Generation 0.06755061 0.07807527
```

```
##
## Call:
## lm(formula = logECNV_noCNV ~ Generation, data = test)
##
## Coefficients:
## (Intercept)   Generation
##    -5.22360      0.06495
```

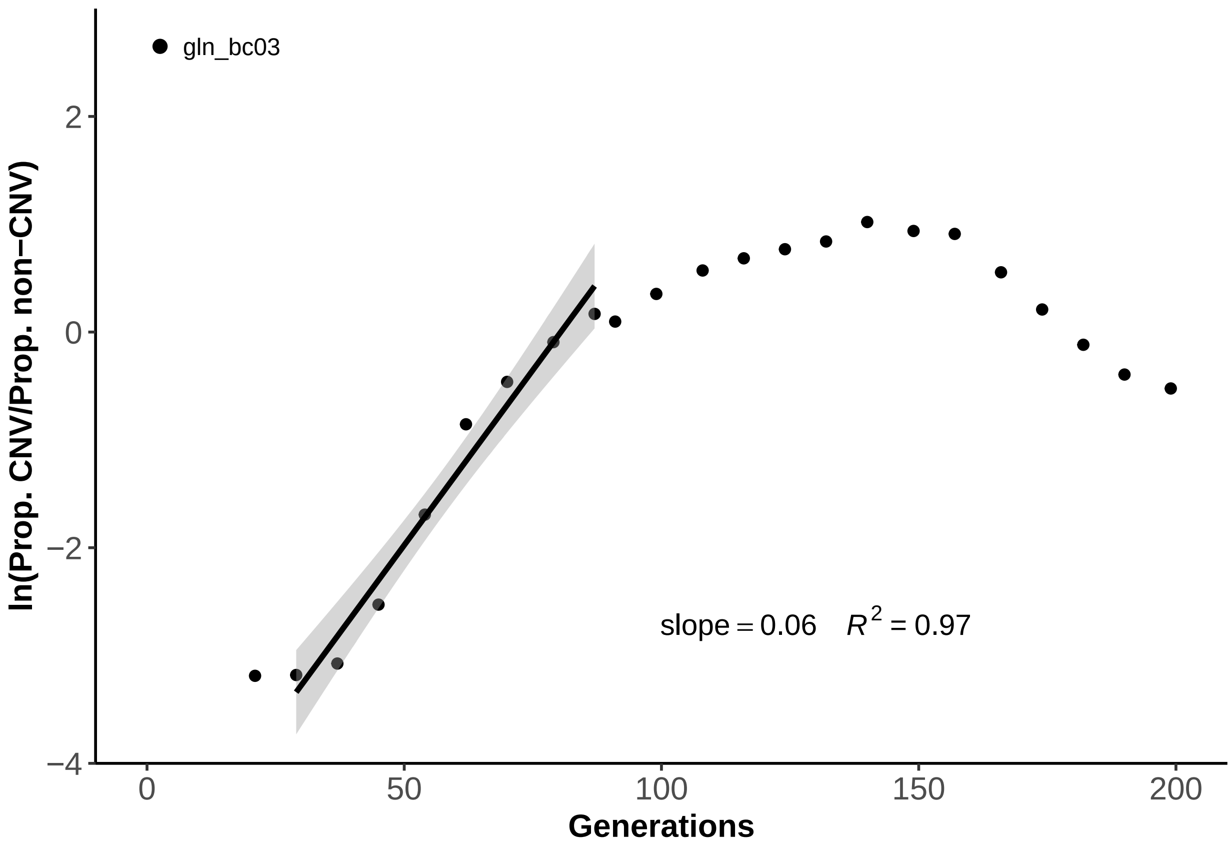


```
## [1] 0.004613785

##                  2.5 %     97.5 %
## Generation 0.05365888 0.07623793

##
## Call:
## lm(formula = logECNV_noCNV ~ Generation, data = test)
##
## Coefficients:
## (Intercept)   Generation
##     -6.2100       0.0685
```

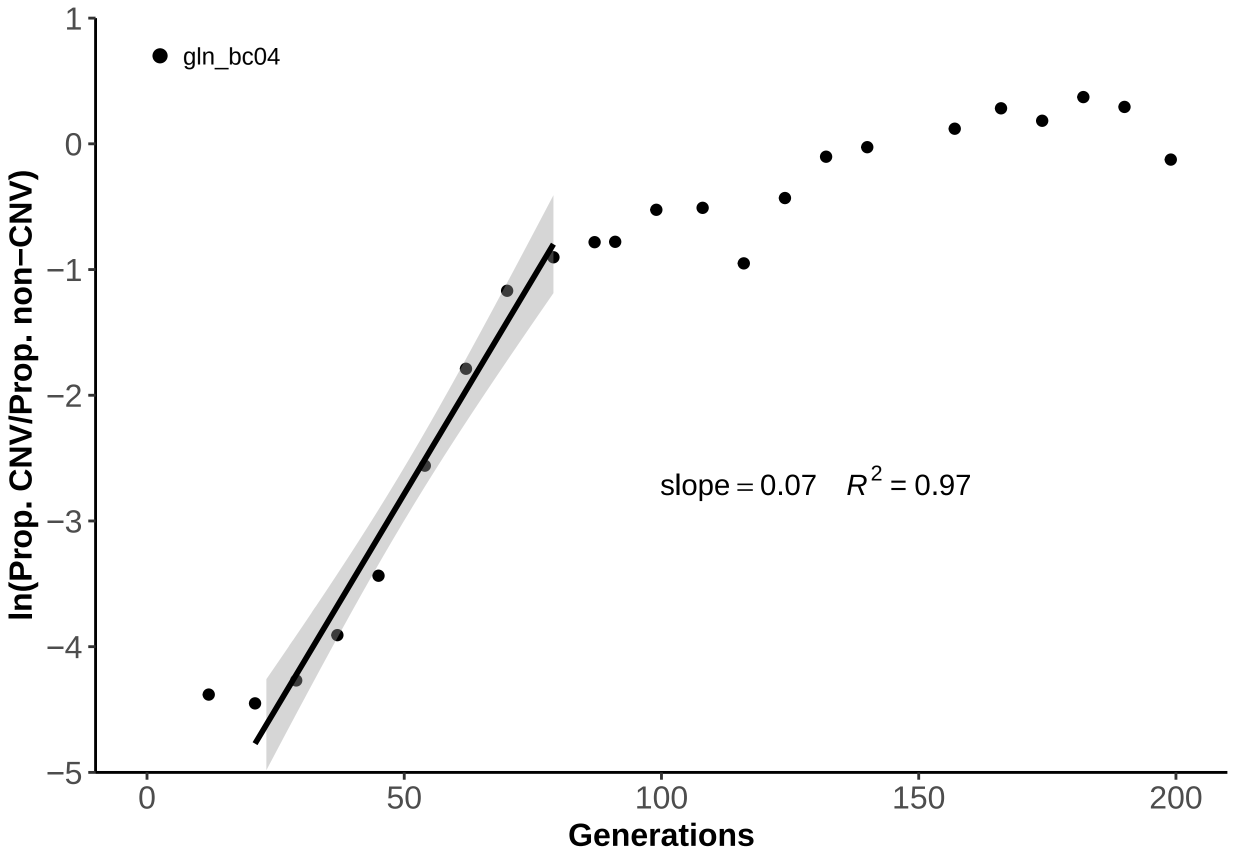


```
## [1] 0.004553106
```

```
##                  2.5 %     97.5 %
## Generation 0.05736273 0.07964483
```

```
##
## Call:
## lm(formula = logECNV_noCNV ~ Generation, data = test)
##
## Coefficients:
## (Intercept)   Generation
##    -6.41715      0.08042
```

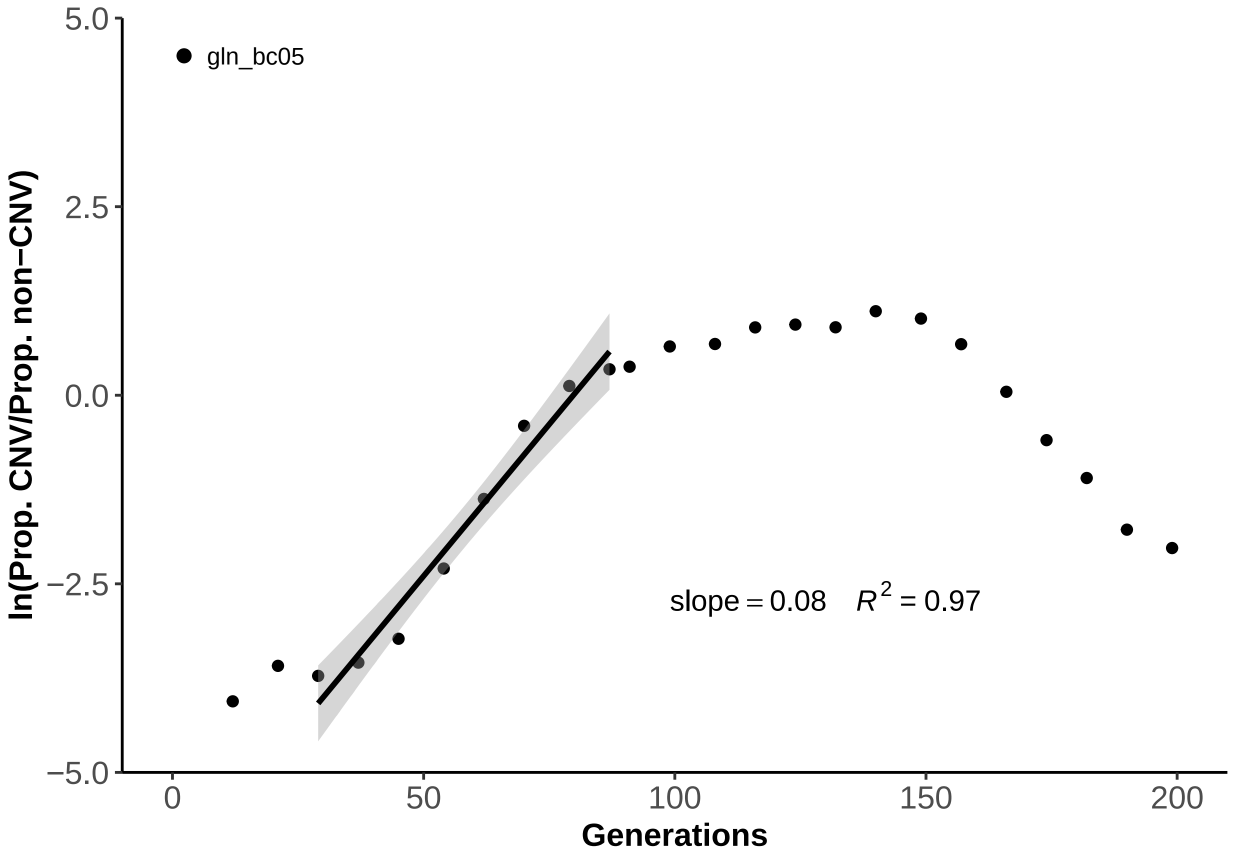


```
## [1] 0.00594899

##                 2.5 %    97.5 %
## Generation 0.0658626 0.0949759

##
## Call:
## lm(formula = logECNV_noCNV ~ Generation, data = test)
##
## Coefficients:
## (Intercept)   Generation
##     -8.0400       0.0957
```

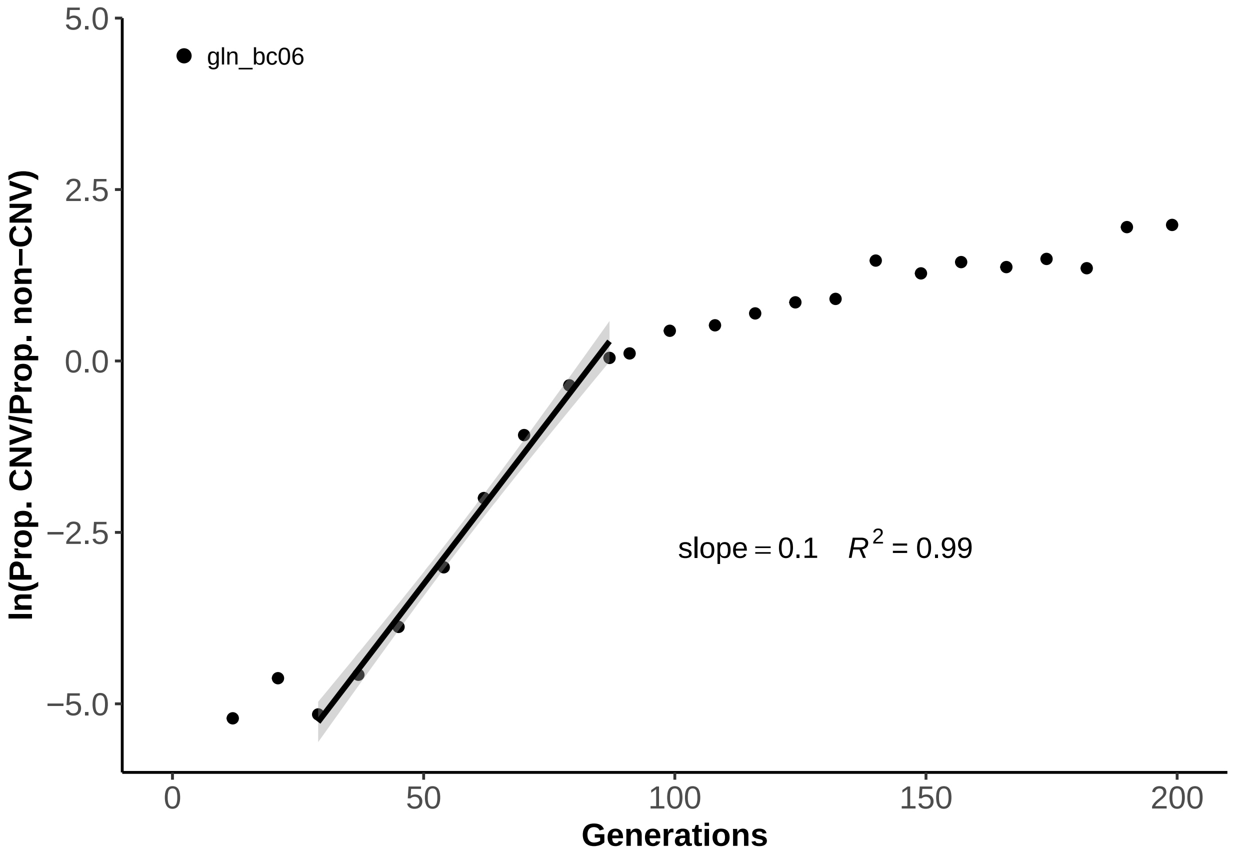


```
## [1] 0.003472746

##                  2.5 %    97.5 %
## Generation 0.08720731 0.1042023
```
