# Supplementary material for: Single-cell copy number variant detection reveals the dynamics and diversity of adaptation
Source: PLoS Biol. 2018 Dec 18;16(12):e3000069. doi: 10.1371/journal.pbio.3000069 (PMC6298651; doi:10.1371/journal.pbio.3000069)

Single-cell copy number variant detection reveals the dynamics and diversity of adaptation: S2 Text

Stephanie Lauer, Grace Avecilla, Pieter Spealman, Gunjan Sethia, Nathan Brandt, Sasha Levy and David Gresham

Compiled 2018-10-31

Contents

1 Glutamine-limited population 01 **3**

2 Glutamine-limited population 02 **9**

3 Glutamine-limited population 03 **10**

4 Glutamine-limited population 04 **15**

5 Glutamine-limited population 05 **18**

6 Glutamine-limited population 06 **21**

7 Glutamine-limited population 07 **22**

8 Glutamine-limited population 08 **25**

9 Glutamine-limited population 09 **32**

10 Glutamine-limited control populations **33**

11 Urea-limited 1 copy control population **35**

12 Urea-limited 2 copy control population **38**

13 Urea-limited population 01 **41**

14 Urea-limited population 02 **43**

15 Urea-limited population 04 **45**

16 Urea-limited population 05 **46**

17 Urea-limited population 06 **48**

18 Urea-limited population 07 **49**

19 Urea-limited population 08 **53**

The relative read depth at generation 150 and generation 250 is plotted for all glutamine-limited populations and for urea-limited populations with DUR3 CNVs. Sequenced clones from their respective populations at generation 150 or 250 are also plotted. In each case, the relative read depth across the chromosome (chromosome 11 for GAP1 and chromosome 8 for DUR3) is shown, with reads randomly downsampled for presentation purposes.

For several clones, nucleotide resolution of breakpoints was determined using a split-read approach (see methods). Each figure depicts a breakpoint assembly contig that was generated by local assembly of split reads and mapped to the reference genome. In some cases, only one breakpoint was resolved, but in other cases two breakpoint maps are shown for a single clone. These maps depict two independent events that ocurred on the left and right-hand side of either GAP1 or DUR3. Mismatches in alignment are denoted with dots. Inverted repeats are shown in red. Sequences in the reference genome (blue) are inversely oriented in the assembled contig, suggestive of an inversion.

1 Glutamine-limited population 01

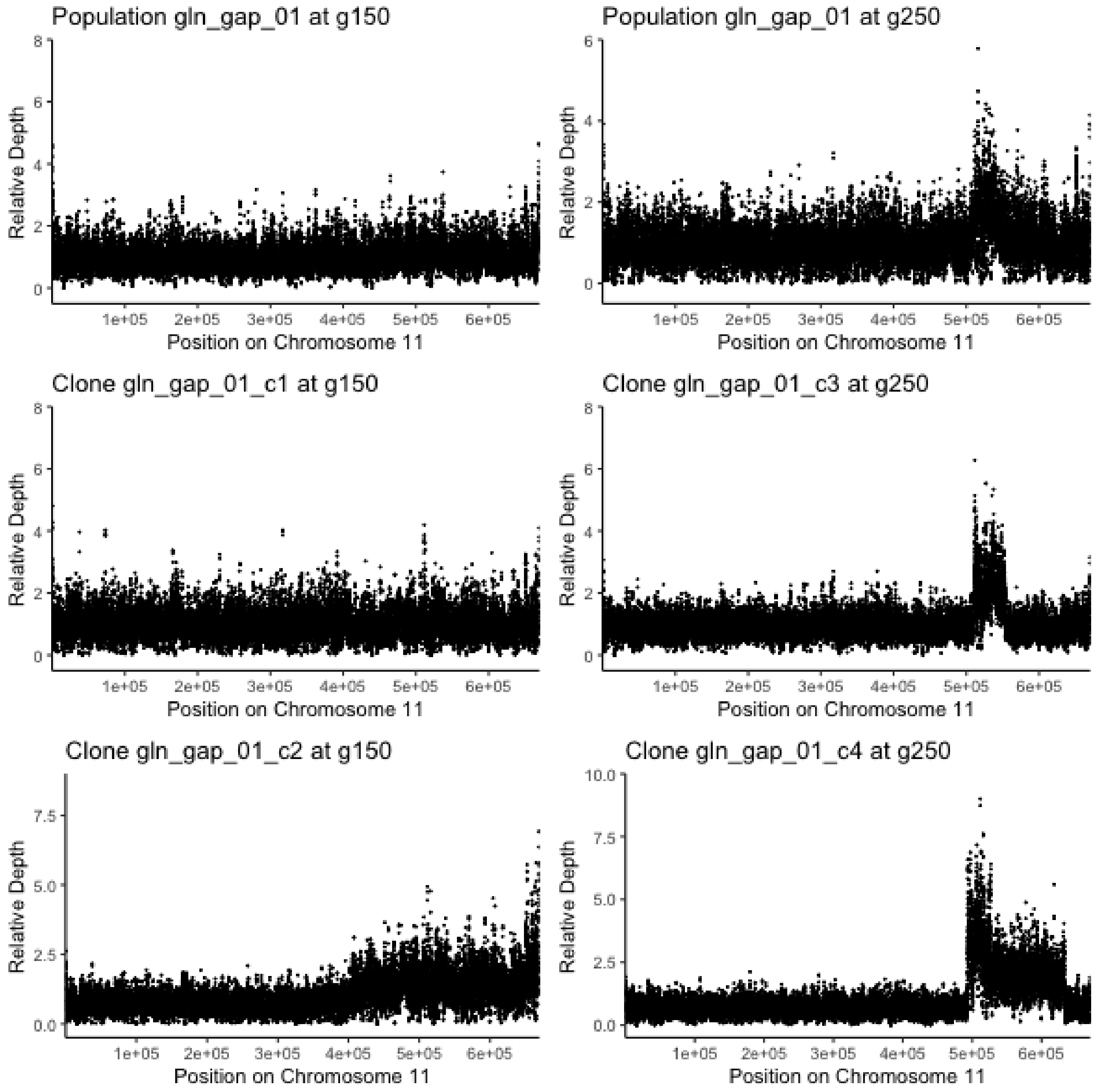

Sample: Gln_01_c2
Contig: 1 of 1

Locus: HCS1, FLO10
Supporting Split Reads: 32
Proposed Event: Segmental aneuploidy

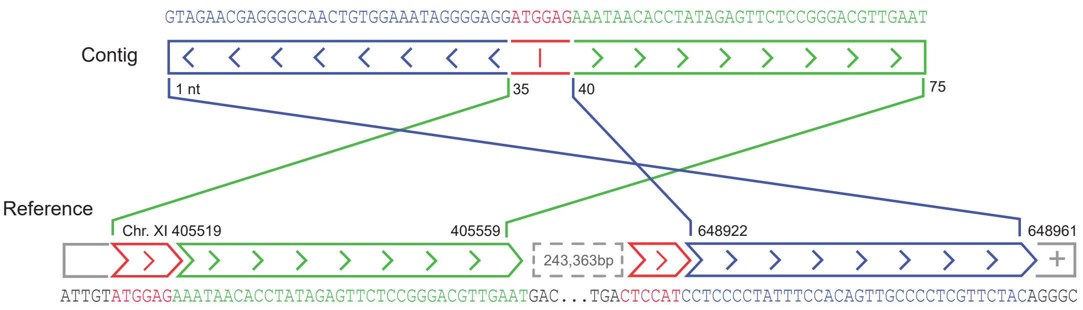

Contig

GTAGA ACGAG GGGCA ACTGT GGAAA TAGGG GAGGA TGGAG
AAATA ACACC TATAG AGTTC TCCGG GACGT TGAAT

Reference

ATTGT ATGGA GAAAT AACAC CTATA GAGTT CTCCG GGACG
TTGAA TGAC ... 243363 bp ... TGACT CCATC CTCCC
CTATT TCCAC AGTTG CCCCT CGTTC TACAG GGC

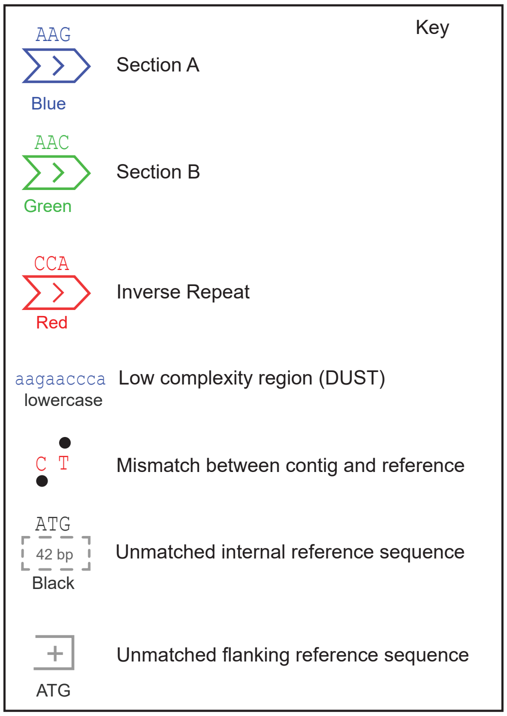

Sample: Gln_01_c3
Contig: 1 of 2

Locus: CAF4
Supporting Split Reads: 6
Proposed Event: CNV, ODIRA

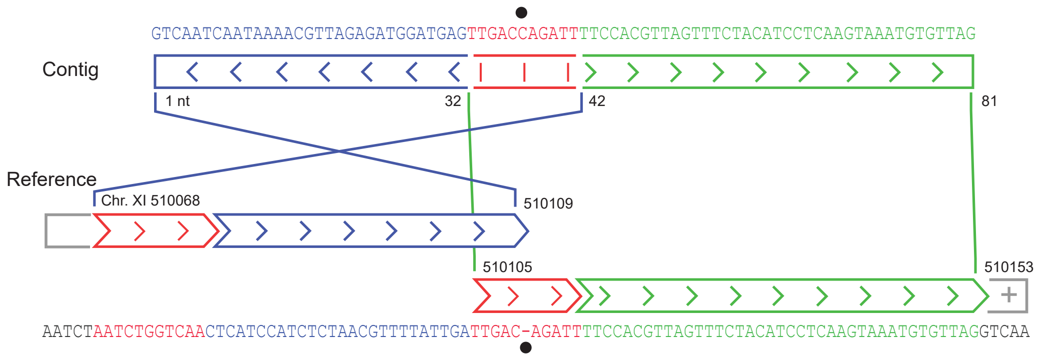

Contig

GTCAA TCAAT AAAAC GTTAG AGATG GATGA GTTGA CCAGA
TTTTC CACGT TAGTT TCTAC ATCCT CAAGT AAATG TGTTA
G

Reference

AATCT AATCT GGTCA ACTCA TCCAT CTCTA ACGTT TTATT
GATTG ACAGA TTTTC CACGT TAGTT TCTAC ATCCT CAAGT
AAATG TGTTA GGTCA A

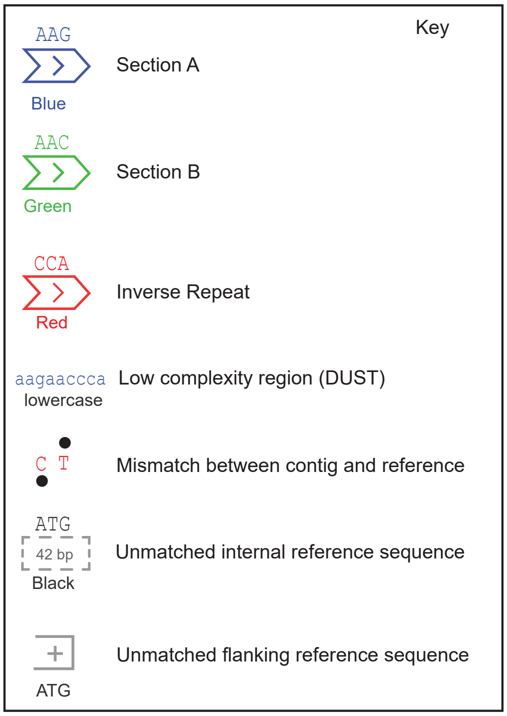

Sample: Gln_01_c3
Contig: 2 of 2

Locus: TRM2
Supporting Split Reads: 8
Proposed Event: CNV, ODIRA

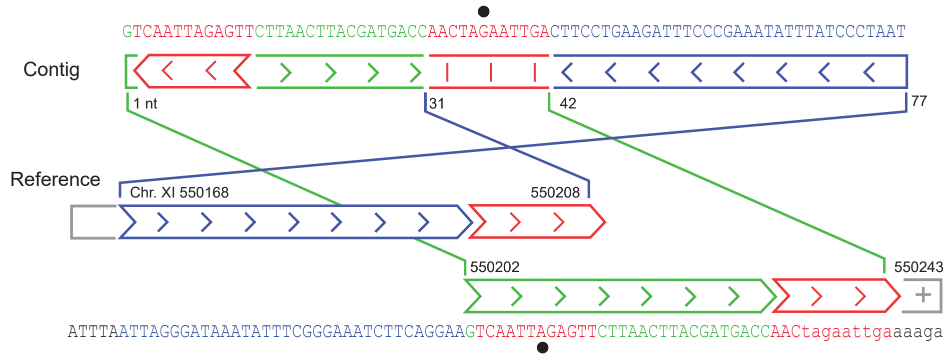

Contig
GTCAA TTAGA GTTCT TAACT TACGA TGACC AACTA GAATT
GACTT CCTGA AGATT TCCCG AAATA TTTAT CCCTA AT

Reference
ATTTA ATTAG GGATA AATAT TTCGG GAAAT CTTCA GGAAG
TCAAT TAGAG TTCTT AACTT ACGAT GACCA ACtag aattg
aaaag a

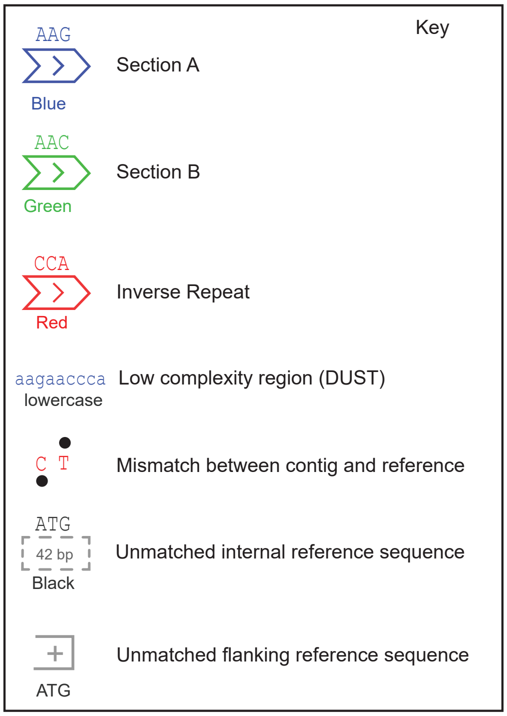

Sample: Gln_01_c4
Contig: 1 of 2

Locus: BCH2
Supporting Split Reads: 9
Proposed Event: CNV, ODIRA

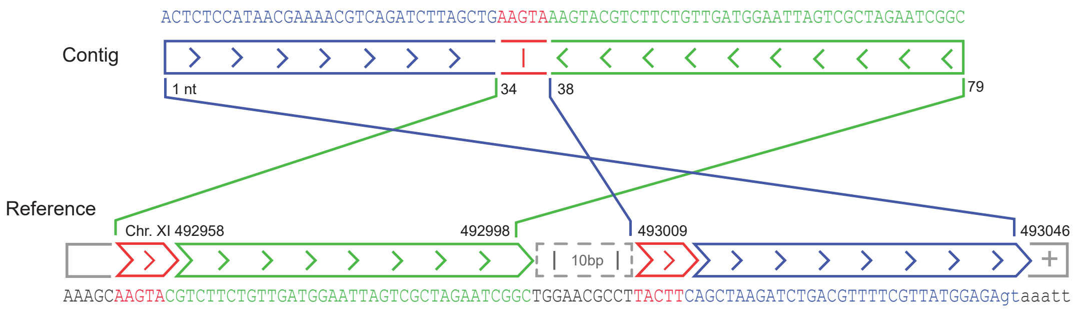

Contig

ACTCT CCATA ACGAA AACGT CAGAT CTTAG CTGAA GTAAA
GTACG TCTTC TGTTG ATGGA ATTAG TCGCT AGAAT CGGC

Reference

AAAGC AAGTA CGTCT TCTGT TGATG GAATT AGTCG CTAGA
ATCGG CTGGA ACGCC TTACT TCAGC TAAGA TCTGA CGTTT
TCGTT ATGGA GAgta aatt

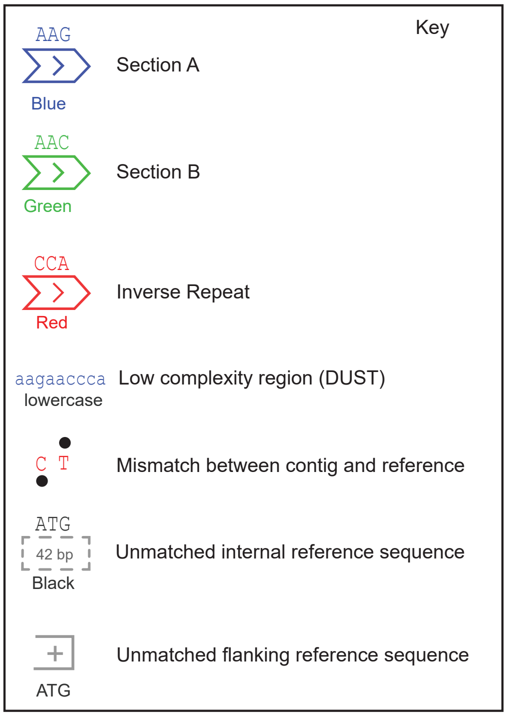

Sample: Gln_01_c4
Contig: 2 of 2

Locus: ESL2
Supporting Split Reads: 4
Proposed Event: CNV, ODIRA

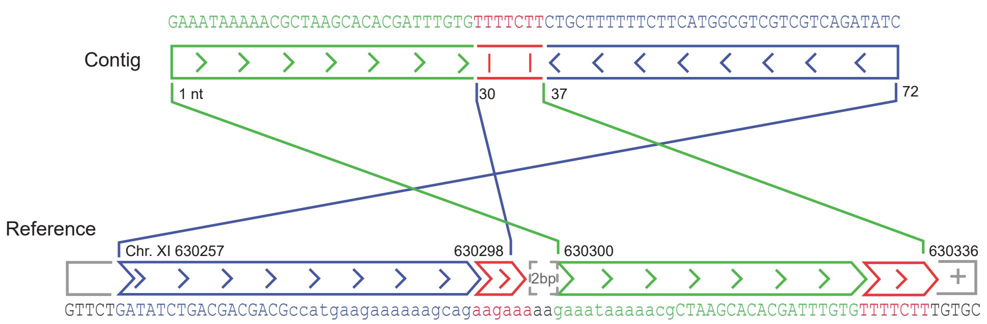

Contig
GAAAT AAAAA CGCTA AGCAC ACGAT TTGTG TTTTC TTCTG
CTTTT TTCTT CATGG CGTCG TCGTC AGATA TC

Reference
GTTCT GATAT CTGAC GACGA CGcca tgaag aaaaa agcag
aagaa aaaga aataa aaacg CTAAG CACAC GATTT GTGTT
TTCTT TGTGC

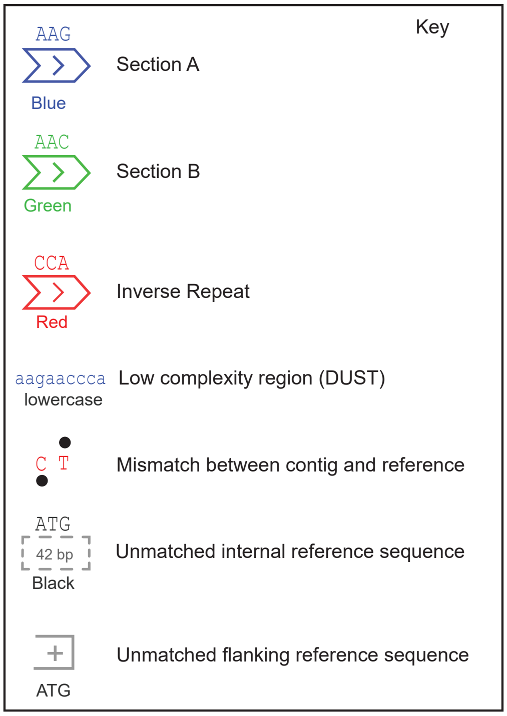

2 Glutamine-limited population 02

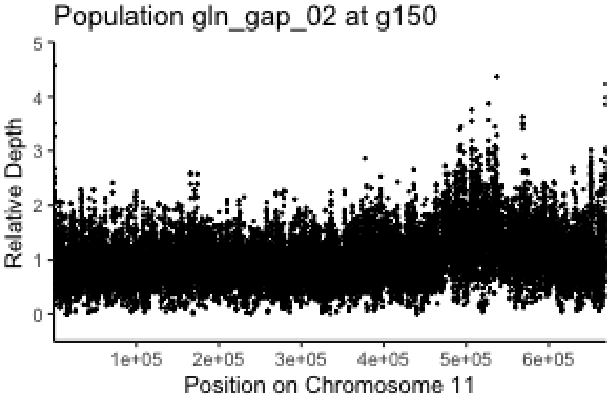

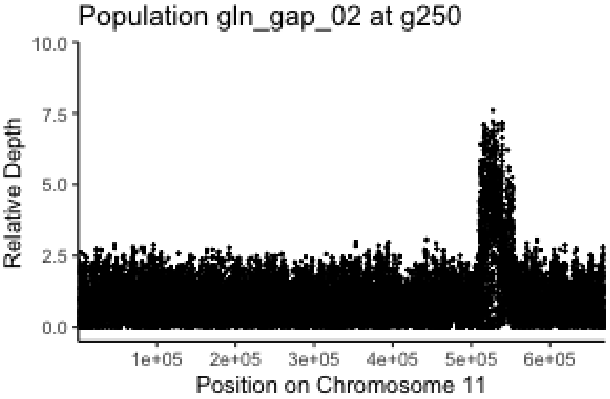

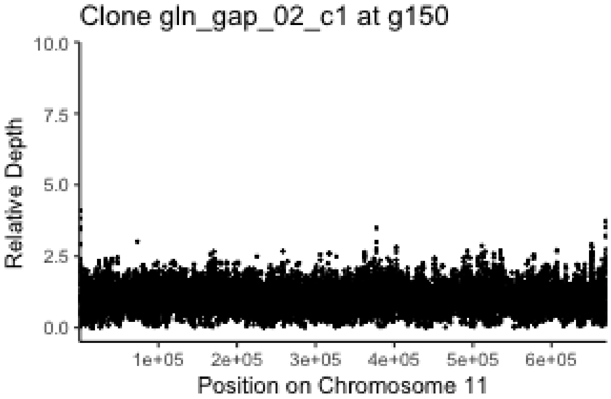

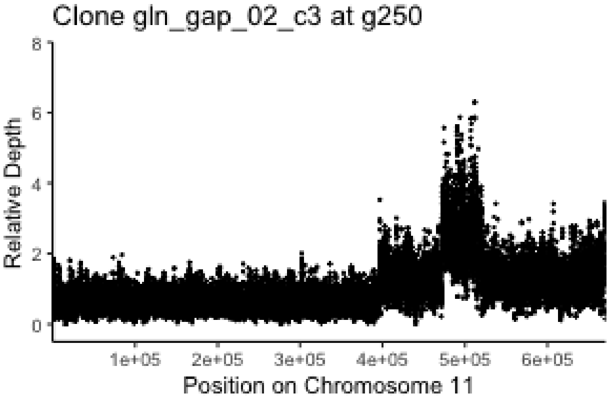

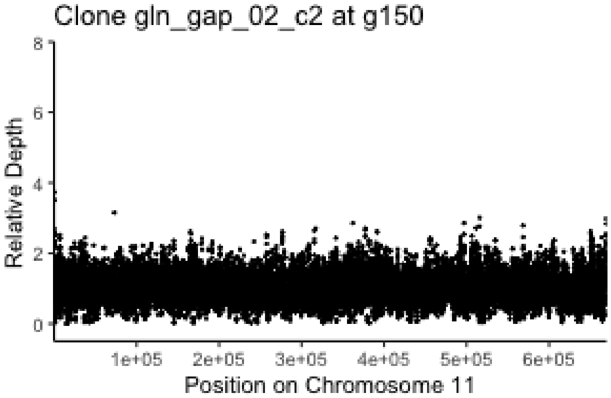

3 Glutamine-limited population 03

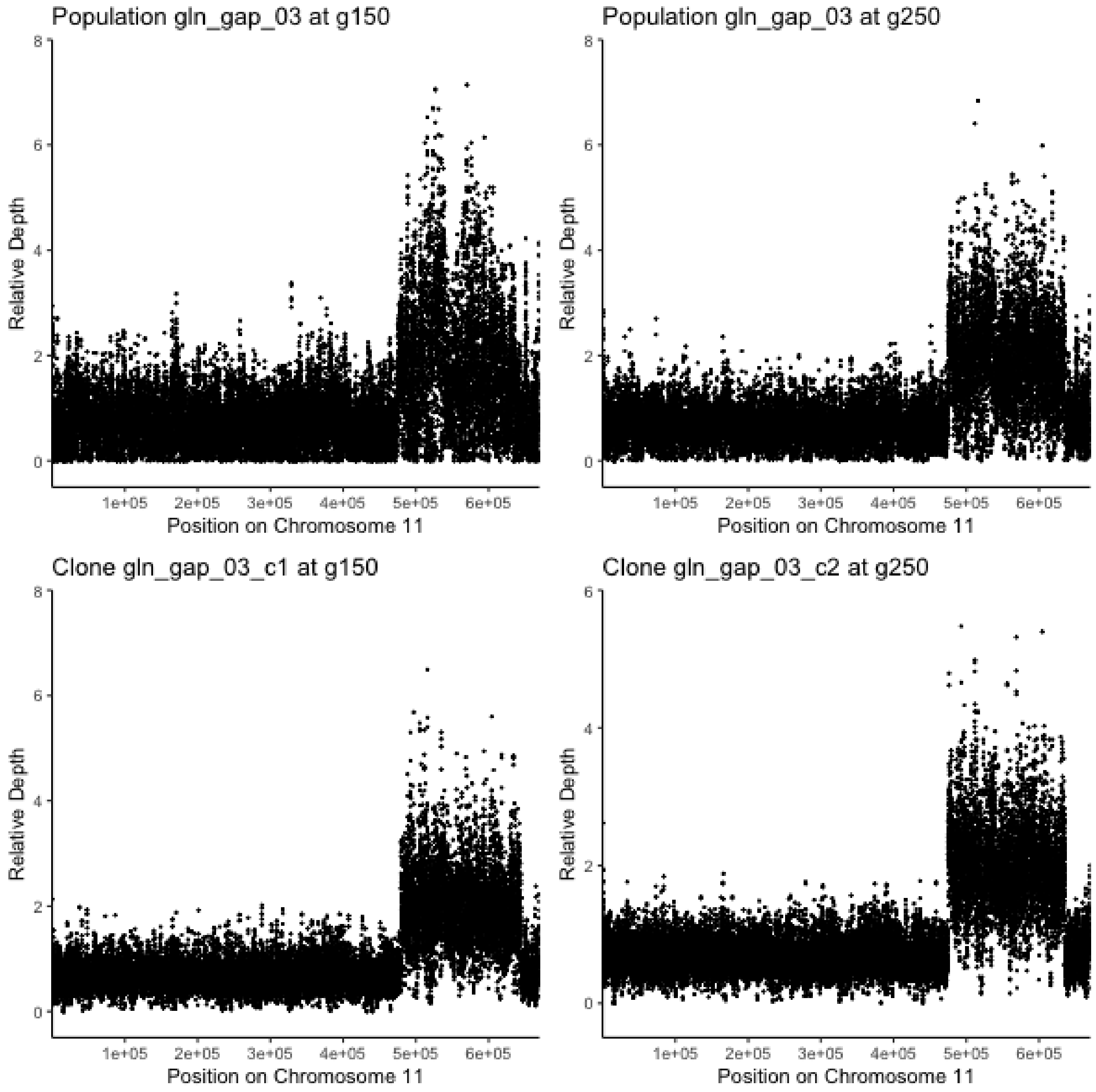

Sample: Gln_03_c1
Contig: 1 of 2

Locus: ALY1
Supporting Split Reads: 8
Proposed Event: CNV, ODIRA

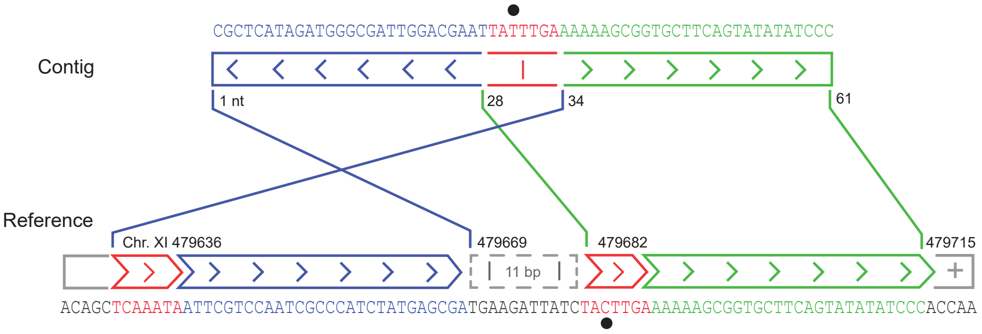

Contig
CGCTC ATAGA TGGGC GATTG GACGA ATTAT TTGAA AAAAG
CGGTG CTTCA GTATA TATCC C

Reference
ACAGC TCAAA TAATT CGTCC AATCG CCCAT CTATG AGCGA
TGAAG ATTAT CTACT TGAAA AAAGC GGTGC TTCAG TATAT
ATCCC ACCAA

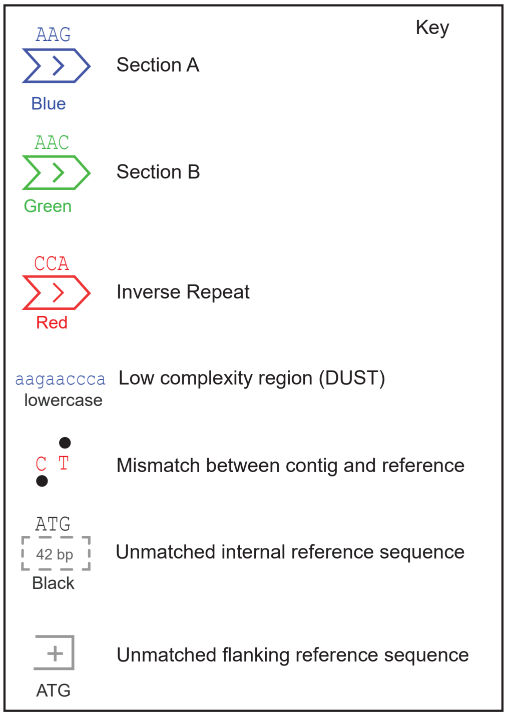

Sample: Gln_03_c1
Contig: 2 of 2

Locus: SIR1
Supporting Split Reads: 10
Proposed Event: CNV, ODIRA

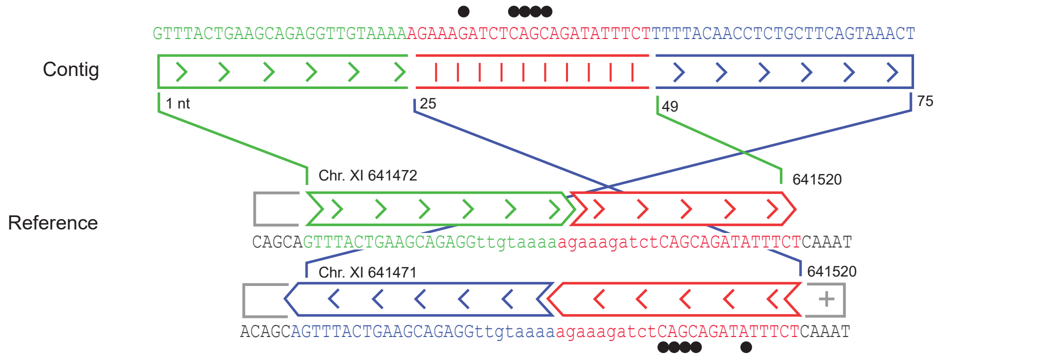

Contig

GTTTA CTGAA GCAGA GGTTG TAAAA AGAAA GATCT CAGCA
GATAT TTCTT TTTAC AACCT CTGCT TCAGT AAACT

Reference

ACAGC AGTTT ACTGA AGCAG AGGtt gtaaa aagaa agatc
tCAGC AGATA TTTCT CAAAT

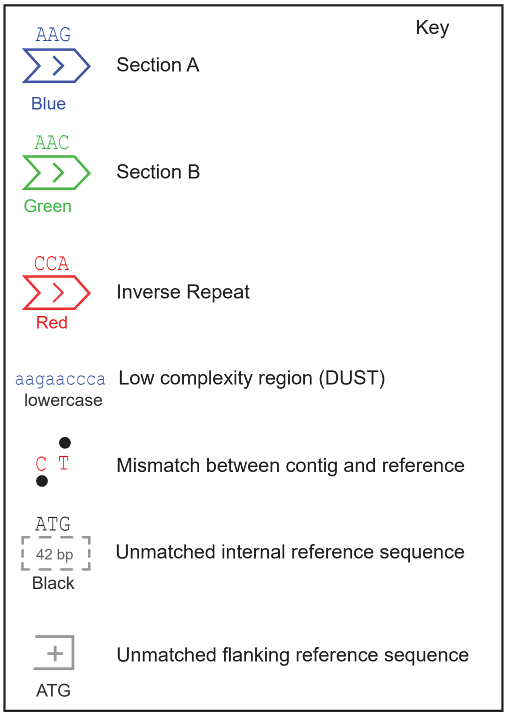

Sample: Gln_03_c2
Contig: 1 of 2

Locus: YKR018C
Supporting Split Reads: 11
Proposed Event: CNV, ODIRA

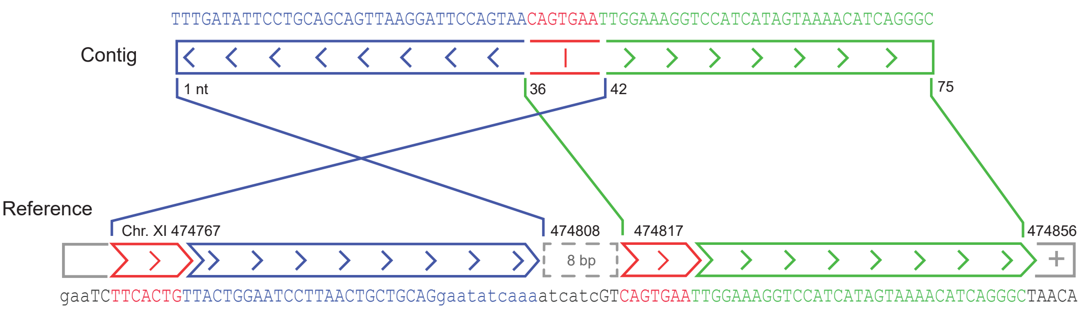

Contig

TTTGA TATTC CTGCA GCAGT TAAGG ATTCC AGTAA CAGTG
AATTG GAAAG GTCCA TCATA GTAAA ACATC AGGGC

Reference

gaaTC TTCAC TGTTA CTGGA ATCCT TAACT GCTGC AGgaa
tatca aaatc atcGT CAGTG AATTG GAAAG GTCCA TCATA
GTAAA ACATC AGGGC TAACA

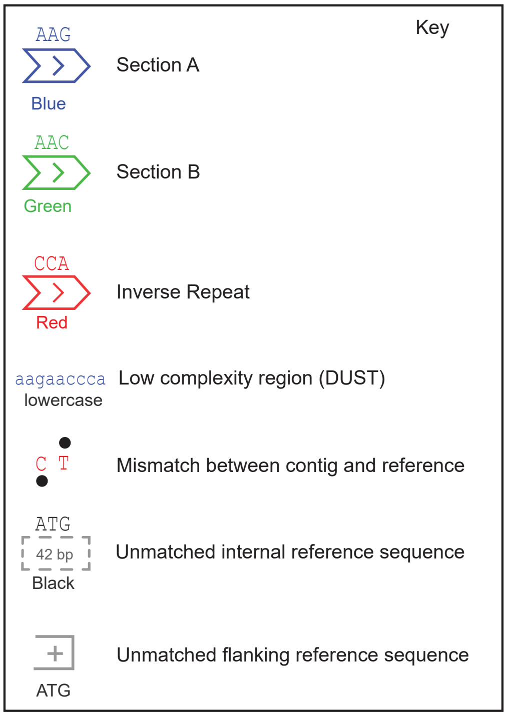

Sample: Gln_03_c2
Contig: 2 of 2

Locus: PCK1
Supporting Split Reads: 23
Proposed Event: CNV, ODIRA

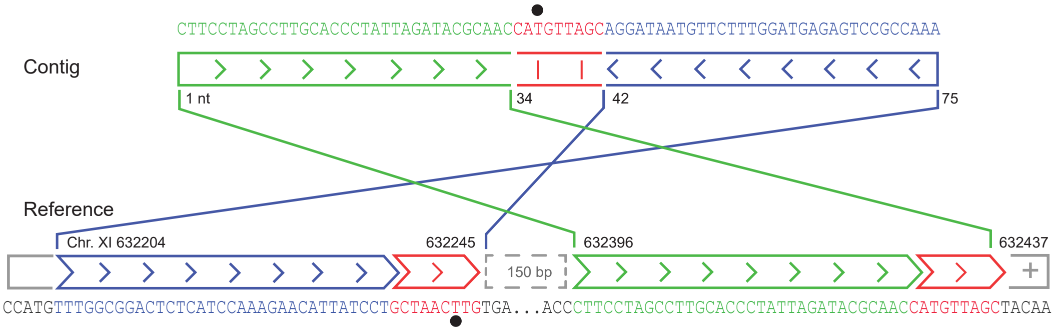

Contig

AATAA ACTCT TCCAA CTCTG ttttt ttCAA AGCTA GTGGT
GCATT TACAC GGTCA GCTTT ATGAT ACCGC TATTG

Reference

CCATG TTTGG CGGAC TCTCA TCCAA AGAAC ATTAT CCTGC
TAACT TGTGA TGCTT CGGGT GTTTT ACCAC CAGTA TCTAA
ATTGA CTCCT GAACA AGTCA TGTAC CATTT CATCT CTGGT
TACAC TTCTA AAATG GCTGG TACTG AGCAA GGTGT CACTG
AACCT GAACc aacat tttca tcttg TTTCG GACAA CCCTT
CCTAG CCTTG CACCC TATTA GATAC GCAAC CATGT TAGCT
ACAA

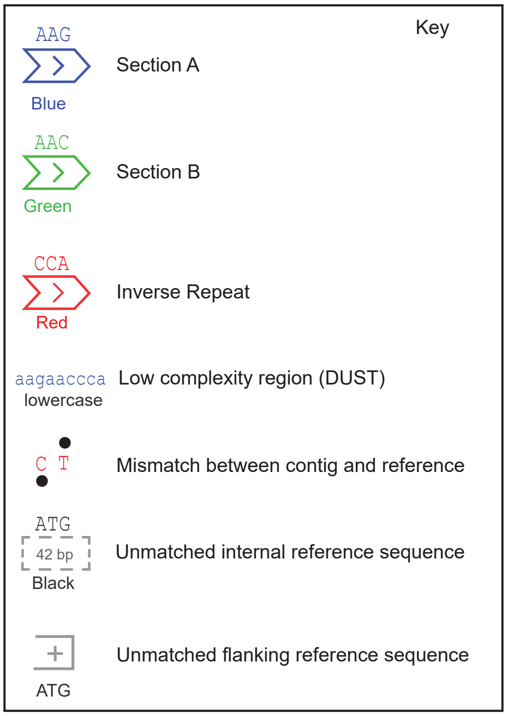

4 Glutamine-limited population 04

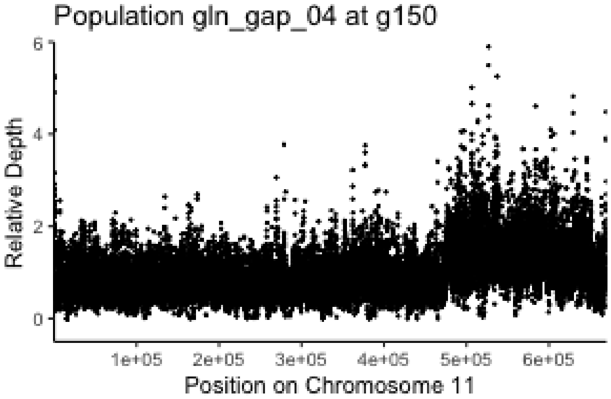

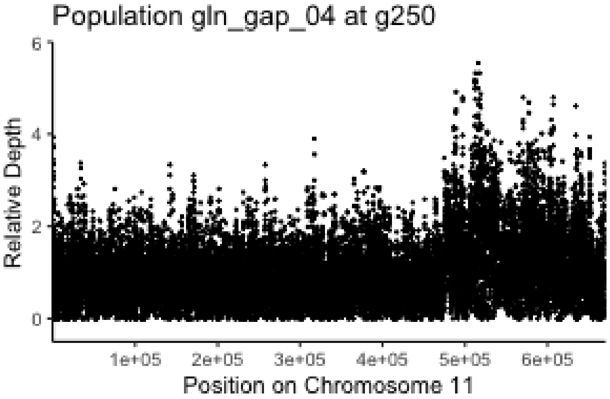

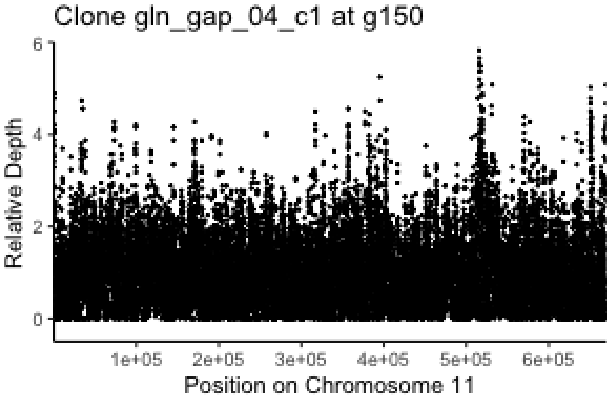

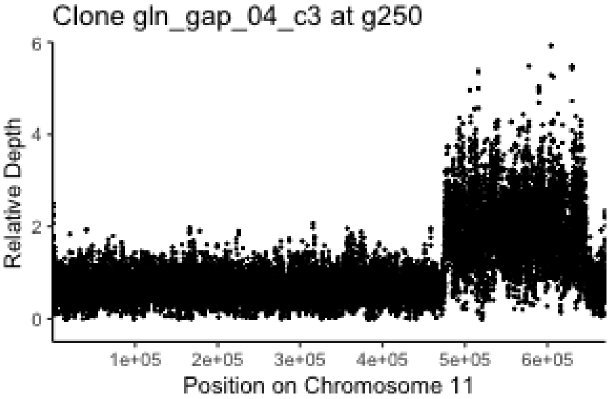

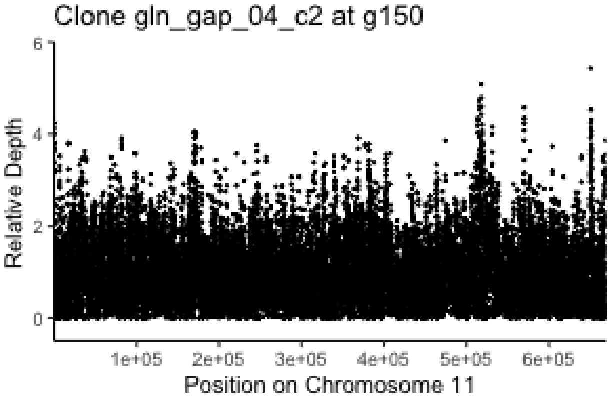

Sample: Gln_04_c3
Contig: 1 of 2

Locus: YKR018C
Supporting Split Reads: 6
Proposed Event: CNV, ODIRA

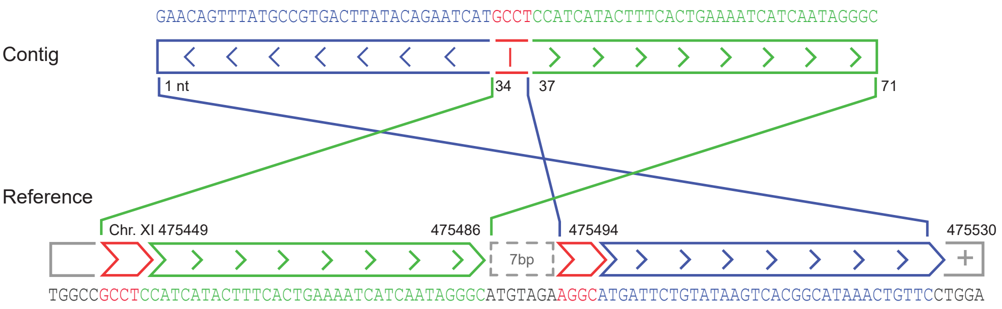

Contig
GAACA GTTTA TGCCG TGACT TATAC AGAAT CATGC CTCCA
TCATA CTTTC ACTGA AAATC ATCAA TAGGG C

Reference
TGGCC GCCTC CATCA TACTT TCACT GAAAA TCATC AATGG
GCATG TAGAA GGCAT GATTC TGTAT AAGTC ACGGC ATAAA
CTGTT CCTGG A

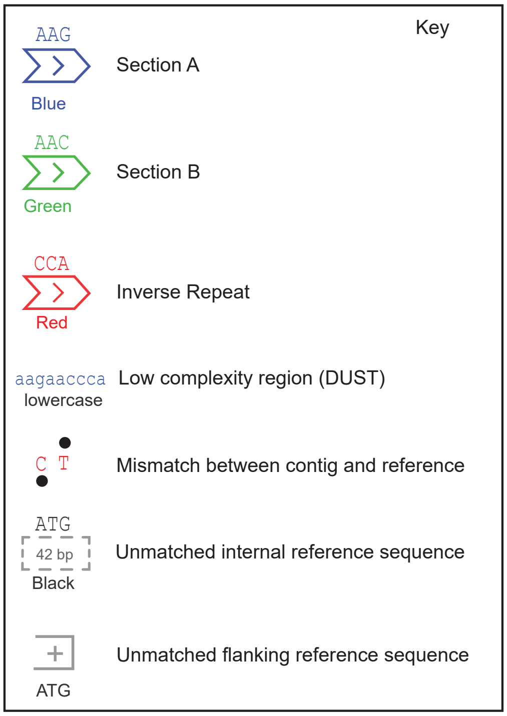

Sample: Gln_04_c3
Contig: 2 of 2

Locus: Intergenic SIR1, FLO10
Supporting Split Reads: 5
Proposed Event: CNV, ODIRA

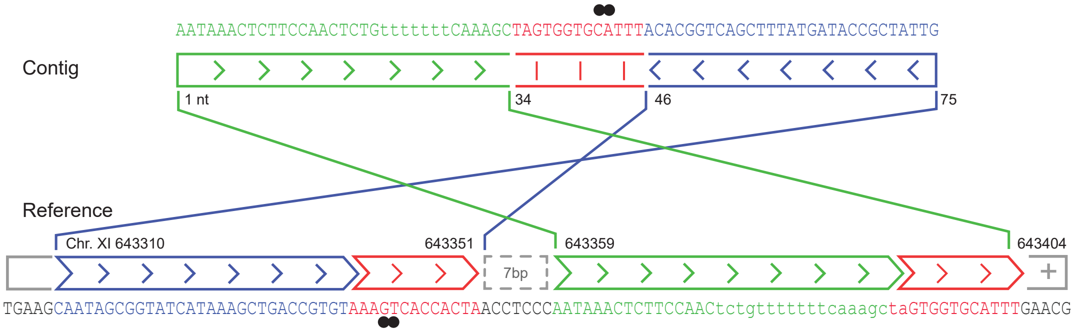

Contig

AATAA ACTCT TCCAA CTCTG ttttt ttCAA AGCTA GTGGT
GCATT TACAC GGTCA GCTTT ATGAT ACCGC TATTG

Reference

TGAAG CAATA GCGGT ATCAT AAAGC TGACC GTGTA AAGTC
ACCAC TAACC TCCCA ATAAA CTCTT CCAAC tctgt ttttt
caaag ctaGT GGTGC ATTTG AACG

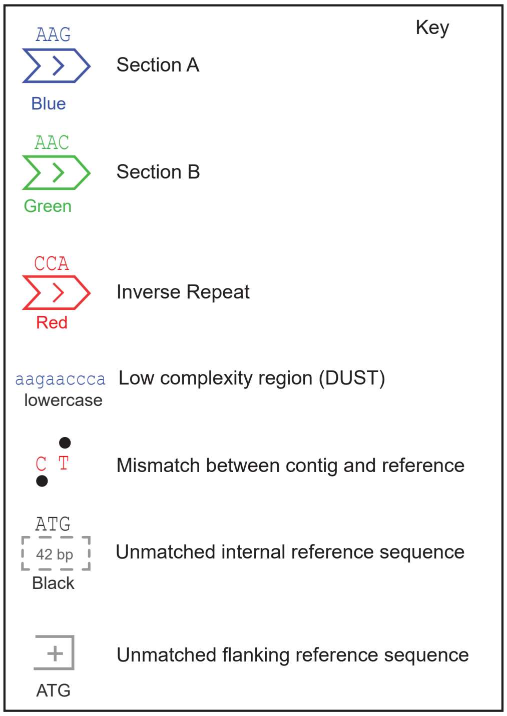

5 Glutamine-limited population 05

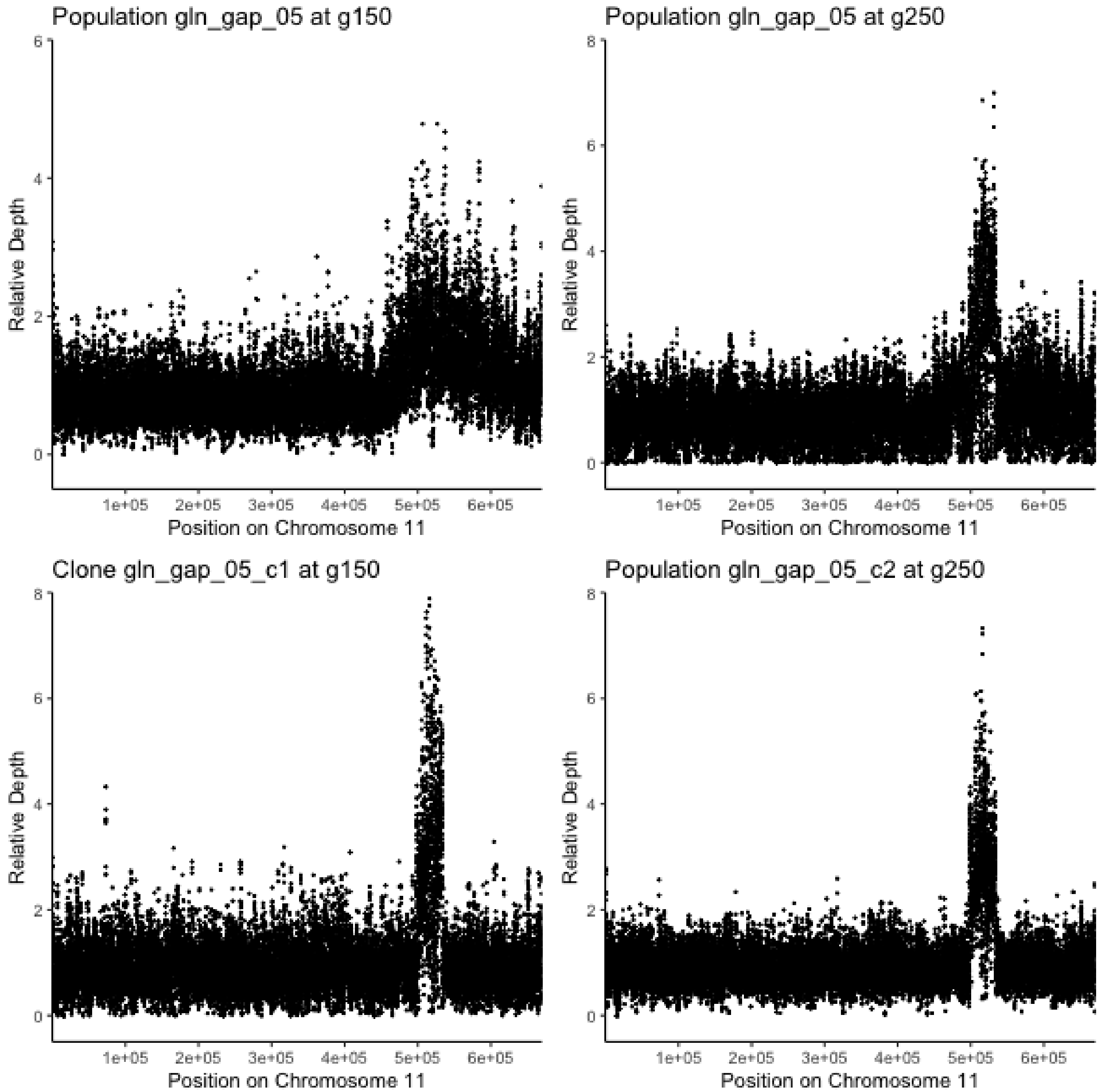

Sample: Gln_05_c2
Contig: 1 of 2

Locus: SET3
Supporting Split Reads: 10
Proposed Event: Unresolved

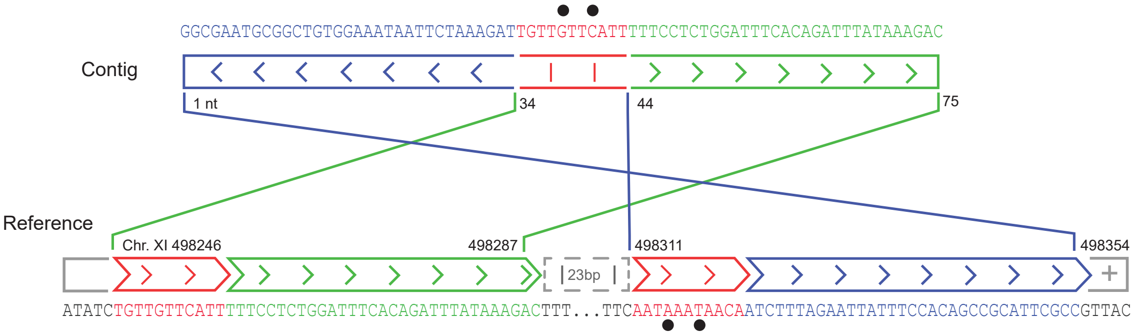

Contig
GGCGA ATGCG GCTGT GGAAA TAATT CTAAA GATTG TTGTT
CATTT TTCCT CTGGA TTTCA CAGAT TTATA AAGAC

Reference
ATATC TGTTG TTCAT TTTTC CTCTG GATTT CACAG ATTTA
TAAAG ACTTT GCGCG TATCT CTTCA CTTTC AATAA ATAAC
AATCT TTAGA ATTAT TTCCA CAGCC GCATT CGCCG TTAC

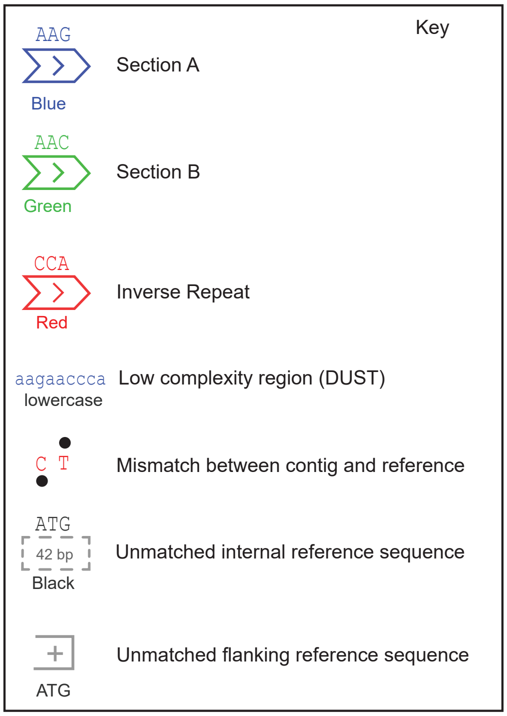

Sample: Gln_05_c2
Contig: 2 of 2

Locus: Intergenic TRK2, YKR051W
Supporting Split Reads: 8
Proposed Event: Unresolved

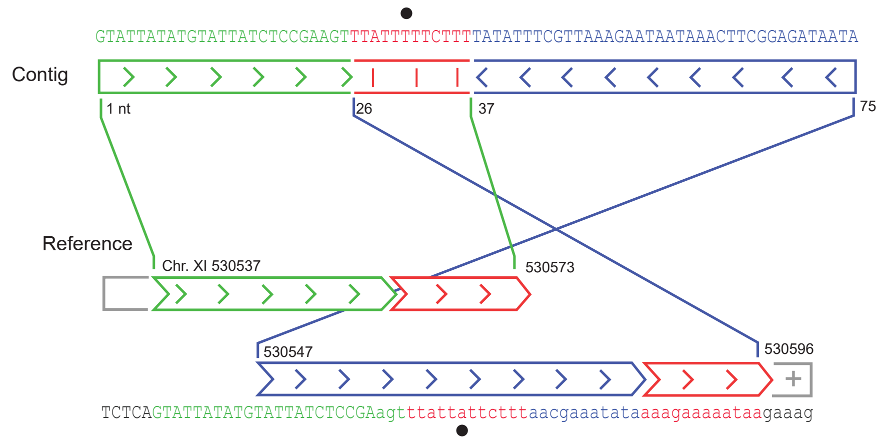

Contig
GTATT ATATG TATTA TCTCC GAAGT TTATT TTTCT TTTAT
ATTTC GTTAA AGAAT AATAA ACTTC GGAGA TAATA

Reference
TCTCA GTATT ATATG TATTA TCTCC GAagt ttatt attct
ttaac gaaat ataaa agaaa aataa gaaag

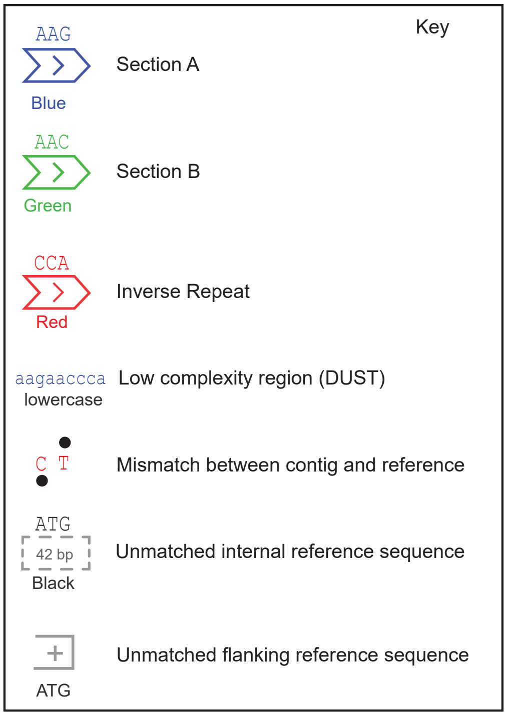

6 Glutamine-limited population 06

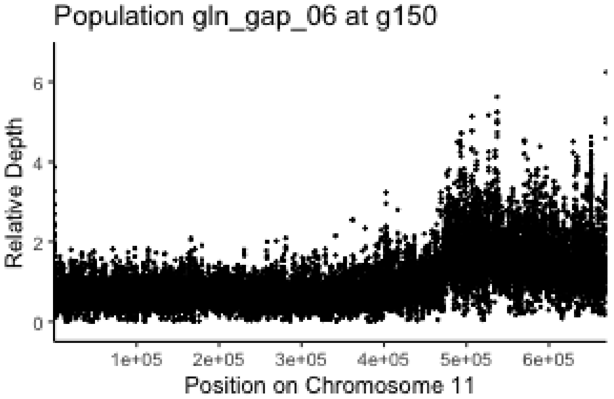

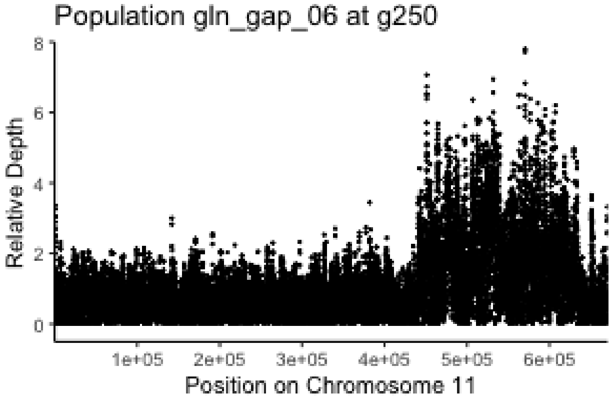

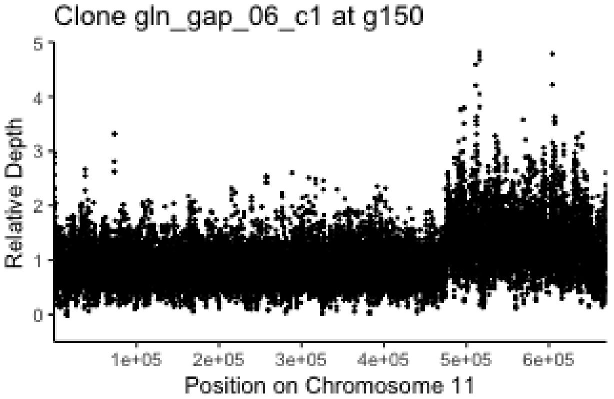

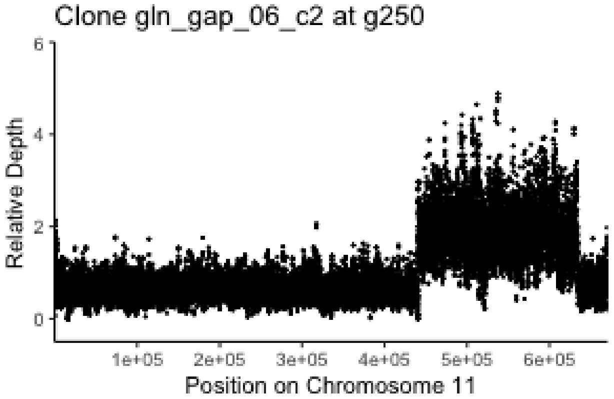

7 Glutamine-limited population 07

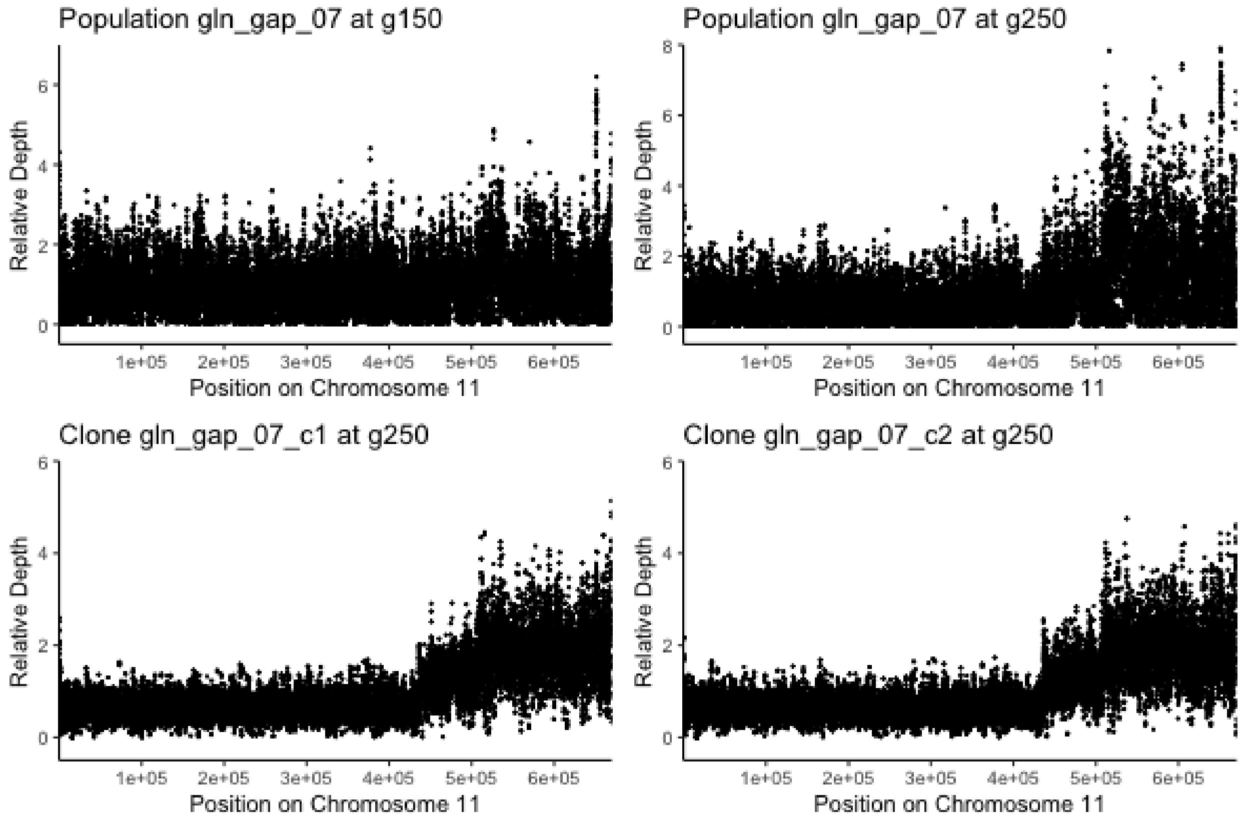

Sample: Gln_07_c1
Contig: 1 of 1

Locus: DID1, AUR1
Supporting Split Reads: 14
Proposed Event: Segmental Aneuploid

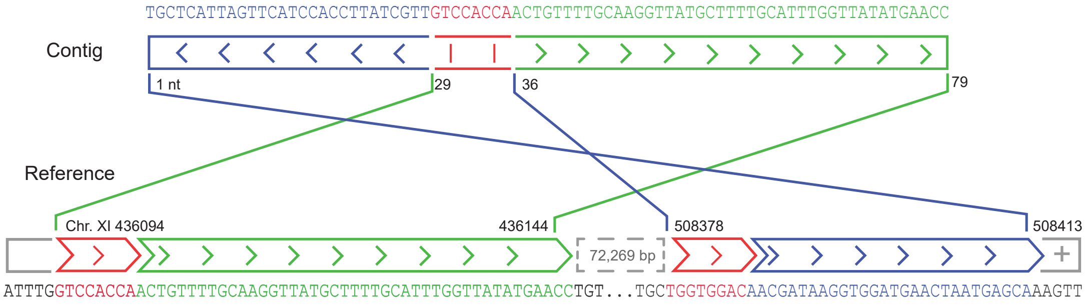

Contig

TGCTC ATTAG TTCAT CCACC TTATC GTTGT CCACC AACTG
TTTTG CAAGG TTATG CTTTT GCATT TGGTT ATATG AACC

Reference Chr. XI 436094 - 436144

ATTTG GTCCA CCAAC TGTTT TGCAA GGTTA TGCTT TTGCA
TTTGG TTATA TGAAC CTGTT T

Reference Chr. XI 508378 - 508413

ATTGC TGGTG GACAA CGATA AGGTG GATGA ACTAA TGAGC
AAAGT T

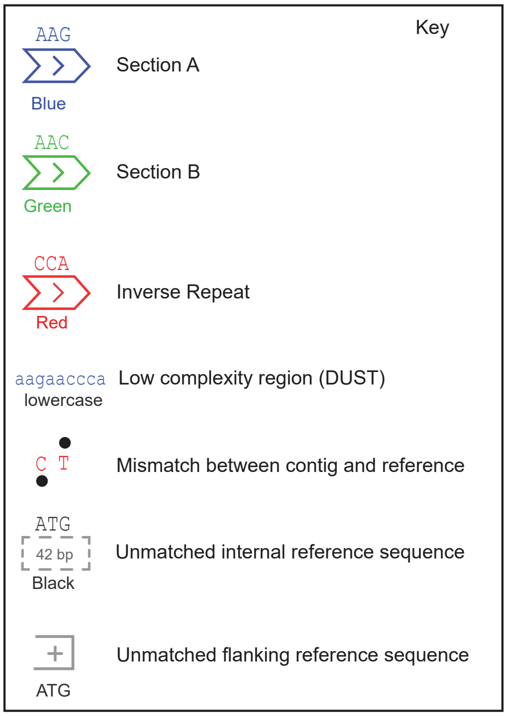

Sample: Gln_07_c2
Contig: 1 of 1

Locus: DID1, AUR1
Supporting Split Reads: 12
Proposed Event: Segmental Anueploid

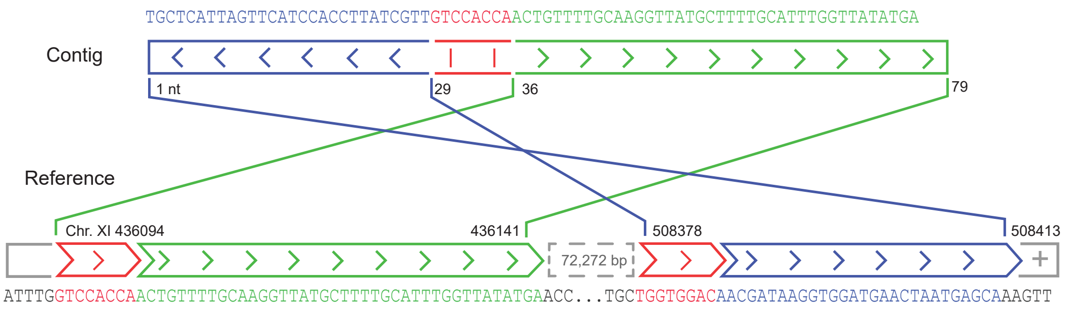

Contig
TGCTC ATTAG TTCAT CCACC TTATC GTTGT CCACC AACTG
TTTTG CAAGG TTATG CTTTT GCATT TGGTT ATATG AACC

Reference Chr. XI 436094 - 436144
ATTTG GTCCA CCAAC TGTTT TGCAA GGTTA TGCTT TTGCA
TTTGG TTATA TGAAC CTG

Reference Chr. XI 508378 - 508413
ATTGC TGGTG GACAA CGATA AGGTG GATGA ACTAA TGAGC
AAAGT T

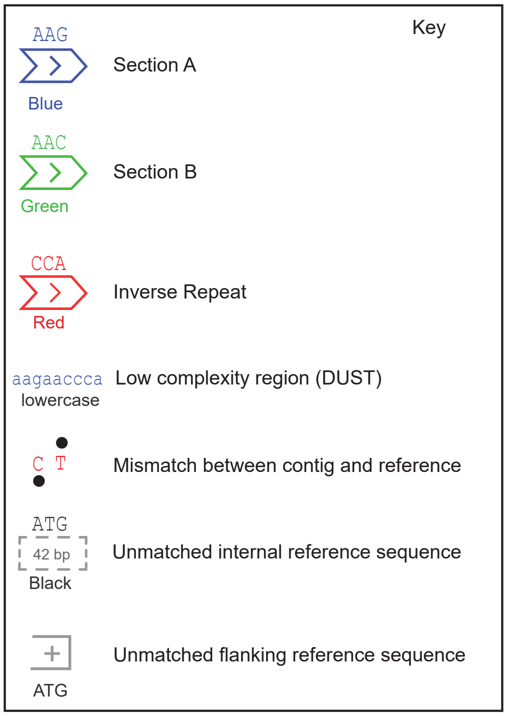

8 Glutamine-limited population 08

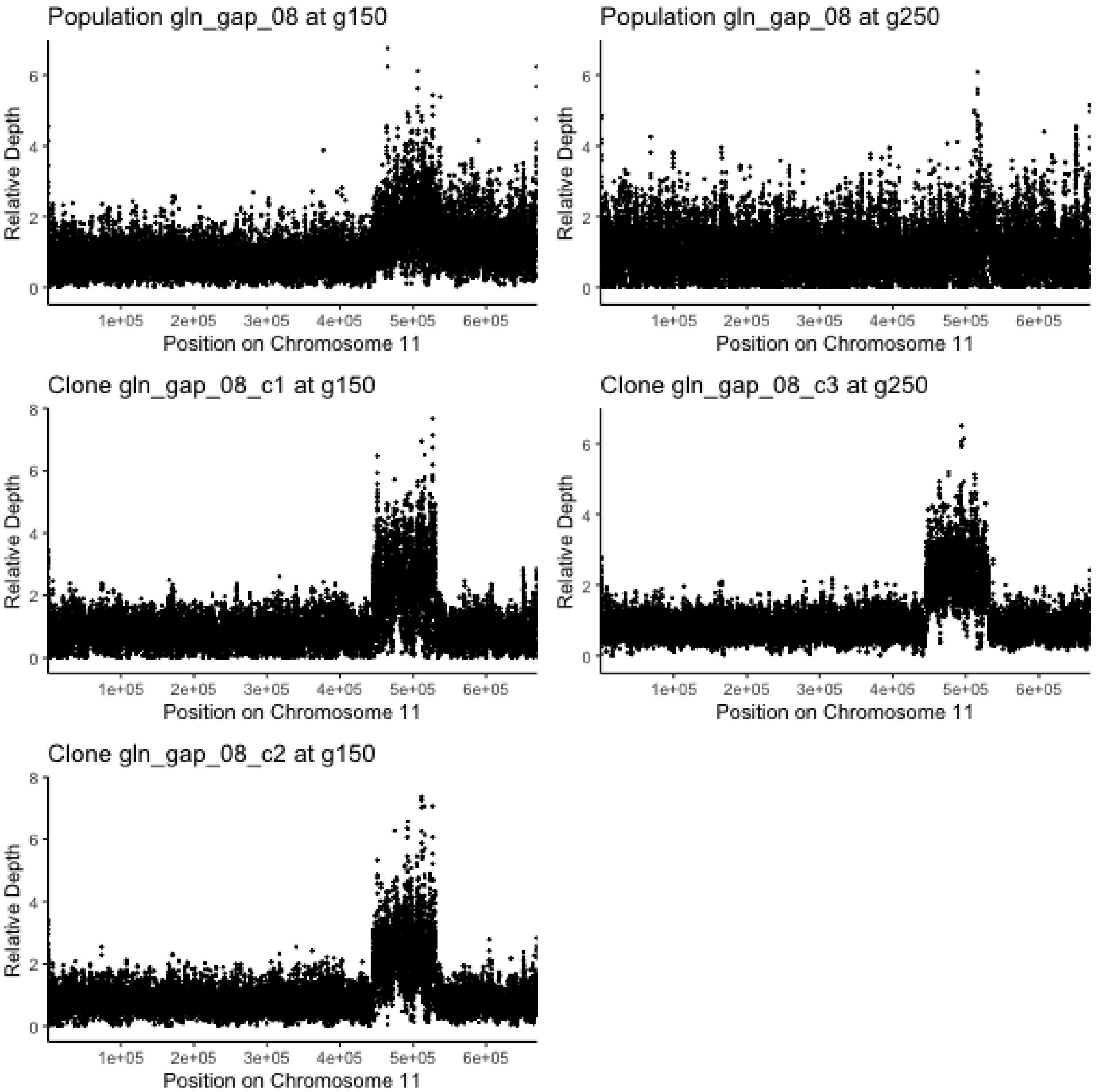

Sample: Gln_08_c1
Contig: 1 of 2

Locus: PAP1
Supporting Split Reads: 16
Proposed Event: CNV, ODIRA

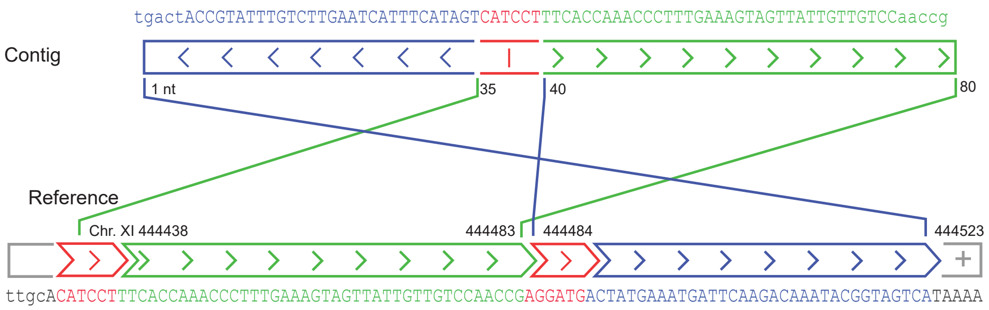

Contig

tgact ACCGT ATTTG TCTTG AATCA TTTCA TAGTC ATCCT
TTCAC CAAAC CCTTT GAAAG TAGTT ATTGT TGTCC aaccg

Reference

ttgcA CATCCT TTCAC CAAAC CCTTT GAAAG TAGTT
ATTGT TGTCCA ACCGA GGATG ACTAT GAAAT GATTC
AAGAC AAATAC GGTAG TCATA AAA

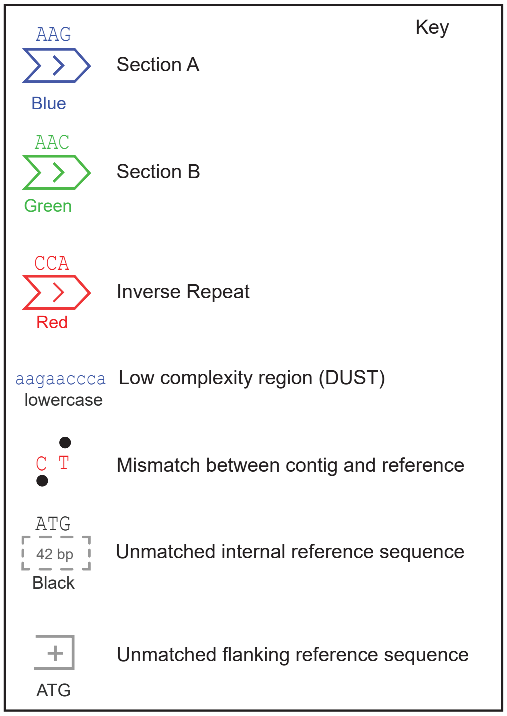

Sample: Gln_08_c1
Contig: 2 of 2

Locus: Intergenic FMP46, TRK2
Supporting Split Reads: 16
Proposed Event: CNV, ODIRA

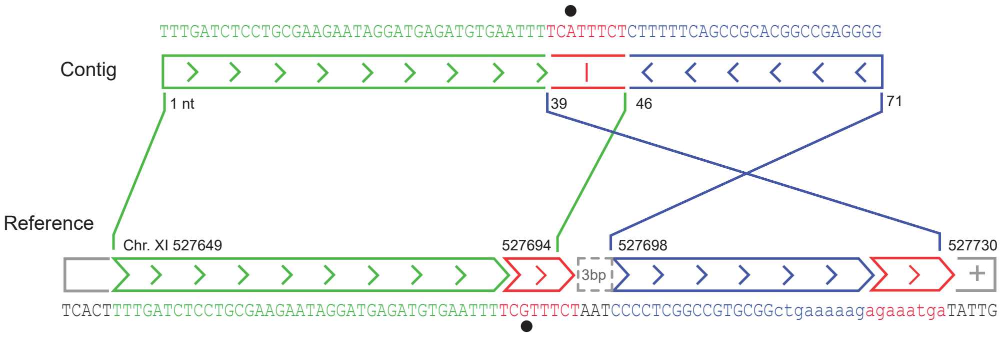

Contig

TTTGA TCTCC TGCGA AGAAT AGGAT GAGAT GTGAA TTTTC
ATTTC TCTTT TTCAG CCGCA CGGCC GAGGG G

Reference

TCACT TTTGA TCTCC TGCGA AGAAT AGGAT GAGAT GTGAA
TTTTC GTTTC TAATC CCCTC GGCCG TGCGG ctgaa aaaga
gaaat gaTAT TG

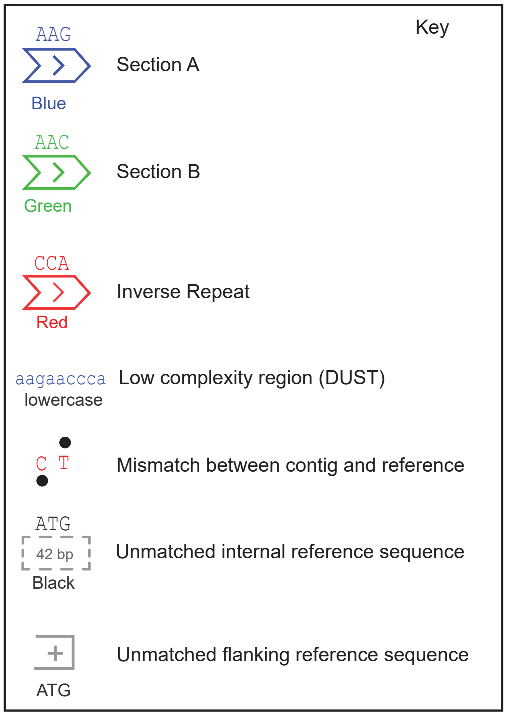

Sample: Gln_08_c2
Contig: 1 of 2

Locus: PAP1
Supporting Split Reads: 15
Proposed Event: CNV, ODIRA

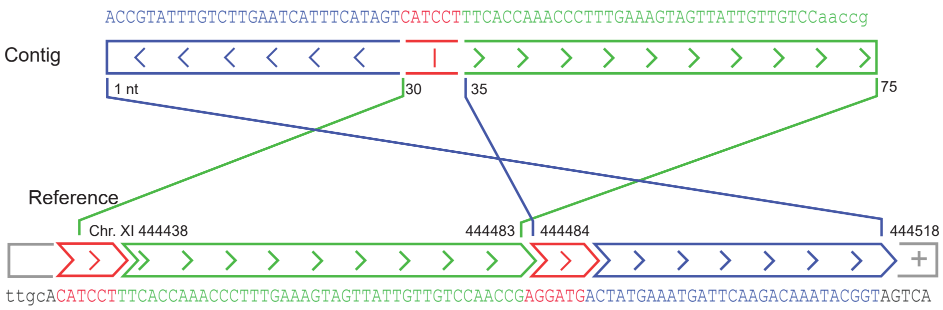

Contig
tgact ACCGT ATTTG TCTTG AATCA TTTCA TAGTC ATCCT
TTCAC CAAAC CCTTT GAAAG TAGTT ATTGT TGTCC aaccg

Reference
ttgcA CATCCT TTCAC CAAAC CCTTT GAAAG TAGTT
ATTGT TGTCCA ACCGA GGATG ACTAT GAAAT GATTC
AAGAC AAATAC GGTAG TCA

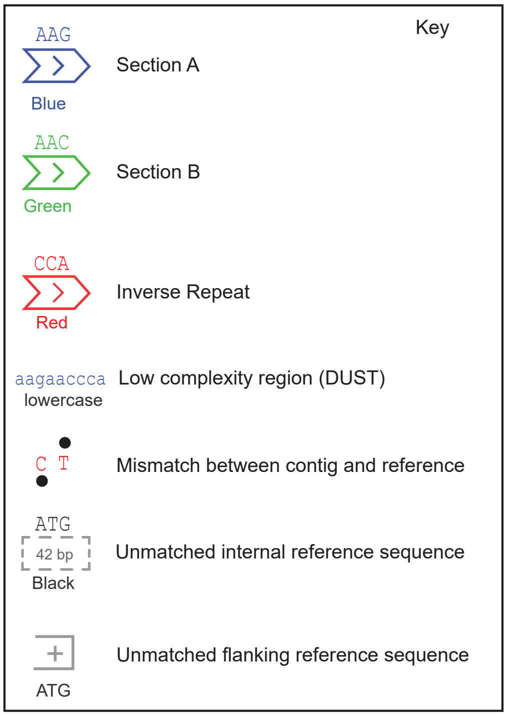

Sample: Gln_08_c2
Contig: 2 of 2

Locus: Intergenic FMP46, TRK2
Supporting Split Reads: 15
Proposed Event: CNV, ODIRA

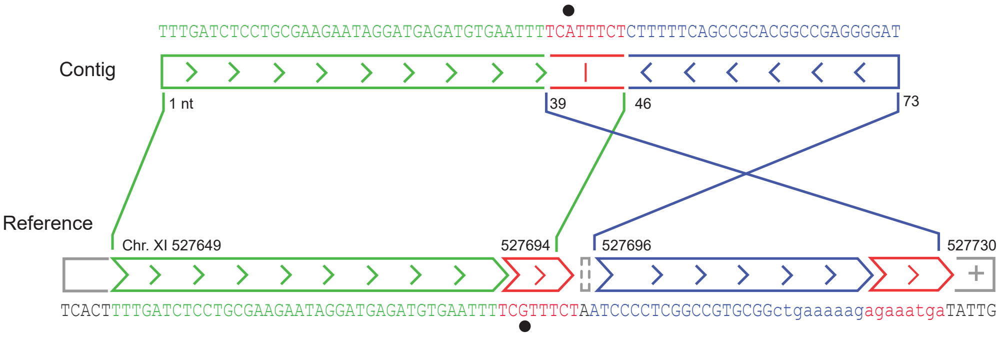

Contig
TTTGA TCTCC TGCGA AGAAT AGGAT GAGAT GTGAA TTTTC
ATTTC TCTTT TTCAG CCGCA CGGCC GAGGG G

Reference
TCACT TTTGA TCTCC TGCGA AGAAT AGGAT GAGAT GTGAA
TTTTC GTTTC TAATC CCCTC GGCCG TGCGG ctgaa aaaga
gaaat gaTAT TG

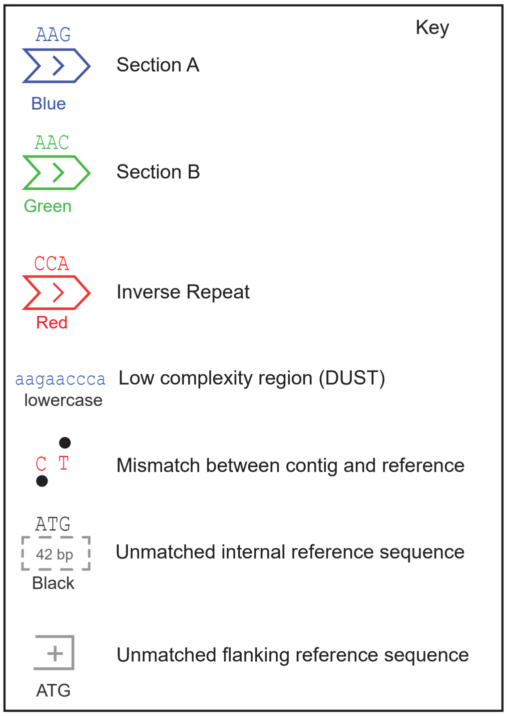

Sample: Gln_08_c3
Contig: 1 of 2

Locus: PAP1
Supporting Split Reads: 8
Proposed Event: CNV, ODIRA

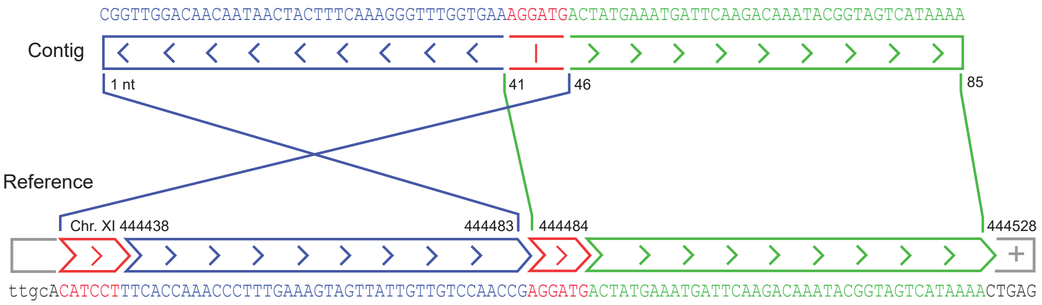

Contig

CGGTT GGACA ACAAT AACTA CTTTC AAAGG GTTTG GTGAA
AGGAT GACTA TGAAA TGATT CAAGA CAAAT ACGGT AGTCA
TAAAA

Reference

ttgcA CATCC TTTCA CCAAA CCCTT TGAAA GTAGT TATTG
TTGTC CAACC GAGGA TGACT ATGAA ATGAT TCAAG ACAAA
TACGG TAGTC ATAAA ACTGA G

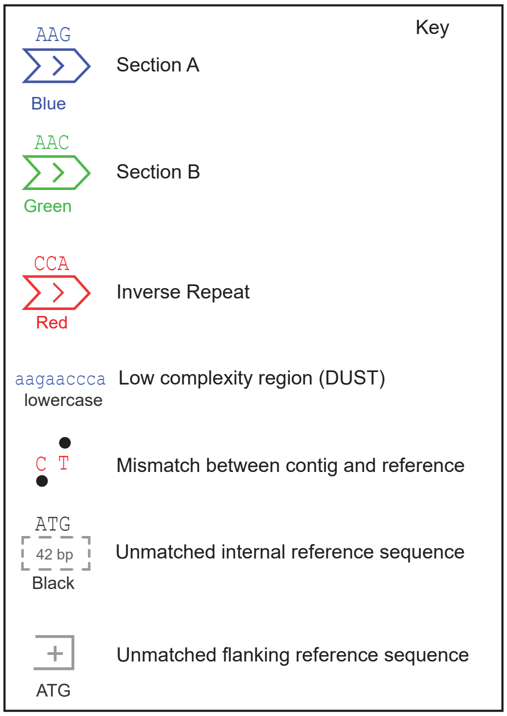

Sample: Gln_08_c3
Contig: 2 of 2

Locus: Intergenic FMP46, TRK2
Supporting Split Reads: 4
Proposed Event: CNV, ODIRA

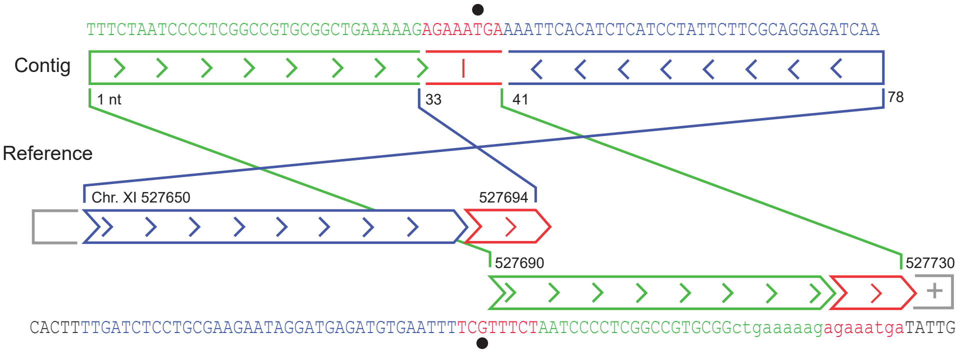

Contig
TTTCT AATCC CCTCG GCCGT GCGGC TGAAA AAGAG AAATG
AAAAT TCACA TCTCA TCCTA TTCTT CGCAG GAGAT CAA

Reference
CACTT TTGAT CTCCT GCGAA GAATA GGATG AGATG TGAAT
TTTCG TTTCT AATCC CCTCG GCCGT GCGGc tgaaa aagag
aaatg aTATT G

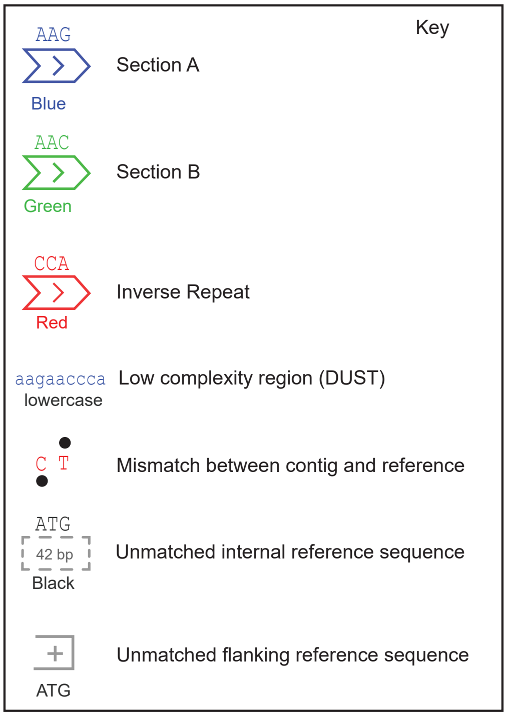

9 Glutamine-limited population 09

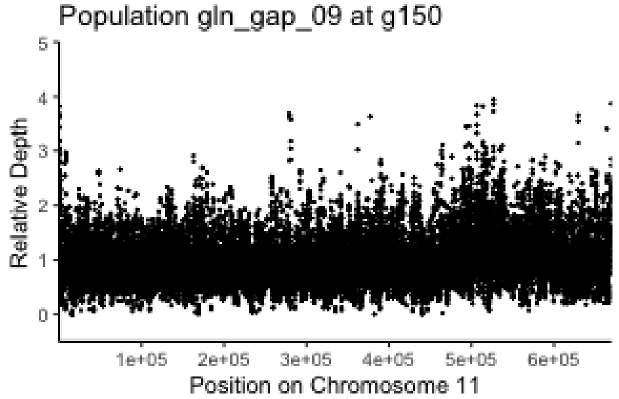

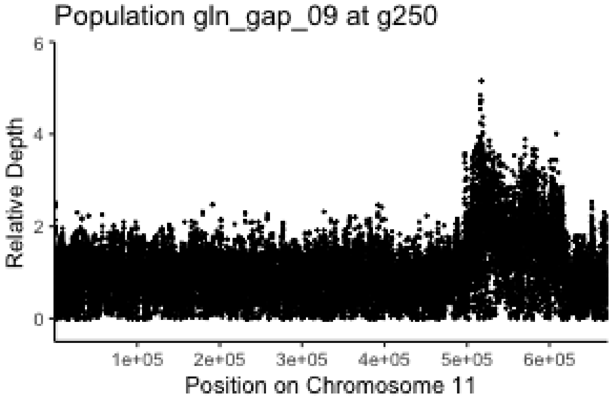

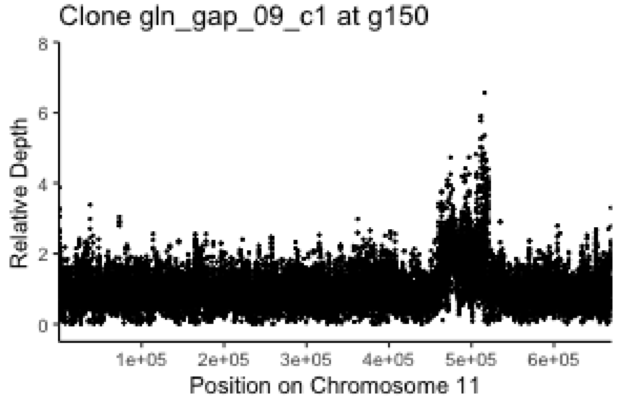

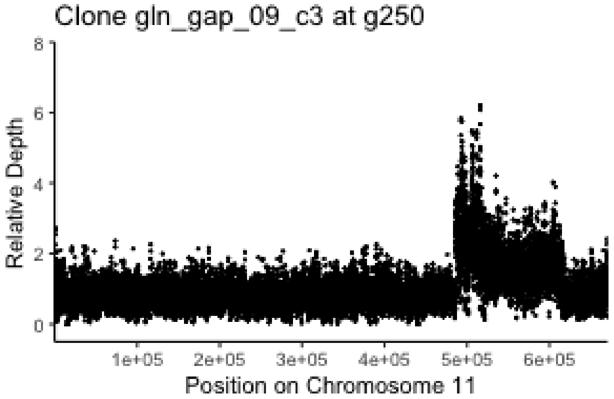

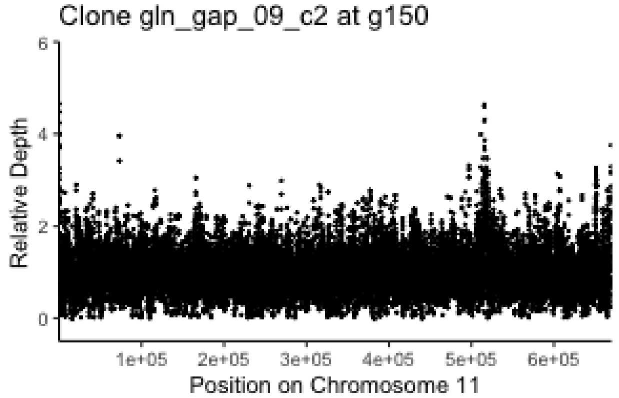

10 Glutamine-limited control populations

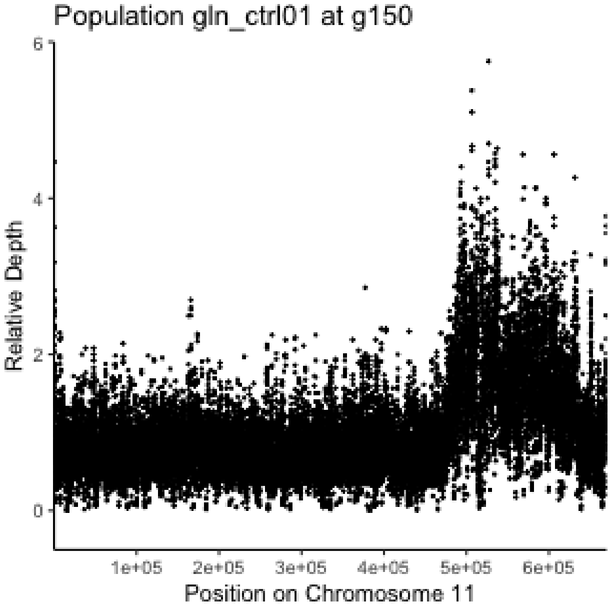

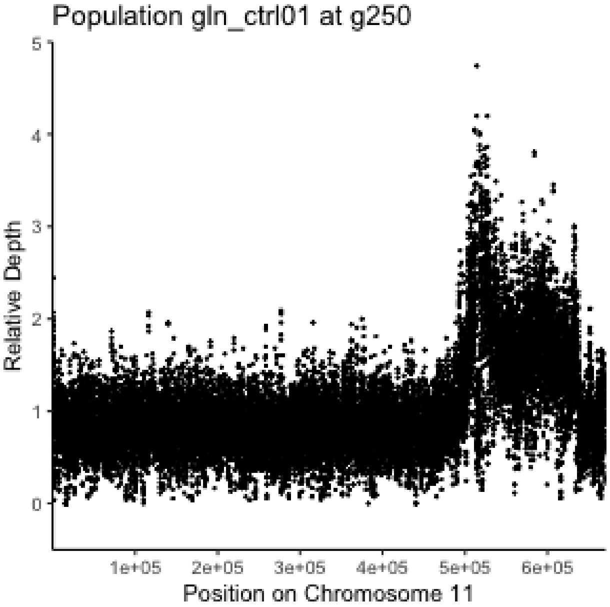

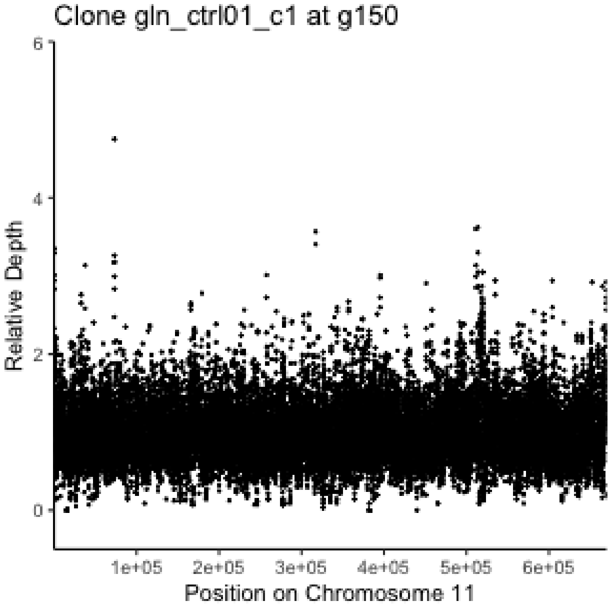

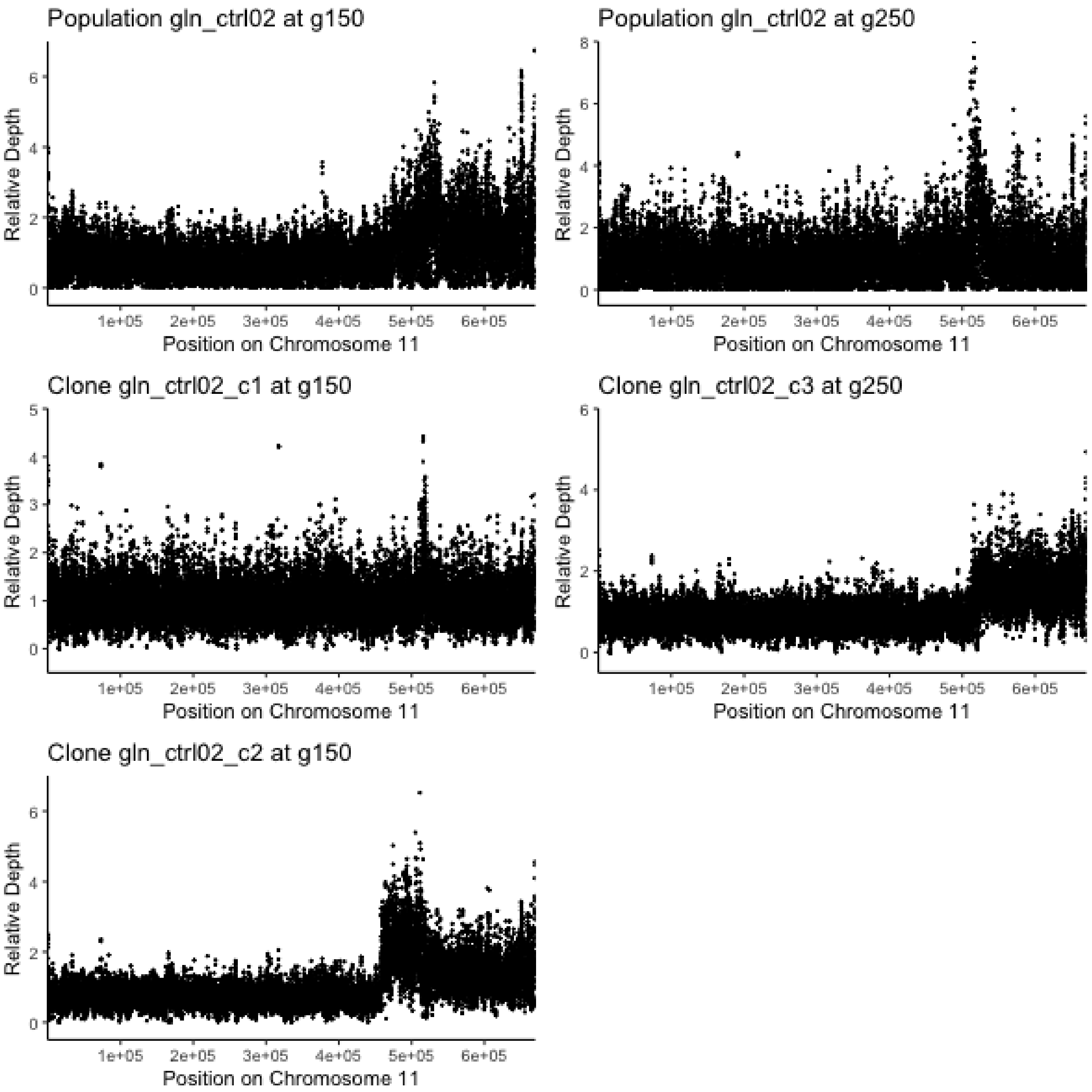
Population gln_ctrl02 at g150
Population gln_ctrl02 at g250
Clone gln_ctrl02_c1 at g150
Clone gln_ctrl02_c3 at g250
Clone gln_ctrl02_c2 at g150
Relative Depth
Position on Chromosome 11
1e+05
2e+05
3e+05
4e+05
5e+05
6e+05

11 Urea-limited 1 copy control population

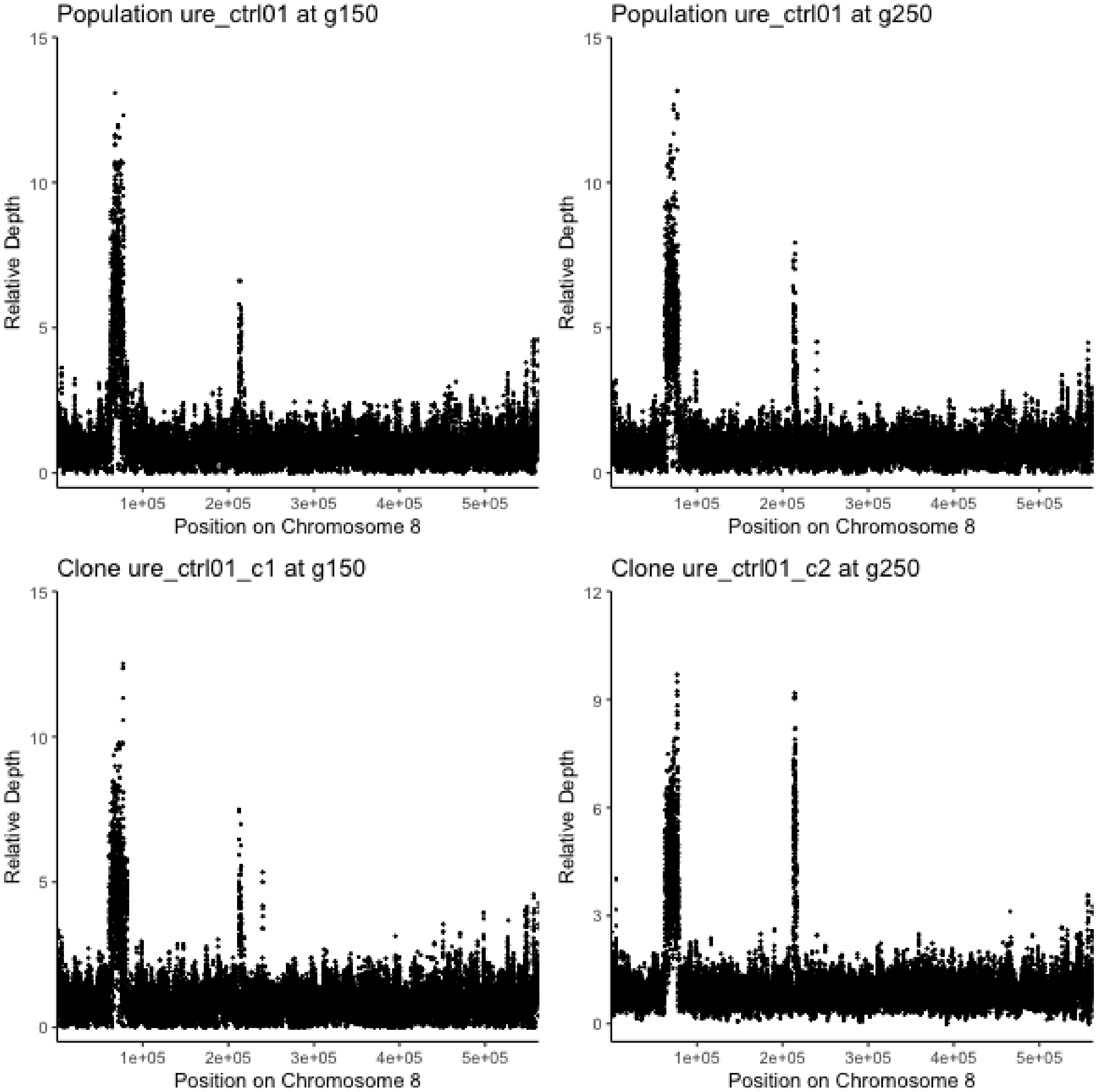

Sample: Ure_ctrl01_c1
Contig: 1 of 1

Locus: NPR3
Supporting Split Reads: 16
Proposed Event: CNV, ODIRA

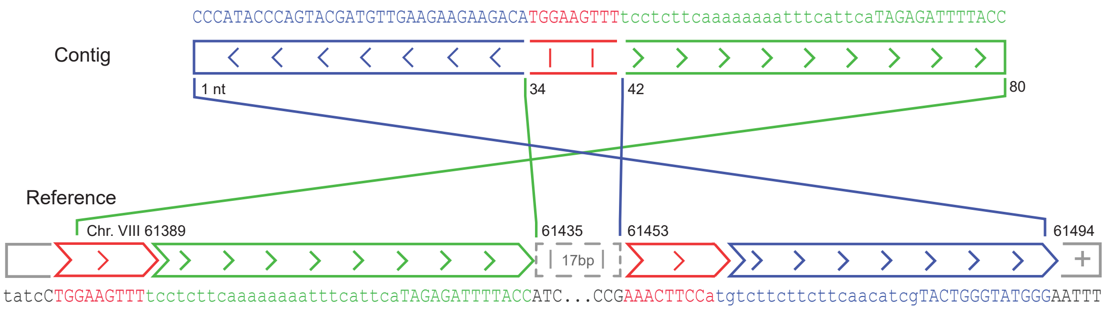

Contig

AACAG GTGCT GCGGG CTCGA GCGCT GATCT CTCAC ATCAA
TCAAC CGCTG TCGAT GAAGA TTCAC TGGAT ATACC

Reference

tatcC TGGAA GTTTt cctct tcaaa aaaaa tttca ttcaT
AGAGA TTTTA CCATC CTGCA ACATT GCCGA AACTT CCatg
tcttc ttctt caaca tcgTA CTGGG TATGG GAATT T

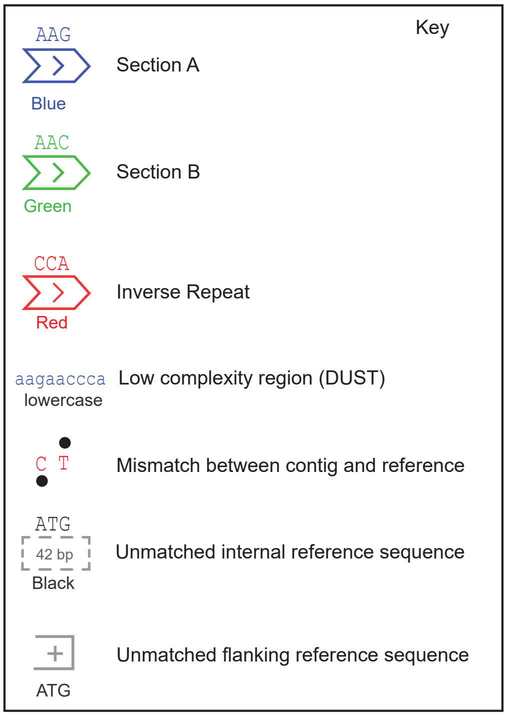

Sample: Ure_ctrl01_c2
Contig: 1 of 1

Locus: SPO11
Supporting Split Reads: 4
Proposed Event: CNV, ODIRA

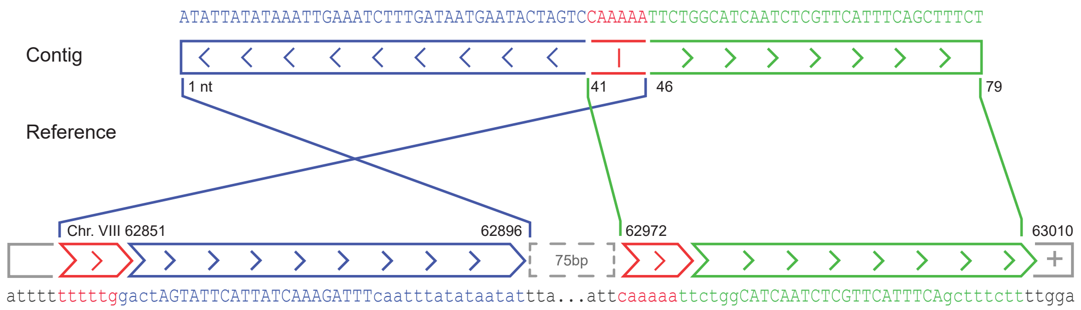

Contig

ATATT ATATA AATTG AAATC TTTGA TAATG AATAC TAGTC
CAAAA ATTCT GGCAT CAATC TCGTT CATTT CAGCT TTCT

Reference

attttt ttttt ggact AGTAT TCATT ATCAA AGATT Tcaat
ttata taata tttaa ATGTA ACcgt tttca attct tgaaa
aacat ttttt ataaa gCAAC AGCTC CCATT CTTAT TCATT
tgtat tcaaa aattc tggCA TCAAT CTCGTTCA TTTCA
gcttt ctttt gga

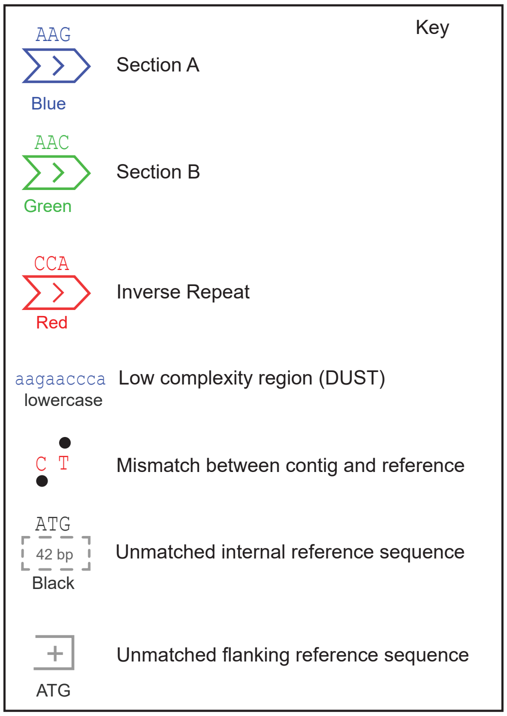

12 Urea-limited 2 copy control population

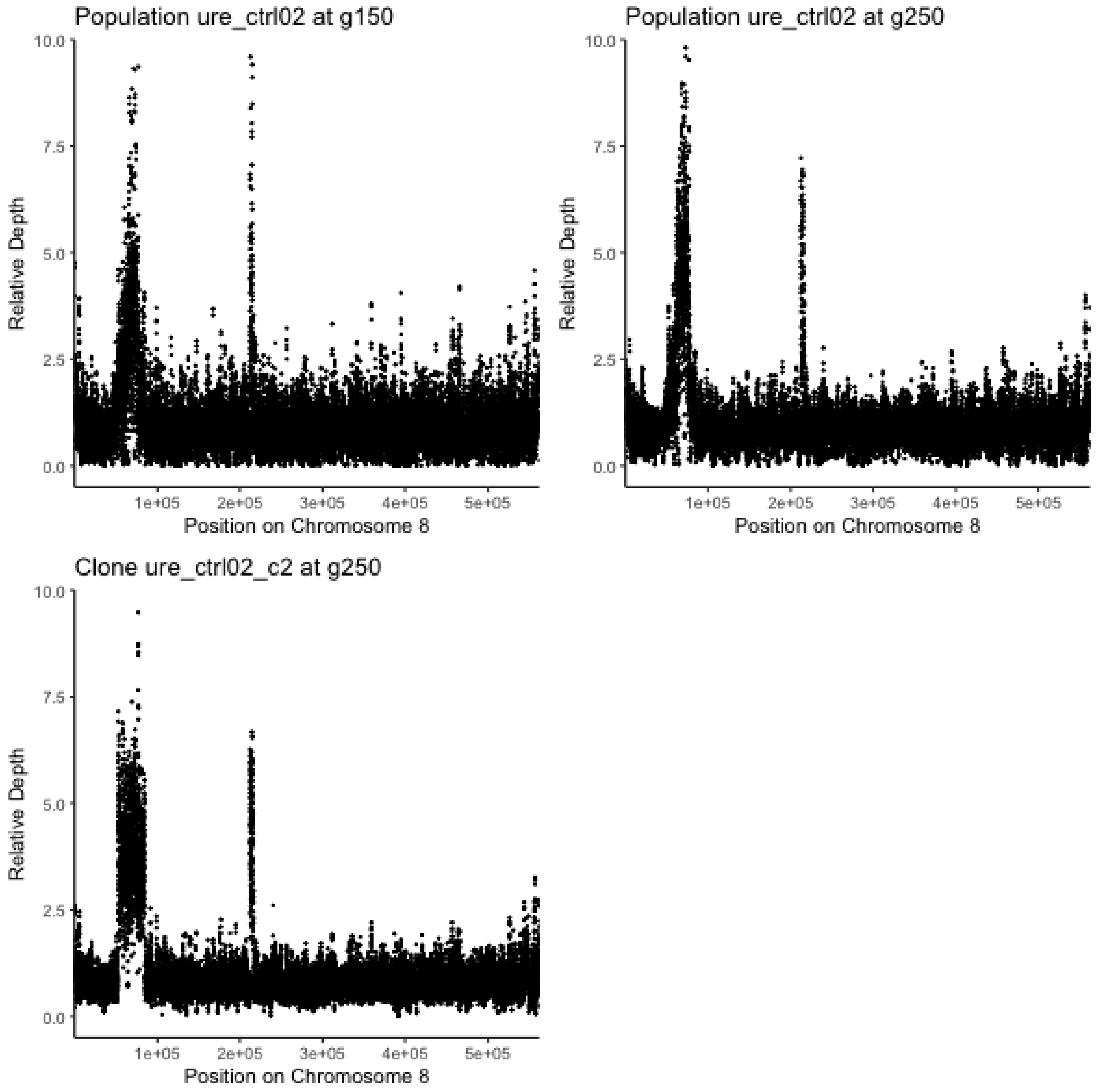

Sample: Ure_ctrl02_c2
Contig: 1 of 2

Locus: Rim101
Supporting Split Reads: 17
Proposed Event: CNV, ODIRA

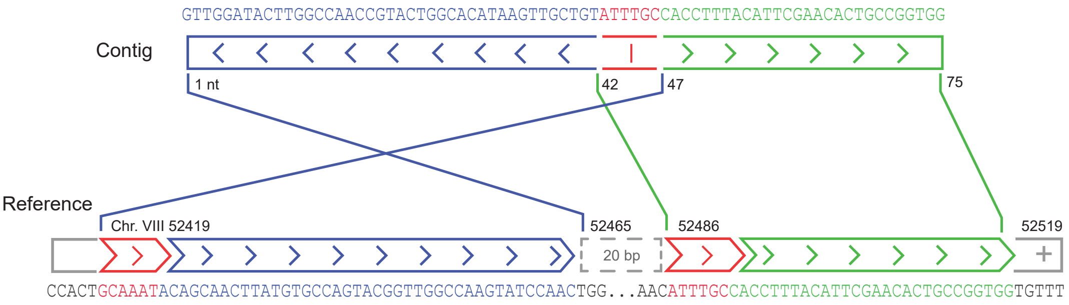

Contig

GTTGG ATACT TGGCC AACCG TACTG GCACA TAAGT TGCTG
TATTT GCCAC CTTTA CATTC GAACA CTGCC GGTGG

Reference

CCACT GCAAA TACAG CAACT TATGT GCCAG TACGG TTGGC
CAAGT ATCCA ACTGG TCCTT CGCTT ACTGA ACATT TGCCA
CCTTT ACATT CGAAC ACTGC CGGTG GTGTT T

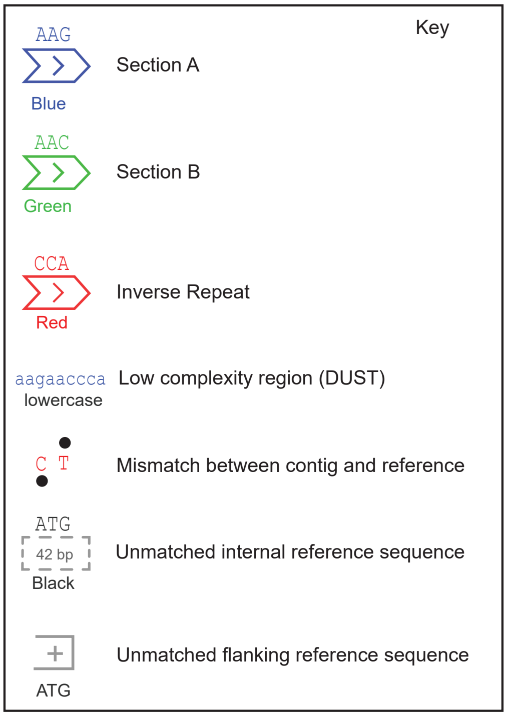

Sample: Ure_ctrl02_c2
Contig: 2 of 2

Locus: Intergenic YAP3
Supporting Split Reads: 27
Proposed Event: CNV, ODIRA

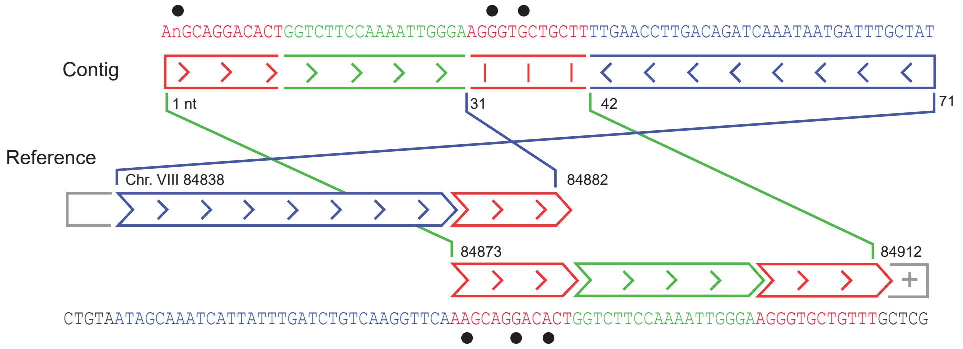

Contig
AnGCA GGACA CTGGT CTTCC AAAAT TGGGA AGGGT GCTGC
TTTTG AACCT TGACA GATCA AATAA TGATT TGCTA T

Reference
CTGTA ATAGC AAATC ATTAT TTGAT CTGTC AAGGT TCAAA
GCAGG ACACT GGTCT TCCAA AATTG GGAAG GGTGC TGTTT
GCTCG

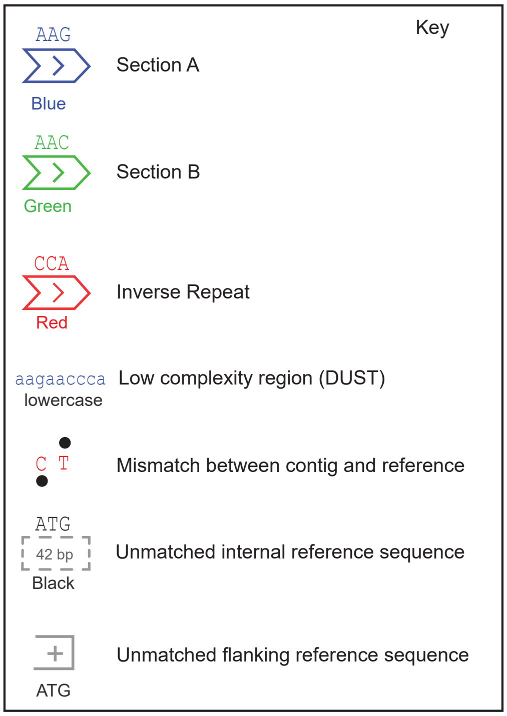

13 Urea-limited population 01

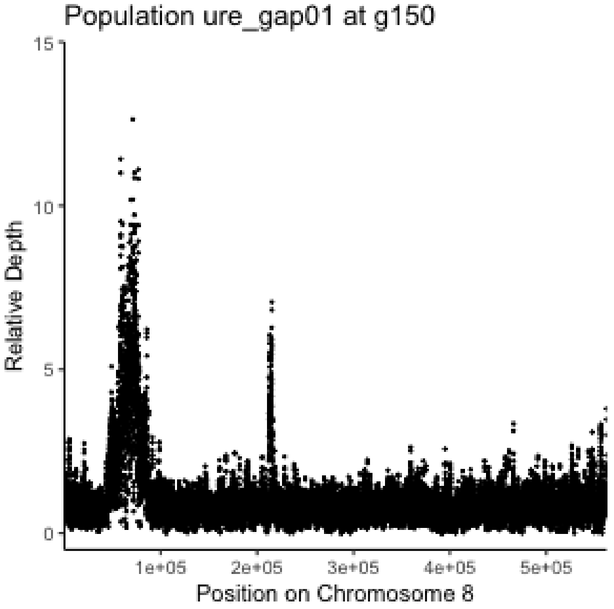

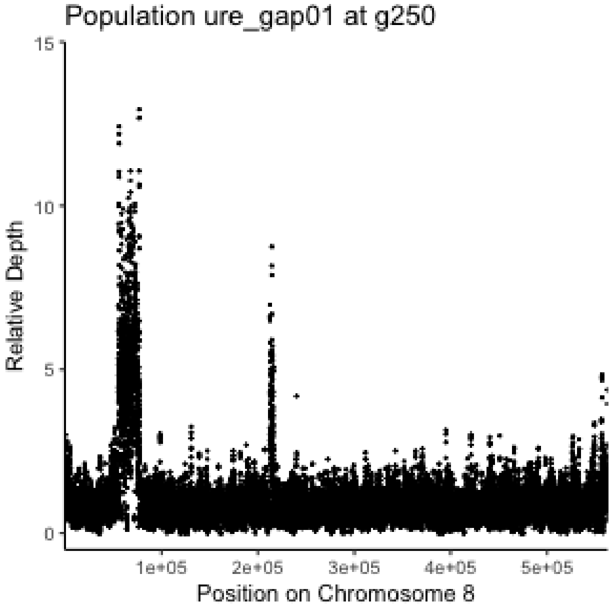

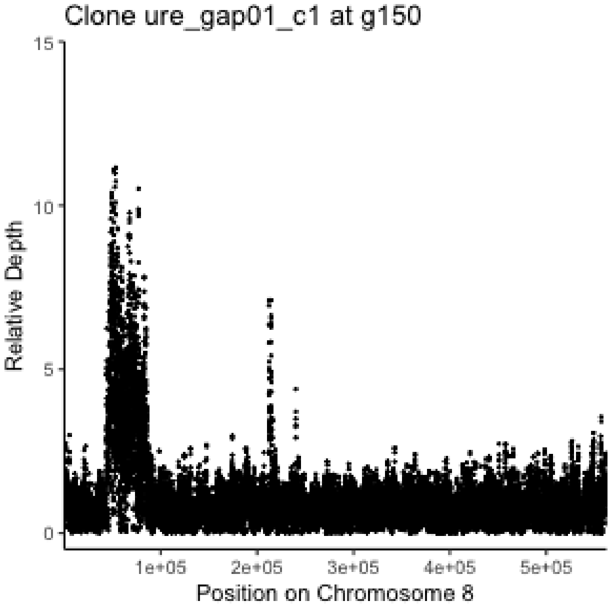

Sample: Ure_01_c1
Contig: 1 of 1

Locus: ECM29
Supporting Split Reads: 9
Proposed Event: CNV, ODIRA

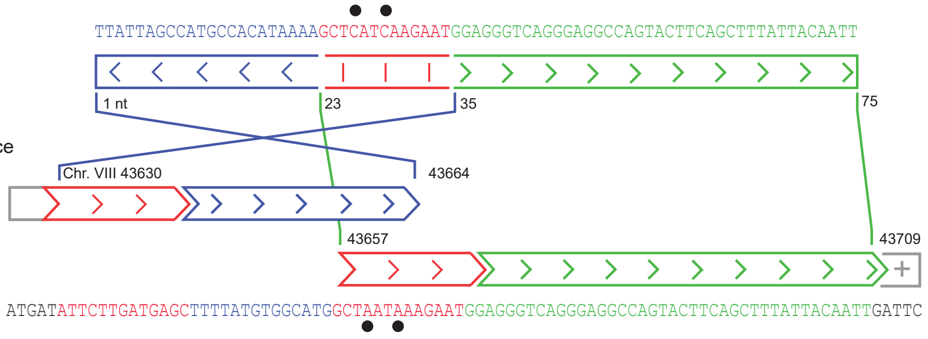

Contig

TTATT AGCCA TGCCA CATAA AAGCT CATCA AGAAT GGAGG
GTCAG GGAGG CCAGT ACTTC AGCTT TATTA CAATT

Reference

ATGAT ATTCT TGATG AGCTT TTATG TGGCA TGGCT AATAA
AGAAT GGAGG GTCAG GGAGG CCAGT ACTTC AGCTT TATTA
CAATT GATTC

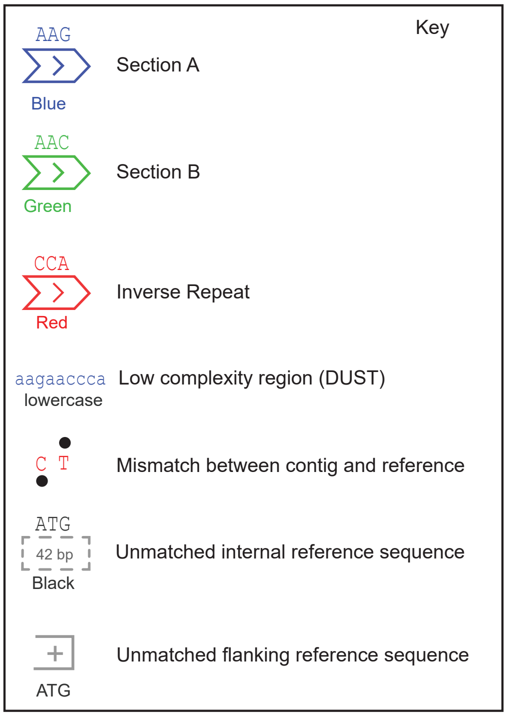

14 Urea-limited population 02

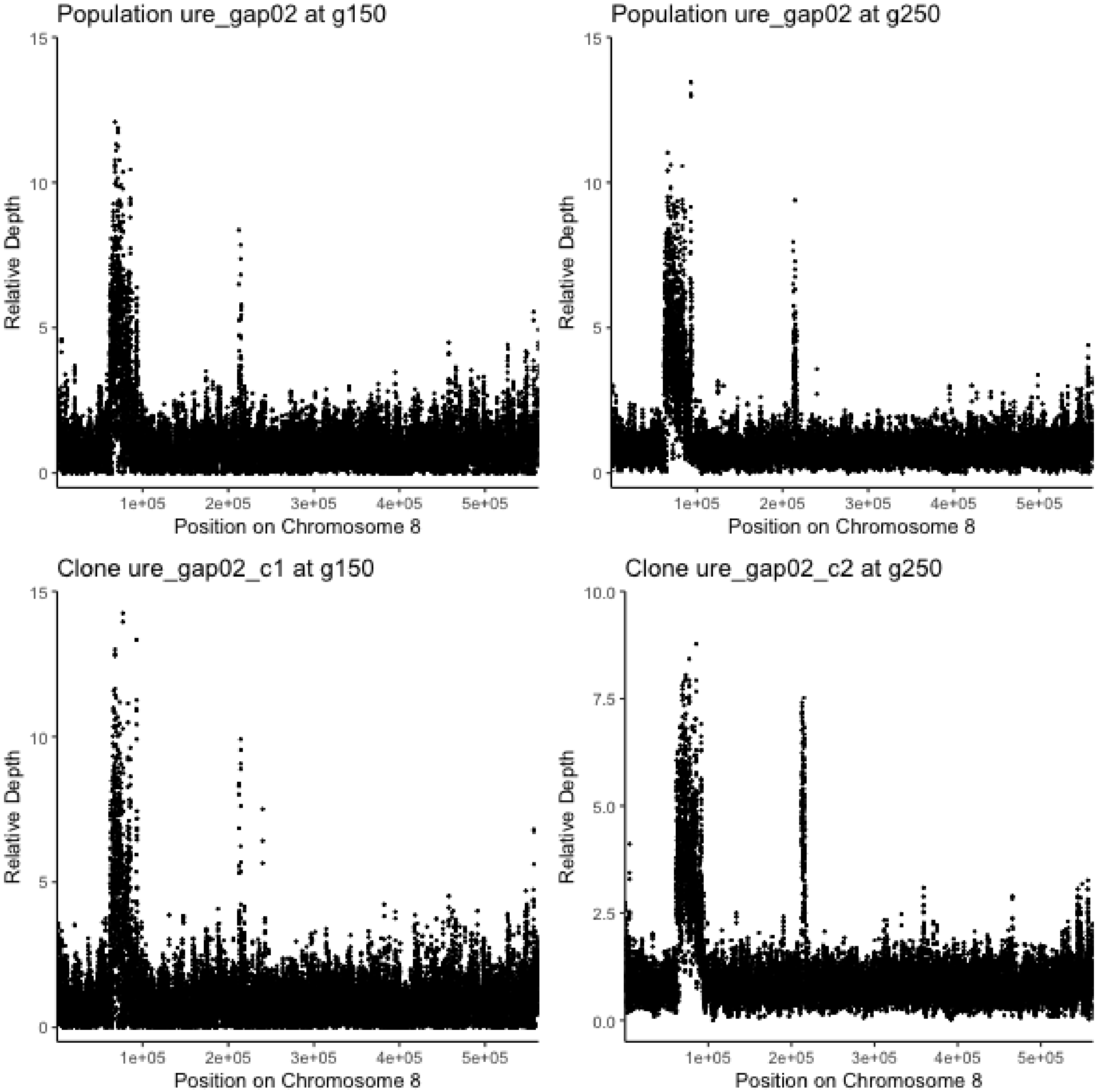

Sample: Ure_02_c2
Contig: 1 of 1

Locus: NPR3
Supporting Split Reads: 15
Proposed Event: CNV, ODIRA

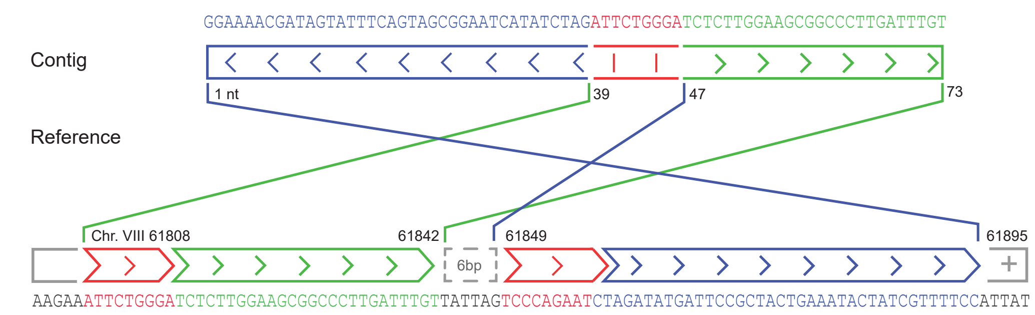

Contig

GGAAA ACGAT AGTAT TTCAG TAGCG GAATC ATATC TAGAT
TCTGG GATCT CTTGG AAGCG GCCCT TGATT TGT

Reference

AAGAA ATTCT GGGAT CTCTT GGAAG CGGCC CTTGA TTTGT
TATTA GTCCC AGAAT CTAGA TATGA TTCCG CTACT GAAAT
ACTAT CGTTT TCCAT TAT

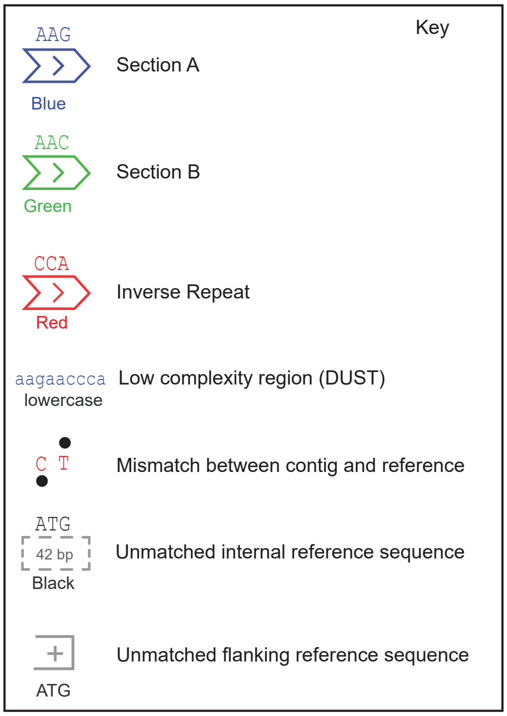

15 Urea-limited population 04

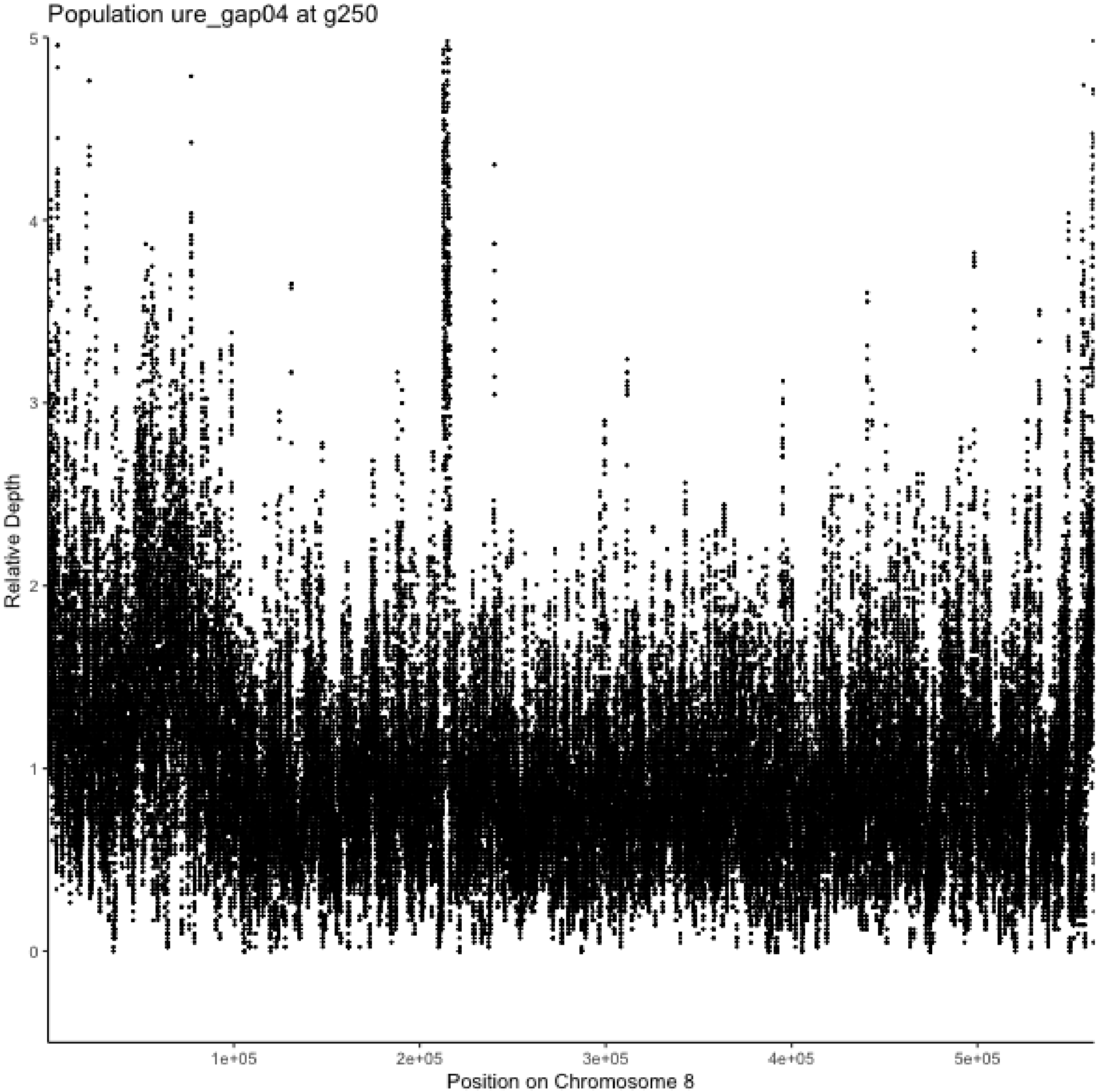

16 Urea-limited population 05

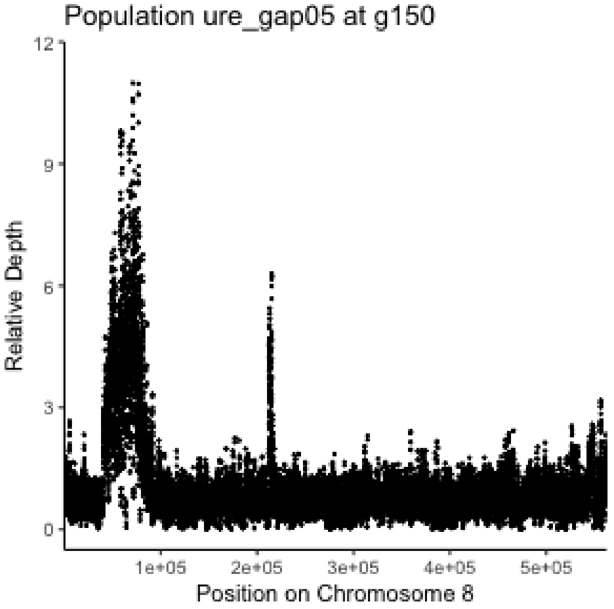

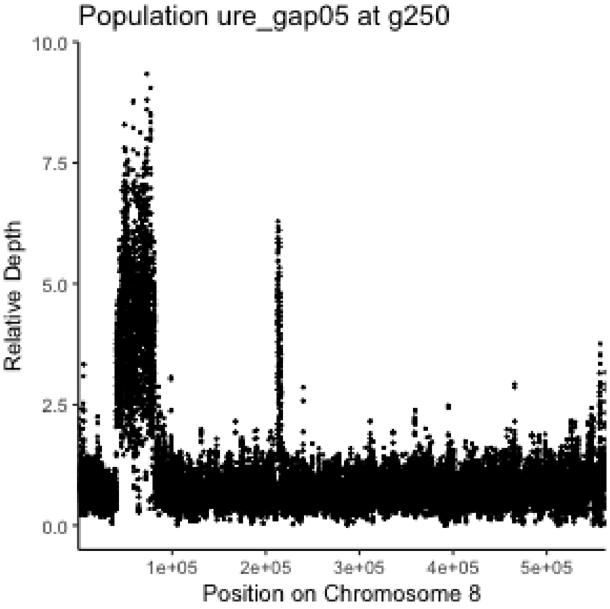

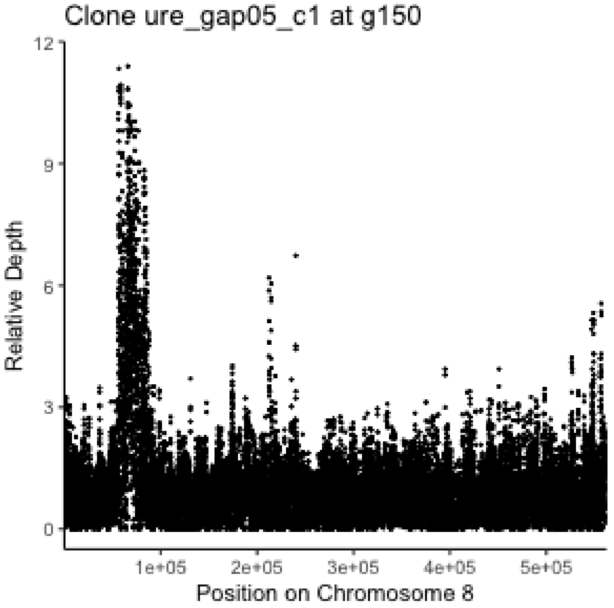

Sample: Ure_05_c1
Contig: 1 of 1

Locus: Intergenic SNF6, RIM4
Supporting Split Reads: 24
Proposed Event: CNV, ODIRA

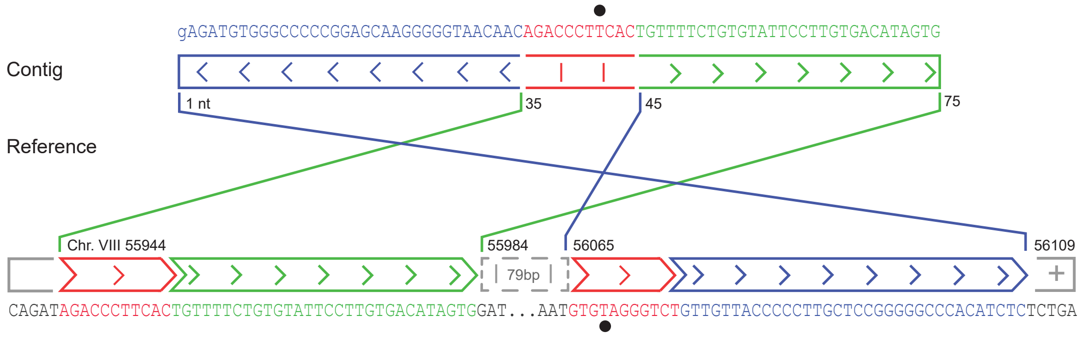

Contig

gAGAT GTGGG CCCCC GGAGC AAGGG GGTAA CAACA GACCC
TTCAC TGTTT TCTGT GTATT CCTTG TGACA TAGTG

Reference

CAGAT AGACC CTTCA CTGTT TTCTG TGTAT TCCTT GTGAC
ATAGT GGATT TGTGC AGCTA CTGCT TATAG GAAGC AGTGC
CTGCA AAACA ATTCA TTCGT TGGTT GATGT GGCGC GCTGA
CTGAA TGTGT AGGGT CTGTT GTTAC CCCCT TGCTC CGGGG
GCCCA CATCT CTCTG A

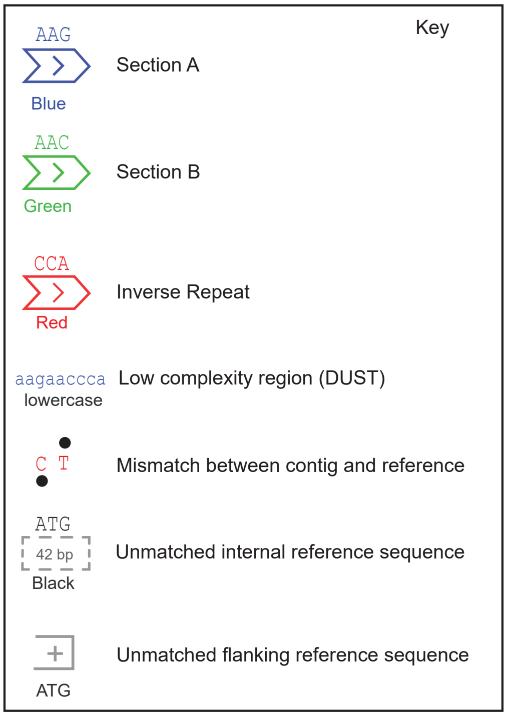

17 Urea-limited population 06

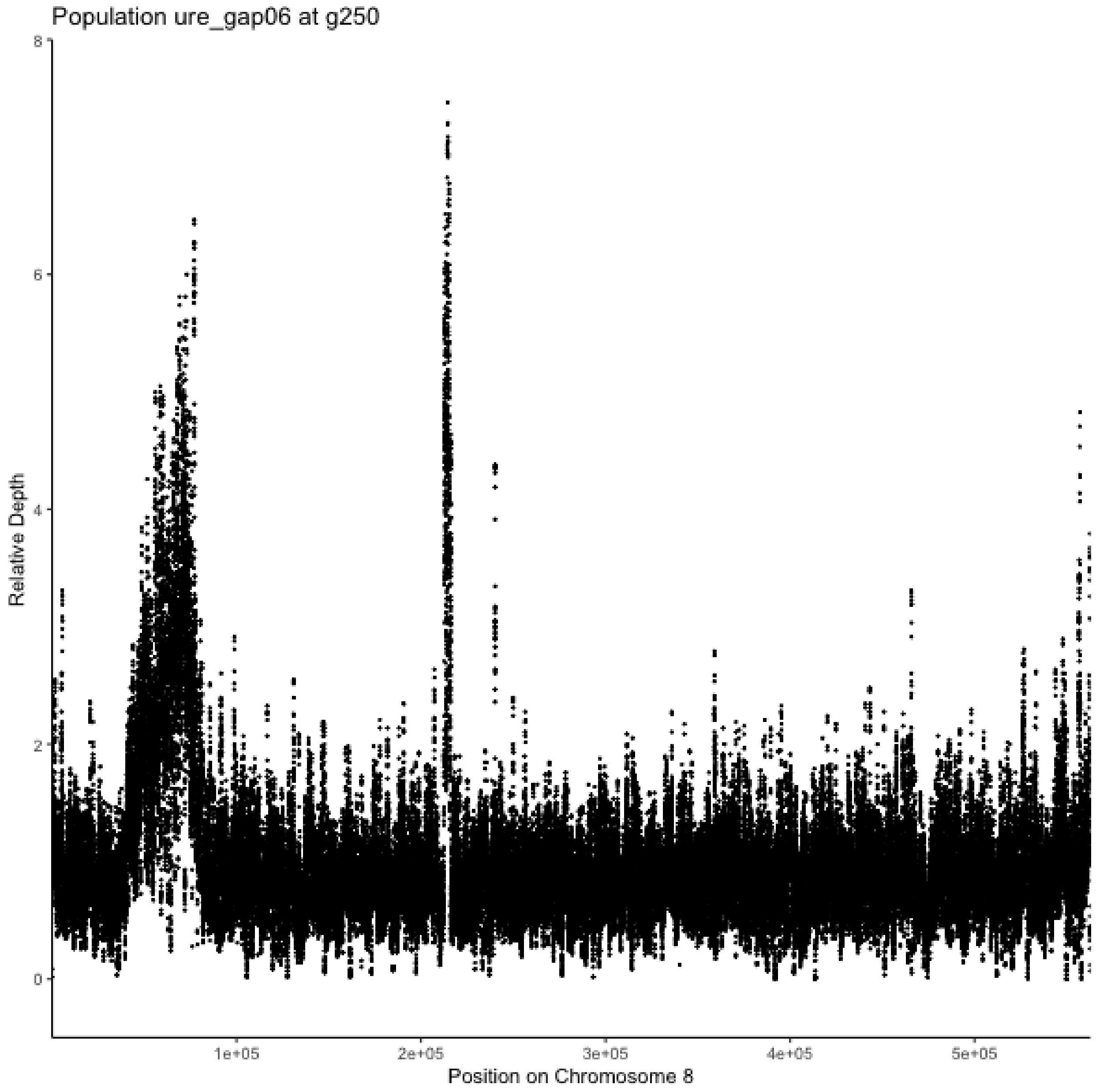

18 Urea-limited population 07

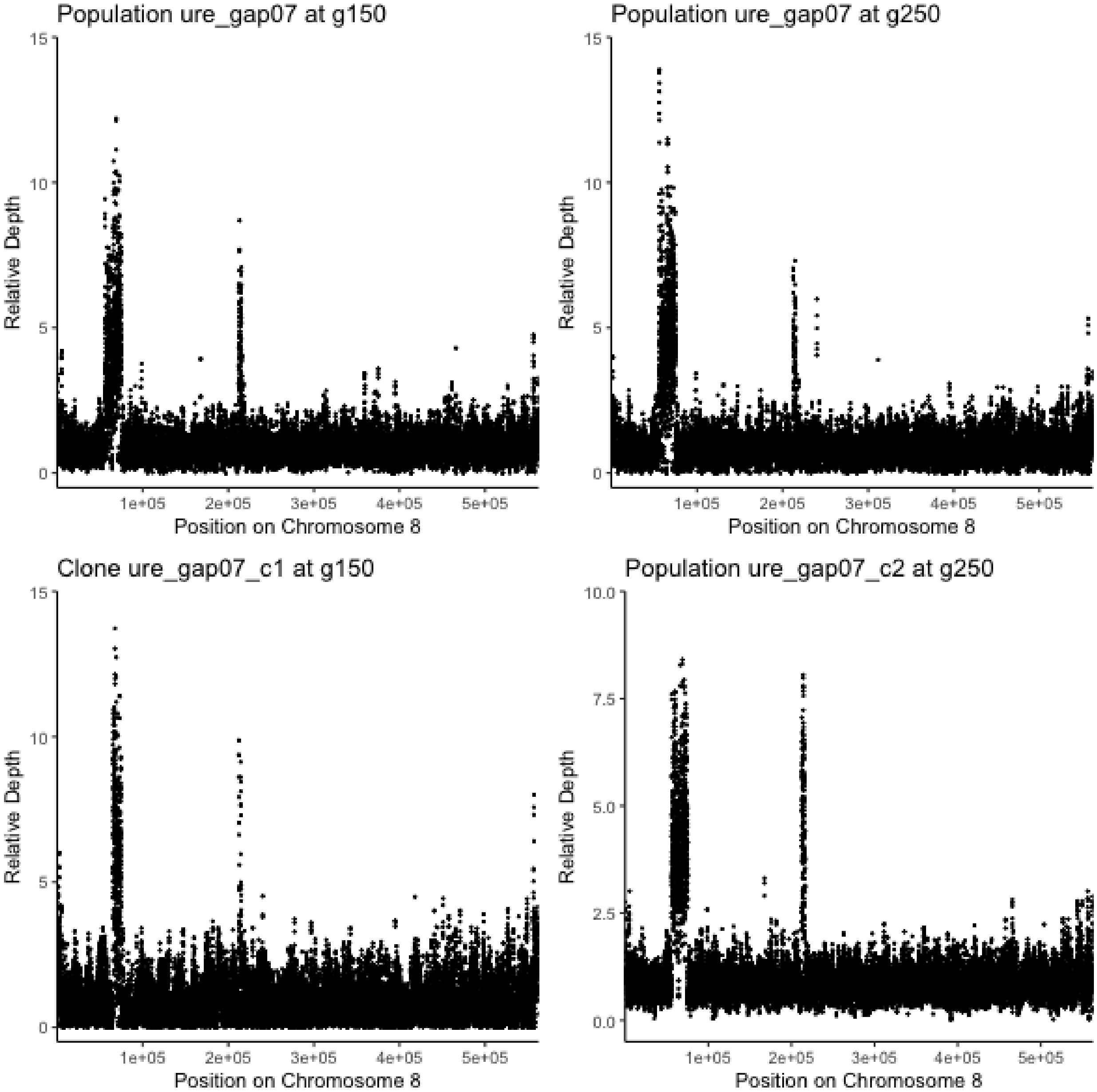

Sample: Ure_07_c1
Contig: 1 of 1

Locus: AIM17
Supporting Split Reads: 24
Proposed Event: CNV, ODIRA

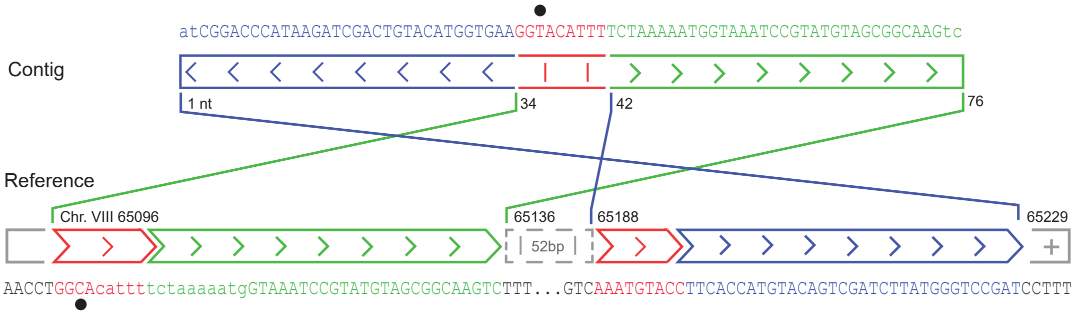

Contig

atCGG ACCCA TAAGA TCGAC TGTAC ATGGT GAAGG TACAT
TTTCT AAAAA TGGTA AATCC GTATG TAGCG GCAAG tc

Reference

AACCT GGCAc atttt ctaaa aatgG TAAAT CCGTA TGTAG
CGGCA AGTCT TTATT GGCAT AATGG GCATT AACAC TTGTC
GCTTG GGATG CATTC ACGTC AAATG TACCT TCACC ATGTA
CAGTC GATCT TATGG GTCCG ATCCT TT

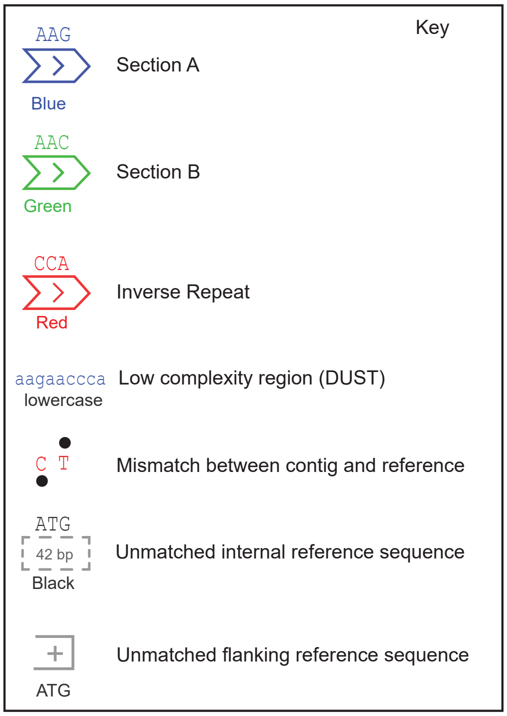

Sample: Ure_07_c2
Contig: 1 of 2

Locus: SNF6
Supporting Split Reads: 13
Proposed Event: CNV, ODIRA

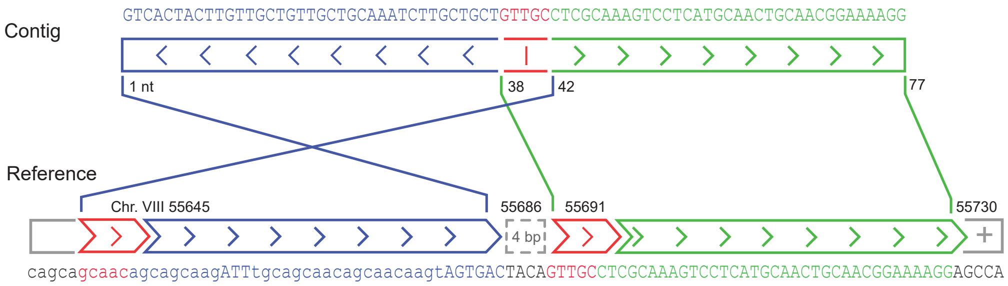

Contig

```
GTCAC TACTT GTTGC TGTTG CTGCA AATCT TGCTG CTGTT
GCCTC GCAAA GTCCT CATGC AACTG CAACG GAAAA GG
```

Reference

```
cagca gcaac agcag caagA TTtgc agcaa cagca acaag
tAGTG ACTAC AGTTG CCTCG CAAAG TCCTC ATGCA ACTGC
AACGG AAAAG GAGCC A
```

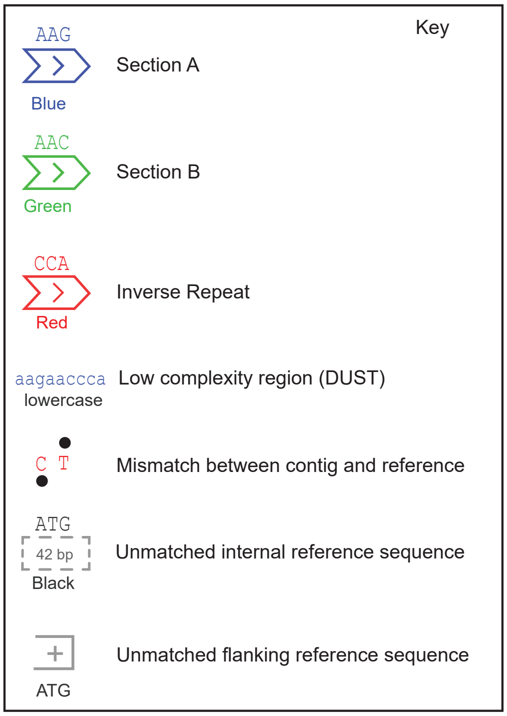

Sample: Ure_07_c2
Contig: 2 of 2

Locus: Intergenic YHL015W-A, RPS20
Supporting Split Reads: 12
Proposed Event: CNV, ODIRA

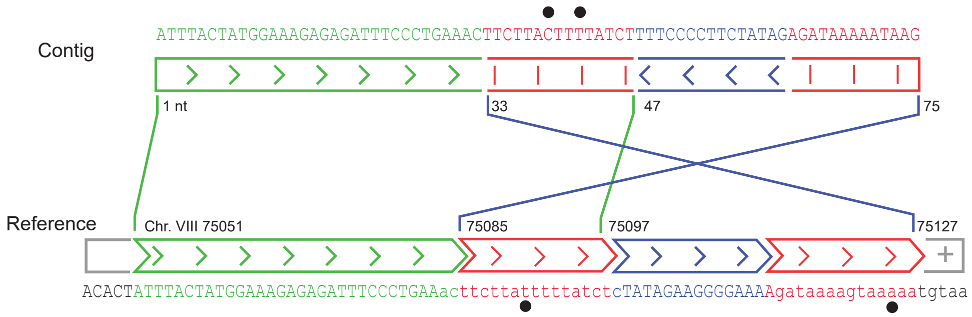

Contig

ATTTA CTATG GAAAG AGAGA TTTCC CTGAA ACTTC TTACT
TTTAT CTTTT CCCCT TCTAT AGAGA TAAAA ATAAG

Reference

ACACT ATTTA CTATG GAAAG AGAGA TTTCC CTGAA acttc
ttatt tttat ctcTA TAGAA GGGGA AAAga taaaa gtaaa
aatgt aa

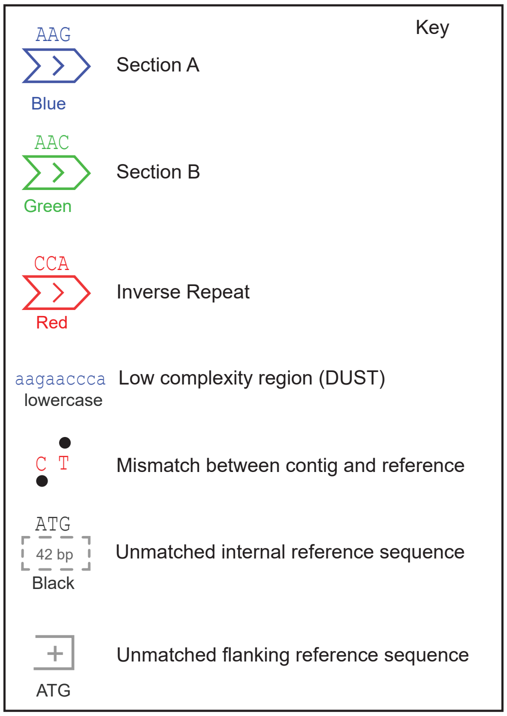

19 Urea-limited population 08

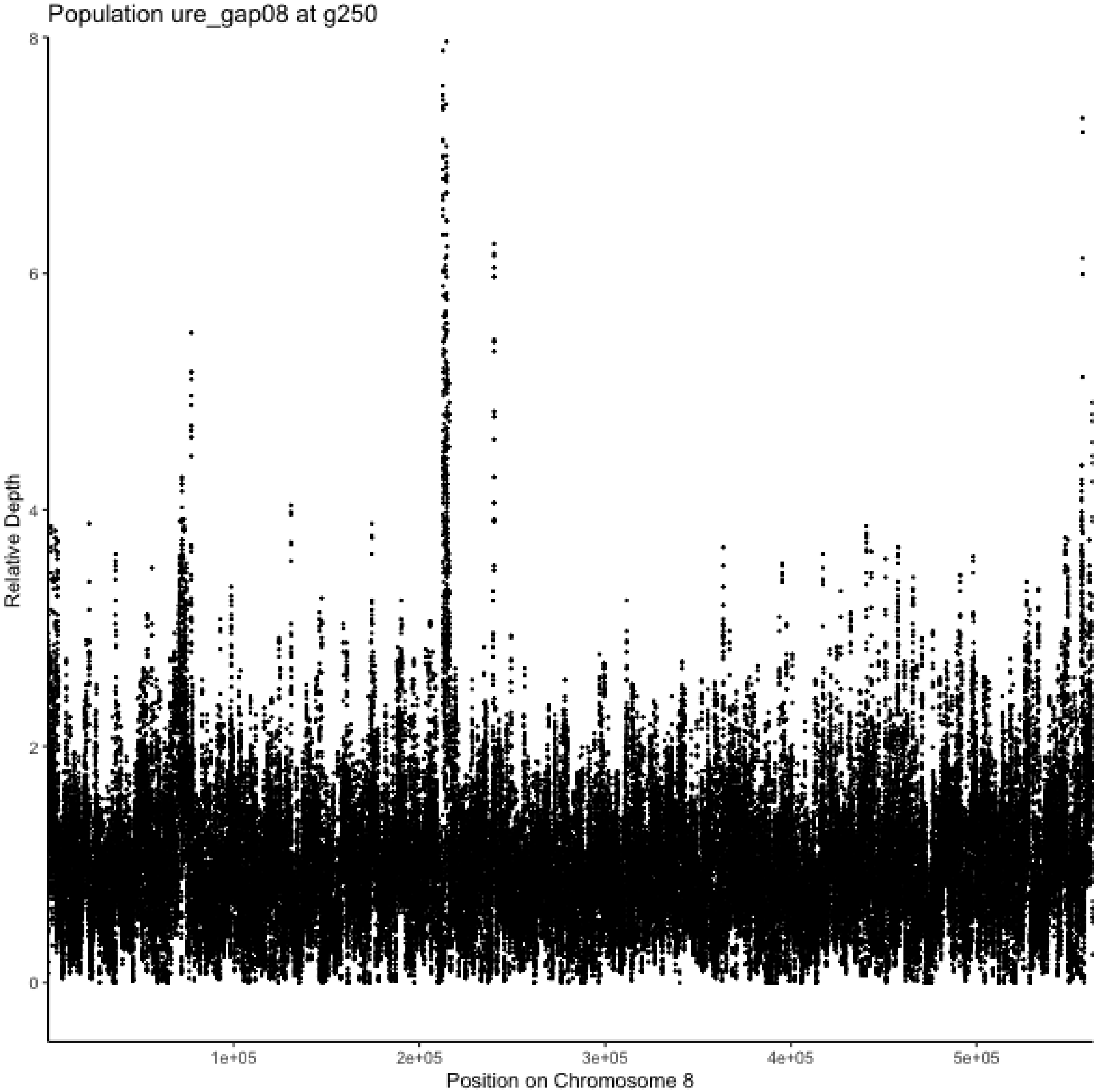

Sample: Ure_05_c1
Contig: 1 of 1

Locus: Intergenic SNF6, RIM4
Supporting Split Reads: 24
Proposed Event: CNV, ODIRA

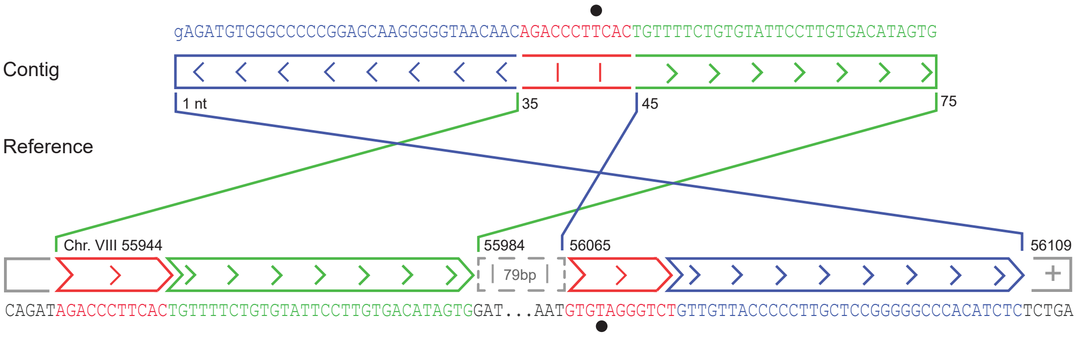

Contig

gAGAT GTGGG CCCCC GGAGC AAGGG GGTAA CAACA GACCC
TTCAC TGTTT TCTGT GTATT CCTTG TGACA TAGTG

Reference

CAGAT AGACC CTTCA CTGTT TTCTG TGTAT TCCTT GTGAC
ATAGT GGATT TGTGC AGCTA CTGCT TATAG GAAGC AGTGC
CTGCA AAACA ATTCA TTCGT TGGTT GATGT GGCGC GCTGA
CTGAA TGTGT AGGGT CTGTT GTTAC CCCCT TGCTC CGGGG
GCCCA CATCT CTCTG A

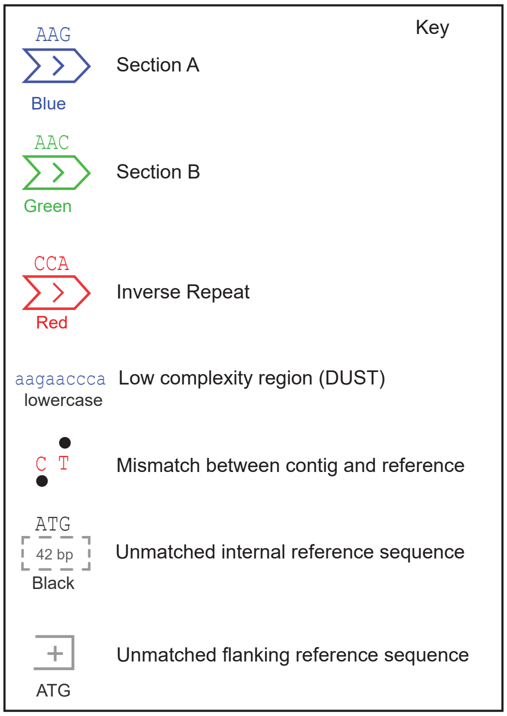

Supplement: S2 Text — Relative read-depth plots for each population and corresponding clones isolated from these populations at generation 150 and 250. For a subset of clones with GAP1 and DUR3 CNVs, breakpoint maps are shown. Breakpoint maps were generated using local assembly of split reads and alignment to the reference genome. Code used to generate these figures can be accessed in OSF: https://osf.io/fxhze/. CNV, copy number variant. (PDF) [file pbio.3000069.s002.pdf]
