# Supplementary material for: Single-cell copy number variant detection reveals the dynamics and diversity of adaptation
Source: PLoS Biol. 2018 Dec 18;16(12):e3000069. doi: 10.1371/journal.pbio.3000069 (PMC6298651; doi:10.1371/journal.pbio.3000069)
Supplement: S3 Text — Application of existing CNV detection algorithms for analysis of genome sequencing data. CNV, copy number variant. (DOCX) [file pbio.3000069.s003.docx]

### Application of existing CNV detection algorithms fails to identify *GAP1* and *DUR3* duplications

The identification of CNVs using a fluorescent reporter enables assessment of the extent to which current algorithms are able to detect CNVs using short read sequencing. To evaluate algorithm performance, we first tested each algorithm using simulated data (**methods**). We simulated haploid clonal samples and found that LUMPY, SvABA and Pindel performed reasonably well (FDR < 5% and F-score > 80%) with average genome coverages ranging from 5X-50X (**S8 Fig**). We then simulated mixed non-clonal populations containing CNVs at differing allele frequencies. We found that LUMPY and SvABA performed well in all scenarios whereas Pindel performed well only when simulated populations contained CNVs at frequencies of at least 50% **(S9 Fig)**.

On the basis of these results, we generated an in-house pipeline for detecting CNVs that integrates results from Pindel, Lumpy, and SvABA [[61–63]](https://paperpile.com/c/AZcECH/df4lt+t6i4z+UyoBM). We first applied this pipeline to clonal CNV samples with increased fluorescence. We found that in 29% of cases, in which a CNV is predicted to be present on the basis of fluorescence and clear increases in sequencing read depth, all three algorithms failed to call a CNV at the *GAP1* locus. LUMPY consistently identified the most breakpoints, which we compared against manual CNV boundary calls (**S5 Table**). This high false negative rate was further exacerbated for population sequencing data in which fluorescence clearly indicated the presence of CNV lineages at >50% frequencies: in 28% of tested populations a CNV was not called by any of the algorithms (**S6 Table**). We conclude that existing CNV calling algorithms are poorly suited to detection of CNVs in heterogeneous samples and suffer from a high false negative rate even for clonal haploid samples.
