# Supplementary figures and images for: Single-cell copy number variant detection reveals the dynamics and diversity of adaptation
Source: PLoS Biol. 2018 Dec 18;16(12):e3000069. doi: 10.1371/journal.pbio.3000069 (PMC6298651; doi:10.1371/journal.pbio.3000069)

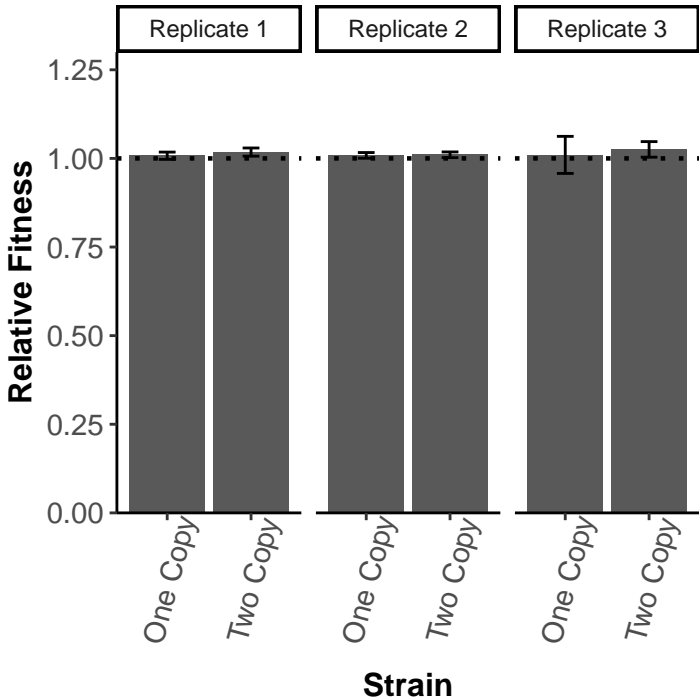

Supplement: S1 Fig — The fitness of strains carrying one (DGY500) or two copies (DGY1315) of a constitutively expressed mCitrine gene was assayed. Fluorescent strains were cocultured with the nonfluorescent, unevolved reference strain (FY4). We performed three independent competitive fitness assays in glutamine-limited chemostats using the same conditions as evolution experiments. No significant fitness defect was observed for either strain, indicating that constitutive expression of one or two copies of the fluorescent gene does not confer a fitness cost in these conditions. Error bars are 95% confidence intervals. Data and code used to generate this figure can be accessed in OSF: https://osf.io/fxhze/. CNV, copy number variant. (PDF) [file pbio.3000069.s004.pdf]

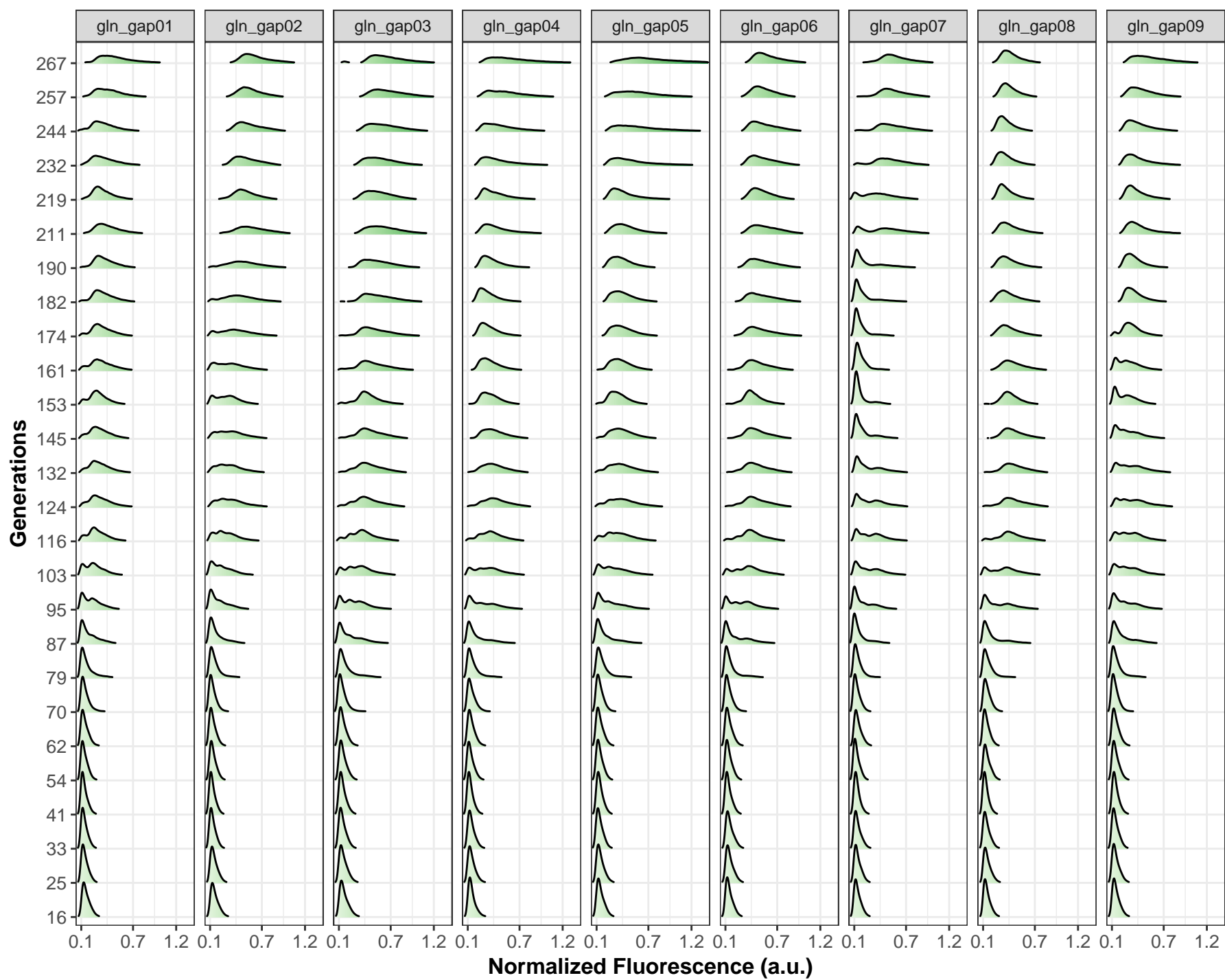

Supplement: S2 Fig — Distributions of single-cell fluorescence over time for all glutamine-limited experimental populations. Fluorescent signal is normalized by forward scatter, which varies as a function of cell size. Each distribution is based on 100,000 single cell measurements. Data and code used to generate this figure can be accessed in OSF: https://osf.io/fxhze/. CNV, copy number variant. (PDF) [file pbio.3000069.s005.pdf]

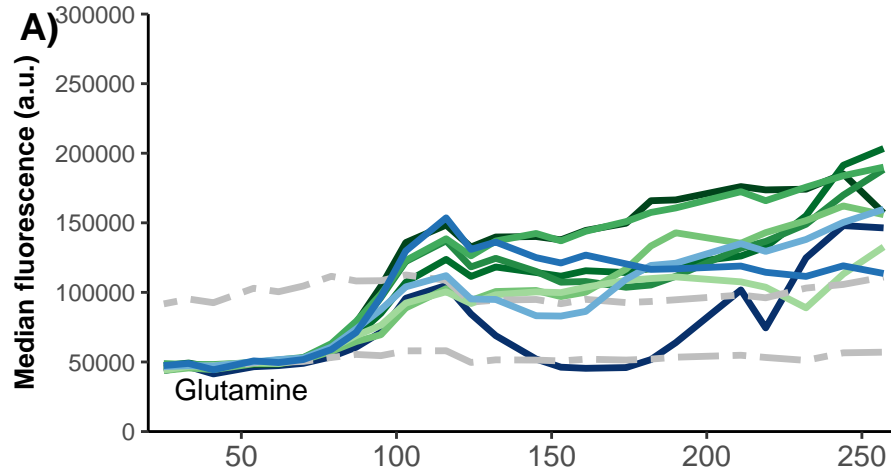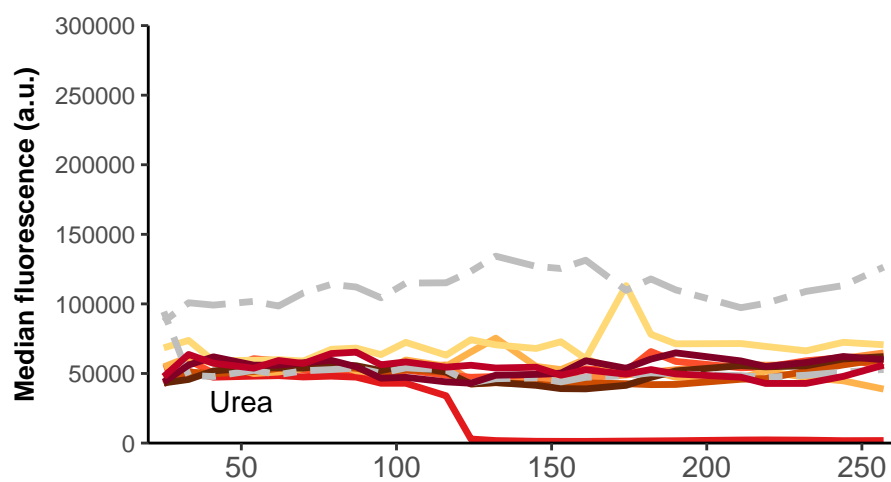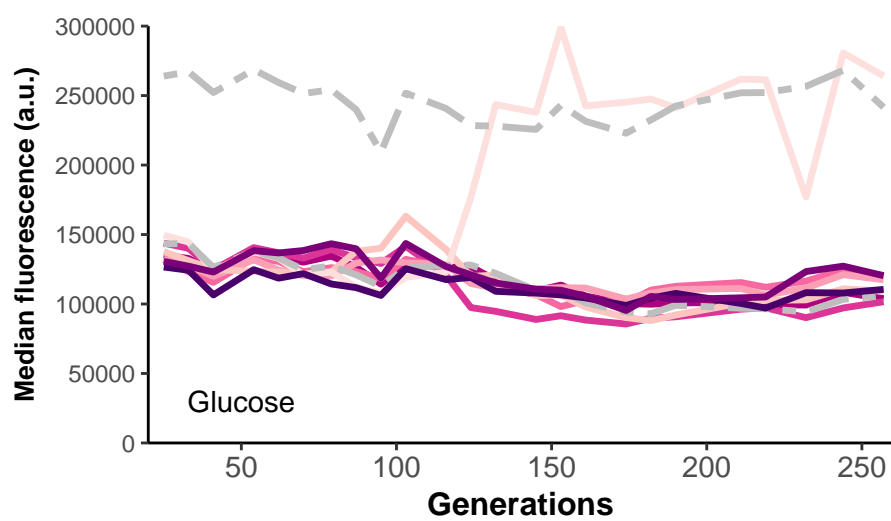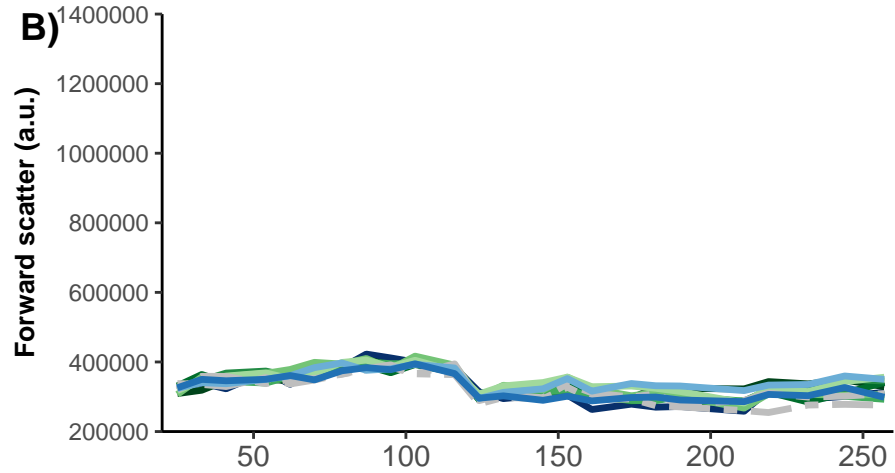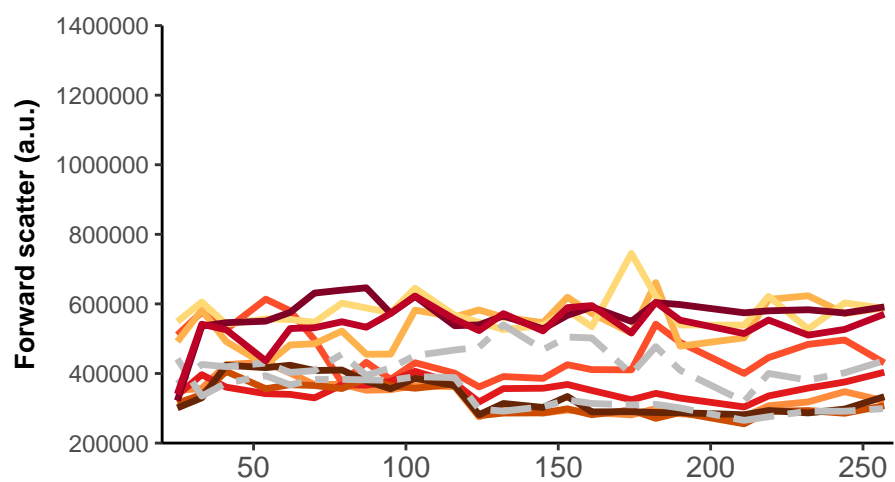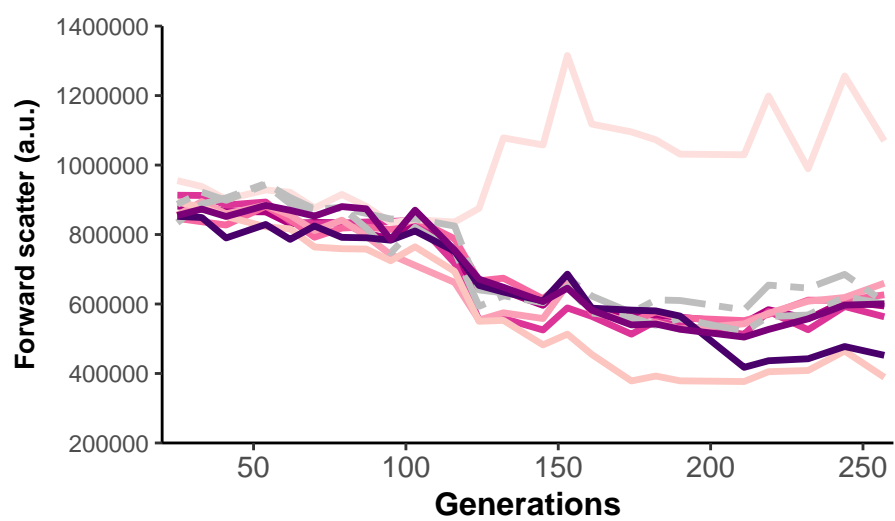

Supplement: S3 Fig — Dashed gray lines represent one- and two-copy control populations. (A) Median unnormalized fluorescence across time for all evolving populations. (B) Median forward scatter over time for all populations. One glucose-limited population (pink) developed a bud separation defect, resulting in a cell aggregation phenotype and large forward scatter and fluorescence measurements. Normalizing by forward scatter accounts for this issue and other changes in overall cell physiology during the evolution experiments (see Fig 2B). Data and code used to generate this figure can be accessed in OSF: https://osf.io/fxhze/. CNV, copy number variant. (PDF) [file pbio.3000069.s006.pdf]

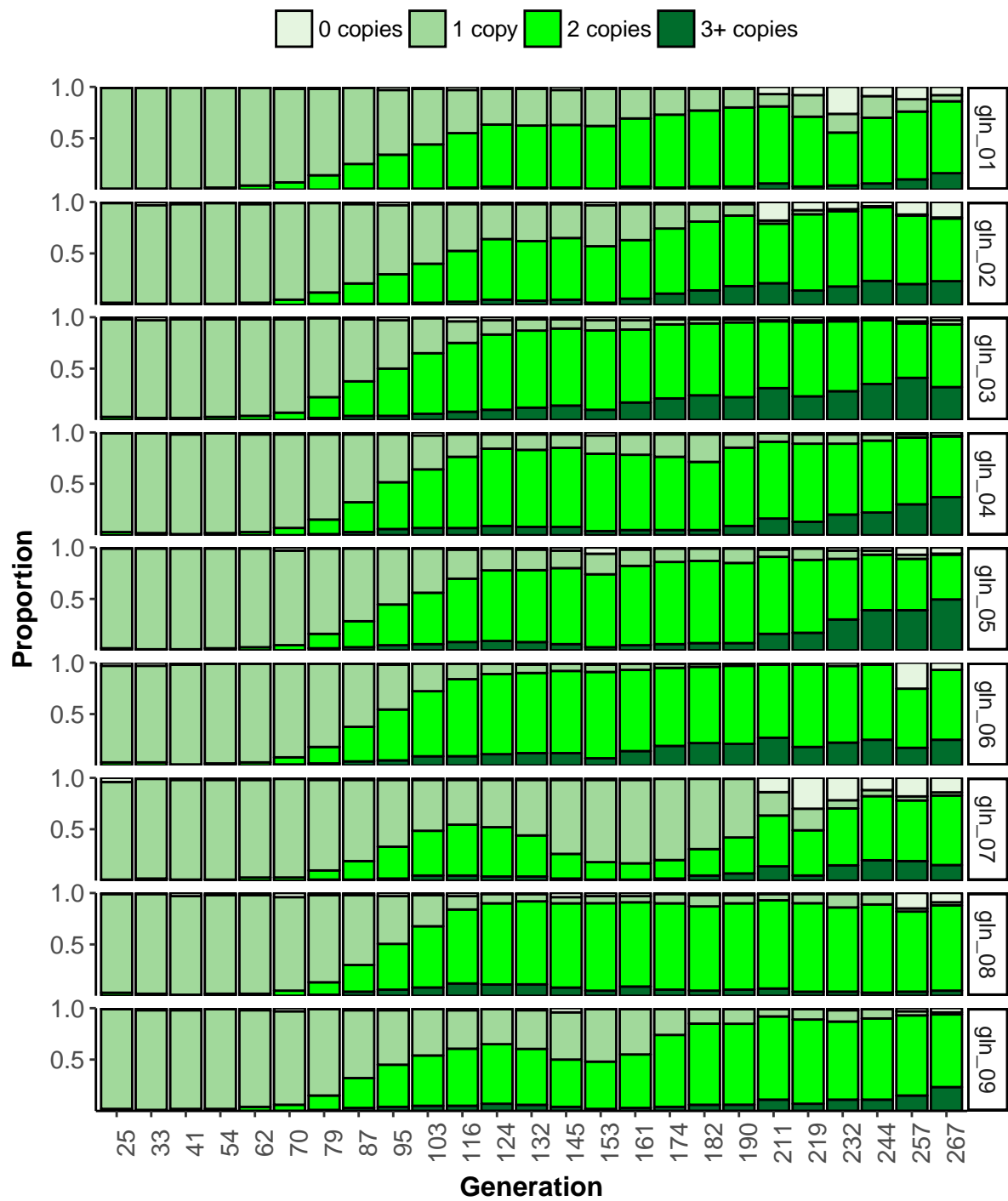

Supplement: S4 Fig — The proportion of cells with zero, one, two, and three or more copies of GAP1 in each glutamine-limited experimental population. Proportions were calculated after generating gating criteria based on one- and two-copy control populations. Data and code used to generate this figure can be accessed in OSF: https://osf.io/fxhze/. CNV, copy number variant. (PDF) [file pbio.3000069.s007.pdf]

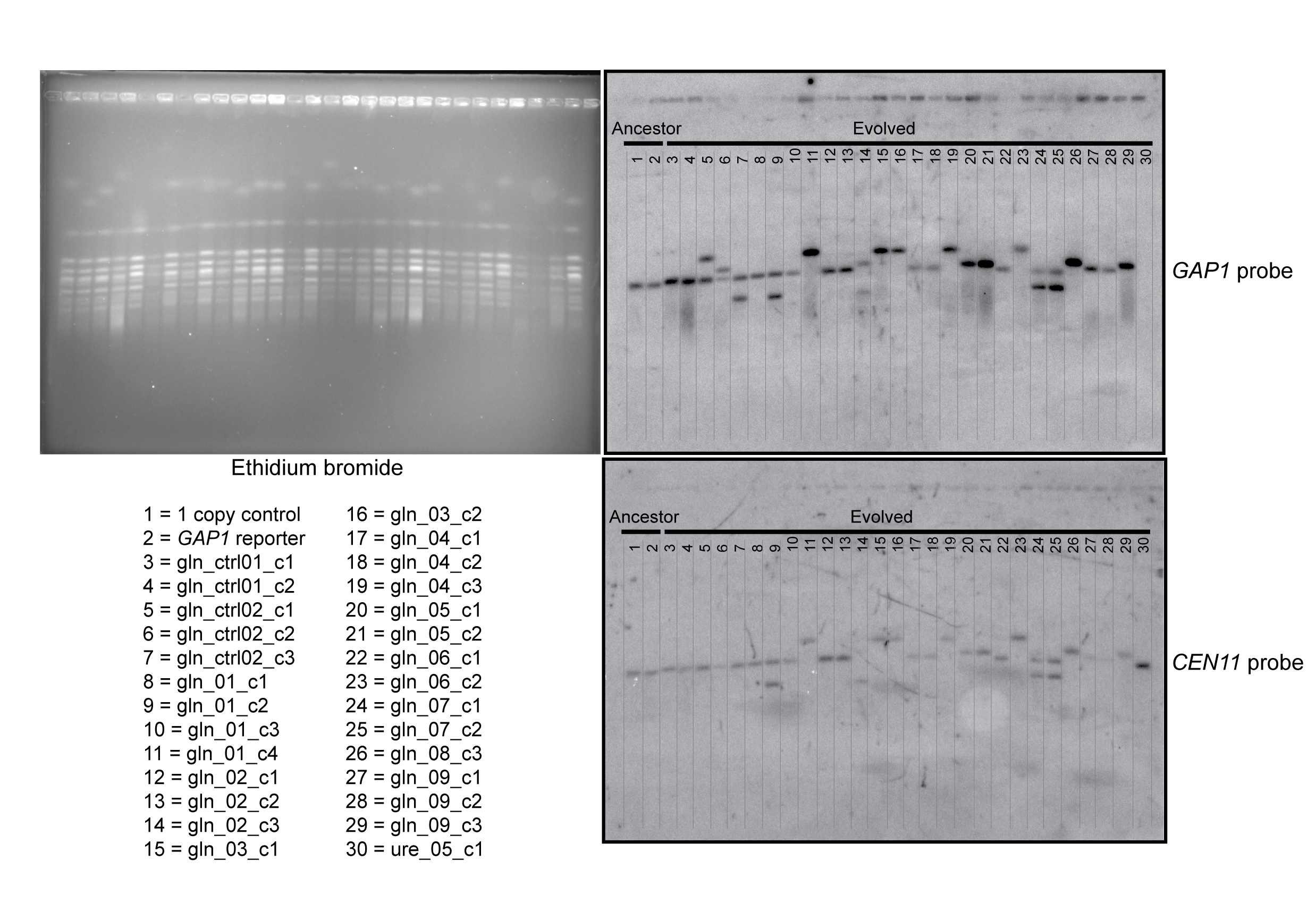

Supplement: S5 Fig — Analysis of ancestral and evolved clones. Whole chromosomes were visualized by ethidium bromide staining (left) and then probed for GAP1 and CEN11 (right). In the majority of cases, the CEN11 probe correlates with GAP1 probe signal, indicating that these GAP1 amplifications are located on Chromosome XI. Instances when the CEN11 and GAP1 probes do not correlate are indicative of nonreciprocal translocations. Duplication of CEN11 may indicate segmental aneuploidy. CNV, copy number variant. (TIF) [file pbio.3000069.s008.tif]

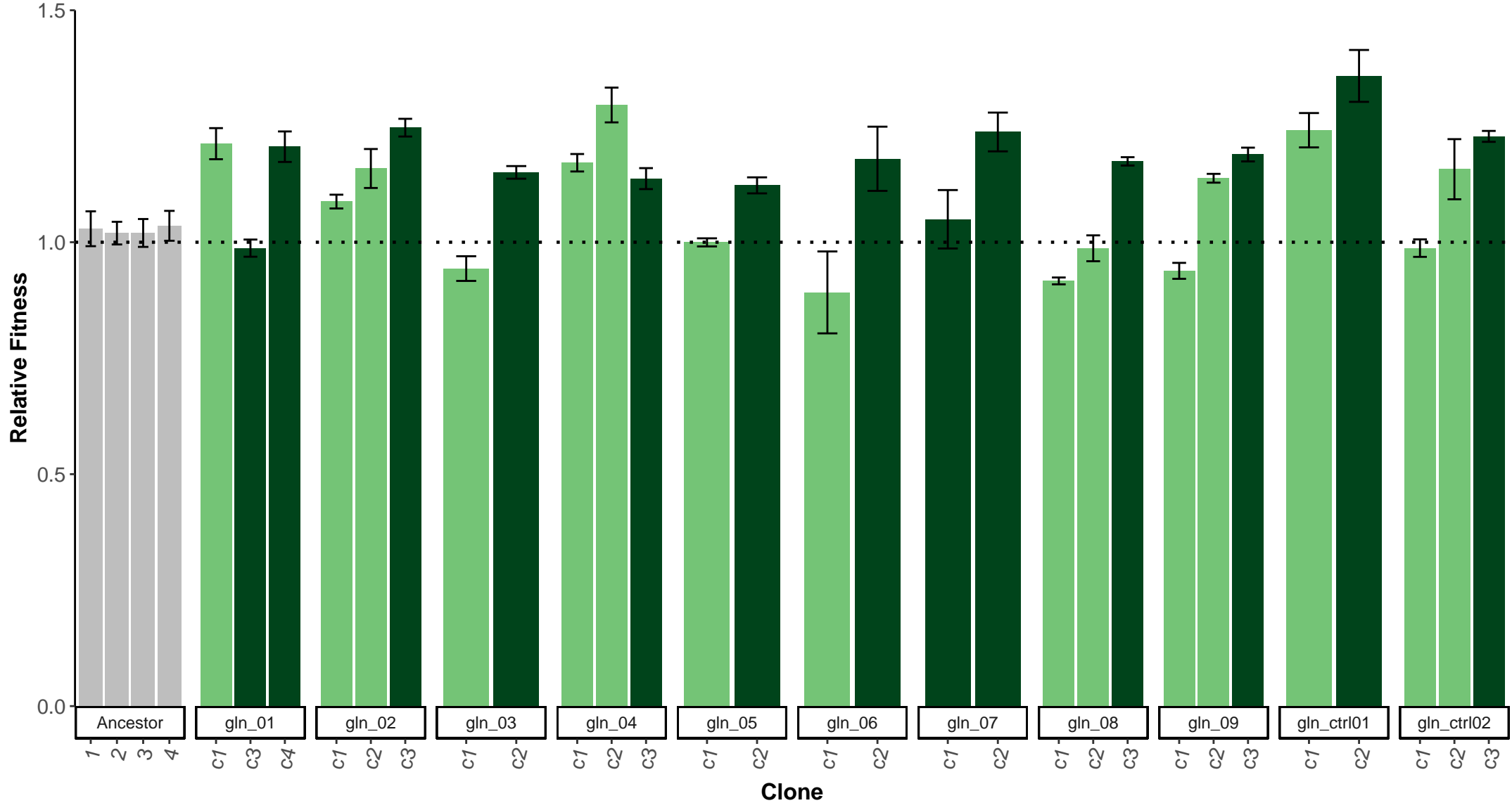

Supplement: S6 Fig — The fitness of evolved lineages containing GAP1 CNVs was determined by pairwise competition experiments with a nonfluorescent, unevolved reference strain (FY4) in glutamine-limited chemostats. The majority (18/28) of evolved CNV-containing lineages have significantly higher fitness (t test, Bonferroni-corrected p-value < 0.00156) than the ancestor. Decreased (2/28) or insignificant fitness differences (8/28) may reflect context-specific fitness effects of GAP1 CNV-containing lineages. Error bars are 95% confidence intervals. Data and code used to generate this figure can be accessed in OSF: https://osf.io/fxhze/. CNV, copy number variant. (PDF) [file pbio.3000069.s009.pdf]

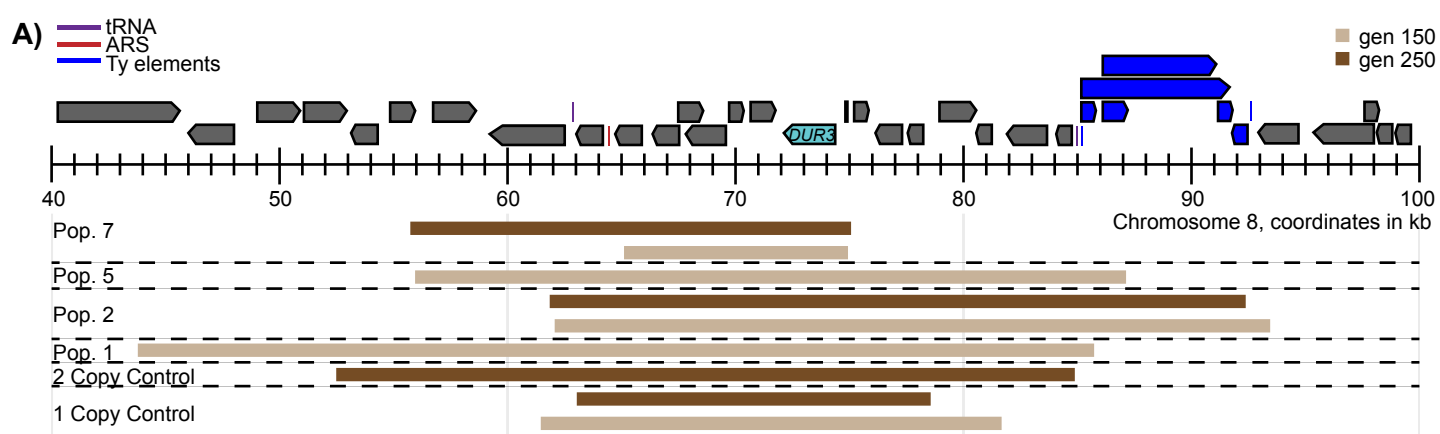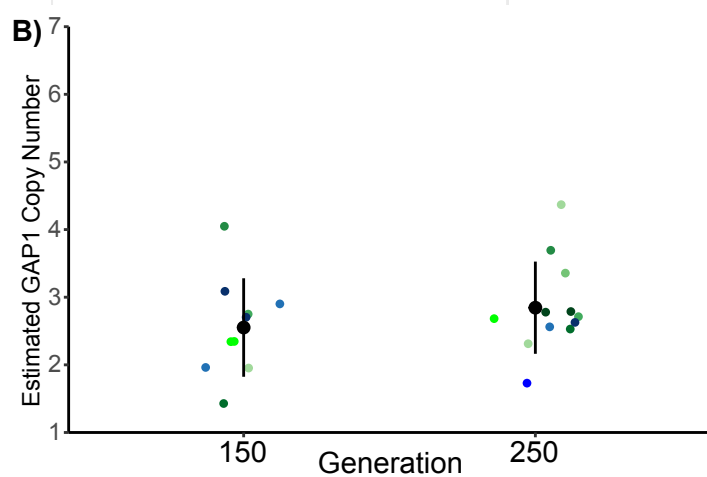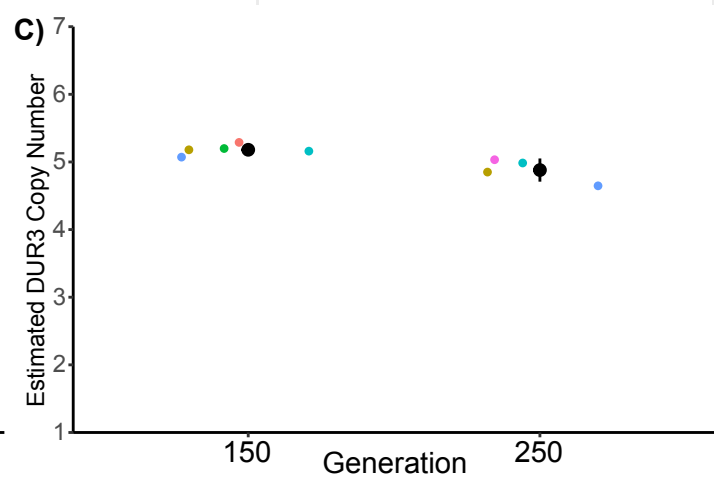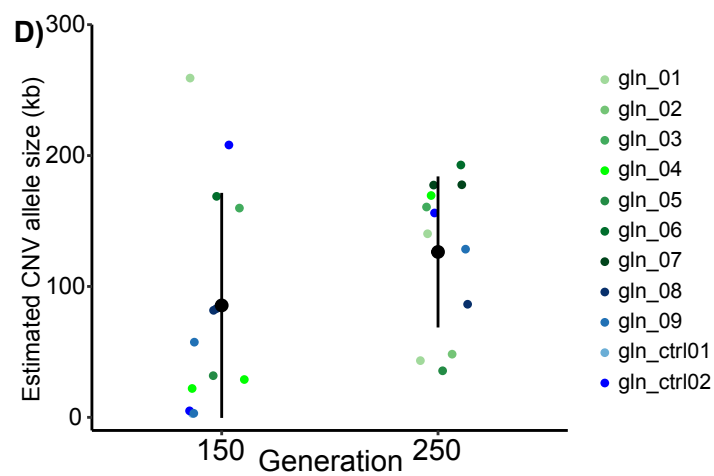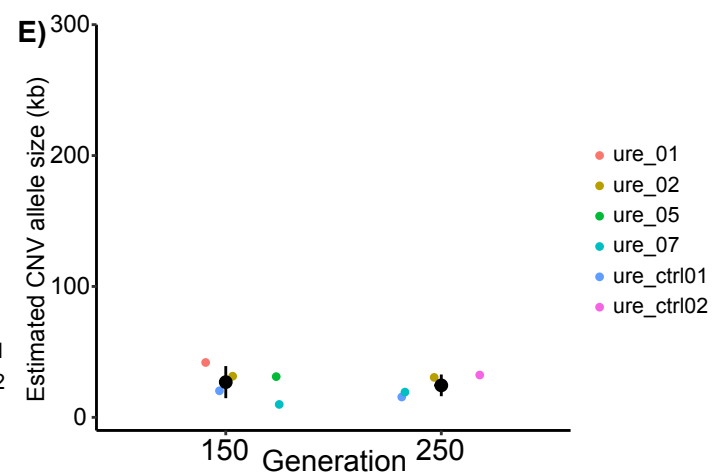

Supplement: S7 Fig — (A) A schematic illustrating the genomic context and estimated breakpoints for clones containing DUR3 CNVs isolated from urea-limited chemostats at generation 150 and generation 250. Breakpoint boundaries were estimated using a read depth–based approach. Compared to (B) clones isolated from glutamine-limited chemostats containing GAP1 CNVs, (C) clones isolated from urea-limited chemostats have a significantly higher copy number (t test p-value < 0.01). (D) GAP1 CNV alleles are significantly larger than (E) DUR3 CNV alleles (t test p-value < 0.01). Data and code used to generate this figure can be accessed in OSF: https://osf.io/fxhze/. ARS, autonomously replicating sequence; CNV, copy number variant. (PDF) [file pbio.3000069.s010.pdf]

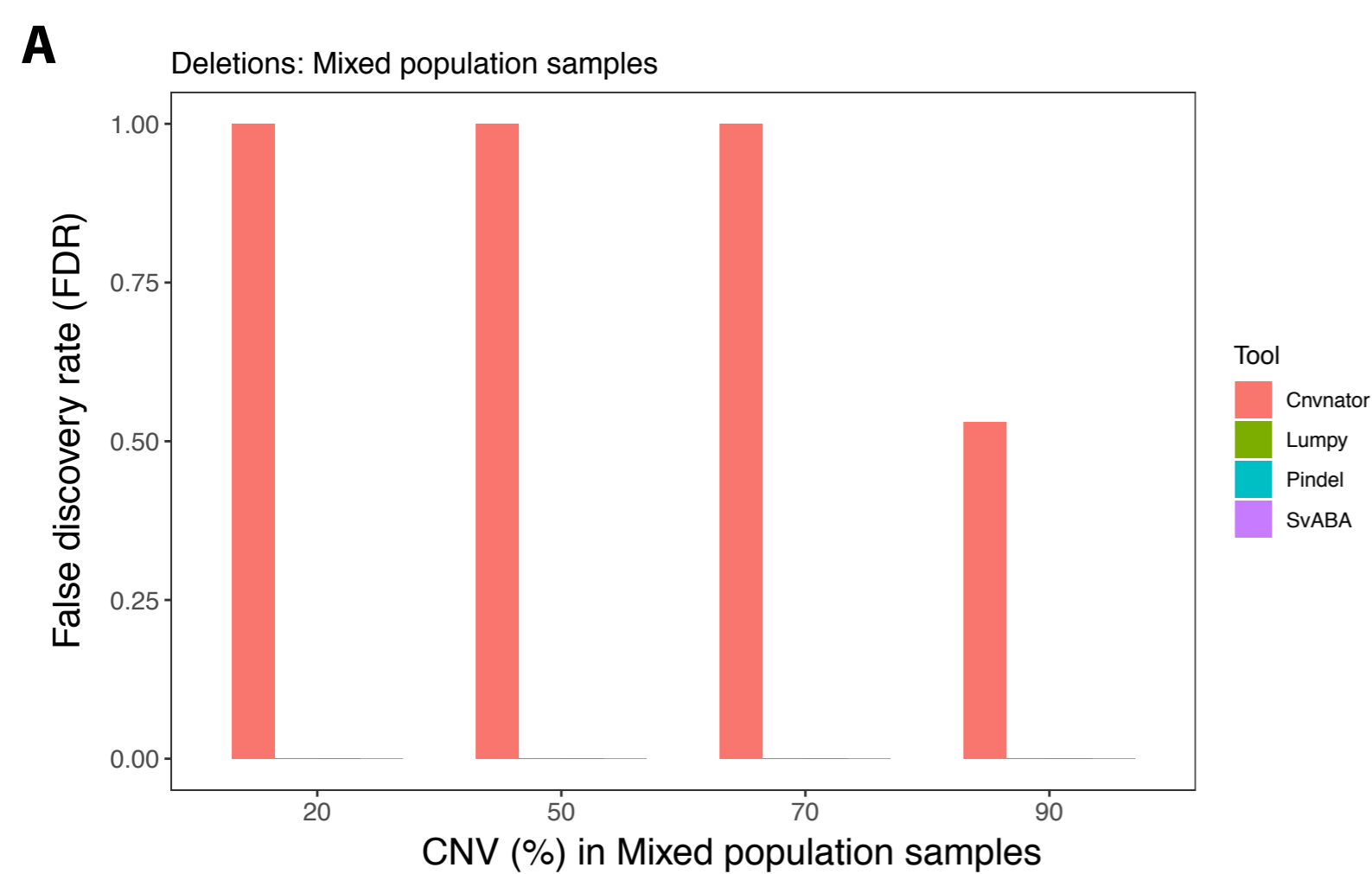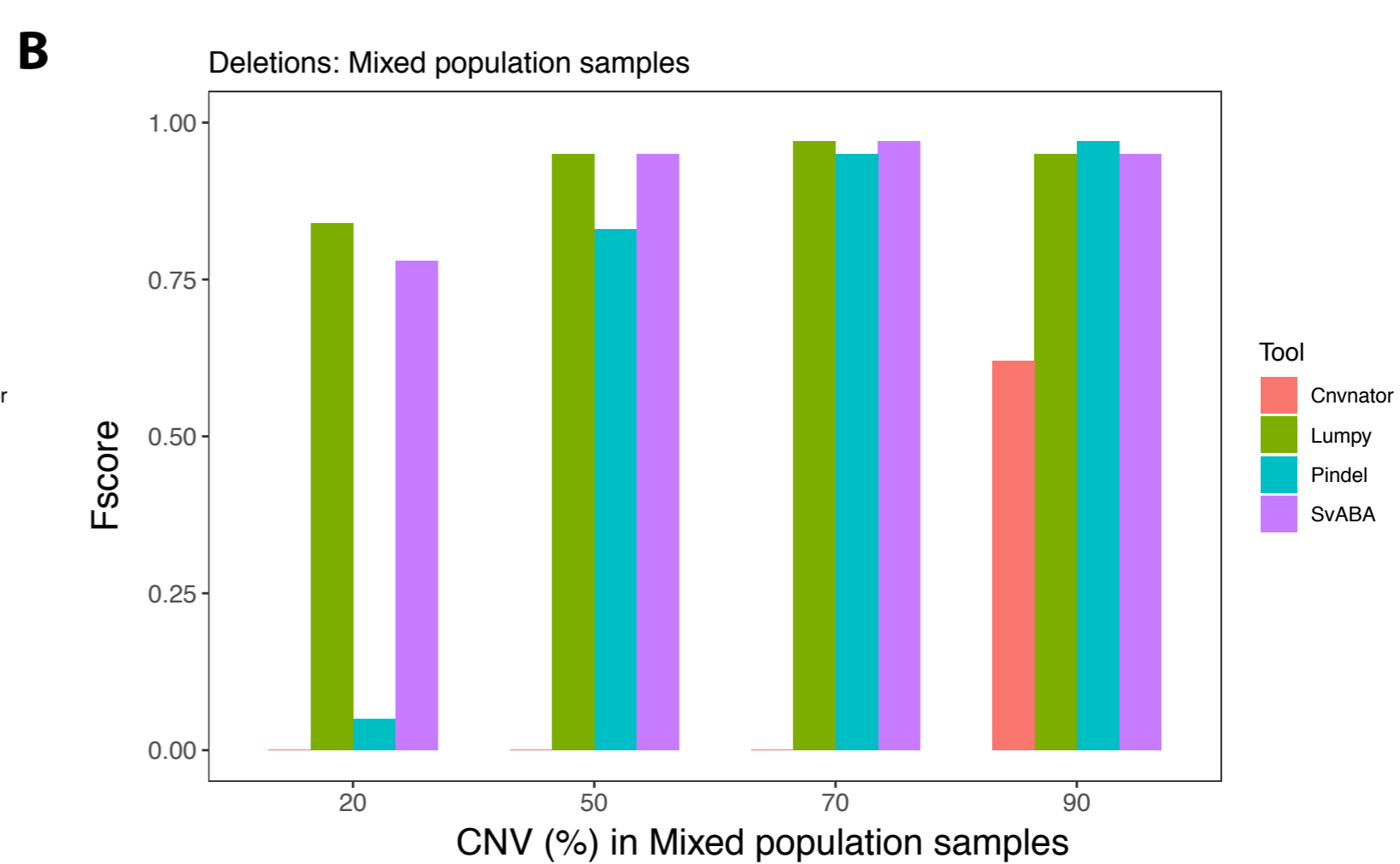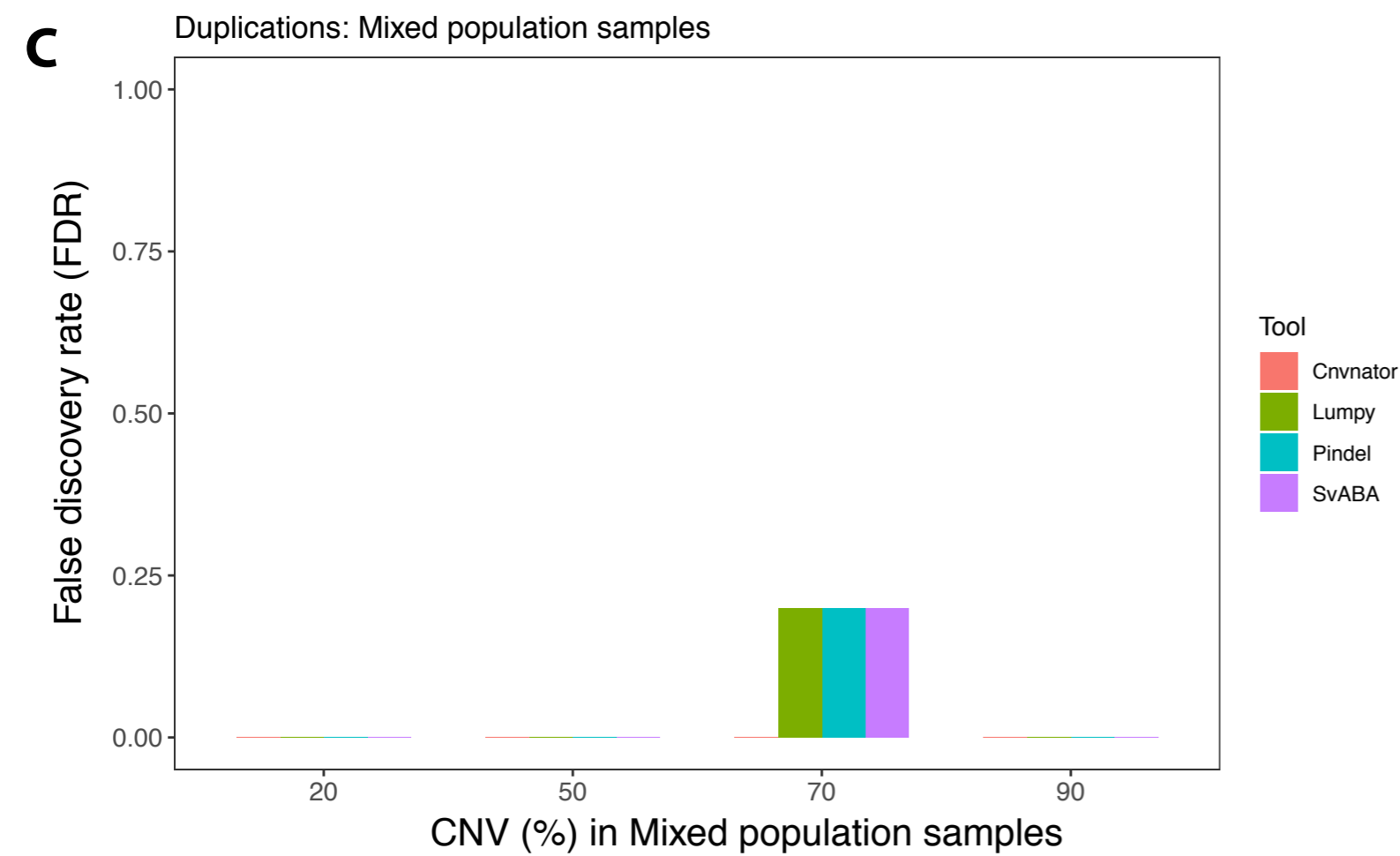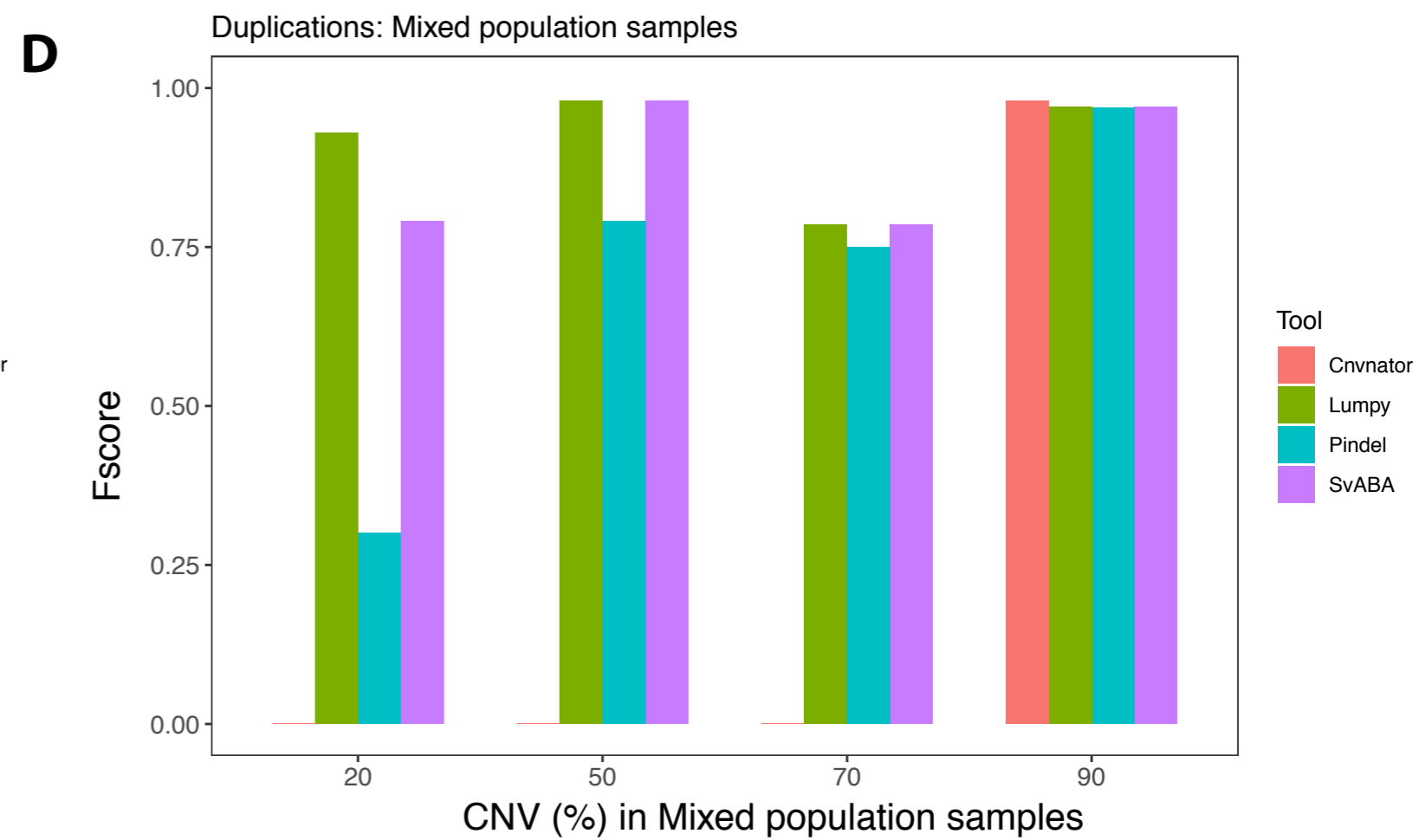

Supplement: S9 Fig — We simulated heterogeneous populations containing CNVs at varying frequencies and assessed algorithm performance. Most algorithms perform reasonably well when CNVs are present at 50% or higher in the population. Data and code used to generate this figure can be accessed in OSF: https://osf.io/fxhze/. CNV, copy number variant. (PDF) [file pbio.3000069.s012.pdf]

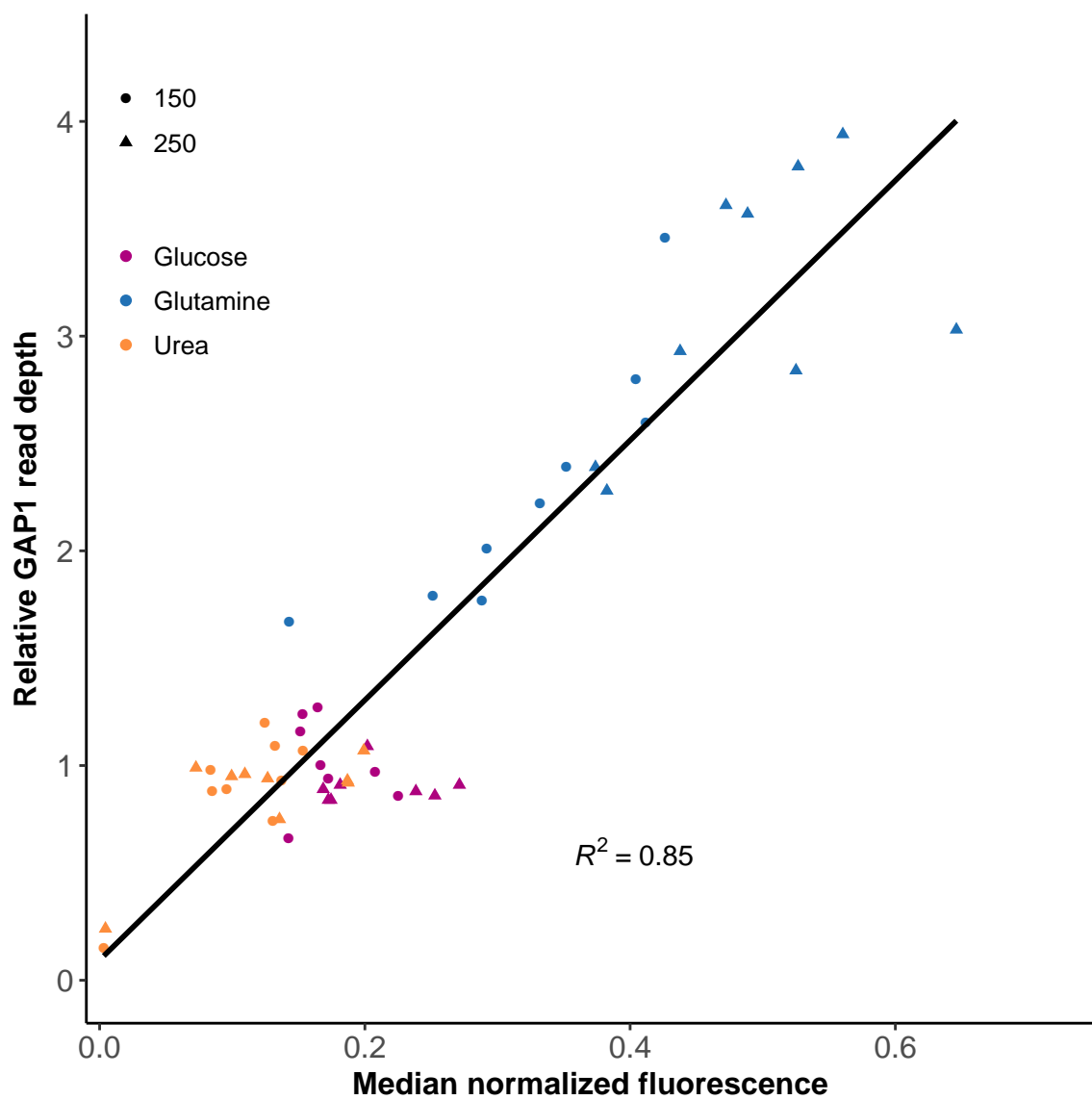

Supplement: S10 Fig — Relative depth at the GAP1 locus, calculated from whole-genome sequencing data, is strongly correlated with the median normalized fluorescence of the GAP1 CNV reporter in populations. Glutamine-limited populations measured at generation 250 tend to have higher fluorescence and higher relative read depth at the GAP1 locus than at generation 150. Data and code used to generate this figure can be accessed in OSF: https://osf.io/fxhze/. CNV, copy number variant. (PDF) [file pbio.3000069.s013.pdf]

A)

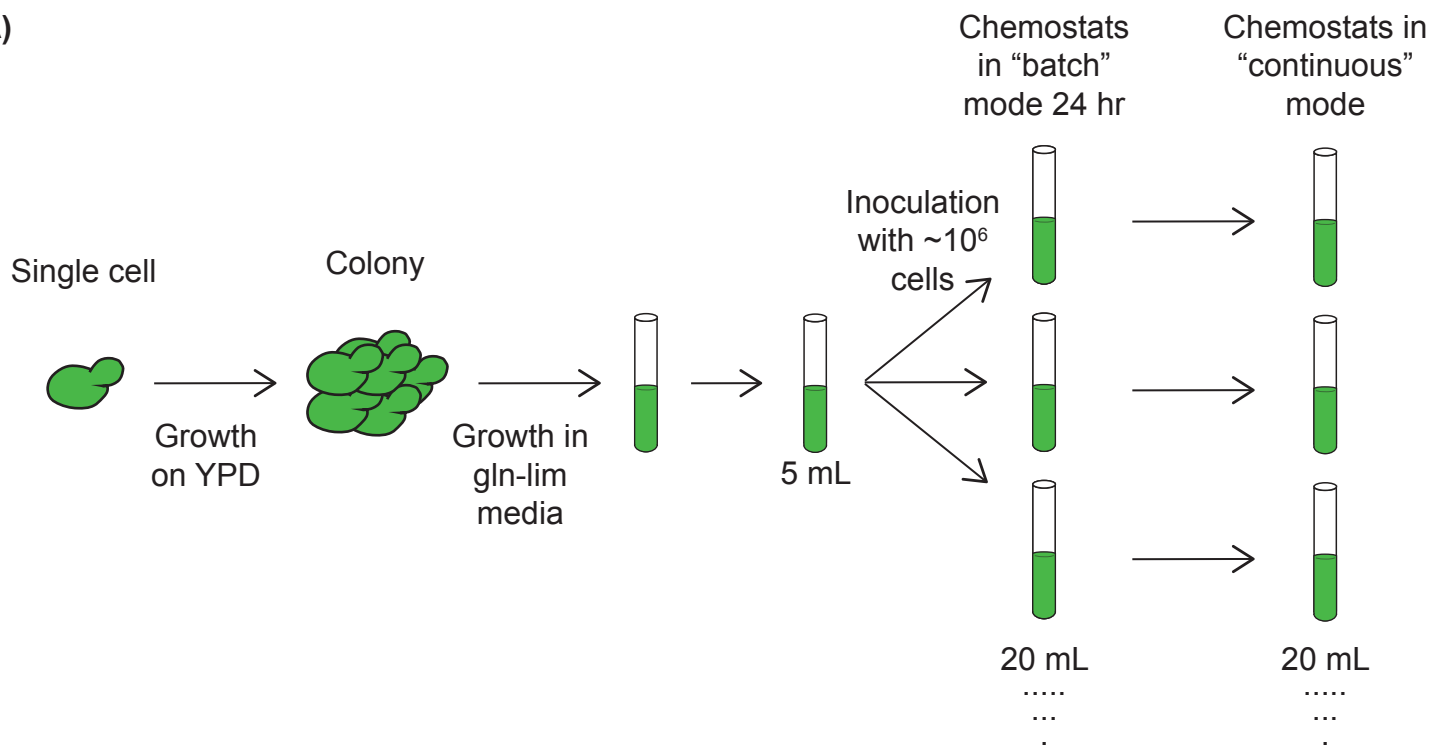

B)

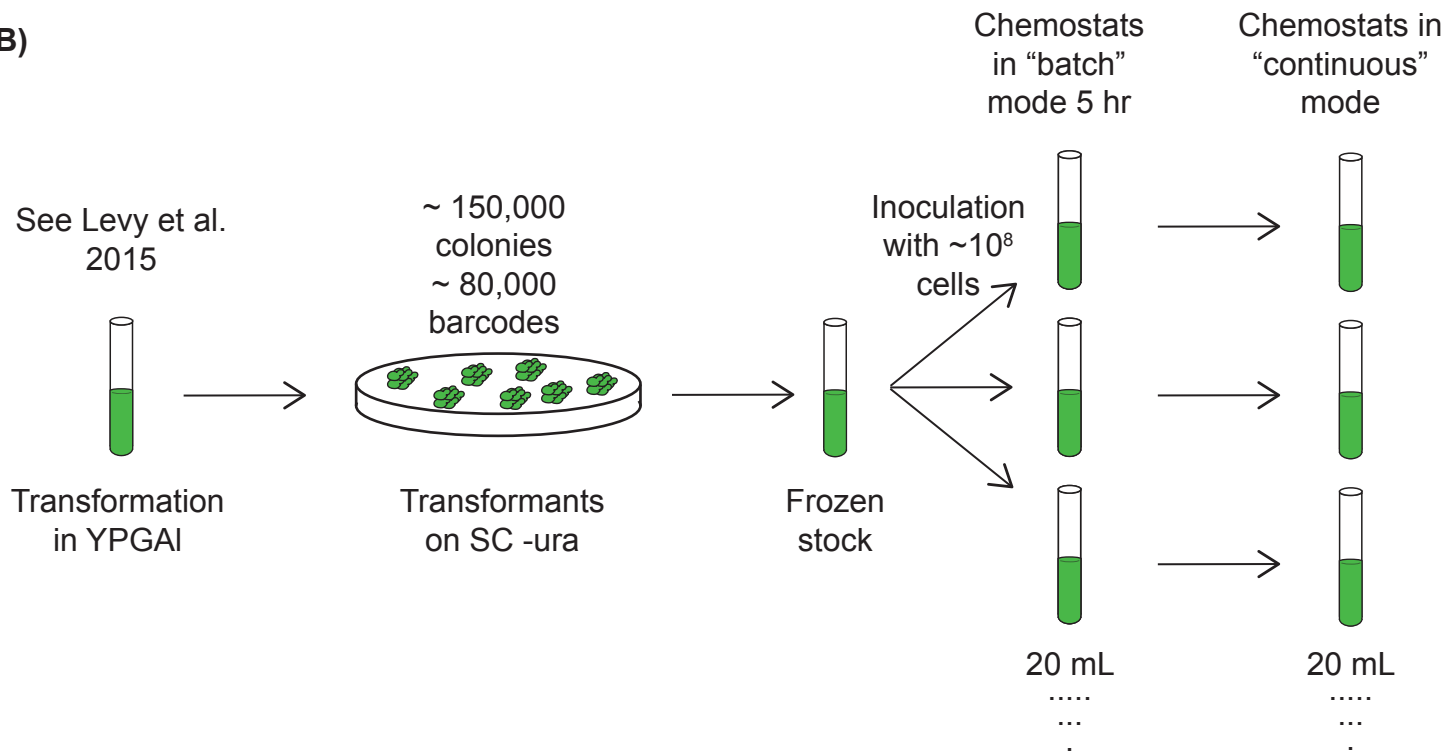

Supplement: S11 Fig — All independent populations share a common history prior to founding of individual populations. The prehistory of experiments using the GAP1 CNV reporter (A) differ with respect to the size of the founding population in experiments using a lineage-tracking library (B). Any variation that is introduced prior to founding of individual populations may contribute to the evolution of all populations. Variation that is introduced after separation into individual populations contributes to evolutionary outcomes in that population only. CNV, copy number variant; Gln-lim, glutamine limited; YPD, yeast extract-peptone-dextrose (rich media); YPGAL, yeast extract-peptone-galactose. (PDF) [file pbio.3000069.s014.pdf]

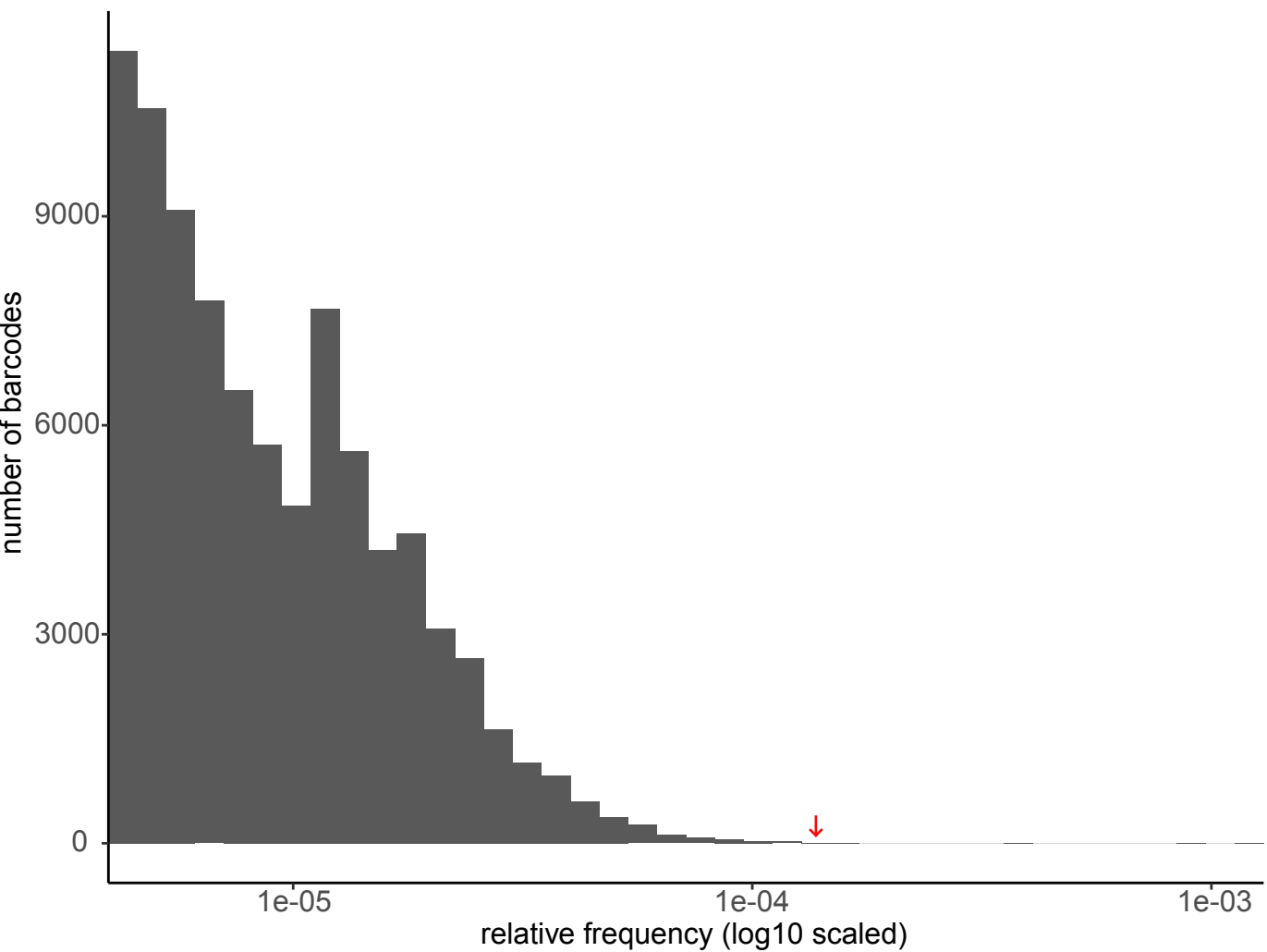

Supplement: S12 Fig — We determined the distribution of read counts supporting each unique barcode in the ancestral population, after filtering out low-confidence clusters. The relative frequencies of barcodes vary by over an order of magnitude, and we observe a long tail with a few barcodes significantly overrepresented in the ancestral population. The red arrow indicates an overrepresented barcode in the ancestral population that was identified in the CNV subpopulation in both independent barcoded evolution experiments (indicated in purple in Fig 5B). This distribution is consistent with that found in other barcode lineage-tracking experiments [68]. Data and code used to generate this figure can be accessed in OSF: https://osf.io/fxhze/. CNV, copy number variant. (PDF) [file pbio.3000069.s015.pdf]

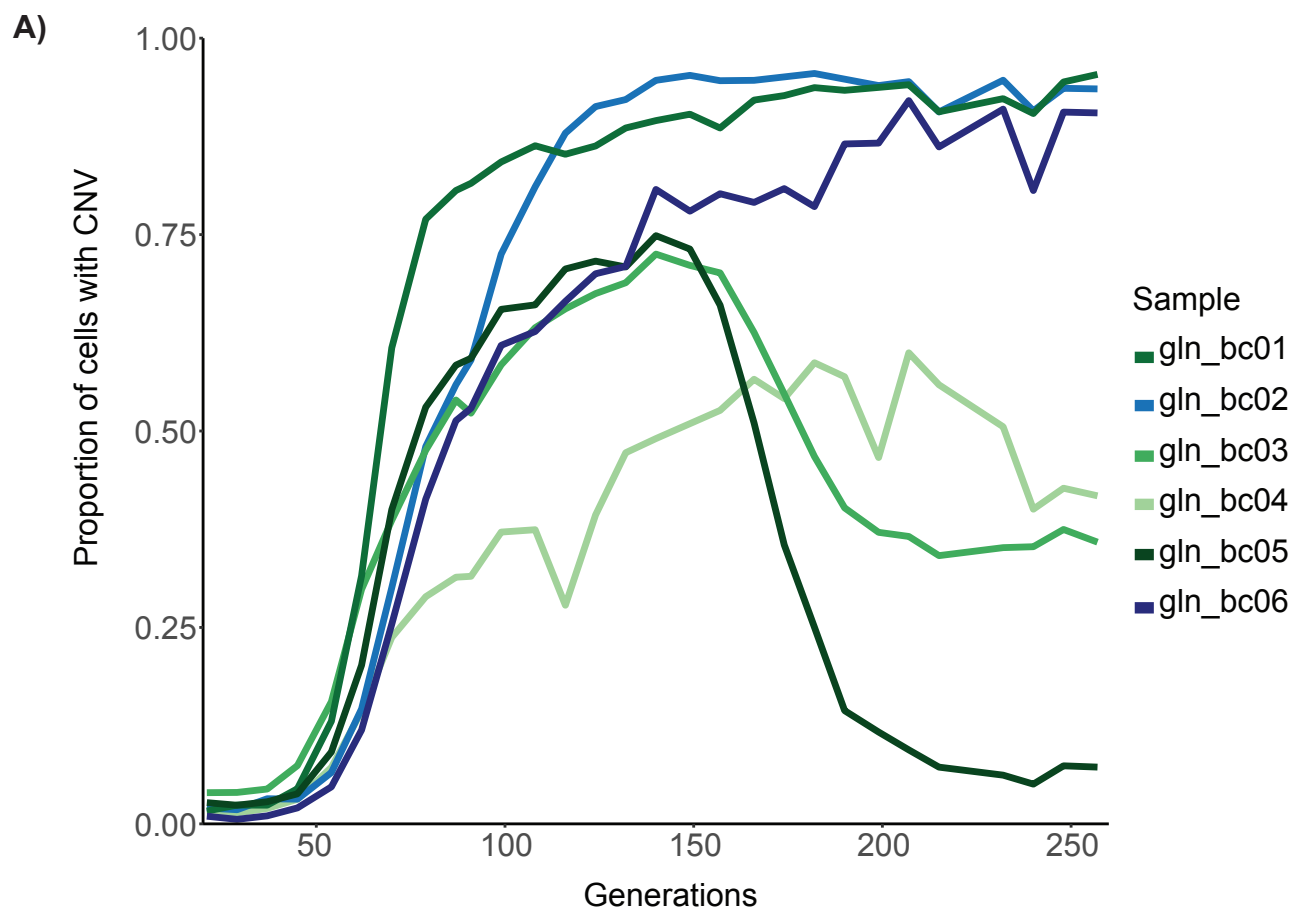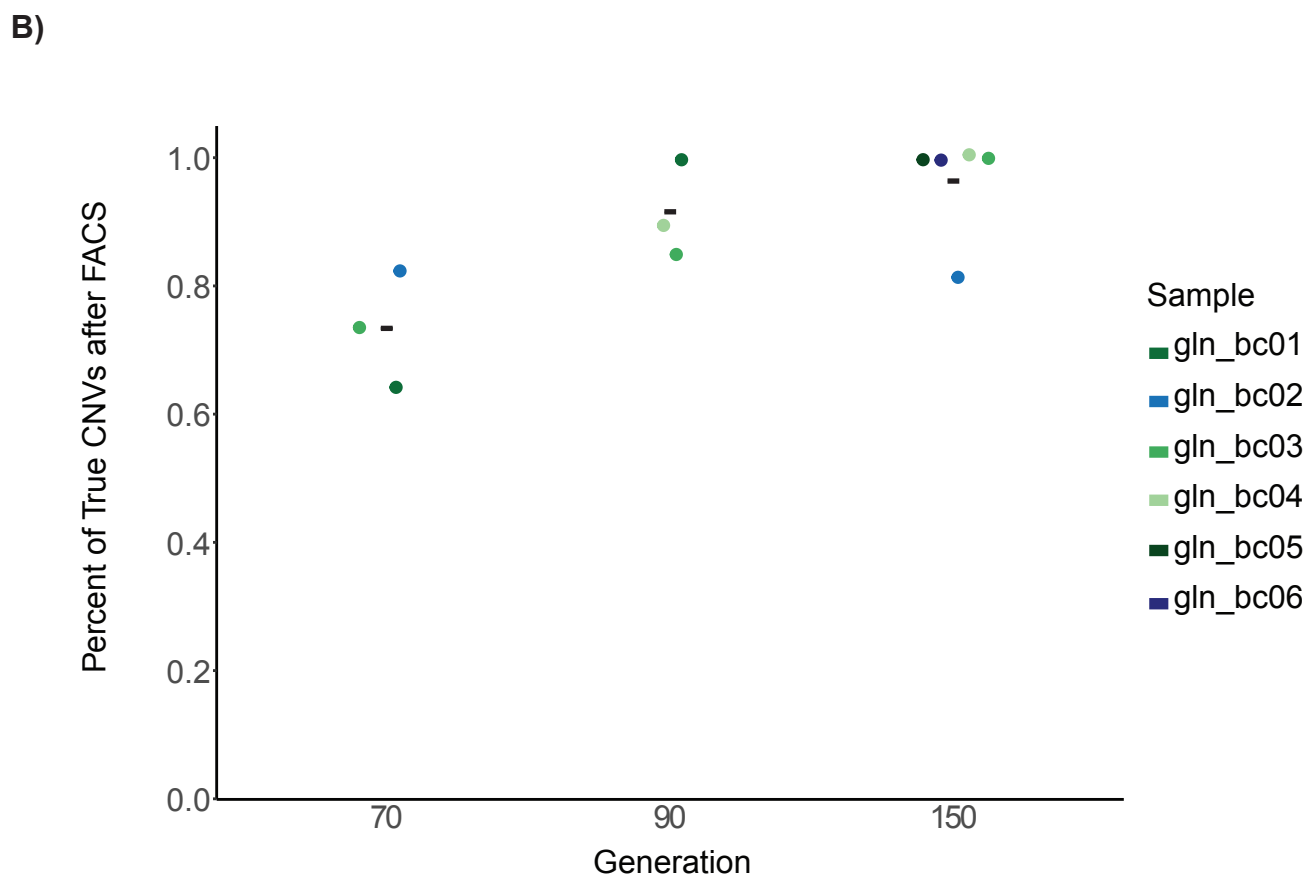

Supplement: S13 Fig — (A) GAP1 CNV dynamics in barcoded populations assayed using a CNV reporter. (B) Estimation of true positive rate of CNV isolation by FACS at generations 70, 90, and 150. CNV subpopulations were isolated by FACS at each time point and clones isolated by plating for single colonies. The percentage of cells containing a CNV in the fractionated subpopulation was estimated using at least 25 clones. A one-copy control strain was used to define gates. Data and code used to generate this figure can be accessed in OSF: https://osf.io/fxhze/. CNV, copy number variant; FACS, fluorescence-activated cell sorting. (PDF) [file pbio.3000069.s016.pdf]
