# Supplementary material for: Single-cell copy number variant detection reveals the dynamics and diversity of adaptation
Source: PLoS Biol. 2018 Dec 18;16(12):e3000069. doi: 10.1371/journal.pbio.3000069 (PMC6298651; doi:10.1371/journal.pbio.3000069)
Supplement: S8 Fig — We simulated CNVs in the yeast genome at different average sequencing depths to assess the performance of CNVnator, LUMPY, Pindel, and SvABA. Algorithm performance was evaluated using and F-score. We find that with increased read depth, (A) the FDR increases for deletion detection, but (B) overall performance improves for all algorithms as determined by F-score. Conversely, for duplication detection, (C) the false positive rate is not increased with increasing read depth, and (D) overall performance improves with increased read depth. Data and code used to generate this figure can be accessed in OSF: https://osf.io/fxhze/. CNV, copy number variant; FDR, false discovery rate. (PDF) [file pbio.3000069.s011.pdf]

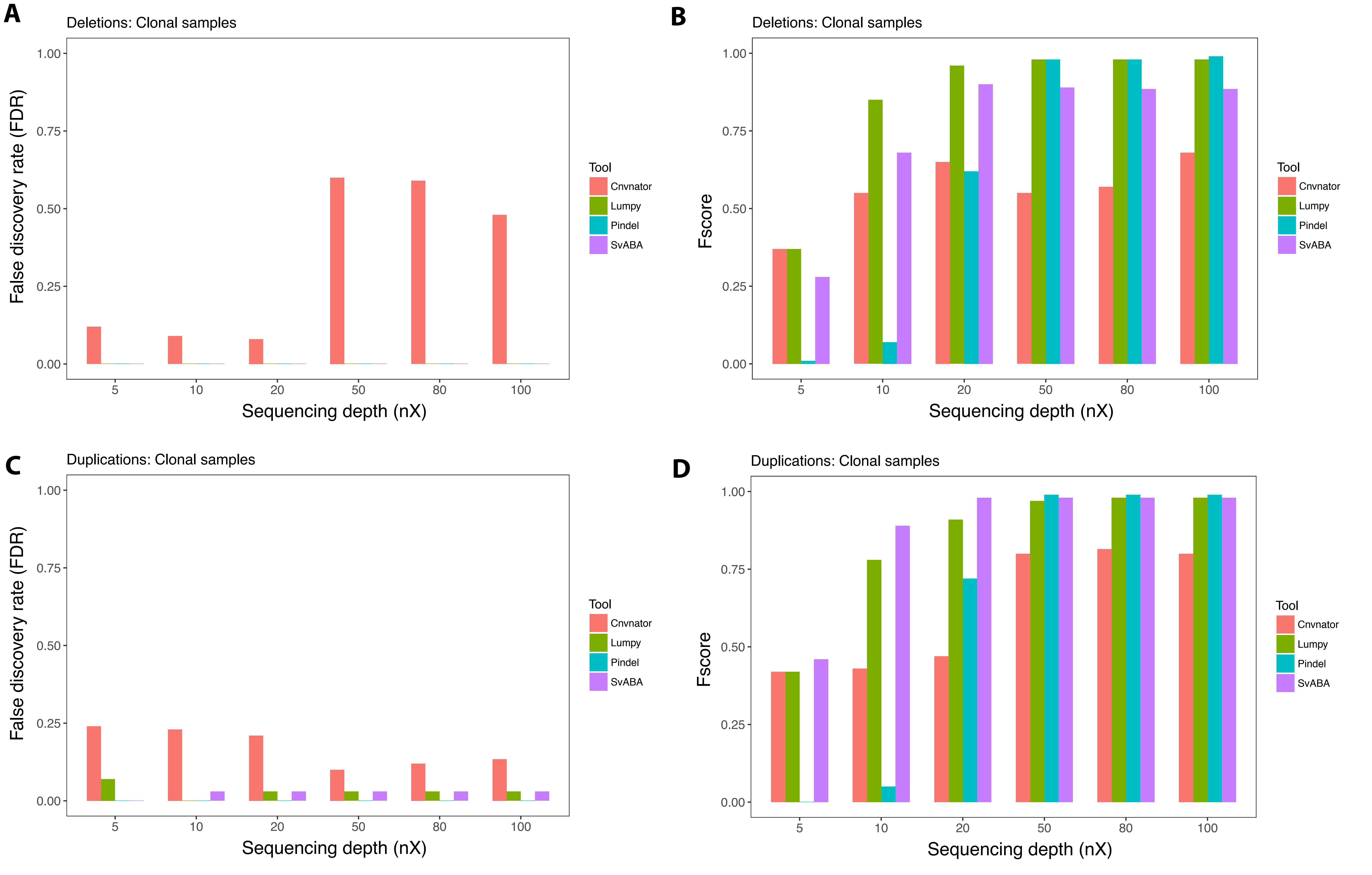
A
Deletions: Clonal samples
False discovery rate (FDR)
1.00
0.75
0.50
0.25
0.00
5
10
20
50
80
100
Sequencing depth (nX)
Tool
Cnvnator
Lumpy
Pindel
SvABA
B
Deletions: Clonal samples
Fscore
Sequencing depth (nX)
C
Duplications: Clonal samples
False discovery rate (FDR)
Sequencing depth (nX)
D
Duplications: Clonal samples
Fscore
Sequencing depth (nX)
