# Supplementary material for: Single-cell copy number variant detection reveals the dynamics and diversity of adaptation
Source: PLoS Biol. 2018 Dec 18;16(12):e3000069. doi: 10.1371/journal.pbio.3000069 (PMC6298651; doi:10.1371/journal.pbio.3000069)
Supplement: S14 Fig — Reports for CNV subpopulation isolation at generation 70 (A–E), 90 (F–J), 150 (K–O), and 270 (P–T). Gates were drawn based on zero-, one-, and two-copy control populations, and cells were isolated from the P4 population. CNV, copy number variant; FACS, fluorescence-activated cell sorting. (PDF) [file pbio.3000069.s017.pdf]

A)

BD FACSDiva 8.0.2

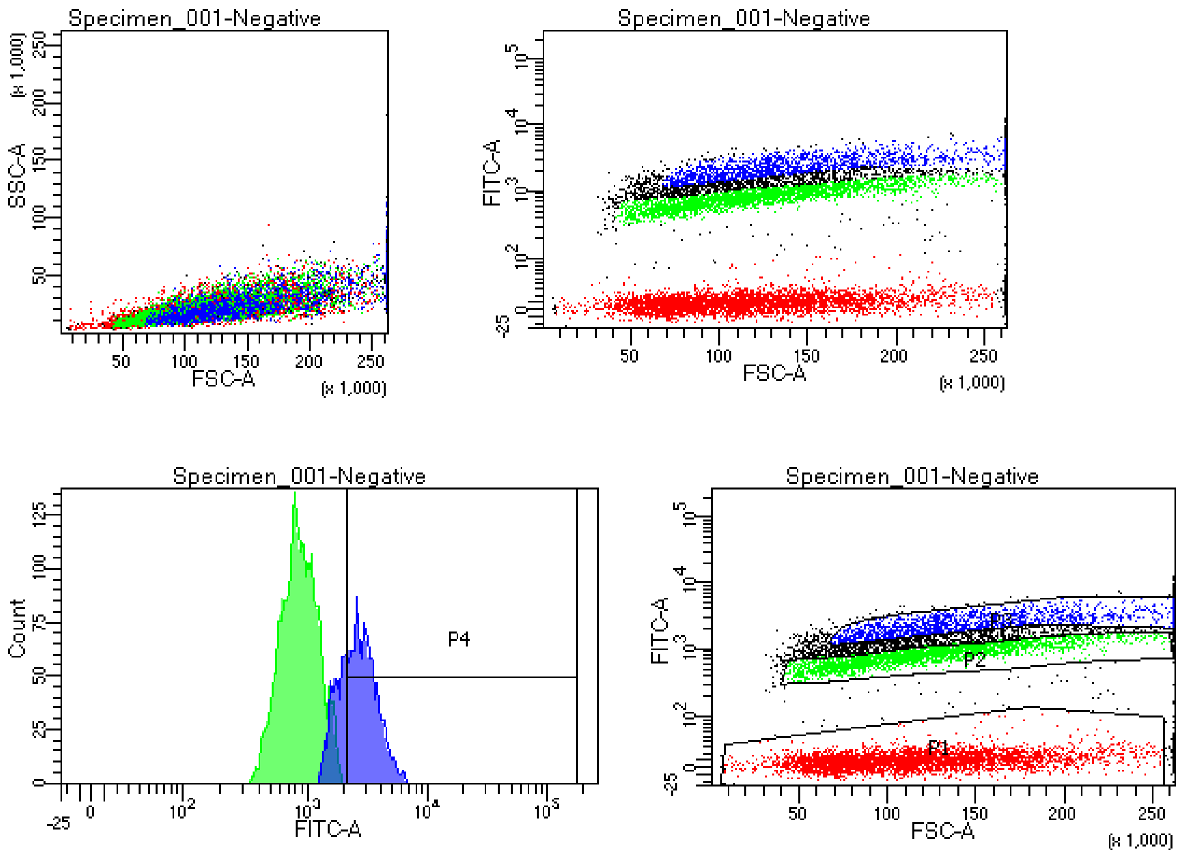

Experiment Name:	Grace 2017-08-31
Specimen Name:	Specimen_001
Tube Name:	Negative
Record Date:	Aug 31, 2017 1:45:50 PM
$OP:	Administrator

Population	#Events	%Parent
All Events	10,000	####
P1	3,937	39.4
P2	2,680	26.8
P3	1,656	16.6
P4	1,162	11.6

B)

BD FACSDiva 8.0.2

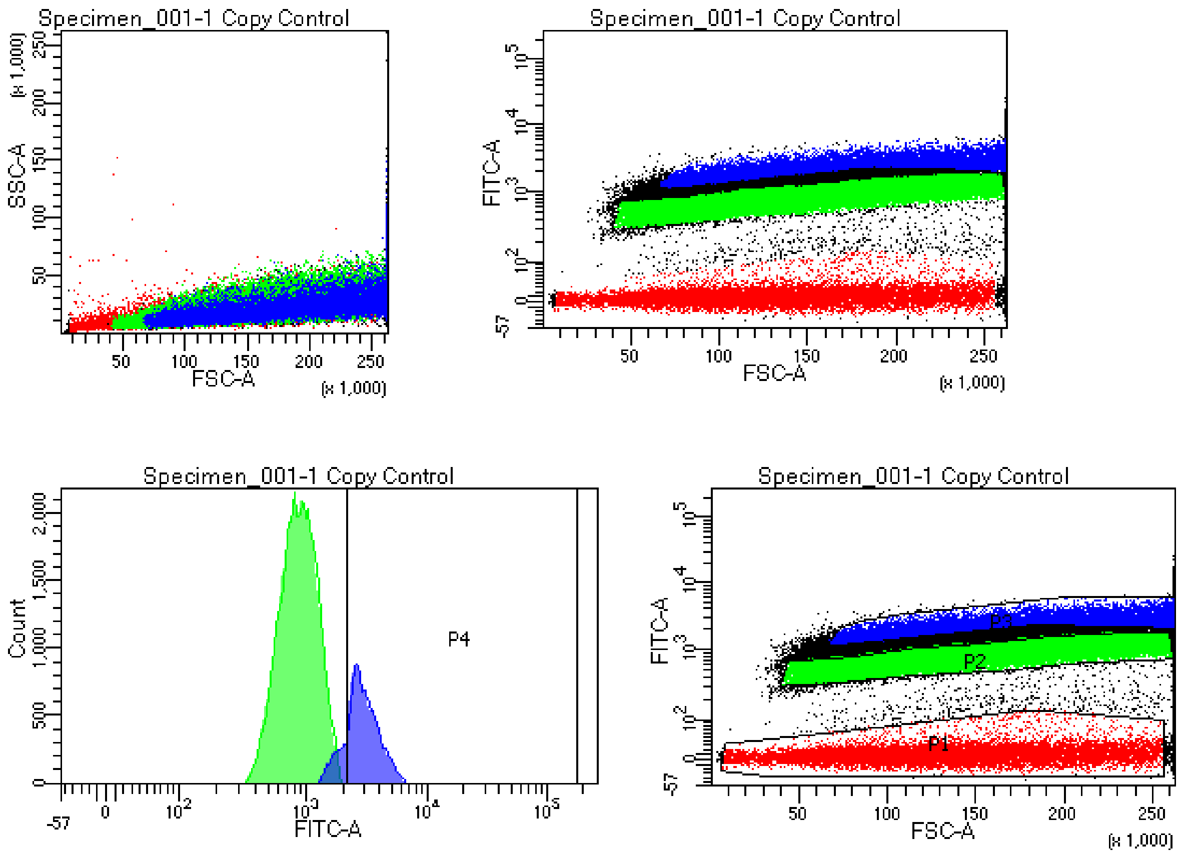

Experiment Name:	Grace 2017-08-31
Specimen Name:	Specimen_001
Tube Name:	1 Copy Control
Record Date:	Aug 31, 2017 1:49:35 PM
$OP:	Administrator

Population	#Events	%Parent
All Events	100,000	####
P1	12,457	12.5
P2	49,996	50.0
P3	15,002	15.0
P4	12,832	12.8

C)

BD FACSDiva 8.0.2

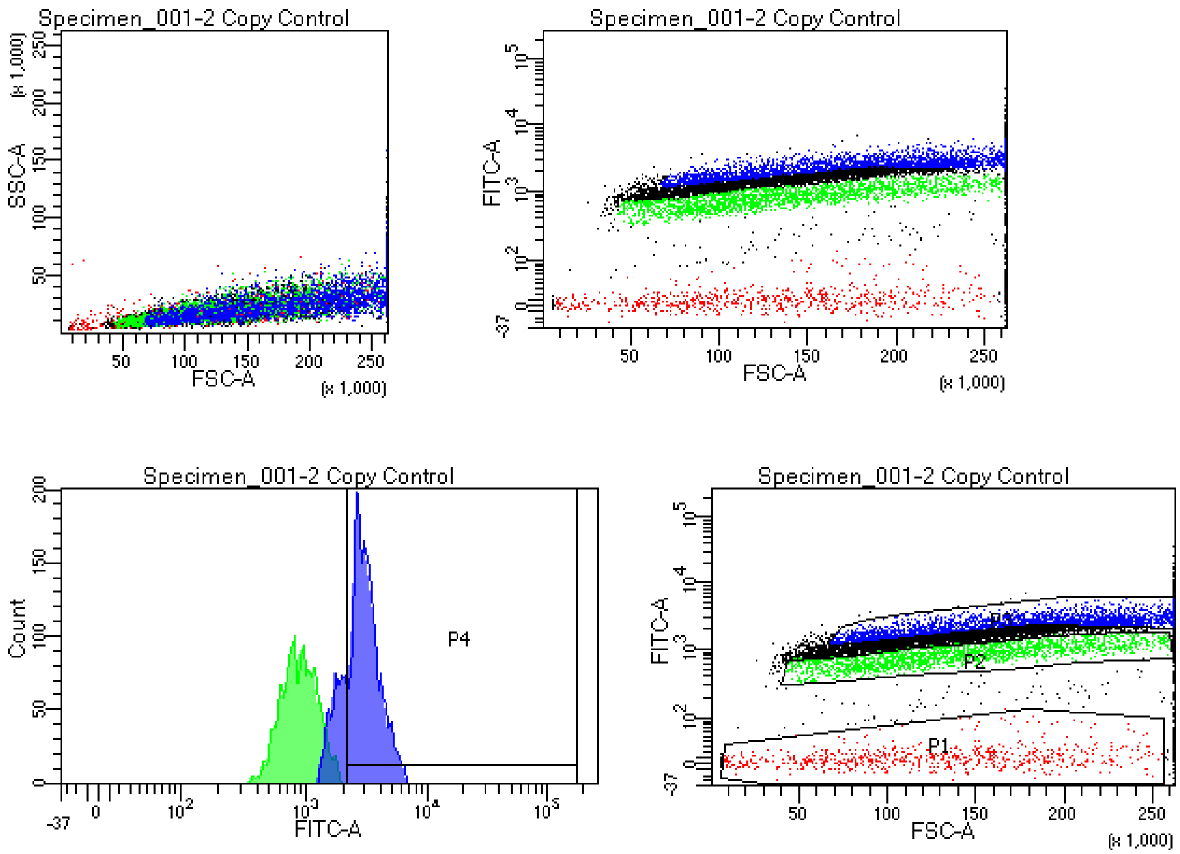

Experiment Name:	Grace 2017-08-31
Specimen Name:	Specimen_001
Tube Name:	2 Copy Control
Record Date:	Aug 31, 2017 1:52:52 PM
$OP:	Administrator

Population	#Events	%Parent
All Events	10,000	####
P1	706	7.1
P2	2,078	20.8
P3	3,240	32.4
P4	2,712	27.1

D)

BD FACSDiva 8.0.2

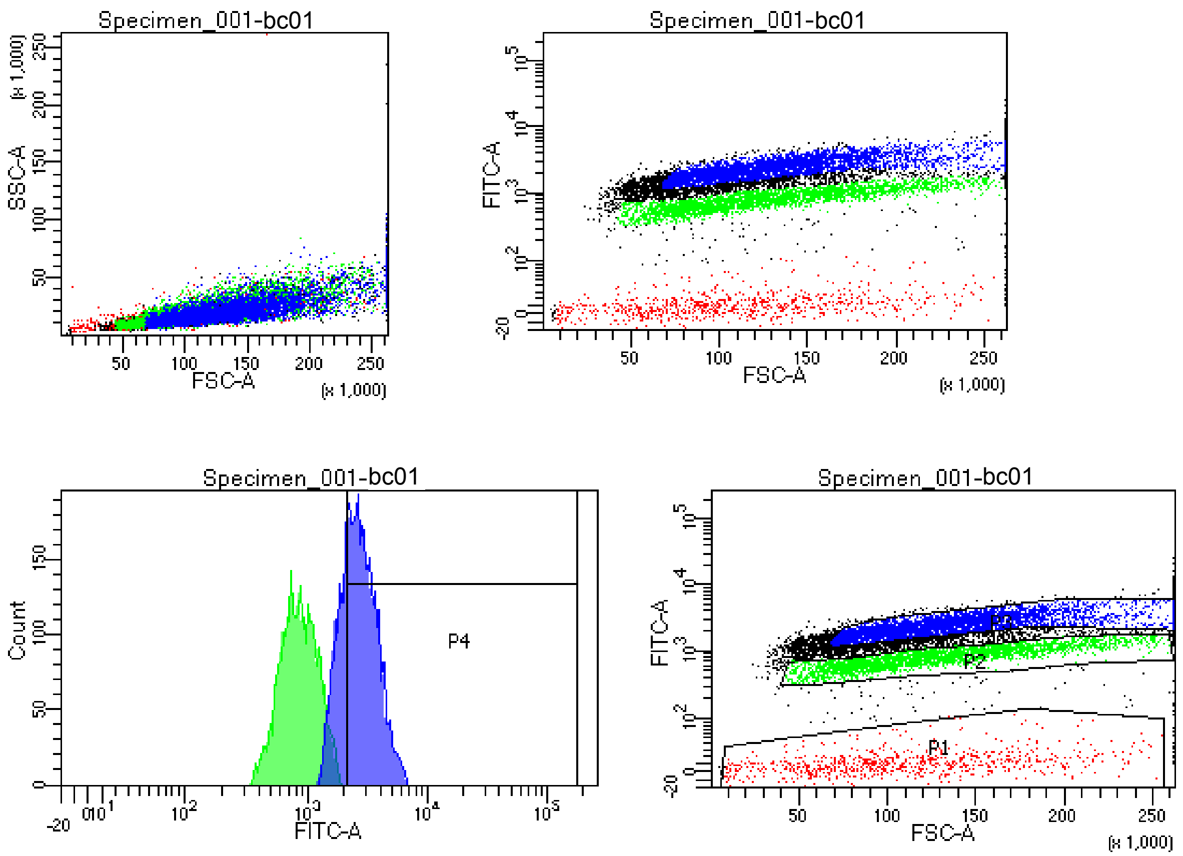

Experiment Name:	Grace 2017-08-31
Specimen Name:	Specimen_001
Tube Name:	bc01
Record Date:	Aug 31, 2017 2:03:27 PM
$OP:	Administrator

Population	#Events	%Parent
All Events	10,000	####
P1	736	7.4
P2	2,930	29.3
P3	4,029	40.3
P4	2,783	27.8

E)

BD FACSDiva 8.0.2

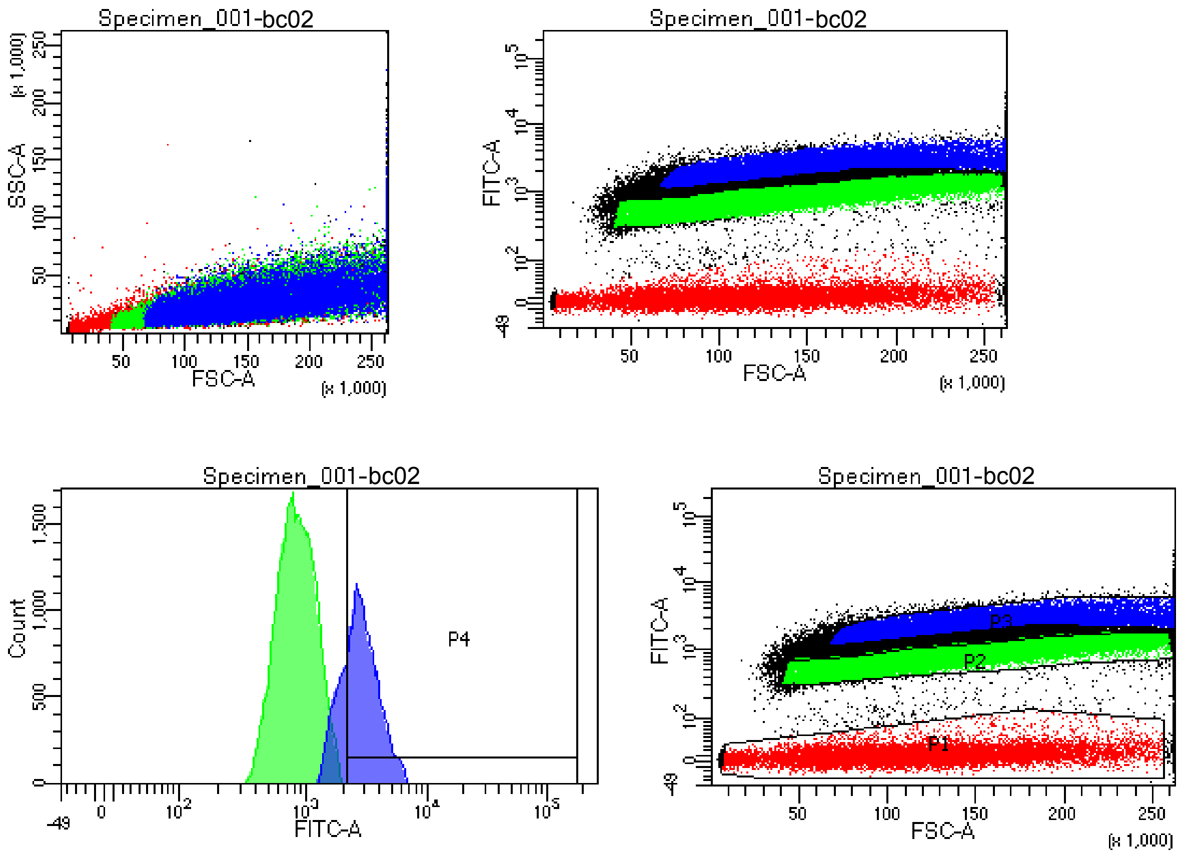

Experiment Name:	Grace 2017-08-31
Specimen Name:	Specimen_001
Tube Name:	bc02
Record Date:	Aug 31, 2017 2:53:46 PM
$OP:	Administrator

Population	#Events	%Parent
All Events	100,000	####
P1	13,493	13.5
P2	39,954	40.0
P3	23,121	23.1
P4	17,966	18.0

F)

BD FACSDiva 8.0.2

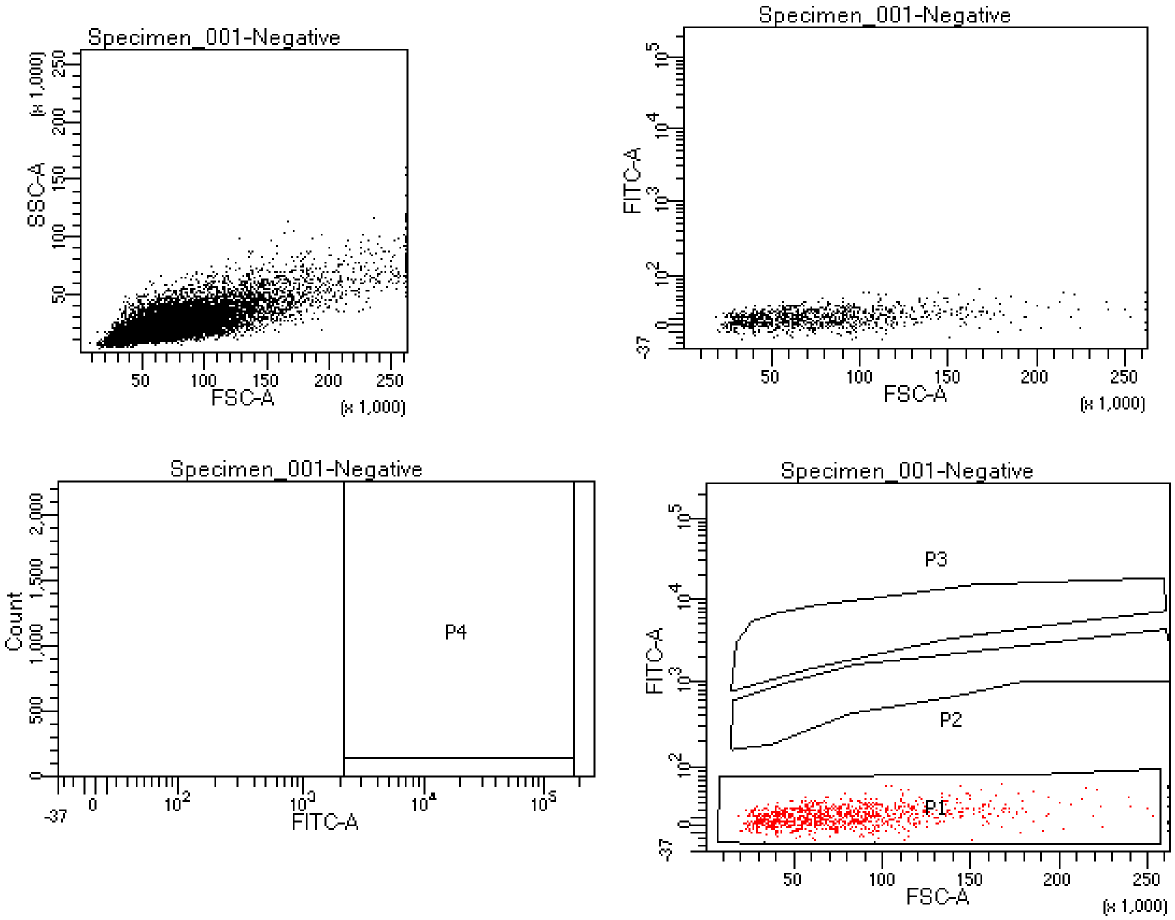

Experiment Name:	Grace 2017-09-05
Specimen Name:	Specimen_001
Tube Name:	Negative
Record Date:	Sep 5, 2017 3:29:40 PM
$OP:	Administrator

Population	#Events	%Parent
All Events	10,000	####
P1	9,904	99.0
P2	0	0.0
P3	0	0.0
P4	0	0.0

Experiment Name:	Grace 2017-09-05
Specimen Name:	Specimen_001
Tube Name:	Negative
Record Date:	Sep 5, 2017 3:29:40 PM
$OP:	Administrator
GUID:	256e4209-e736-4118-b0ce-dd4a7be...

Population	#Events	%Parent	FSC-A Mean	FSC-A Mean	FITC-A Mean	FITC-A Mean
All Events	10,000	####	####	78,585	####	10
P1	9,904	99.0	####	77,219	####	10
P2	0	0.0	####	####	####	####
P3	0	0.0	####	####	####	####
P4	0	0.0	####	####	####	####

G)

BD FACSDiva 8.0.2

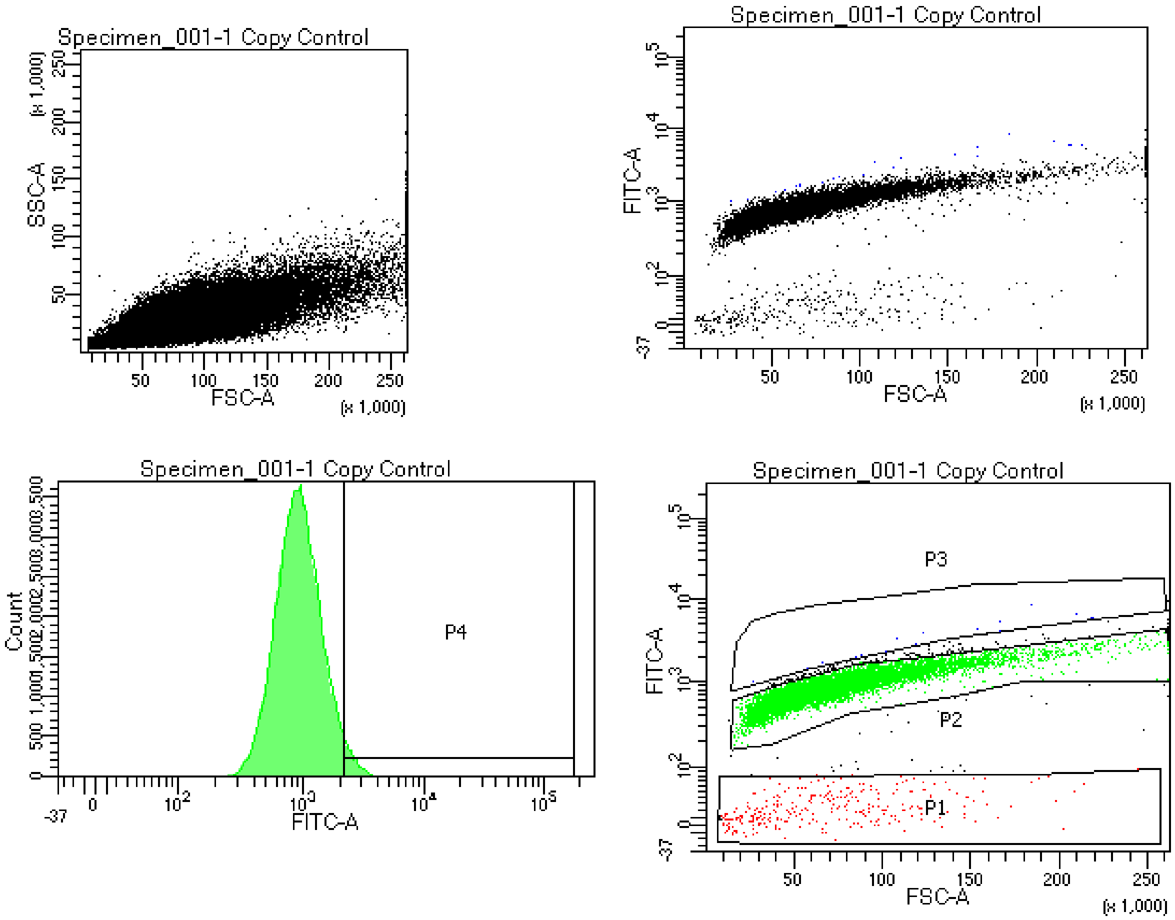

Experiment Name:	Grace 2017-09-05
Specimen Name:	Specimen_001
Tube Name:	1 Copy Control
Record Date:	Sep 5, 2017 3:30:51 PM
$OP:	Administrator

Population	#Events	%Parent
All Events	100,000	####
P1	2,775	2.8
P2	94,945	94.9
P3	151	0.2
P4	3,168	3.2

Experiment Name:	Grace 2017-09-05
Specimen Name:	Specimen_001
Tube Name:	1 Copy Control
Record Date:	Sep 5, 2017 3:30:51 PM
$OP:	Administrator
GUID:	b274fcbe-ceff-47ba-8267-f6477d7e9...

Population	#Events	%Parent	FSC-A Mean	FSC-A Mean	FITC-A Mean	FITC-A Mean
All Events	100,000	####	####	76,785	####	939
P1	2,775	2.8	####	69,014	####	23
P2	94,945	94.9	####	76,019	####	941
P3	151	0.2	####	91,456	####	2,592
P4	3,168	3.2	####	200,230	####	2,786

H)

BD FACSDiva 8.0.2

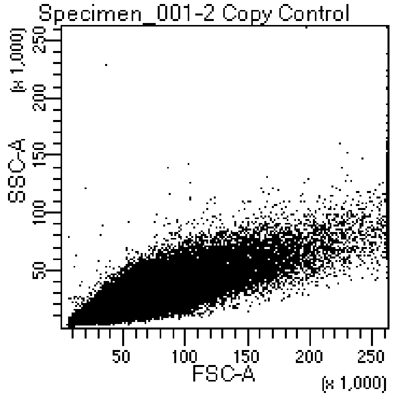

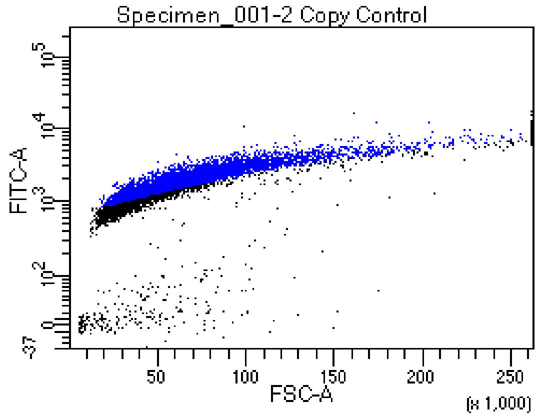

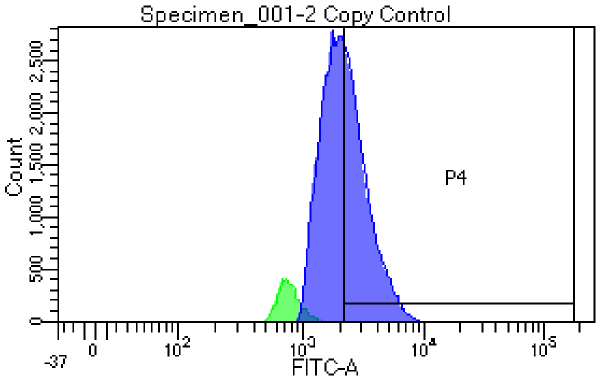

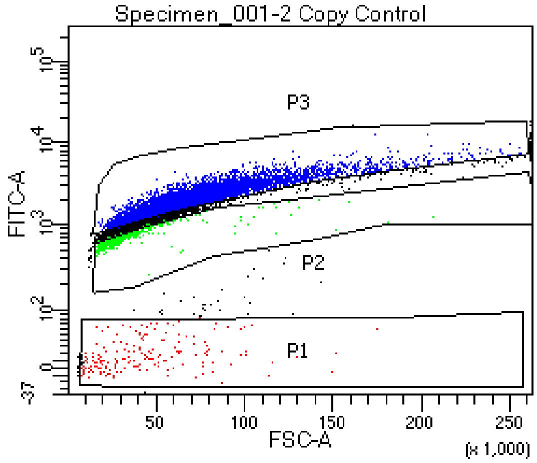

Experiment Name:	Grace 2017-09-05
Specimen Name:	Specimen_001
Tube Name:	2 Copy Control
Record Date:	Sep 5, 2017 3:38:04 PM
$OP:	Administrator

Population	#Events	%Parent
All Events	100,000	####
P1	1,999	2.0
P2	5,700	5.7
P3	71,741	71.7
P4	31,337	31.3

Experiment Name:	Grace 2017-09-05
Specimen Name:	Specimen_001
Tube Name:	2 Copy Control
Record Date:	Sep 5, 2017 3:38:04 PM
$OP:	Administrator
GUID:	1cbf163f-bd8c-4e2d-970b-b6298de5...

Population	#Events	%Parent	FSC-A Mean	FSC-A Mean	FITC-A Mean	FITC-A Mean
All Events	100,000	####	####	62,888	####	1,898
P1	1,999	2.0	####	42,321	####	17
P2	5,700	5.7	####	35,625	####	772
P3	71,741	71.7	####	69,153	####	2,203
P4	31,337	31.3	####	100,767	####	3,237

I)

BD FACSDiva 8.0.2

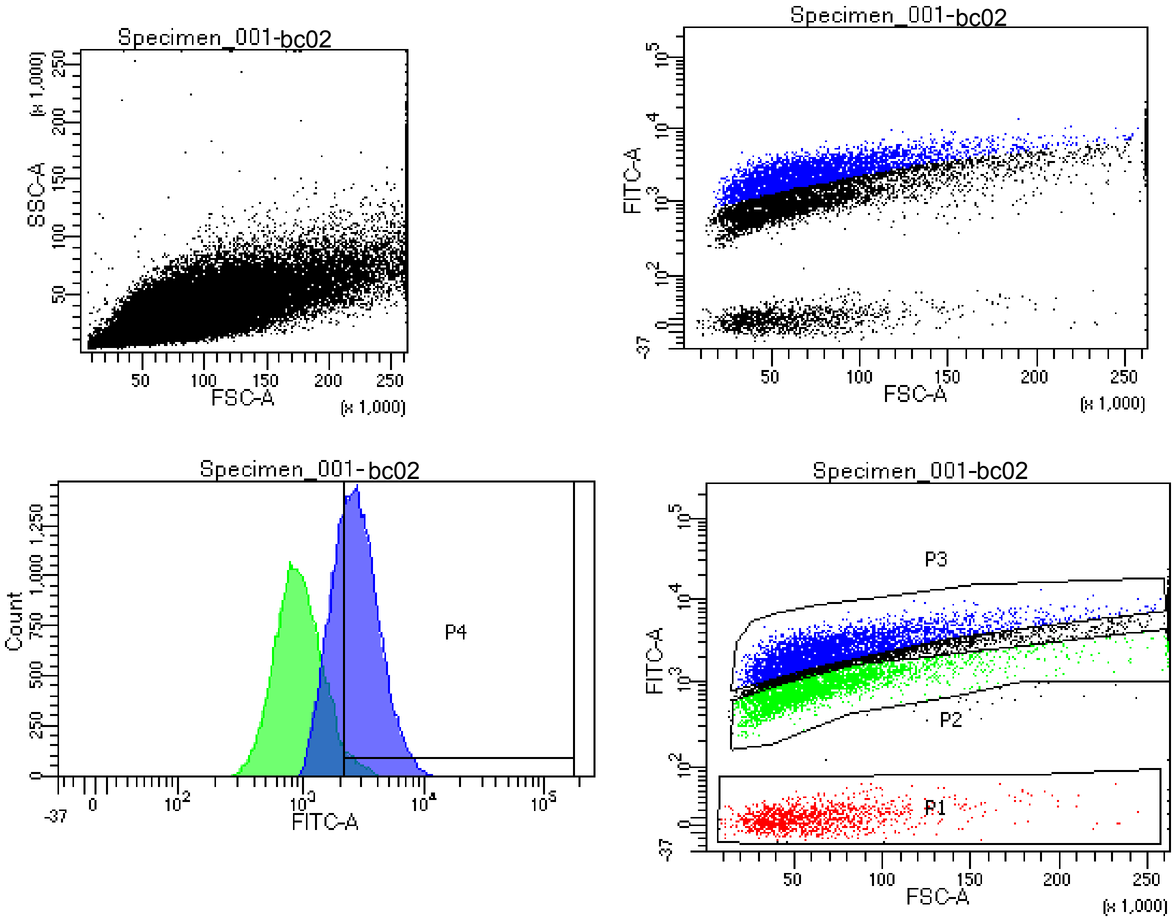

Experiment Name:	Grace 2017-09-05
Specimen Name:	Specimen_001
Tube Name:	bc02
Record Date:	Sep 5, 2017 3:52:47 PM
$OP:	Administrator

Population	#Events	%Parent
All Events	100,000	####
P1	11,939	11.9
P2	29,369	29.4
P3	40,376	40.4
P4	33,529	33.5

Experiment Name:	Grace 2017-09-05
Specimen Name:	Specimen_001
Tube Name:	bc02
Record Date:	Sep 5, 2017 3:52:47 PM
$OP:	Administrator
GUID:	fb18162a-14d9-413c-938e-4cb18e20...

Population	#Events	%Parent	FSC-A Mean	FSC-A Mean	FITC-A Mean	FITC-A Mean
All Events	100,000	####	####	77,734	####	1,826
P1	11,939	11.9	####	63,252	####	9
P2	29,369	29.4	####	69,755	####	957
P3	40,376	40.4	####	75,440	####	2,752
P4	33,529	33.5	####	111,981	####	3,523

J)

BD FACSDiva 8.0.2

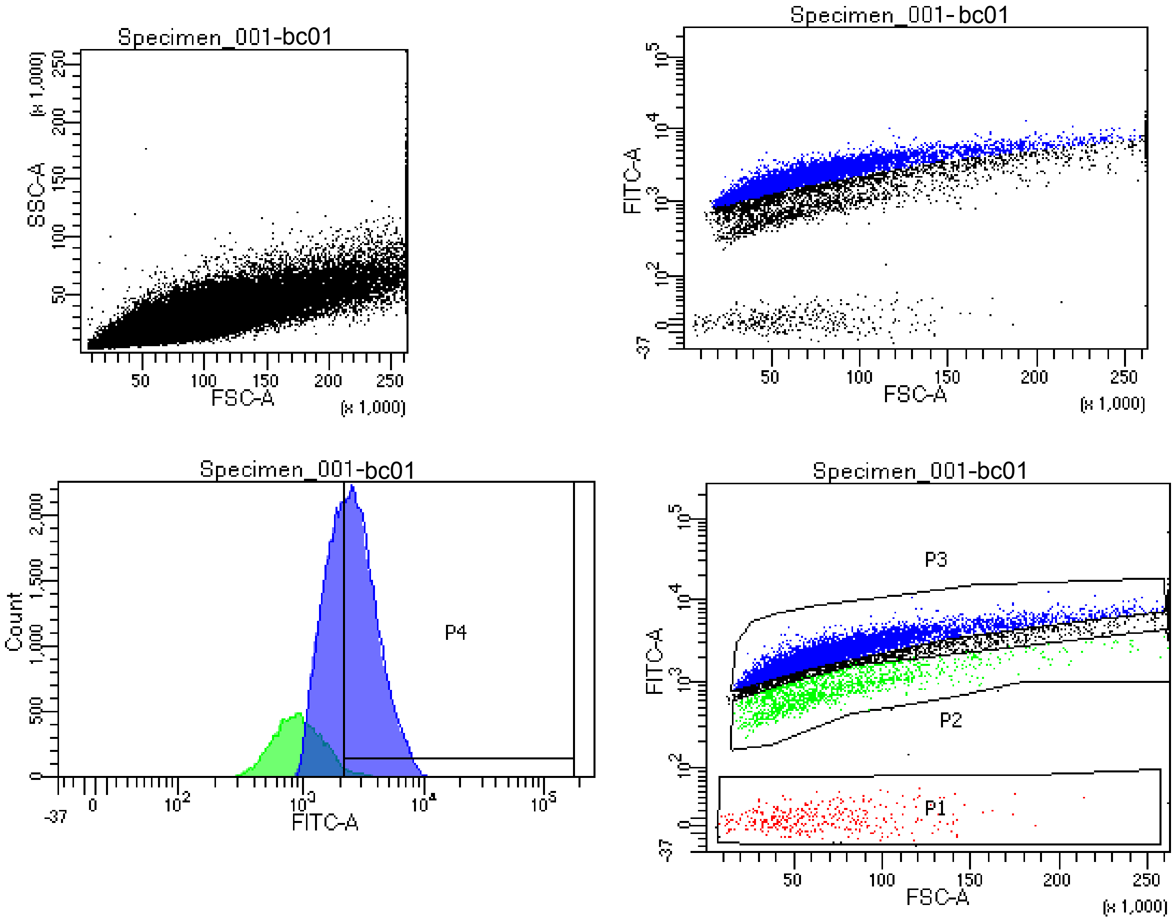

Experiment Name: Grace 2017-09-05
Specimen Name: Specimen_001
Tube Name: bc01
Record Date: Sep 5, 2017 4:03:00 PM
$OP: Administrator

Population	#Events	%Parent
All Events	100,000	####
P1	3,338	3.3
P2	14,717	14.7
P3	68,047	68.0
P4	44,843	44.8

Experiment Name: Grace 2017-09-05
Specimen Name: Specimen_001
Tube Name: bc01
Record Date: Sep 5, 2017 4:03:00 PM
$OP: Administrator
GUID: 7ad9a62f-e86f-45a4-b39b-da13c3d9...

Population	#Events	%Parent	FSC-A Mean	FSC-A Mean	FITC-A Mean	FITC-A Mean
All Events	100,000	####	####	81,365	####	2,298
P1	3,338	3.3	####	63,898	####	8
P2	14,717	14.7	####	72,601	####	965
P3	68,047	68.0	####	76,605	####	2,580
P4	44,843	44.8	####	115,623	####	3,584

K)

BD FACSDiva 8.0.2

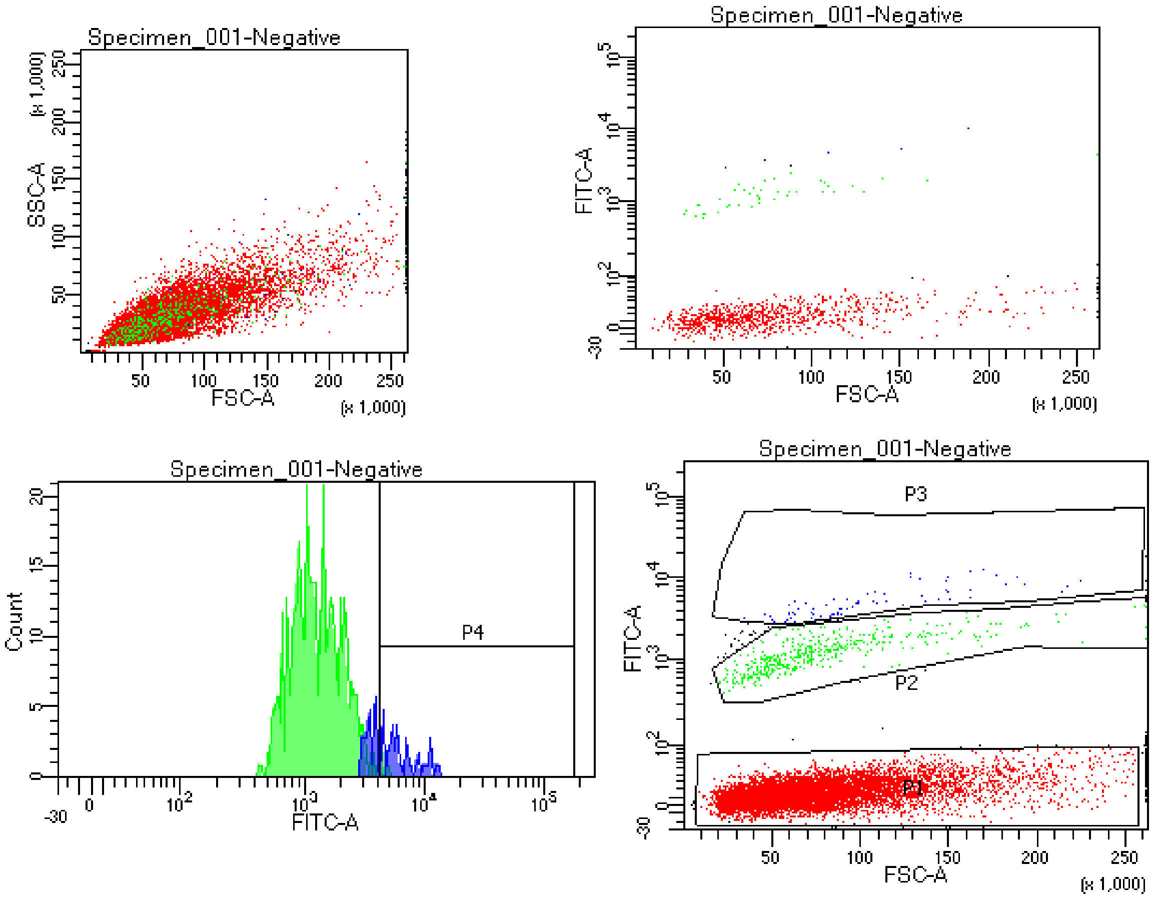

Experiment Name:	Grace 2017-09-05_001
Specimen Name:	Specimen_001
Tube Name:	Negative
Record Date:	Sep 19, 2017 12:46:27 PM
$OP:	Administrator

Population	#Events	%Parent
All Events	10,000	####
P1	9,308	93.1
P2	449	4.5
P3	75	0.8
P4	52	0.5

Experiment Name:	Grace 2017-09-05_001
Specimen Name:	Specimen_001
Tube Name:	Negative
Record Date:	Sep 19, 2017 12:46:27 PM
$OP:	Administrator
GUID:	65ddcd06-d65c-428b-b71a-5d0aedc...

Population	#Events	%Parent	FSC-A Mean	FITC-A Mean
All Events	10,000	####	77,217	124
P1	9,308	93.1	74,907	14
P2	449	4.5	76,959	1,335
P3	75	0.8	101,918	5,289
P4	52	0.5	148,122	6,800

L)

BD FACSDiva 8.0.2

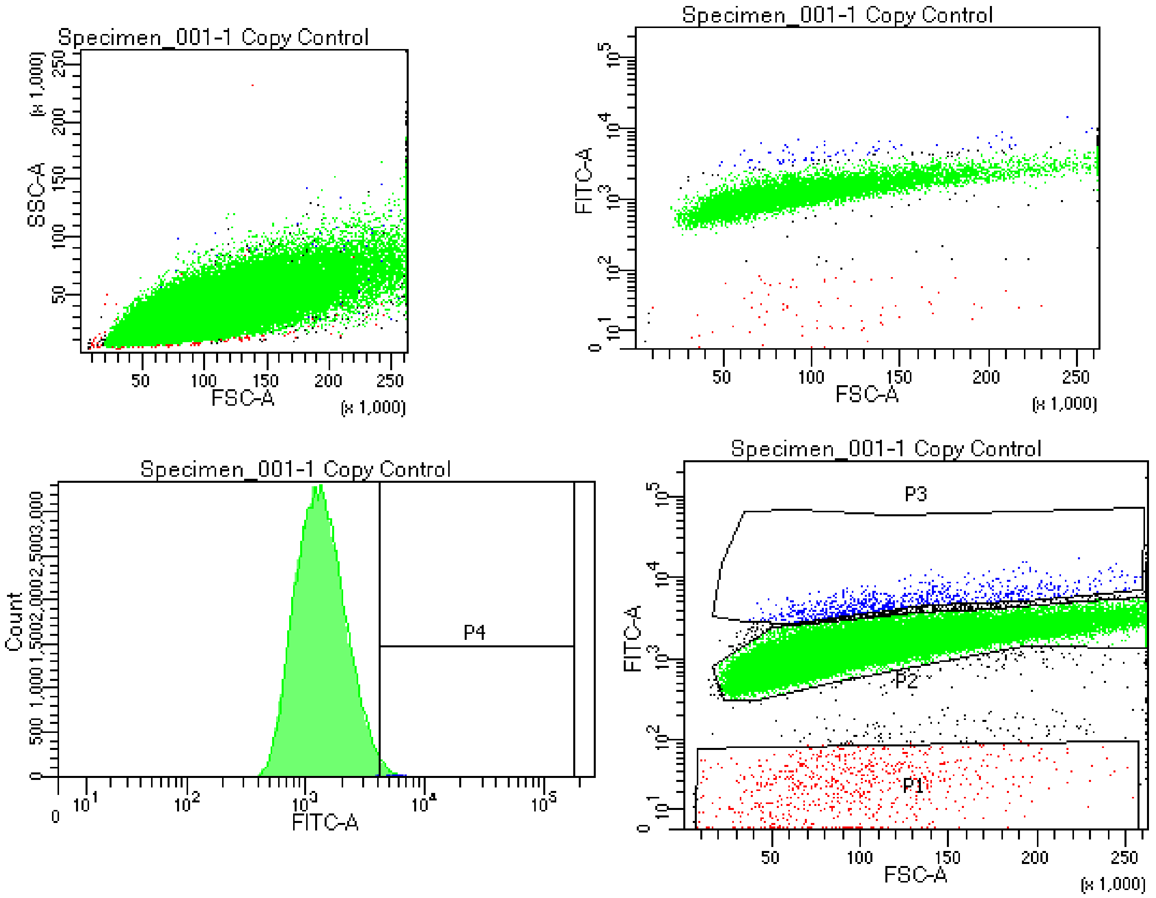

Experiment Name:	Grace 2017-09-05_001
Specimen Name:	Specimen_001
Tube Name:	1 Copy Control
Record Date:	Sep 19, 2017 12:48:24 PM
$OP:	Administrator

Population	#Events	%Parent
All Events	100,000	####
P1	701	0.7
P2	97,576	97.6
P3	799	0.8
P4	1,745	1.7

Experiment Name:	Grace 2017-09-05_001
Specimen Name:	Specimen_001
Tube Name:	1 Copy Control
Record Date:	Sep 19, 2017 12:48:24 PM
$OP:	Administrator
GUID:	18653049-a86c-4f23-bf7a-6a6a6b5fa...

Population	#Events	%Parent	FSC-A Mean	FITC-A Mean
All Events	100,000	####	101,053	1,454
P1	701	0.7	96,666	30
P2	97,576	97.6	100,216	1,407
P3	799	0.8	127,481	5,567
P4	1,745	1.7	212,920	5,801

M)

BD FACSDiva 8.0.2

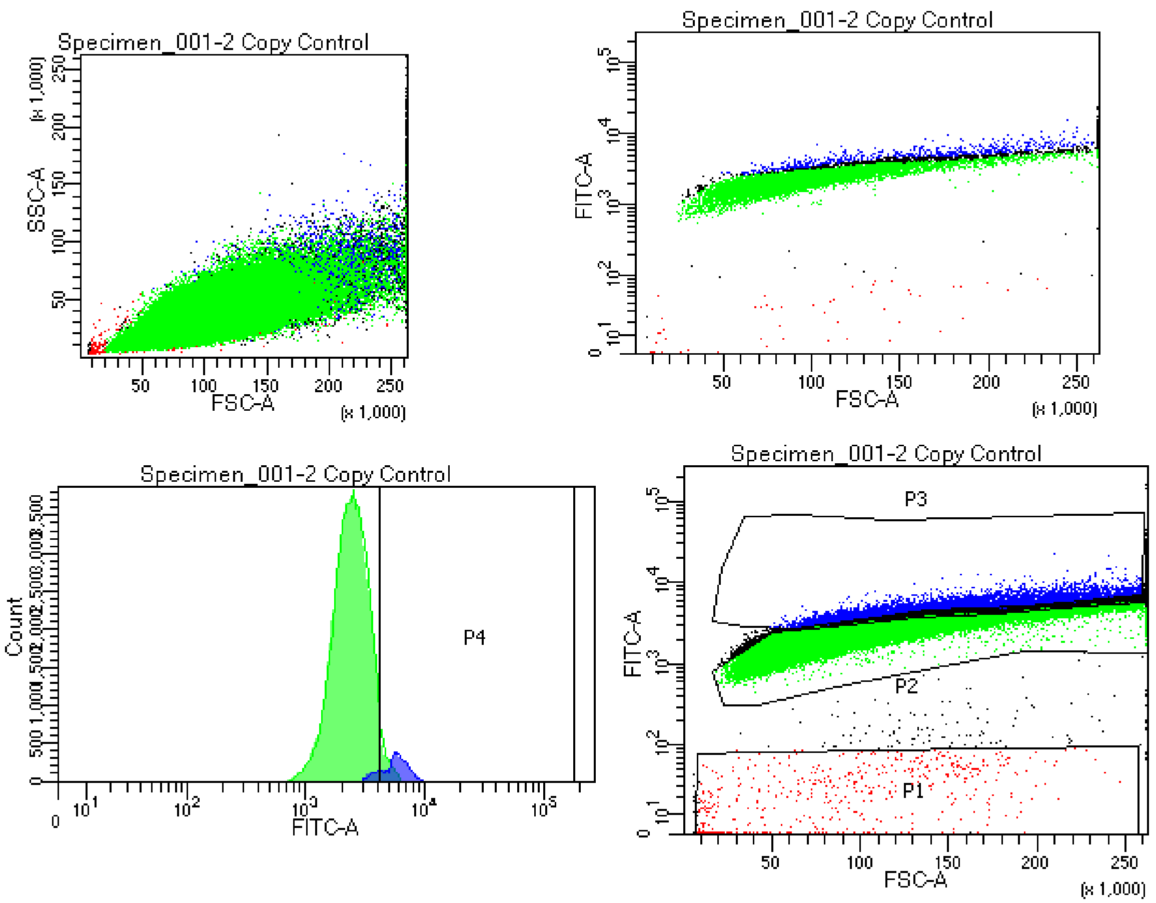

Experiment Name:	Grace 2017-09-05_001
Specimen Name:	Specimen_001
Tube Name:	2 Copy Control
Record Date:	Sep 19, 2017 12:50:08 PM
$OP:	Administrator

Population	#Events	%Parent
All Events	100,000	####
P1	485	0.5
P2	81,860	81.9
P3	5,765	5.8
P4	14,351	14.4

Experiment Name:	Grace 2017-09-05_001
Specimen Name:	Specimen_001
Tube Name:	2 Copy Control
Record Date:	Sep 19, 2017 12:50:08 PM
$OP:	Administrator
GUID:	b08136f7-74ad-4b4d-815c-df9b6ef41...

Population	#Events	%Parent	FSC-A Mean	FITC-A Mean
All Events	100,000	####	111,527	2,861
P1	485	0.5	90,507	29
P2	81,860	81.9	99,271	2,364
P3	5,765	5.8	157,567	5,425
P4	14,351	14.4	202,182	5,894

N)

BD FACSDiva 8.0.2

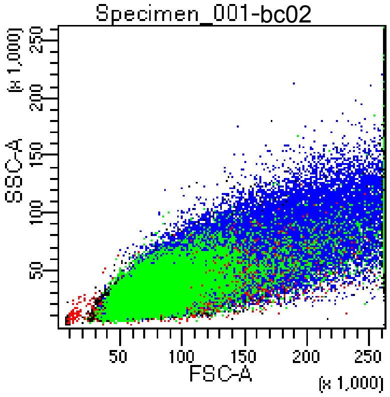

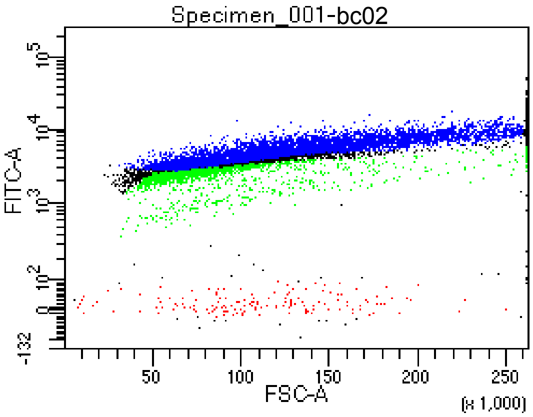

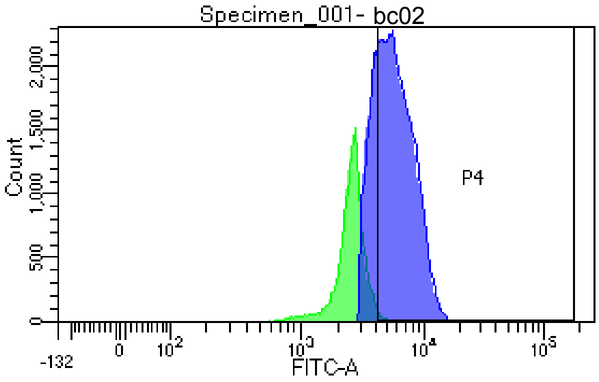

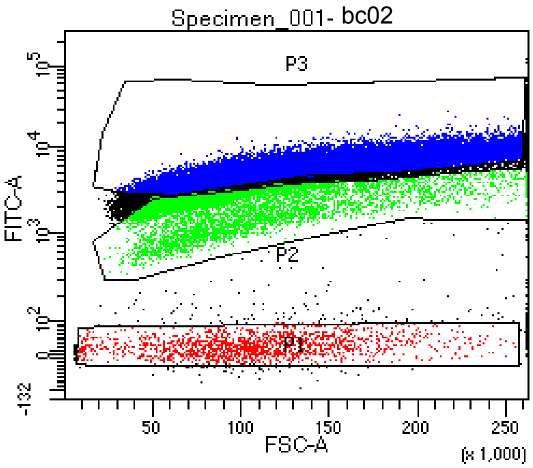

Experiment Name:	Grace 2017-09-05_001
Specimen Name:	Specimen_001
Tube Name:	bc02
Record Date:	Sep 19, 2017 1:03:24 PM
$OP:	Administrator

Population	#Events	%Parent
All Events	100,000	####
P1	1,626	1.6
P2	17,839	17.8
P3	57,182	57.2
P4	51,708	51.7

Experiment Name:	Grace 2017-09-05_001
Specimen Name:	Specimen_001
Tube Name:	bc02
Record Date:	Sep 19, 2017 1:03:24 PM
$OP:	Administrator
GUID:	0ebca134-bb28-4452-a588-0e834f4d...

Population	#Events	%Parent	FSC-A Mean	FITC-A Mean
All Events	100,000	####	128,149	5,254
P1	1,626	1.6	108,927	25
P2	17,839	17.8	83,969	2,383
P3	57,182	57.2	130,960	5,571
P4	51,708	51.7	170,826	7,512

O)

BD FACSDiva 8.0.2

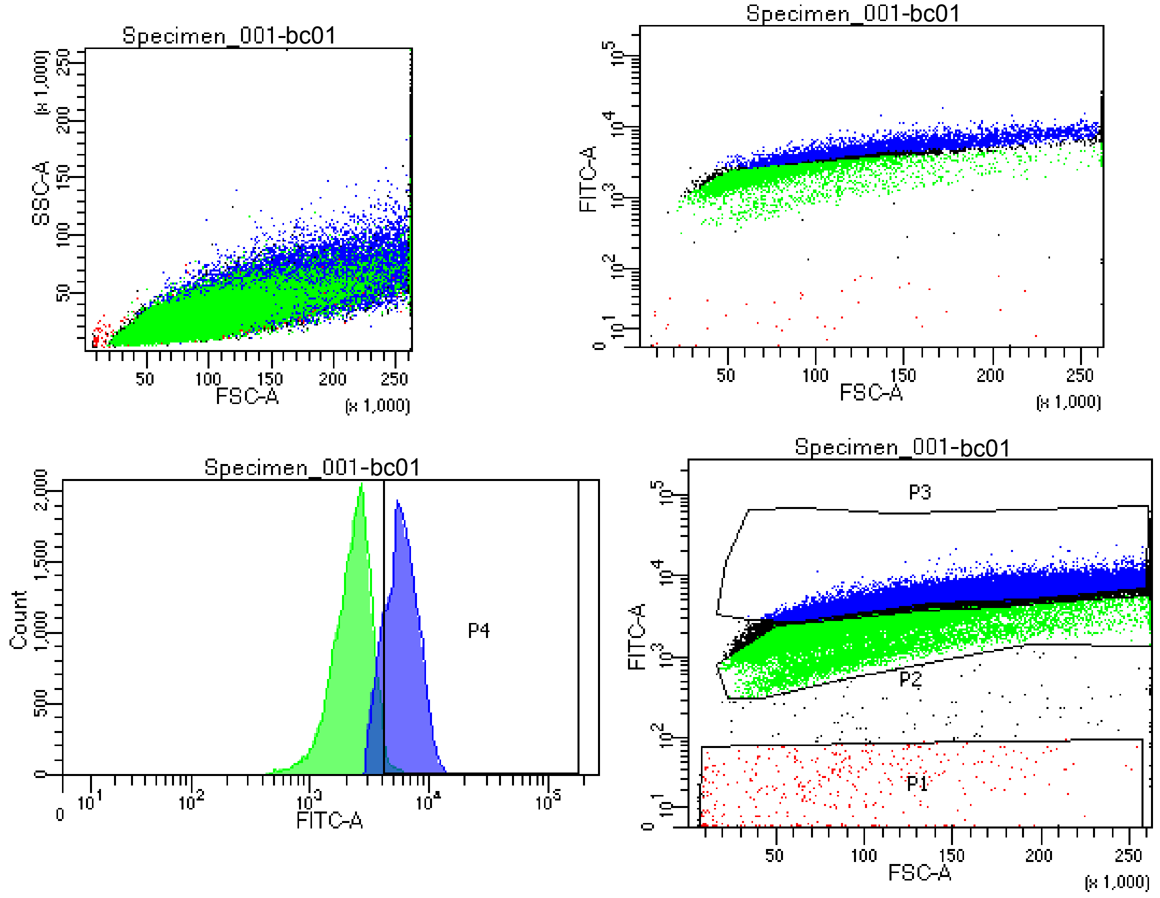

Experiment Name:	Grace 2017-09-05_001
Specimen Name:	Specimen_001
Tube Name:	bc01
Record Date:	Sep 19, 2017 1:13:11 PM
$OP:	Administrator

Population	#Events	%Parent
All Events	100,000	####
P1	382	0.4
P2	40,088	40.1
P3	37,055	37.1
P4	42,072	42.1

Experiment Name:	Grace 2017-09-05_001
Specimen Name:	Specimen_001
Tube Name:	bc01
Record Date:	Sep 19, 2017 1:13:11 PM
$OP:	Administrator
GUID:	7c4f41a3-3ac3-43a5-869b-76149b9c...

Population	#Events	%Parent	FSC-A Mean	FITC-A Mean
All Events	100,000	####	126,459	4,448
P1	382	0.4	87,626	31
P2	40,088	40.1	85,821	2,223
P3	37,055	37.1	147,748	5,731
P4	42,072	42.1	181,620	7,074

P)

BD FACSDiva 8.0.2

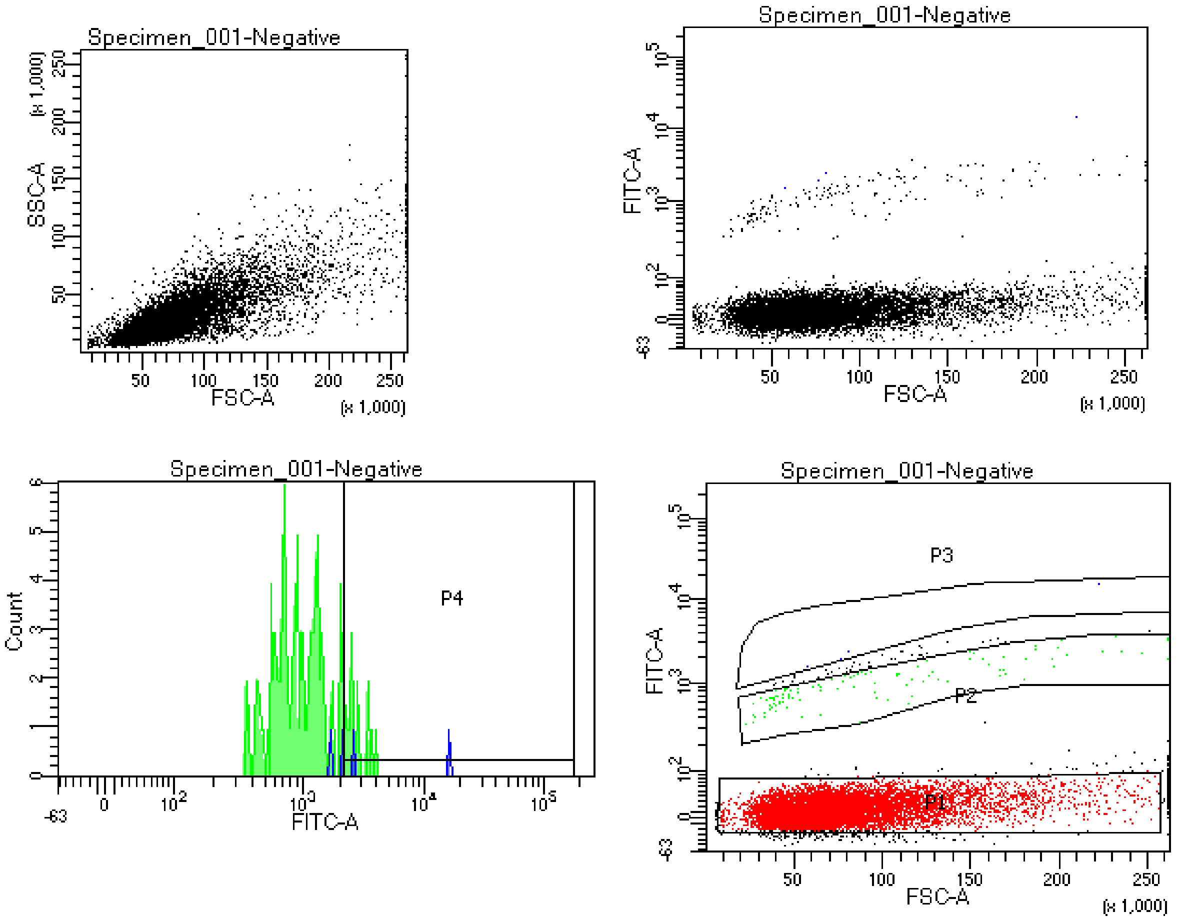

Experiment Name: Grace 2017-10-17
Specimen Name: Specimen_001
Tube Name: Negative
Record Date: Oct 17, 2017 12:35:19 PM
$OP: Administrator

Population	#Events	%Parent
All Events	10,000	####
P1	9,429	94.3
P2	108	1.1
P3	4	0.0
P4	32	0.3

Experiment Name: Grace 2017-10-17
Specimen Name: Specimen_001
Tube Name: Negative
Record Date: Oct 17, 2017 12:35:19 PM
$OP: Administrator
GUID: 5eedb4d7-bbf7-40d3-8c37-1c40777a...

Population	#Events	%Parent	FSC-A Mean	FITC-A Mean
All Events	10,000	####	80,455	38
P1	9,429	94.3	78,843	15
P2	108	1.1	97,041	1,157
P3	4	0.0	108,869	5,317
P4	32	0.3	167,384	3,155

Q)

BD FACSDiva 8.0.2

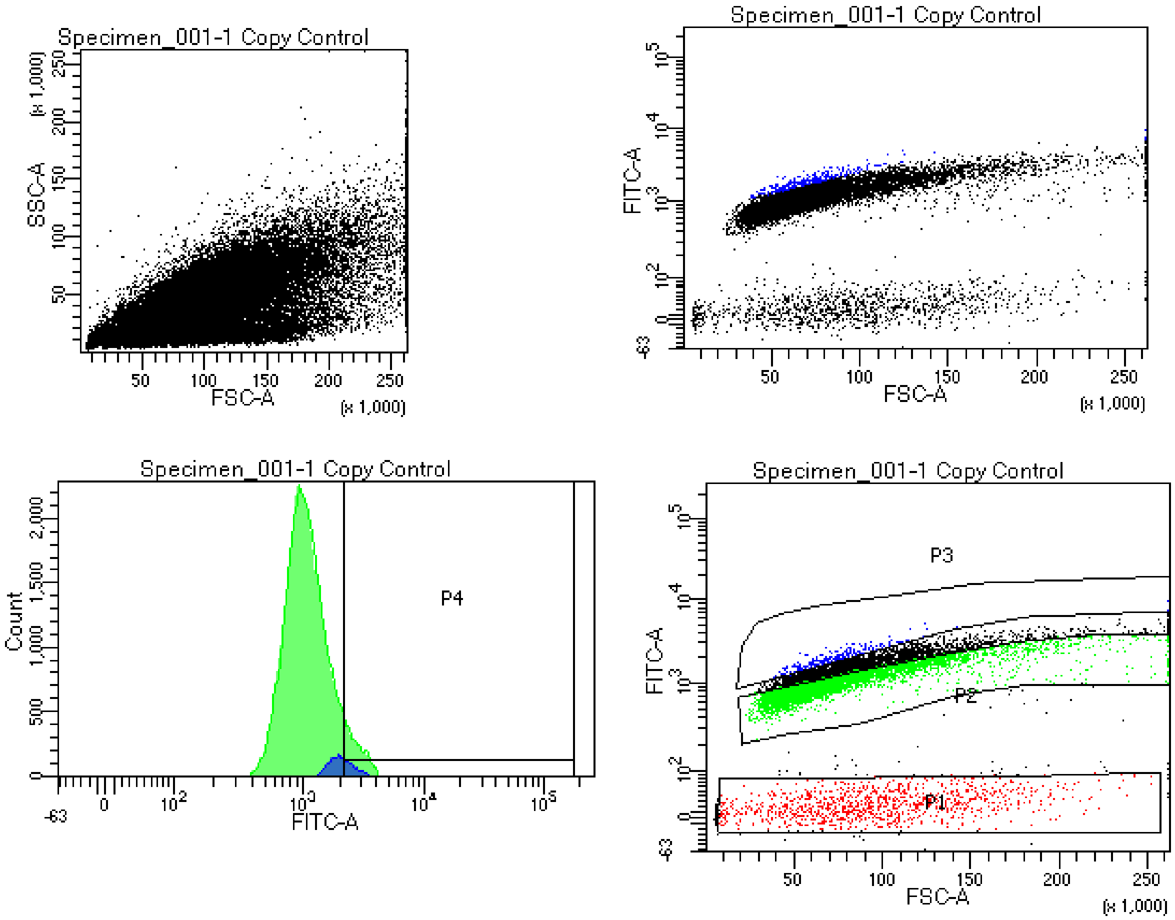

Experiment Name:	Grace 2017-10-17
Specimen Name:	Specimen_001
Tube Name:	1 Copy Control
Record Date:	Oct 17, 2017 12:36:45 PM
$OP:	Administrator

Population	#Events	%Parent
All Events	100,000	####
P1	10,287	10.3
P2	53,066	53.1
P3	2,669	2.7
P4	11,478	11.5

Experiment Name:	Grace 2017-10-17
Specimen Name:	Specimen_001
Tube Name:	1 Copy Control
Record Date:	Oct 17, 2017 12:36:45 PM
$OP:	Administrator
GUID:	1c58b535-c384-4236-8a22-a3b3f0ea...

Population	#Events	%Parent	FSC-A Mean	FITC-A Mean
All Events	100,000	####	85,056	1,234
P1	10,287	10.3	95,216	23
P2	53,066	53.1	80,010	1,128
P3	2,669	2.7	73,831	2,118
P4	11,478	11.5	151,552	2,823

R)

BD FACSDiva 8.0.2

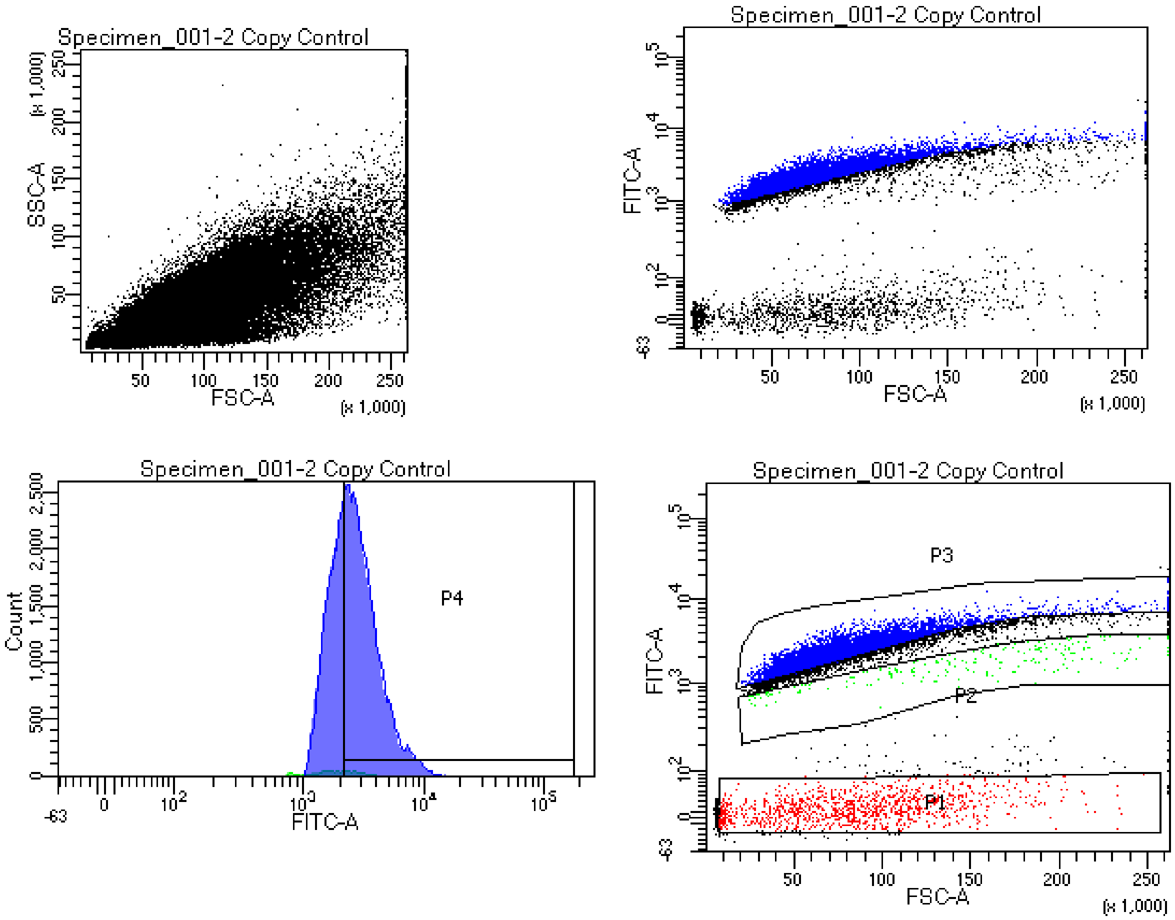

Experiment Name:	Grace 2017-10-17
Specimen Name:	Specimen_001
Tube Name:	2 Copy Control
Record Date:	Oct 17, 2017 12:37:57 PM
$OP:	Administrator

Population	#Events	%Parent
All Events	100,000	####
P1	11,085	11.1
P2	2,104	2.1
P3	68,115	68.1
P4	49,311	49.3

Experiment Name:	Grace 2017-10-17
Specimen Name:	Specimen_001
Tube Name:	2 Copy Control
Record Date:	Oct 17, 2017 12:37:57 PM
$OP:	Administrator
GUID:	47ebd015-ecab-4e1b-89fc-7d21bc89...

Population	#Events	%Parent	FSC-A Mean	FITC-A Mean
All Events	100,000	####	84,178	2,328
P1	11,085	11.1	81,280	17
P2	2,104	2.1	147,923	1,783
P3	68,115	68.1	79,618	2,741
P4	49,311	49.3	108,183	3,559

S)

BD FACSDiva 8.0.2

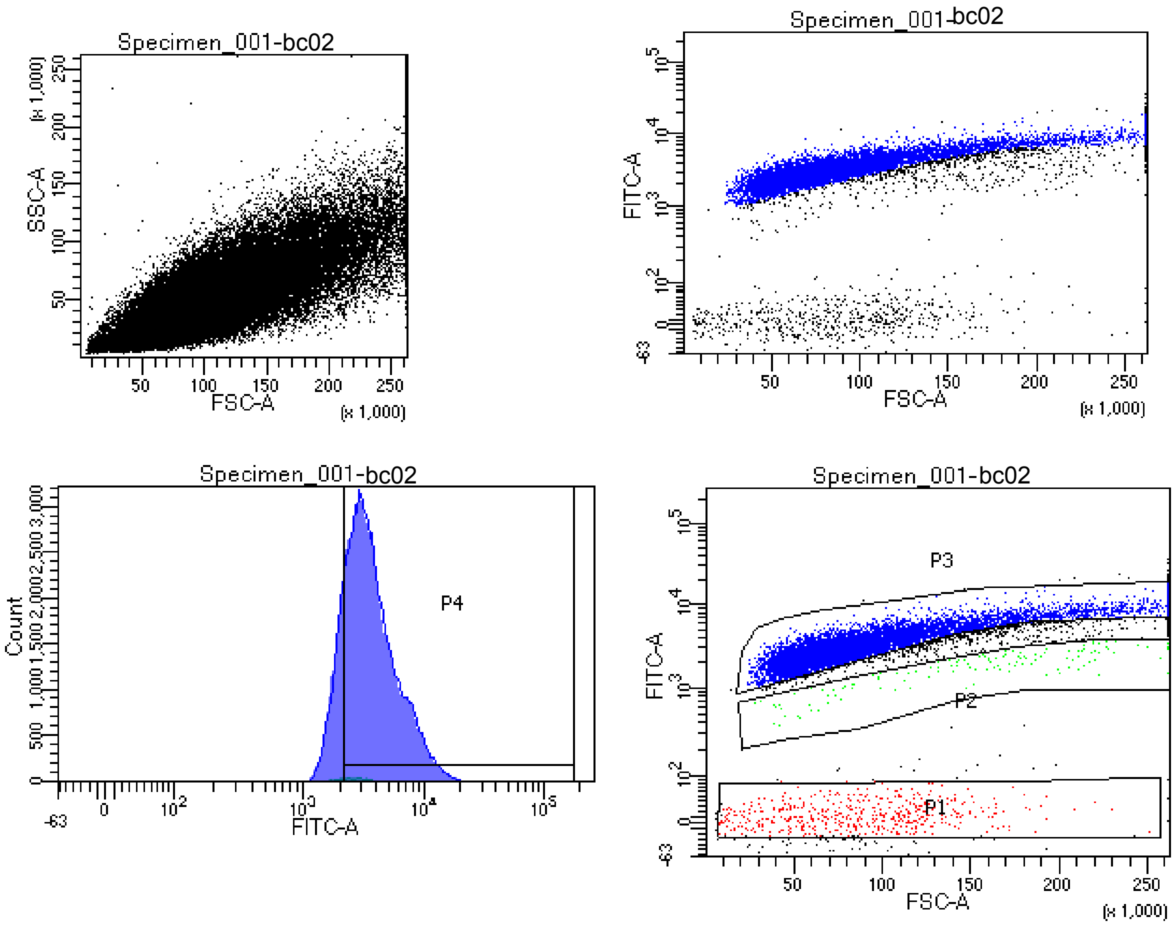

Experiment Name:	Grace 2017-10-17
Specimen Name:	Specimen_001
Tube Name:	bc02
Record Date:	Oct 17, 2017 12:50:58 PM
$OP:	Administrator

Population	#Events	%Parent
All Events	100,000	####
P1	5,073	5.1
P2	1,455	1.5
P3	86,734	86.7
P4	75,646	75.6

Experiment Name:	Grace 2017-10-17
Specimen Name:	Specimen_001
Tube Name:	bc02
Record Date:	Oct 17, 2017 12:50:58 PM
$OP:	Administrator
GUID:	52ad62b0-fd39-440f-98ad-32e02a10f...

Population	#Events	%Parent	FSC-A Mean	FITC-A Mean
All Events	100,000	####	96,203	3,544
P1	5,073	5.1	90,265	14
P2	1,455	1.5	157,290	1,977
P3	86,734	86.7	91,870	3,756
P4	75,646	75.6	107,318	4,264

T)

BD FACSDiva 8.0.2

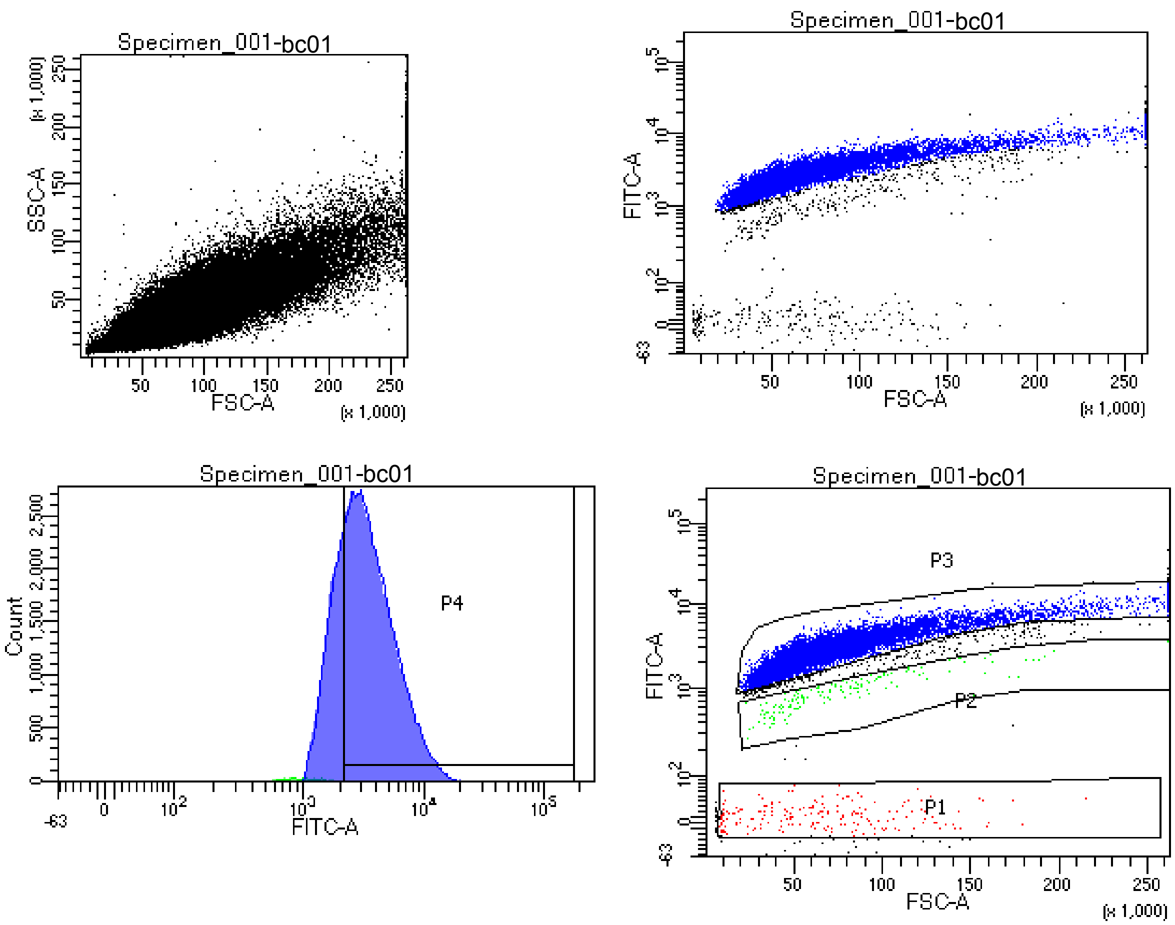

Experiment Name:	Grace 2017-10-17
Specimen Name:	Specimen_001
Tube Name:	bc01
Record Date:	Oct 17, 2017 1:10:29 PM
$OP:	Administrator

Population	#Events	%Parent
All Events	100,000	####
P1	1,912	1.9
P2	1,484	1.5
P3	92,764	92.8
P4	68,500	68.5

Experiment Name:	Grace 2017-10-17
Specimen Name:	Specimen_001
Tube Name:	bc01
Record Date:	Oct 17, 2017 1:10:29 PM
$OP:	Administrator
GUID:	4fda0ad0-304d-419a-9f2c-74fb87462...

Population	#Events	%Parent	FSC-A Mean	FITC-A Mean
All Events	100,000	####	80,551	3,357
P1	1,912	1.9	65,353	14
P2	1,484	1.5	85,487	1,121
P3	92,764	92.8	80,044	3,484
P4	68,500	68.5	96,820	4,226
